# Vaginal Microbiome and Epithelial Gene Array in Post-Menopausal Women with Moderate to Severe Dryness

**DOI:** 10.1371/journal.pone.0026602

**Published:** 2011-11-02

**Authors:** Ruben Hummelen, Jean M. Macklaim, Jordan E. Bisanz, Jo-Anne Hammond, Amy McMillan, Rebecca Vongsa, David Koenig, Gregory B. Gloor, Gregor Reid

**Affiliations:** 1 Canadian Research & Development Centre for Probiotics, Lawson Health Research Institute, London, Canada; 2 Department of Public Health, Erasmus MC, University Medical Centre Rotterdam, Rotterdam, The Netherlands; 3 Biochemistry, The University of Western Ontario, London, Canada; 4 Microbiology and Immunology, and Surgery, The University of Western Ontario, London, Canada; 5 Family Medicine, The University of Western Ontario, London, Canada; 6 Kimberly Clark Corporation, Corporate Research and Engineering-Microbial Control, Neenah, Wisconsin, United States of America; Baylor College of Medicine, United States of America

## Abstract

After menopause, many women experience vaginal dryness and atrophy of tissue, often attributed to the loss of estrogen. An understudied aspect of vaginal health in women who experience dryness due to atrophy is the role of the resident microbes. It is known that the microbiota has an important role in healthy vaginal homeostasis, including maintaining the pH balance and excluding pathogens. The objectives of this study were twofold: first to identify the microbiome of post-menopausal women with and without vaginal dryness and symptoms of atrophy; and secondly to examine any differences in epithelial gene expression associated with atrophy. The vaginal microbiome of 32 post-menopausal women was profiled using Illumina sequencing of the V6 region of the 16S rRNA gene. Sixteen subjects were selected for follow-up sampling every two weeks for 10 weeks. In addition, 10 epithelial RNA samples (6 healthy and 4 experiencing vaginal dryness) were acquired for gene expression analysis by Affymetrix Human Gene array. The microbiota abundance profiles were relatively stable over 10 weeks compared to previously published data on premenopausal women. There was an inverse correlation between *Lactobacillus* ratio and dryness and an increased bacterial diversity in women experiencing moderate to severe vaginal dryness. In healthy participants, *Lactobacillus iners* and *L. crispatus* were generally the most abundant, countering the long-held view that lactobacilli are absent or depleted in menopause. Vaginal dryness and atrophy were associated with down-regulation of human genes involved in maintenance of epithelial structure and barrier function, while those associated with inflammation were up-regulated consistent with the adverse clinical presentation.

## Introduction

The onset of menopause is accompanied by a dramatic increase in reported symptoms of vaginal dryness, soreness, irritation or itching, pain with intercourse and bleeding after intercourse [1]. Collectively these symptoms affect 25–50% post-menopausal women [2], [3] and significantly impact their quality of life. Vulvovaginal atrophy (VVA) is somewhat of a catchment term for these symptoms and is diagnosed by an assessment of vaginal dryness, irritation, soreness, and dyspareunia with urinary frequency, urgency, incontinence, and the presence of pale and dry vulvovaginal mucosa with petechiae, along with pH>4.6 [4]. Though the use of estrogen replacement therapy (ERT) can rapidly reverse some of these changes [5], a large proportion of women are hesitant to use estrogens, even when applied topically [3]. Interestingly, a proportion of women appear to remain symptom free, long after the onset of menopause.

A lactobacilli-dominated vaginal microbiota is associated with retention of health, but with menopause it has long been assumed that ERT is required to maintain the dominant lactobacilli and reduce the risk of infection [5]. This is supported by studies of the vaginal microbiota using denaturing gel electrophoresis [6], [7]. Next-generation deep sequencing provides a means to uncover the extent of bacterial presence in a given niche, including the vagina [8]–[10].

The aim of the present study was to identify the vaginal microbiome of post-menopausal women who were clinically healthy or had vaginal dryness, and examine any differences in epithelial gene expression.

## Results

The reported and observed vaginal symptoms are presented in Figure S1. The observed vaginal dryness correlated well with observed atrophy (*r* ^2^ = 0.84) supporting vaginal dryness as an appropriate proxy for the occurrence of vaginal atrophy. Moderate to severe scores were in agreement for three out of the four measures that included of dryness, discoloration, blanching and petechiae in 11 participants.

**Figure 1:**
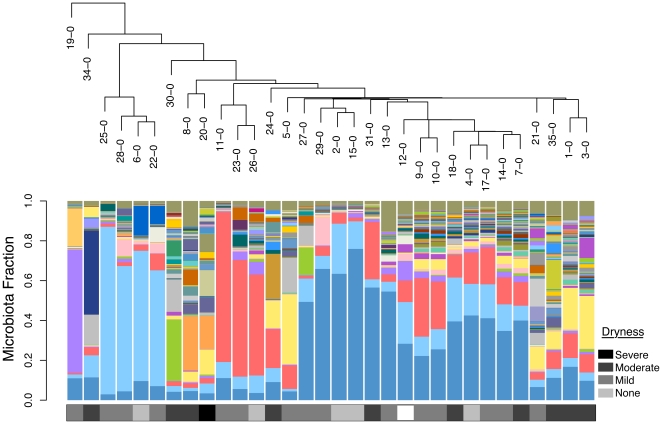
Microbiota profiles for 32 post-menopausal women clustered by biota similarity. Each bar represents a single vaginal sample, and the colored segments represent the relative fraction of each bacterial taxon detected at 1% relative abundance or greater in any one sample. Sequences at less than 1% abundance have been included in the “remainder” fraction at the top of the bar (see color legend of bacterial taxa). The microbiota are clustered by similarity as represented in the dendogram above. The sample name (participant ID-time point) is labeled in the dendogram and corresponds to the bar below. The dryness score as observed by the examining nurse is represented below each microbiota bar.

Illumina sequencing resulted in 1,758,430 total V6 reads after quality filtering, with an average of 17410±9917 reads per sample (range: 2,123–53,054). Figure 1 shows the microbiota findings for the 32 participants. Using a cutoff of 1% abundance in any sample, there were 119 distinct operational taxonomic units (OTUs) detected, each representing a bacterial taxa (Table S1).

**Figure 2:**
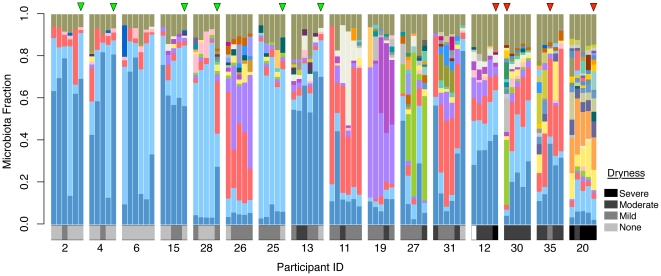
Time-series microbiota profiles for 16 post-menopausal sampled every 2 weeks. Each bar represents a single vaginal sample, and each cluster of bars is a single participant (starting at time 0 and sampled every 2 weeks for up to 10 weeks total). The colored segments represent the relative fraction of each bacterial taxon detected at 1% relative abundance or greater. Sequences at less than 1% abundance have been included in the “remainder” fraction at the top of the bar (see color legend of bacterial taxa). The dryness score as observed by the examining nurse is represented below each microbiota bar. Sample time points that were included in the microarray analysis are marked with an arrowhead: the first six green arrows are controls (no or mild dryness), and the last four red arrows are women experiencing moderate to severe dryness.

Sixteen of the 32 participants were selected at their initial visit and followed for microbiota samples every 2 weeks for 10 weeks total. This time series showed that the microbiome abundance profiles did not fluctuate greatly within a subject (Figure 2). When a linear mixed effect model was applied to the Shannon's diversity index data, a statistical linear trend was not observed (p-value = 0.364) indicating that the abundance of microbial diversity from week to week did not change significantly within a subject. The biotas of women who were least symptomatic (‘none’ or ‘mild’ dryness score) had low bacterial diversity with a dominance of lactobacilli (Figure 1 and 2). Conversely, women with ‘moderate’ or ‘severe’ dryness had a decreased abundance of lactobacilli and a large diversity in number of species detected. In this latter group, there was a high representation of *Prevotella timonensis* (OTU_6), *Porphyromonas* (OTU_9), *Peptoniphilus* (OTU_27), and *Bacillus* (OTU_34) using a Mann-Whitney-Wilcoxen rank-sum test (Figure S2). A weighted least squares model was applied to the data and a statistical linear trend was observed (p-value = 0.00141) indicating an inverse correlation between *Lactobacillus* ratio and dryness.

Lactobacilli were dominant in many women, irrespective of age (participant 13 age 74 versus participant 6 age 42), and the absence of ERT. We noted lactobacilli at an abundance of ≥10% in 29/32 (91%) women at time zero, while 17/32 (53%) had lactobacilli as the overall dominating organism (greater than 50% relative abundance).

In order to perform human genomic array analysis, one sample was collected at random from each subject. Based upon successful high-quality RNA isolation, 10 resulting samples were divided into healthy controls and subjects experiencing dryness based upon their observed dryness scores (Figure 2).

**Figure 3:**
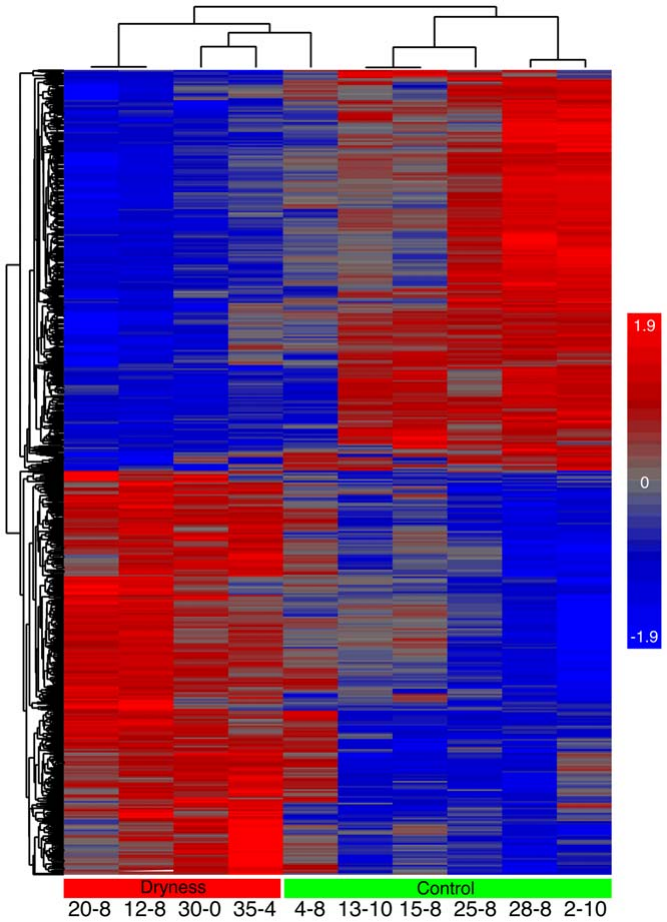
Heatmap of vaginal epithelial gene expression of 10 samples. A clustered heatmap of differential gene expression (>2-fold change, *p*<0.05) between the control and dryness groups. Samples are labeled as participant number-time point (week). Samples assigned to the dryness or control groups based on physiological examination of the vagina are well separated by gene expression differences with exception of 4–8 which has an intermediate gene expression profile and clusters closer to the dryness group.

After importing intensity data into Partek GS 6.5, an ANOVA was applied to identify differentially expressed genes between the control and dryness groups. The list of genes was filtered to only include those differentially expressed by at least 2-fold (*p*<0.05), which yielded a list of 960 probe sets used for subsequent comparative analyses (Table S2). A heatmap of the filtered list (Figure 3) shows two main branches separating the dryness and control groups. Participant 4 had an intermediate gene expression profile between the dryness and control groups despite having no self-reported or observed signs of dryness and correspondingly no additional signs of atrophy.

Then, the top 20 genes in either direction with the greatest fold change between the dryness and control groups were further analyzed (Table 1) to determine where the most dramatic changes occurred. The most up-regulated genes in the dryness group were MMP7 (13.75-fold), and the choline transporter SLC44A4 which was interrogated by three separate probe sets, all up-regulated 9.19-fold with identical *p*-values. The most down-regulated genes in the dryness group were SPINK7 (−38.65-fold) and TGM3 (−21.86-fold).

**Table 1:** The top 20 genes up- and down- regulated genes (*p*<0.05) in the vaginal dryness group compared to controls.

Gene Symbol	Gene Assignment	RefSeq ID	*p*-value	Fold Change
MMP7	matrix metallopeptidase 7 (matrilysin, uterine)	NM_002423	7.73E-03	13.75
SLC44A4	solute carrier family 44, member 4	NM_025257	2.23E-02	9.19
SLC44A4	solute carrier family 44, member 4	NM_025257	2.23E-02	9.19
SLC44A4	solute carrier family 44, member 4	NM_025257	2.23E-02	9.19
CFH	complement factor H	NM_000186	4.79E-03	8.72
PIGR	polymeric immunoglobulin receptor	NM_002644	2.01E-02	8.64
IL19	interleukin 19	NM_153758	3.25E-02	8.31
	Transcript ID: 8158684		9.50E-03	8.28
	Transcript ID: 8180303		2.26E-02	7.73
CFB	complement factor B	NM_001710	4.69E-02	7.33
PLAT	plasminogen activator, tissue	NM_000930	2.23E-02	7.19
	Transcript ID: 8138487		2.08E-03	7.10
CFB	complement factor B	NM_001710	4.78E-02	6.93
WFDC2	WAP four-disulfide core domain 2	NM_006103	8.32E-03	6.61
CXCL6	chemokine (C-X-C motif) ligand 6	NM_002993	1.81E-02	6.50
TSPAN1	tetraspanin 1	NM_005727	2.46E-02	6.30
AGR2	anterior gradient homolog 2 (Xenopus laevis)	NM_006408	1.50E-02	6.18
TRIM31	tripartite motif-containing 31	NM_007028	4.77E-02	5.85
ASS1	argininosuccinate synthetase 1	NM_000050	3.05E-03	5.70
PLAC8	placenta-specific 8	NM_016619	2.03E-02	5.51
SPINK7	serine peptidase inhibitor, Kazal type 7 (putative)	NM_032566	4.81E-04	−38.65
TGM3	transglutaminase 3	NM_003245	4.24E-05	−21.86
SBSN	Suprabasin, part of a gene complex including dermokine	NM_198538	3.28E-05	−20.93
ALOX12	arachidonate 12-lipoxygenase	NM_000697	4.43E-04	−19.61
KPRP	keratinocyte proline-rich protein	NM_001025231	2.30E-04	−17.43
GYS2	glycogen synthase 2 (liver)	NM_021957	1.66E-03	−16.78
DSG1	desmoglein 1	NM_001942	5.02E-03	−16.61
LCE3E	late cornified envelope 3E	NM_178435	6.78E-04	−15.35
LCE3D	late cornified envelope 3D	NM_032563	3.27E-03	−14.61
SERPINB12	serpin peptidase inhibitor, clade B	NM_080474	1.42E-03	−14.11
PNLIPRP3	pancreatic lipase-related protein 3	NM_001011709	3.06E-04	−13.69
KRTDAP	keratinocyte differentiation-associated protein	NM_207392	2.16E-03	−13.08
CLDN17	claudin 17	NM_012131	1.99E-03	−12.88
KRT1	keratin 1	NM_006121	1.16E-03	−11.99
LOC441178	hypothetical LOC441178	AL832737	4.66E-03	−10.89
LOC441178	hypothetical LOC441178	AL832737	4.66E-03	−10.89
ME1	malic enzyme 1, NADP(+)-dependent, cytosolic	NM_002395	1.32E-03	−10.57
	Transcript ID: 8020347		1.70E-03	−10.26
CRCT1	cysteine-rich C-terminal 1	NM_019060	3.86E-03	−9.80
CRISP3	cysteine-rich secretory protein 3	NM_006061	8.76E-03	−9.75

To assess the relationship of differentially expressed genes, a GeneOntology (GO) enrichment analysis of the filtered list of 960 probe sets was performed using Partek GS 6.5. The top 10 GO-functions enriched are displayed in Table 2, and demonstrate changes in epithelial remodeling and immune response. Cornified envelope had the highest enrichment score with 62.50% (10 genes) in this process and was down-regulated in the dryness group.

**Table 2:** Enrichment of GO terms in the filtered gene list (n = 960).

Function	GO ID	Enrichment Score	Enrichment *p*-value	No. of Genes	% of total genes in GO term group differentially expressed
Cornified envelope	1533	21.81	3.37E-10	10	62.50
Keratinocyte differentiation	30216	20.01	2.04E-09	11	47.83
Immune response	6955	19.20	4.60E-09	38	13.52
Cell differentiation	30154	17.96	1.59E-08	24	17.91
Epidermis development	8544	17.55	2.38E-08	17	23.94
Tissue development	9888	17.48	2.55E-08	23	18.11
Cellular developmental process	48869	15.29	2.28E-07	30	13.45
Extracellular region	5576	13.99	8.37E-07	37	11.28
Epithelial cell differentiation	30855	13.12	2.01E-06	6	60.00
Innate immune response	45087	13.03	2.18E-06	11	26.83

## Discussion

The microbiota of this set of post-menopausal women was quite stable with one out of a possible 62 transitions between normal and BV Nugent scores, unlike another study of pre-menopausal women that observed 226 transitions out of a possible 1365 opportunities (*p* = 0.003) [11]. If indeed further studies confirm that the microbial profiles of post-menopausal women are relatively stable, perhaps it is due to lack of menstruation which is known to cause stage-dependent variations in the microbiota [12].

The Illumina sequencing method identified double or triple the number of bacterial types per subject compared to previous gel electrophoresis methods [7]. Similar to recent deep sequencing studies of pre-menopausal women [8]–[10], [13], [14], we noted *Lactobacillus iners* and *Gardnerella vaginalis* were universally present and possibly represent core members in this niche. However, there were much lower abundances (<1%) of *Mobiluncus*, *Staphylococcus*, *Sneathia*, *Bifidobacterium*, and *Gemella* in our study set of post-menopausal women. This could be due to our smaller sample size rather than micro-environmental changes associated with menopause, and so the physiological significance is not known. As more information emerges about the different organisms in the microbiota, the types of clones and virulence properties of them, for example differences in expression of various toxins and mucus-degrading enzymes by *G. vaginalis* [15], the better will be our understanding of which strains equate to health versus disease.

Vaginal dryness, a condition that is associated with significant changes in the transcription of genes associated with cellular structure and immune function, as shown here by the human microarray data, is a symptom of atrophy which afflicts large numbers of menopausal women and has been linked with depletion of estrogen [16]. The muscular layer of the vaginal wall tends to be thicker in postmenopausal than premenopausal women, while the epithelial layer is much thinner [17]. Having developed a method to use Affymetrix arrays to read epithelial cell gene expression changes in the vagina [18], we applied this here and found down-regulation of genes involved in maintaining proliferation and barrier function of the vaginal epithelium in the dryness group. Without a cornified envelope, epithelial cells are much more sensitive to drying, chemical agents and microbial infection [19]. Many of the genes in this group encode structural components of the cornified envelope, so lowered expression in the dryness group implies reduced structural integrity of the tissue. This is mirrored in the down-regulation of adhesion molecules such as SBSN and DSG1, implicated in maintaining proper epithelial barrier function [20], [21]. Similarly, ALOX 12 (−19.61-fold) has also been shown to be a positive regulator of epidermal barrier function [22], and keratin is an important structural molecule in the epidermis imparting mechanical stability to the tissue [23].

The most down-regulated gene in the dryness group was SPINK7 (alias ECRG2), a member of the SPINK family of proteases, which is known to regulate invasion and migration by preventing extracellular matrix (ECM) degradation through inhibiting the action of urokinase-type plasminogen activator [24]. Matrilysin-1 protein (MMP) 7 opposes the SPINK family proteins, and its up-regulation by 13.75-fold and cleaves ECM components and up-regulate inflammation [25]. Thus, it is possible that the combination of increased ECM degradation with decreased inhibition of protease inhibitors contributes to vaginal thinning.

Both complement factors B and H (CFB and CFH) and other components of the complement system were up-regulated in the dryness group. Since dryness is accompanied by inflammation, this suggests that the up-regulation of the regulatory molecules (CFH and CFI), although perhaps capable of protecting host cells, are not sufficient to dampen the inflammatory response. This potential for dysregulation of the complement system in vaginal dryness warrants further study. The transition from a lactobacilli dominated microbiota to one dominated by Gram negative anaerobes, *Atopobium* and Gram positive cocci could play a role in the inflammatory process, but further studies would be needed to prove the correlation in this environment rich in immune mediators [26].

Differential expression of many cytokines and chemokines, the messengers of inflammation and chemotaxis, was noted. Three CXCL-family chemokines, known to recruit monocytes and leukocytes to the vaginal epithelium, were observed to be up-regulated 4.11 to 6.5 fold in the dryness group. Polymeric immunoglobulin receptor (PIGR) was up-regulated 8.64-fold in the dryness group; indicative of increased mucosal immune activity.

Two highly up-regulated genes, SLC44A4 and PLAT, could be associated with the symptoms of vaginal dryness and atrophy. The SLC44A4 gene product is a sodium-dependent transmembrane transport protein involved in the uptake of choline in neurons, which is used in neurons in the production of acetylcholine, which is one of the main neurotransmitters of the parasympathic nervous system [27]. As the vaginal epithelium is rich in nerve endings and these can increase in density with reduced estrogen, it is possible that increased activity of this transporter may contribute to the sensation of pain through cholinergic nerve endings. The PLAT gene product is a serine protease which converts plasminogen to plasmin, and functions in cell migration and tissue remodeling as well as reducing blood clotting. Up-regulation of this gene may play a role in the increased bleeding and tissue sensitivity associated with vaginal dryness.

Our gene array findings agree with data obtained by Cotreau et al. [28] on vaginal biopsies from 19 women with vulvovaginal atrophy (VVA) before and after treatment with 17β-estradiol. The similarity in results suggests that many of the genes being differentially regulated between dryness and healthy women may be as a result of differential estrogen levels between the groups. This is further supported by the up-regulation of one of the vaginal epithelial cornified envelope genes (LCE3D) following estrogen replacement therapy [29], that was 14-fold down-regulated in women with vaginal dryness.

In summary, the vaginal microbiota of 32 post-menopausal women identified 119 OTUs and subset analysis showed little fluctuation over time, unlike that of pre-menopausal women reported previously. There was an inverse correlation between *Lactobacillus* ratio and dryness, a condition commonly found after menopause, which shown here to be associated with changes in vaginal epithelial cell integrity and inflammation.

## Methods

### Study population

The institutional review board of the University of Western Ontario, London, Canada, approved the protocol. The study, albeit not a clinical trial, was nevertheless registered at clinicaltrials.gov with ID# NCT01084616 for full disclosure.

From a practice with over 500 post-menopausal women, a total of 32 between ages 42 and 77 were identified and recruited at the Victoria Family Medical Center in London, Canada between January and February 2010, and followed until June 2010. Participants were recruited to the study by their physician (Jo-Anne Hammond), or information sheets posted in doctor's offices. All had at least 12 months of amenorrhea, had not used antibiotics or had any vaginal infection one month prior to screening, had not used systemically estrogen products 6 months prior to sampling, or topical estrogen containing product one week before sampling. Women were excluded if they had used douches or lubricants in the past week.

After giving informed consent, each participant completed a questionnaire on demographics, history of vaginal or urinary tract symptoms or infections, and a self-assessment of the severity of five vaginal symptoms: vaginal dryness, vaginal irritation or itching, pain during urination not associated with infection, vaginal soreness, pain during sexual intercourse, bleeding after sexual intercourse. Symptoms were rated as none, mild, moderate or severe. Pain with intercourse and bleeding after intercourse could be scored as “not applicable”.

A gynecologic examination was performed by a nurse trained to detect dryness and atrophy to provide an ‘observed’ assessment of vaginal color, dryness, blanching and integrity of tissue, vaginal tissue petechiae, and overall vaginal atrophy. Vaginal pH was measured using the pHem-Alert indicator (Gynex, Wyoming, USA). A cytobrush (Cytobrush plus GT, Cooper Surgical Inc. USA) was used to collect epithelial cells from the mid-vagina for human mRNA analysis. The cytobrush was immediately placed in a 1.5 ml sterile RNase-free microtube containing 700 µl RNA*later* stabilization agent (Applied Biosystems, Austin, Texas, USA) and stored at 4°C within 4 hours of collection. RNA extraction occurred within 72 hours of collection. Two polyester Dacron swabs were used to sample the mid-vagina for microbial analysis: one was archived at −80°C for later bacterial DNA extraction, and the other was rolled on a microscope glass slide and scored for bacterial vaginosis (BV) using the Nugent criteria [30]. Sixteen participants with moderate to severe vaginal dryness were followed every 2 weeks for up to 10 weeks total.

### Bacterial DNA extraction, amplification, and sequencing

Bacterial DNA was extracted using Instagene (Bio-Rad) and the V6 region of the 16 s rRNA gene was PCR amplified as described previously [9] using barcoded primers: L-V6 (5′-CAACGCGARGAACCTTACC-3′) and R-V6 (5′-ACAACACGAGCTGACGAC-3′). Based on the intensity of the ethidium bromide-stained band on an agarose gel, an approximate equimolar ratio of PCR products were mixed together to give the final sample sent for Illumina paired-end sequencing at The Centre for Applied Genomics in Toronto, Canada.

### OTU clustering and taxonomic assignment

Sequence filtering, processing, and microbial analysis was performed as described previously [9], [31] and summarized as follows. After overlapping paired reads to get full-length V6 sequence, the reads were clustered at 95% nt identity using Uclust version 3.0.617 (http://www.drive5.com/usearch/index.html). The most abundant sequence in a cluster was selected as a representative OTU. Taxonomic assignments for the representative OTUs were made through alignments against the Greengenes [32] database using NAST [33] and further manual curation by BLAST to the NCBI non-redundant database. The representative OTU sequences occurring with at least 1% abundance in any one sample have been deposited in GenBank under accession numbers JF262791–JF262909. OTU taxonomic assignments are presented in Table S1.

### Vaginal epithelial RNA extraction

Each of the 32 subjects provided a single sample for human genomic array. Vaginal cytobrush samples were centrifuged (5000×*g*, 10 min, 4°C) and the supernatant was discarded before RNA extraction by TRIzol following the manufacturer's protocol (Invitrogen, Carlsbad, CA, USA). The pellet was resuspended in 20 µl RNase-free water and stored at −80°C until further analysis. RNA quantity and quality was assessed by a Nanodrop 2000c spectrophotometer (Thermo Fisher Scientific, Waltham, MA, USA) to ensure a 260 nm/280 nm ratio above 1.8 and a 260 nm/230 nm ratio above 1.4 before quality was confirmed using an Agilent 2100 Bioanalyzer (Agilent technologies, Santa Clara, CA, USA). All steps of GeneChip probe preparation and hybridization were performed by the LRGC using standard protocols. All liquid handling steps were performed by a GeneChip Fluidics Station 450 and GeneChips were scanned with the GeneChip Scanner 3000 7G (Affymetrix, Santa Clara, CA) using Command Console v1.1.

Sufficient quality and quantity of RNA was received from 10 of the samples, four from the dryness group (age 57.0±8.9 years: range: 50–70 years, with an average 13.3±11.4 years since last menses: range: 3–21 years). and six from the subjects with a normal Nugent score (age 56.2±9.3 years; range: 48–74 years, with an average time since last menses of 11.3±9.8 years: range: 6–30 years).

### Gene expression analysis

Probe level (.CEL file) data was generated using Affymetrix Command Console v1.1. Probes were summarized to gene level data in Partek Genomics Suite v6.5 (Partek, St. Louis, MO) using the RMA algorithm [34].

Gene expression data has been deposited in the Gene Expression Omnibus Database under accession number GSE26761 (http://www.ncbi.nlm.nih.gov/geo/query/acc.cgi?token=hpatrkqyqcqmulk&acc=GSE26761).

### Statistics

Shannon's diversity index [35] was calculated for each subject at each time point as the abundance values of 119 operational taxonomic units (OTUs). The Shannon's diversity index (*S*) was defined as as 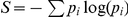 where *p_i_* denotes the proportional abundance values at the *i*th OTU in each sample. The *Lactobacillus* ratio was calculated for each subject at each time point, as the ratio of total abundance value for the *Lactobacillus* OTUs versus all the OTUs.

Hierarchical clustering was performed by calculating the Euclidean distance between microbiota samples using the hclust function in R [36] and the similarity is plotted in a dendogram in Figure 1.

To determine if the linear trends in the *S* trajectories differ significantly across subjects, the following linear mixed effect model [37] was applied to the data 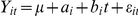. The fixed effect being the intercept only and random effects being subject intercepts and slopes where 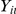 denoted the *S* value of subject *i* at time point *t* and *μ* is an unknown fixed constant, *a_i_* is a random subject intercept, *b_i_* a random subject slope, and *a_i_*, *b_i_*, 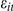 are independent normal random variables.

In order to determine if a statistical relationship existed between microbial diversity and vaginal dryness, a weighted least squares analysis [38] was applied to the data. Observed dryness scores of “none”, “mild”, “moderate”, and “severe” were treated as ordinal values 0, 1, 2, and 3, respectively. The weighted least squares model 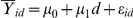 was used to test the linear effect of observed dryness with respect to microbial diversity. Where 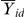 denotes the average *S* of subject *i* at dryness *d*. The quantities 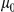 and 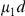 are constants representing intercept and slope respectively. We also applied the Mann-Whitney-Wilcoxen rank-sum to the OTUs by comparing the relative abundances between women with and without dryness at timepoint 0.

## Supporting Information

Figure S1 **A heatmap representing the scored vaginal dryness and atrophy.** The symptoms were self-reported by the participants via a questionnaire, and also reported as observed by the nurse upon examination of the vagina. Atrophy was an overall subjective score by the nurse based on all clinical observations, smoothness was scored as a lack of rugosity, irritation represents symptoms of vaginal irritation and itching. **PDI** - pain during intercourse.
(PDF)

Figure S2 **Relative abundances of each OTU for samples at time point 0 with and without vaginal dryness.** Each plot represents a single OTU and the relative abundance (fraction of microbiota) for each time point 0 sample is plotted on the y-axis. The samples are divided into two groups based on the nurse's observation: women without vaginal dryness (none or mild) and women with vaginal dryness (moderate or severe). The p-value represents the result of the Mann-Whitney-Wilcoxen rank-sum test on the relative OTU abundances between the dry and non-dry groups.
(PDF)

Table S1 **Taxonomic assignments to OTUs at ≥1% relative abundance in any one sample.** Assignments were made using the Greengenes database [32] and manually validated by BLAST [39] to the NCBI non-redundant database.
(PDF)

Table S2 **Affymetrix read outs for 960 probe sets representing those differentially expressed by at least 2-fold.**
(PDF)
