# Supplementary figures and images for: Vaginal Microbiome and Epithelial Gene Array in Post-Menopausal Women with Moderate to Severe Dryness
Source: PLoS One. 2011 Nov 2;6(11):e26602. doi: 10.1371/journal.pone.0026602 (PMC3206802; doi:10.1371/journal.pone.0026602)

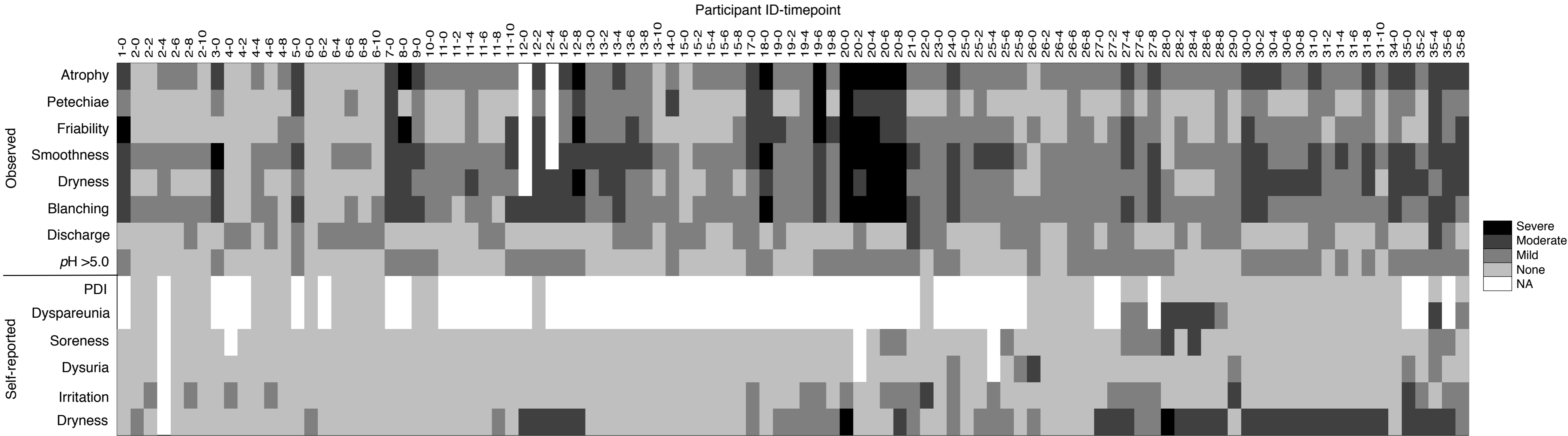

Supplement: Figure S1 — A heatmap representing the scored vaginal dryness and atrophy. The symptoms were self-reported by the participants via a questionnaire, and also reported as observed by the nurse upon examination of the vagina. Atrophy was an overall subjective score by the nurse based on all clinical observations, smoothness was scored as a lack of rugosity, irritation represents symptoms of vaginal irritation and itching. PDI - pain during intercourse. (PDF) [file pone.0026602.s001.pdf]
