# Supplementary material for: Vaginal Microbiome and Epithelial Gene Array in Post-Menopausal Women with Moderate to Severe Dryness
Source: PLoS One. 2011 Nov 2;6(11):e26602. doi: 10.1371/journal.pone.0026602 (PMC3206802; doi:10.1371/journal.pone.0026602)
Supplement: Figure S2 — Relative abundances of each OTU for samples at time point 0 with and without vaginal dryness. Each plot represents a single OTU and the relative abundance (fraction of microbiota) for each time point 0 sample is plotted on the y-axis. The samples are divided into two groups based on the nurse's observation: women without vaginal dryness (none or mild) and women with vaginal dryness (moderate or severe). The p-value represents the result of the Mann-Whitney-Wilcoxen rank-sum test on the relative OTU abundances between the dry and non-dry groups. (PDF) [file pone.0026602.s002.pdf]

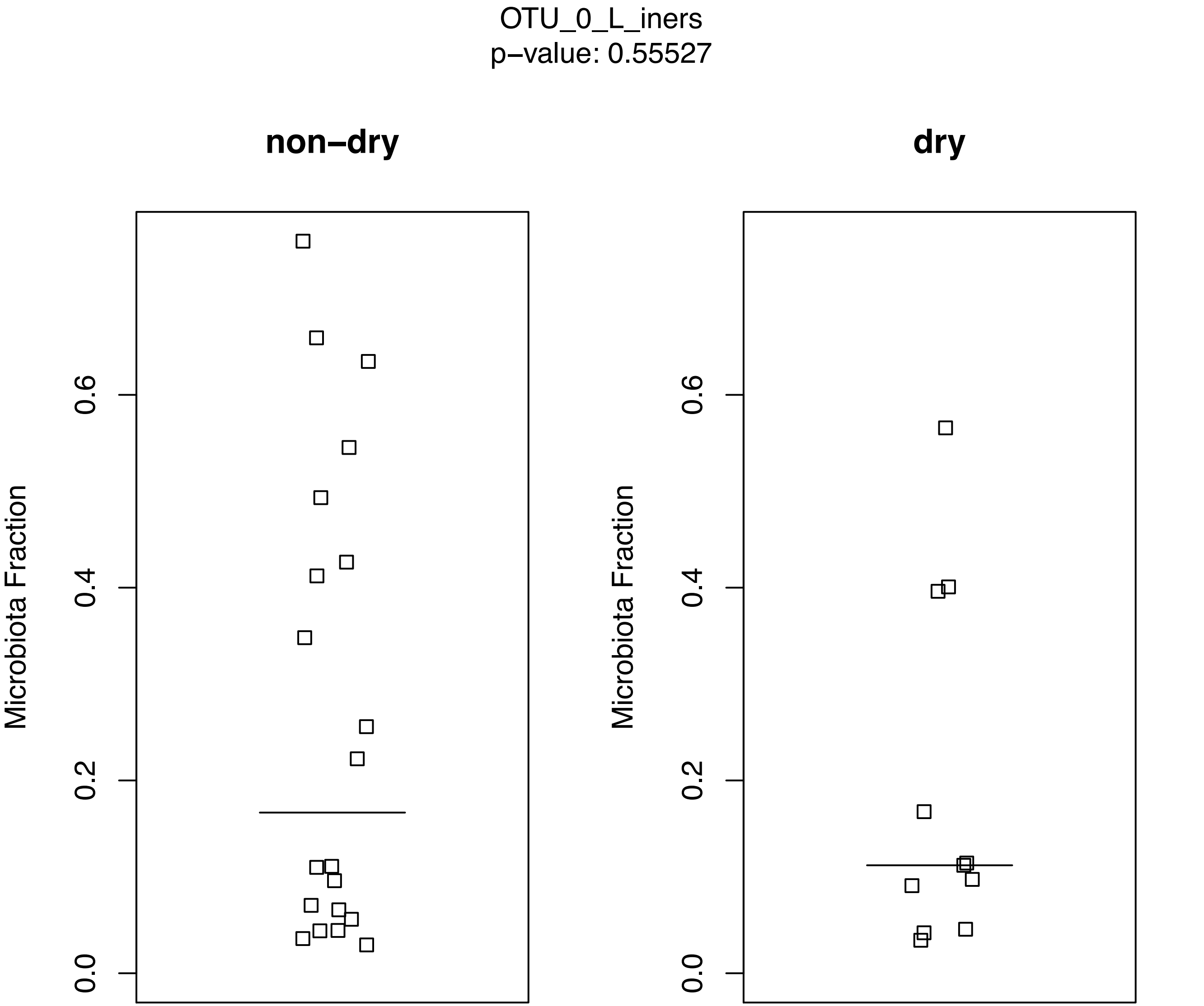

OTU_0_L_iners
p–value: 0.55527
non–dry
dry
Microbiota Fraction
0.0
0.2
0.4
0.6
Microbiota Fraction
0.0
0.2
0.4
0.6

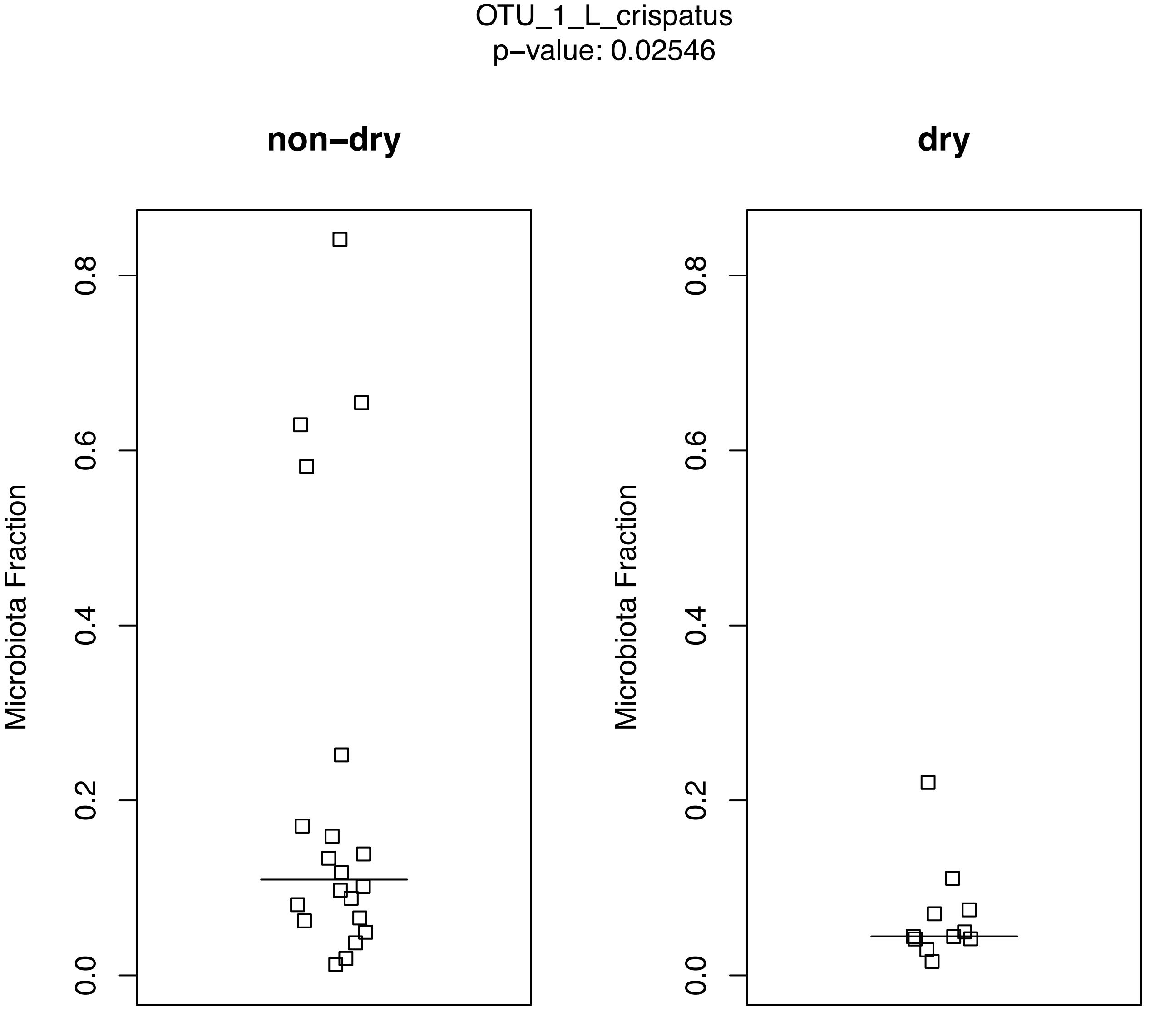
OTU_1_L_crispatus
p−value: 0.02546
non−dry
dry
Microbiota Fraction
0.0
0.2
0.4
0.6
0.8
Microbiota Fraction
0.0
0.2
0.4
0.6
0.8

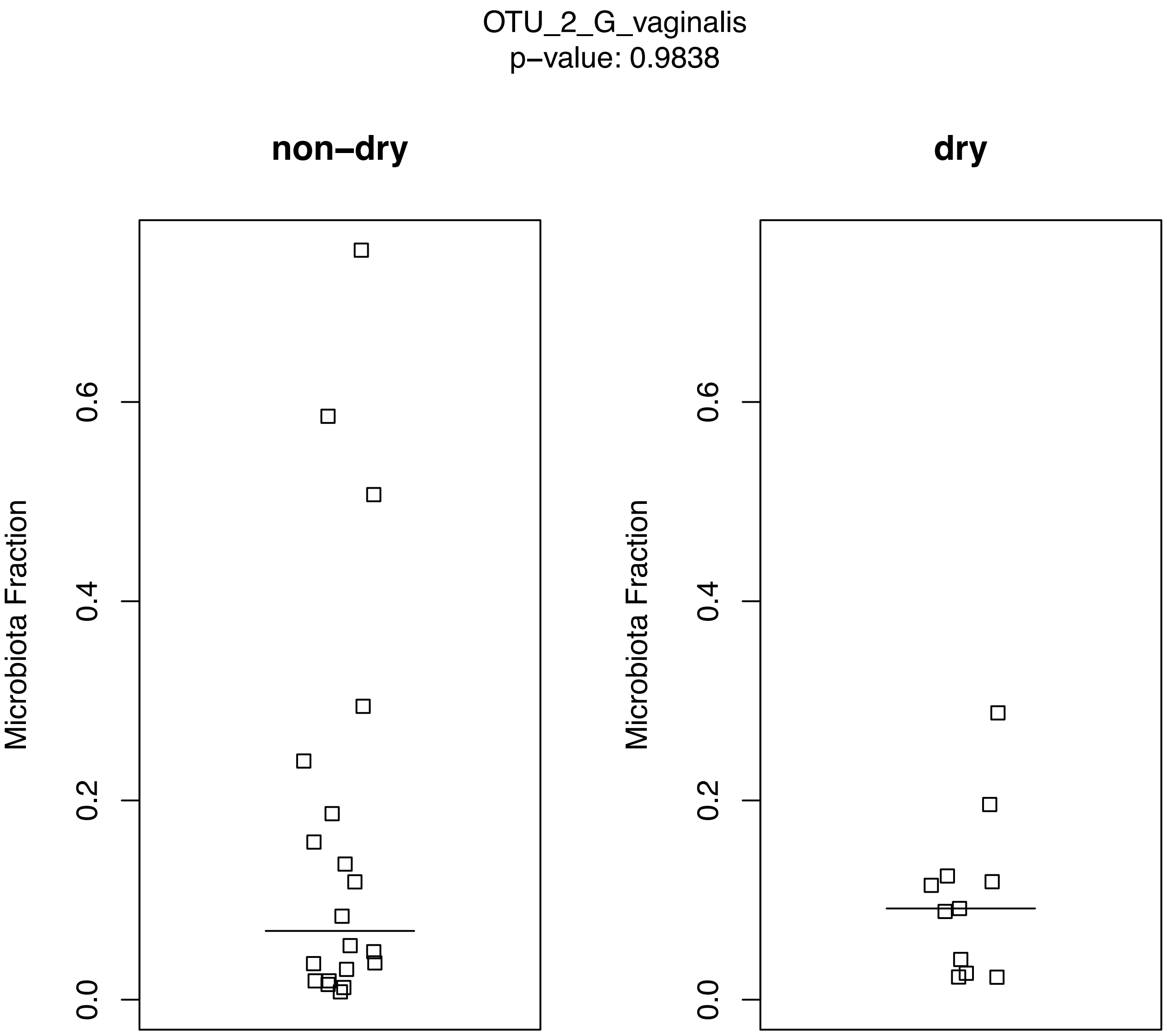
OTU_2_G_vaginalis
p–value: 0.9838
non–dry
dry
Microbiota Fraction
Microbiota Fraction
0.0
0.2
0.4
0.6
0.0
0.2
0.4
0.6

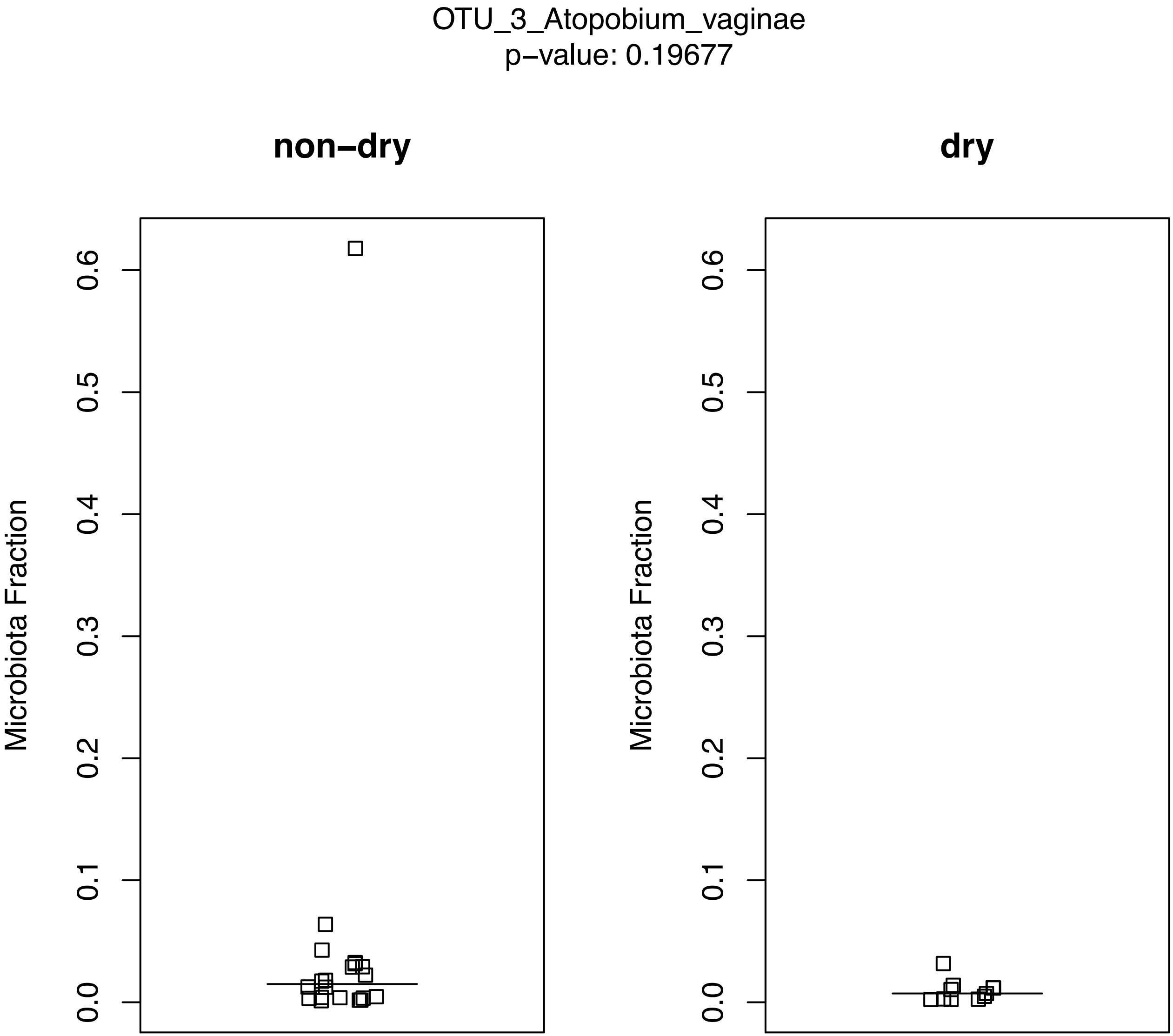

OTU_3_Atopobium_vaginae
p−value: 0.19677
non−dry
dry
Microbiota Fraction
Microbiota Fraction
0.0
0.1
0.2
0.3
0.4
0.5
0.6
0.0
0.1
0.2
0.3
0.4
0.5
0.6

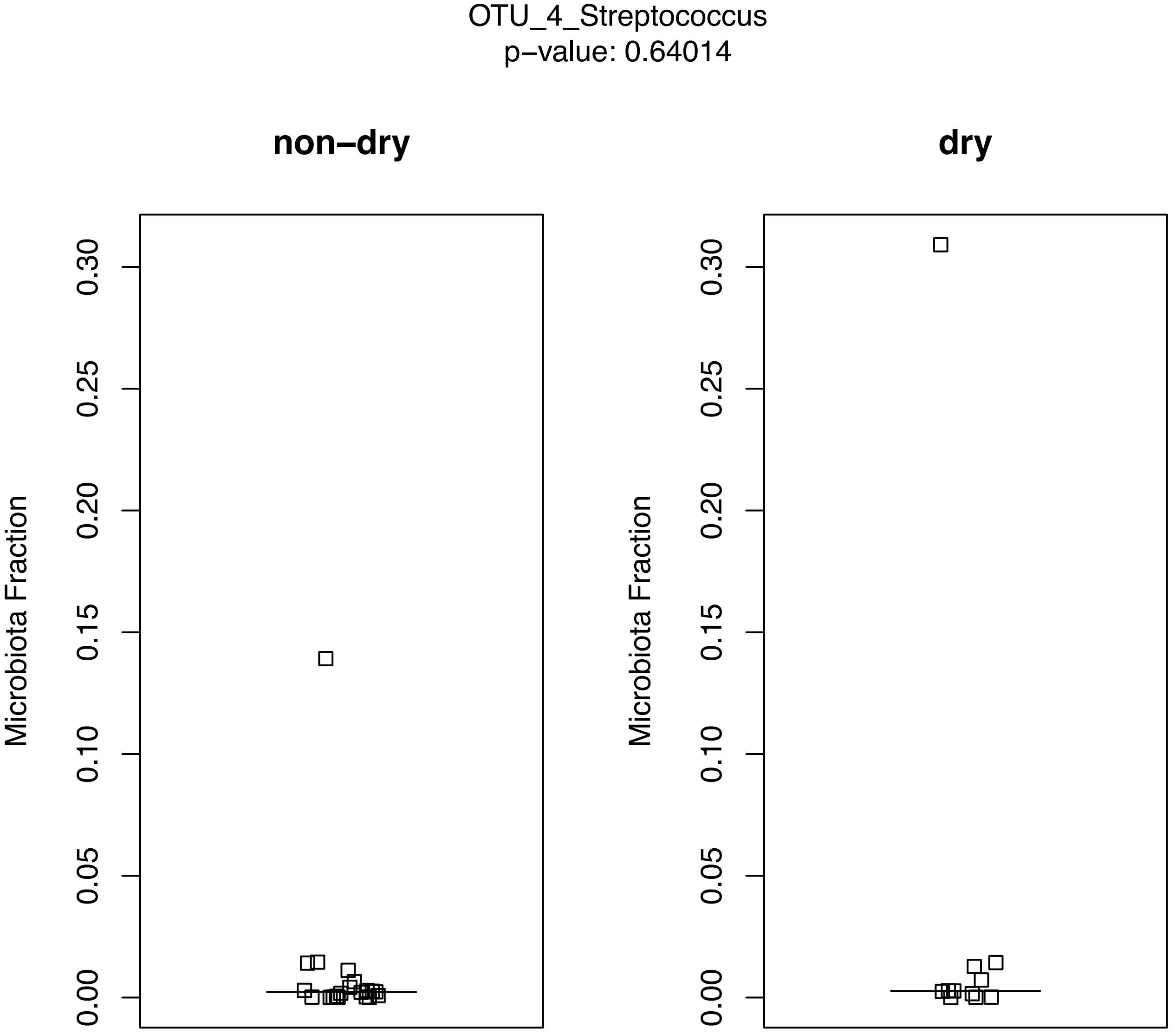

OTU_4_Streptococcus
p−value: 0.64014
non−dry
dry
Microbiota Fraction
Microbiota Fraction
0.00
0.05
0.10
0.15
0.20
0.25
0.30
0.00
0.05
0.10
0.15
0.20
0.25
0.30

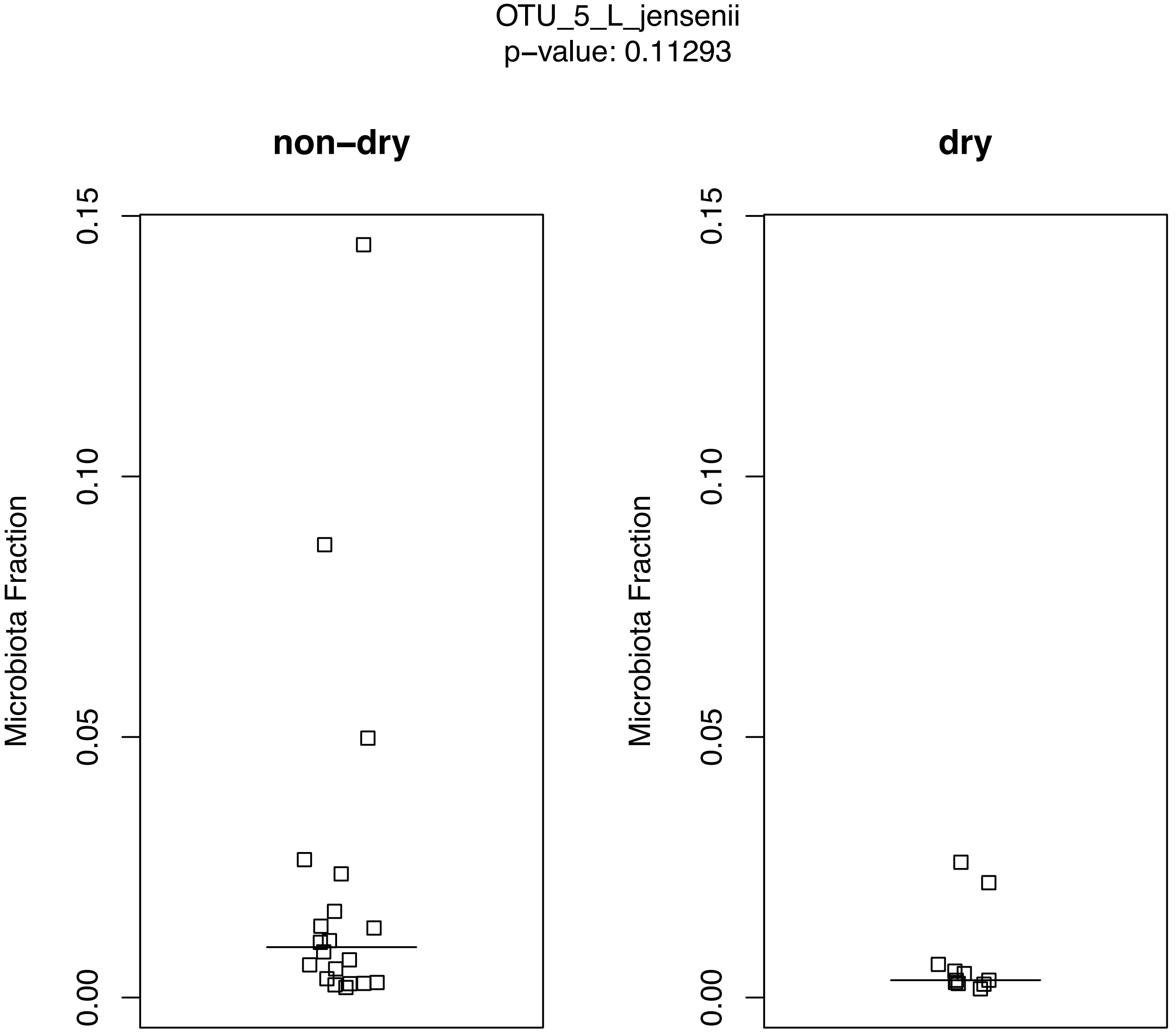
OTU_5_L_jensenii
p−value: 0.11293
non−dry
dry
Microbiota Fraction
Microbiota Fraction
0.00
0.05
0.10
0.15
0.00
0.05
0.10
0.15

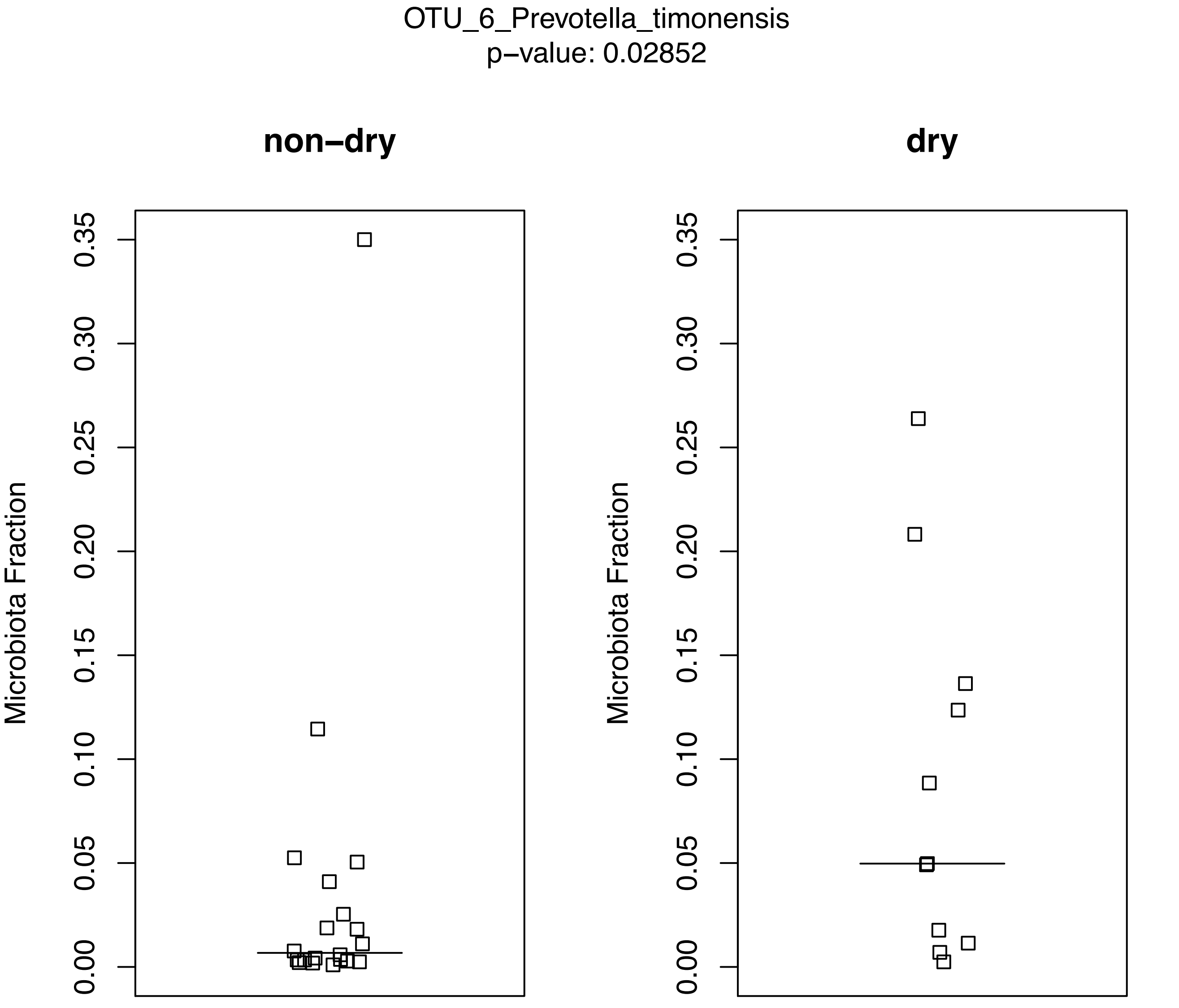

OTU_6_Prevotella_timonensis
p−value: 0.02852
non−dry
dry
Microbiota Fraction
Microbiota Fraction
0.00
0.05
0.10
0.15
0.20
0.25
0.30
0.35
0.00
0.05
0.10
0.15
0.20
0.25
0.30
0.35

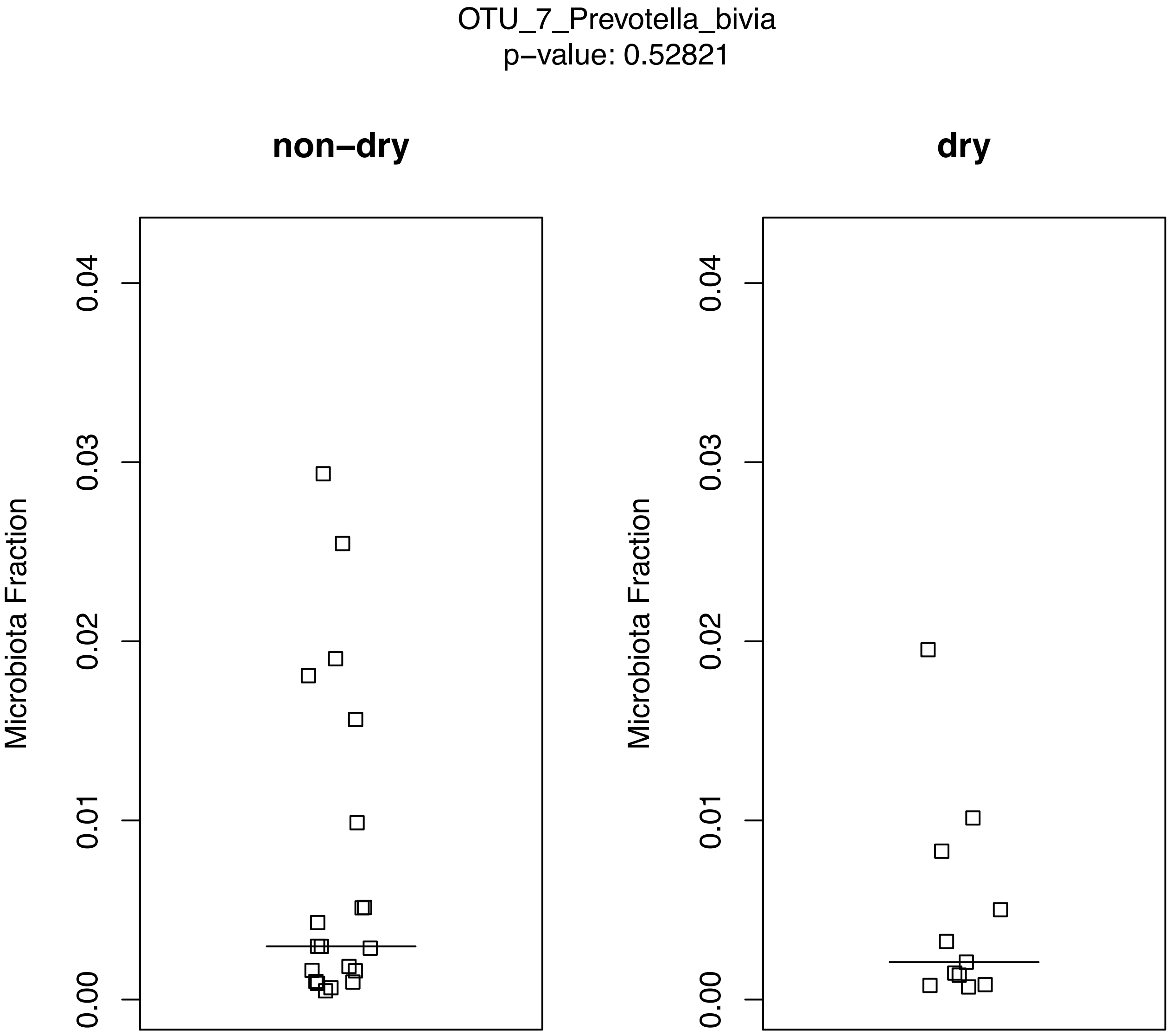
OTU_7_Prevotella_bivia
p−value: 0.52821
non−dry
dry
Microbiota Fraction
0.00
0.01
0.02
0.03
0.04

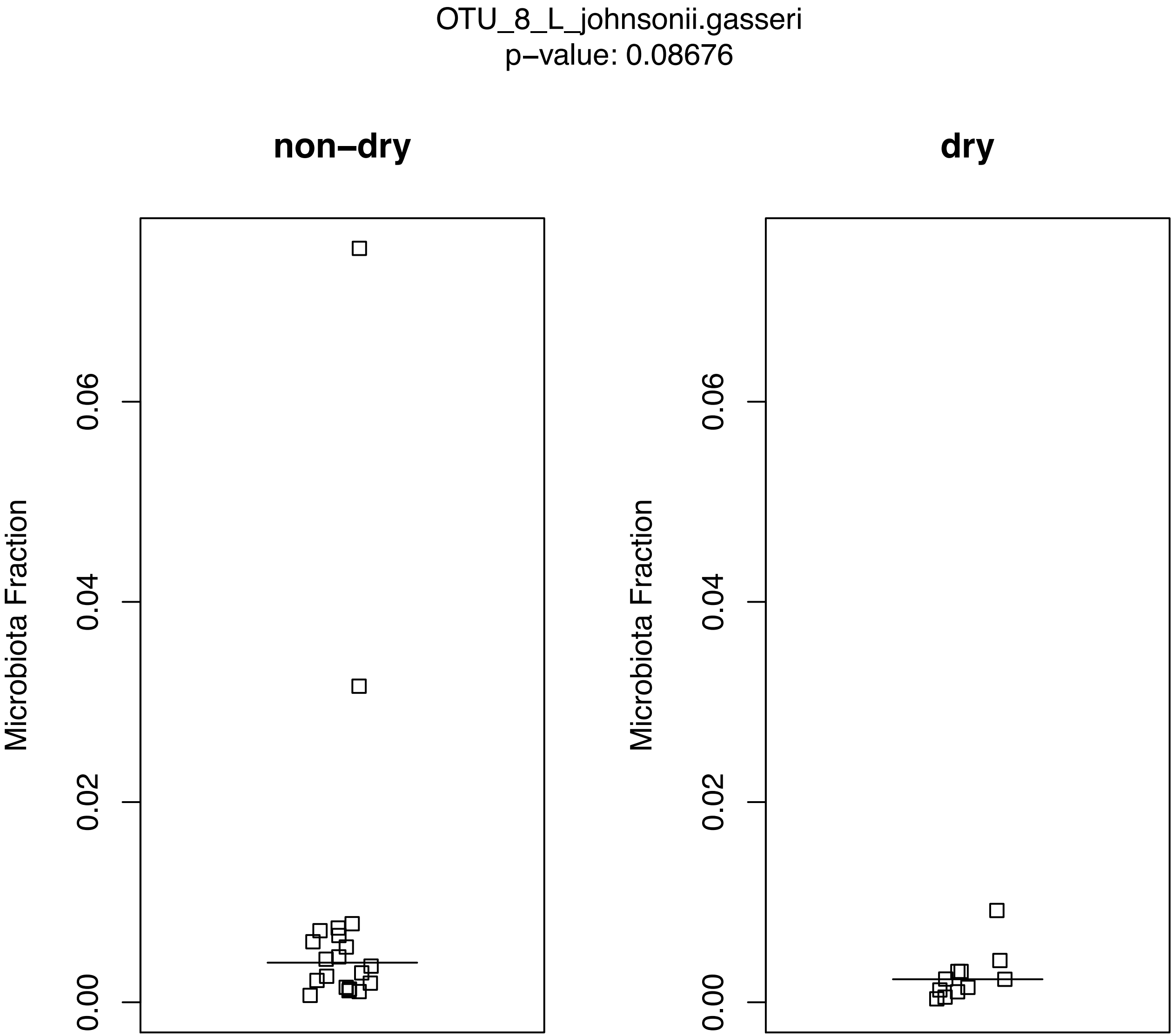
OTU_8_L_johnsonii.gasseri
p−value: 0.08676
non−dry
dry
Microbiota Fraction
0.00
0.02
0.04
0.06
Microbiota Fraction
0.00
0.02
0.04
0.06

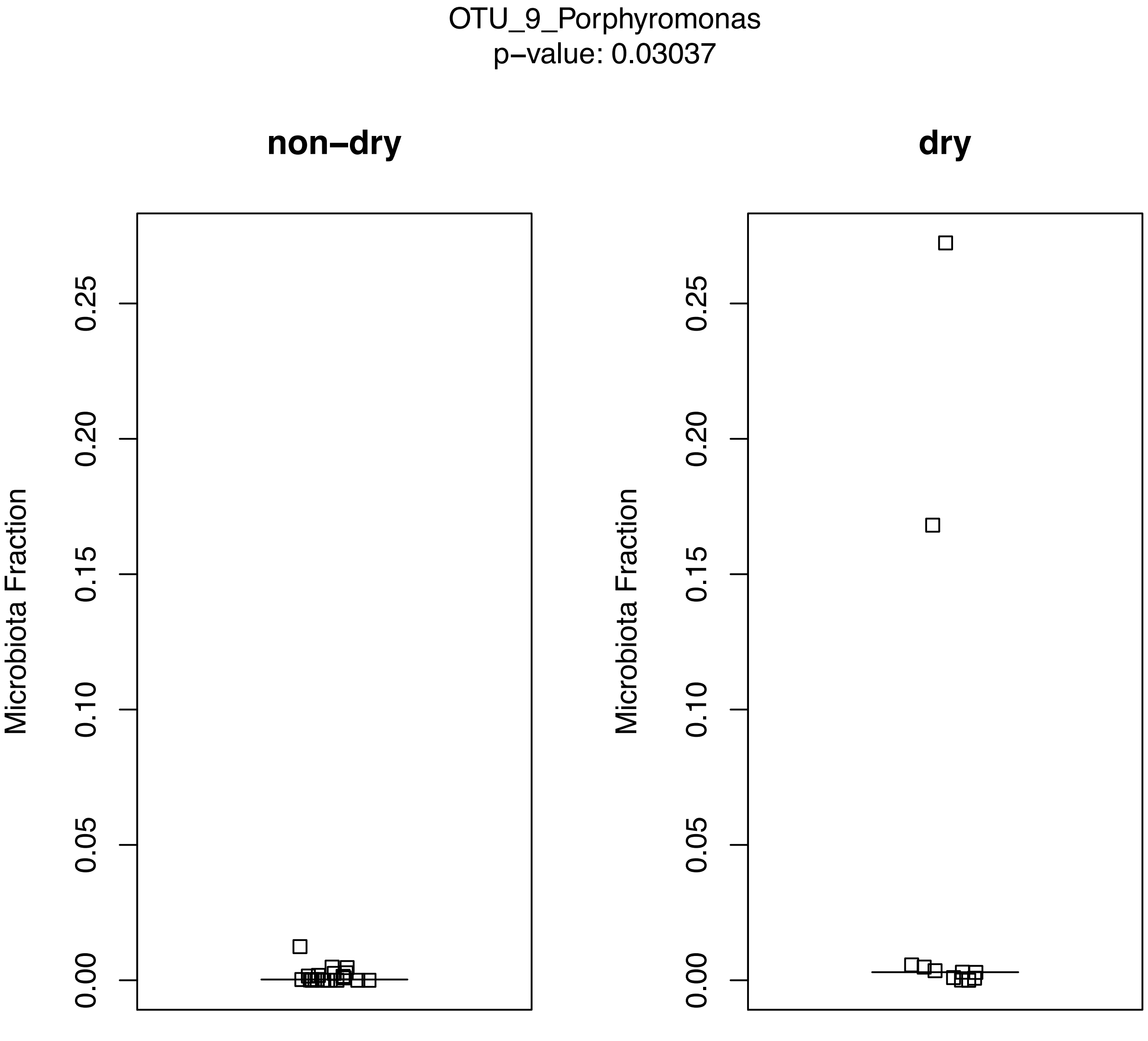
OTU_9_Porphyromonas
p−value: 0.03037
non−dry
dry
Microbiota Fraction
Microbiota Fraction
0.00
0.05
0.10
0.15
0.20
0.25

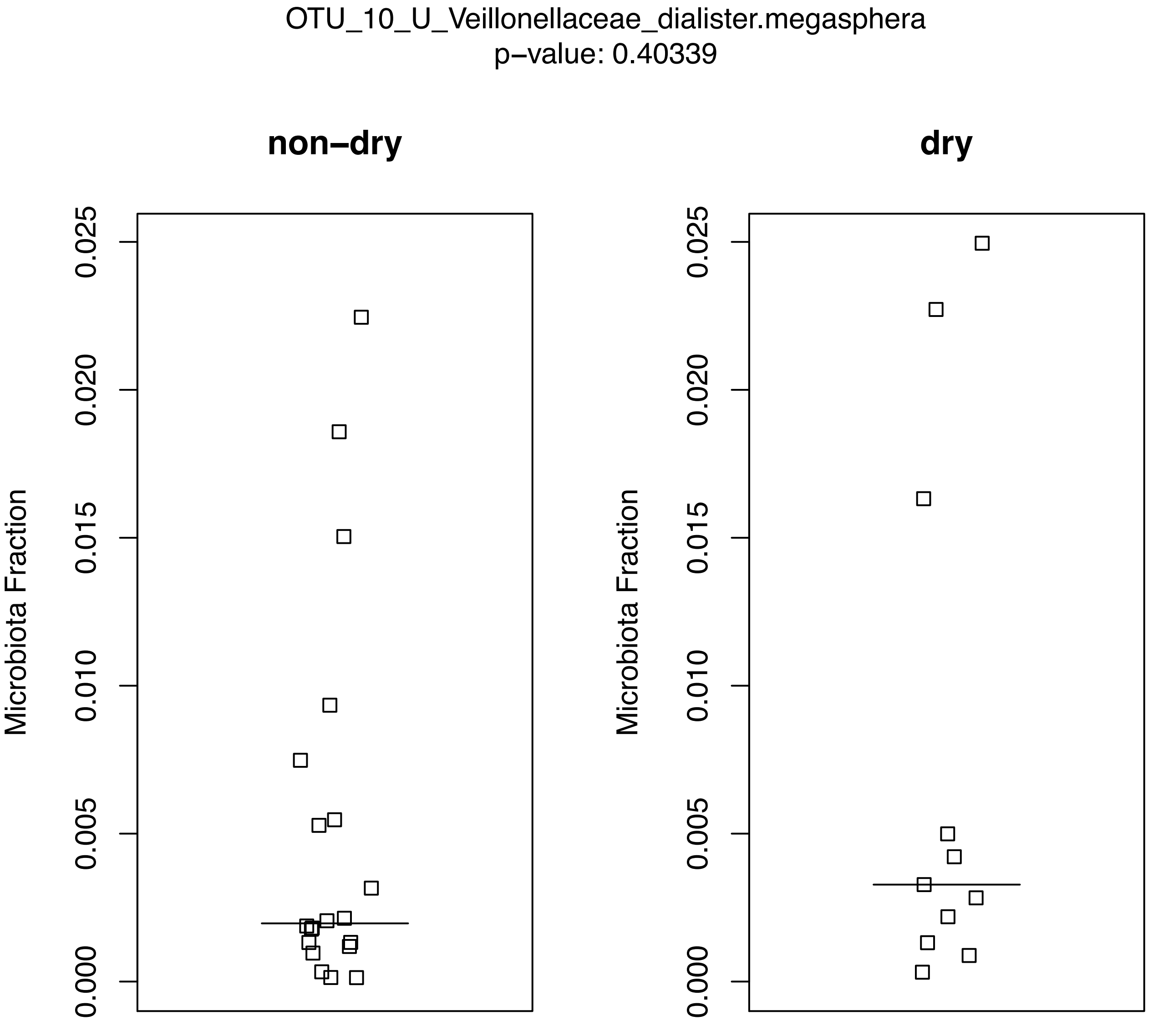
OTU_10_U_Veillonellaceae_dialister.megasphera
p−value: 0.40339
non−dry
dry
Microbiota Fraction
0.000
0.005
0.010
0.015
0.020
0.025

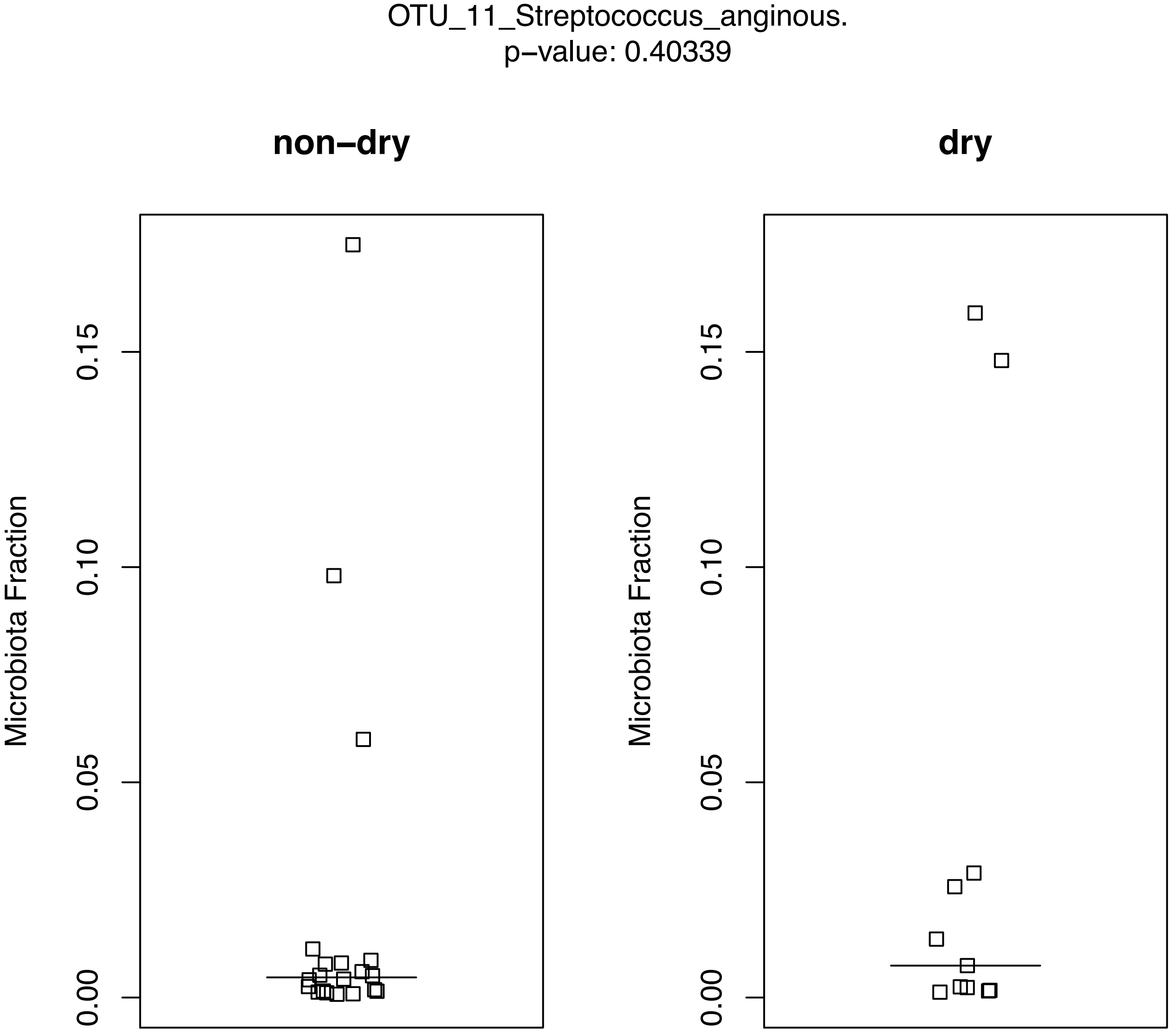
OTU_11_Streptococcus_anginous.
p−value: 0.40339
non−dry
dry
Microbiota Fraction
0.00
0.05
0.10
0.15
Microbiota Fraction
0.00
0.05
0.10
0.15

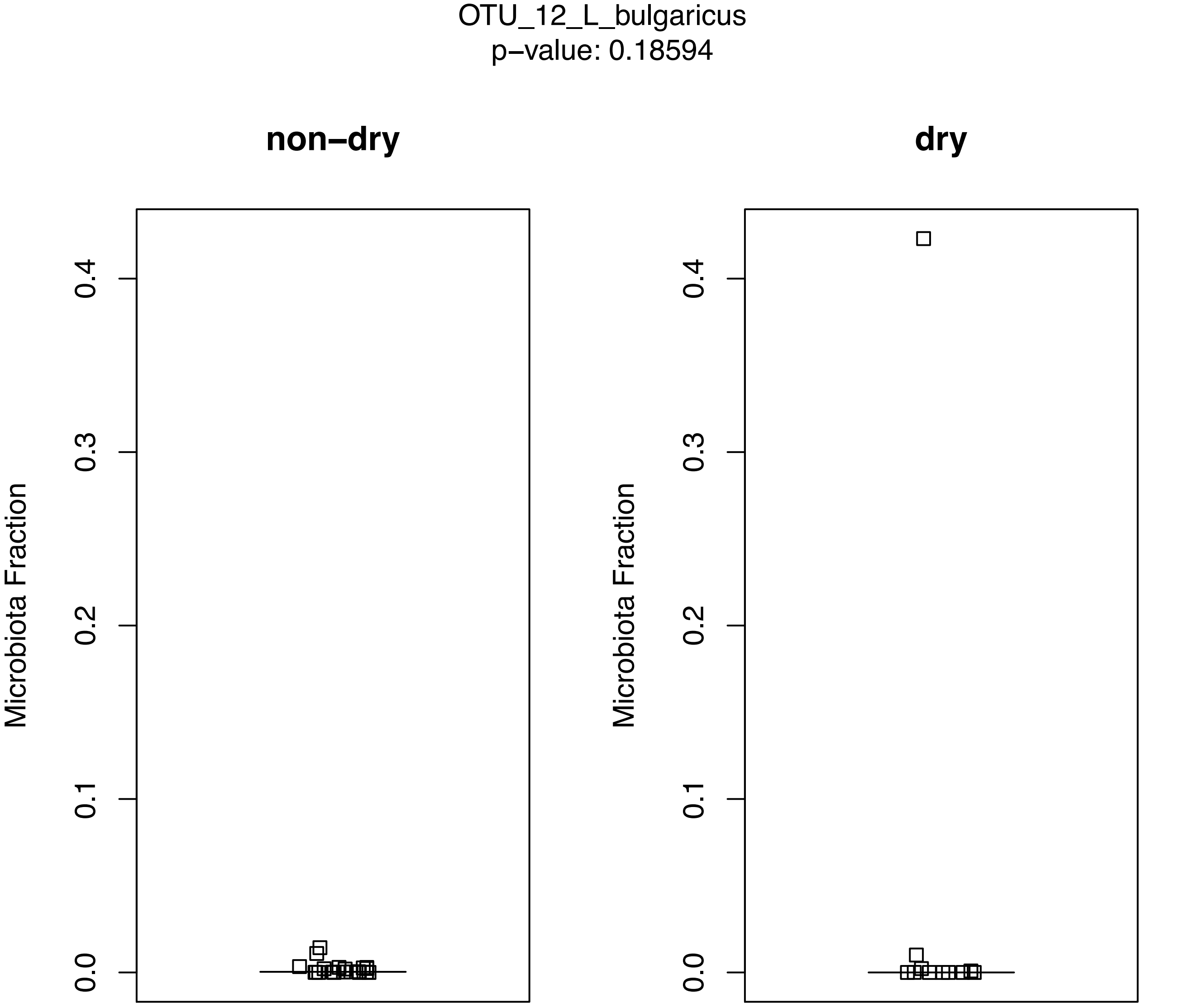

OTU_12_L_bulgaricus
p−value: 0.18594
non−dry
dry
Microbiota Fraction
Microbiota Fraction
0.0
0.1
0.2
0.3
0.4
0.0
0.1
0.2
0.3
0.4

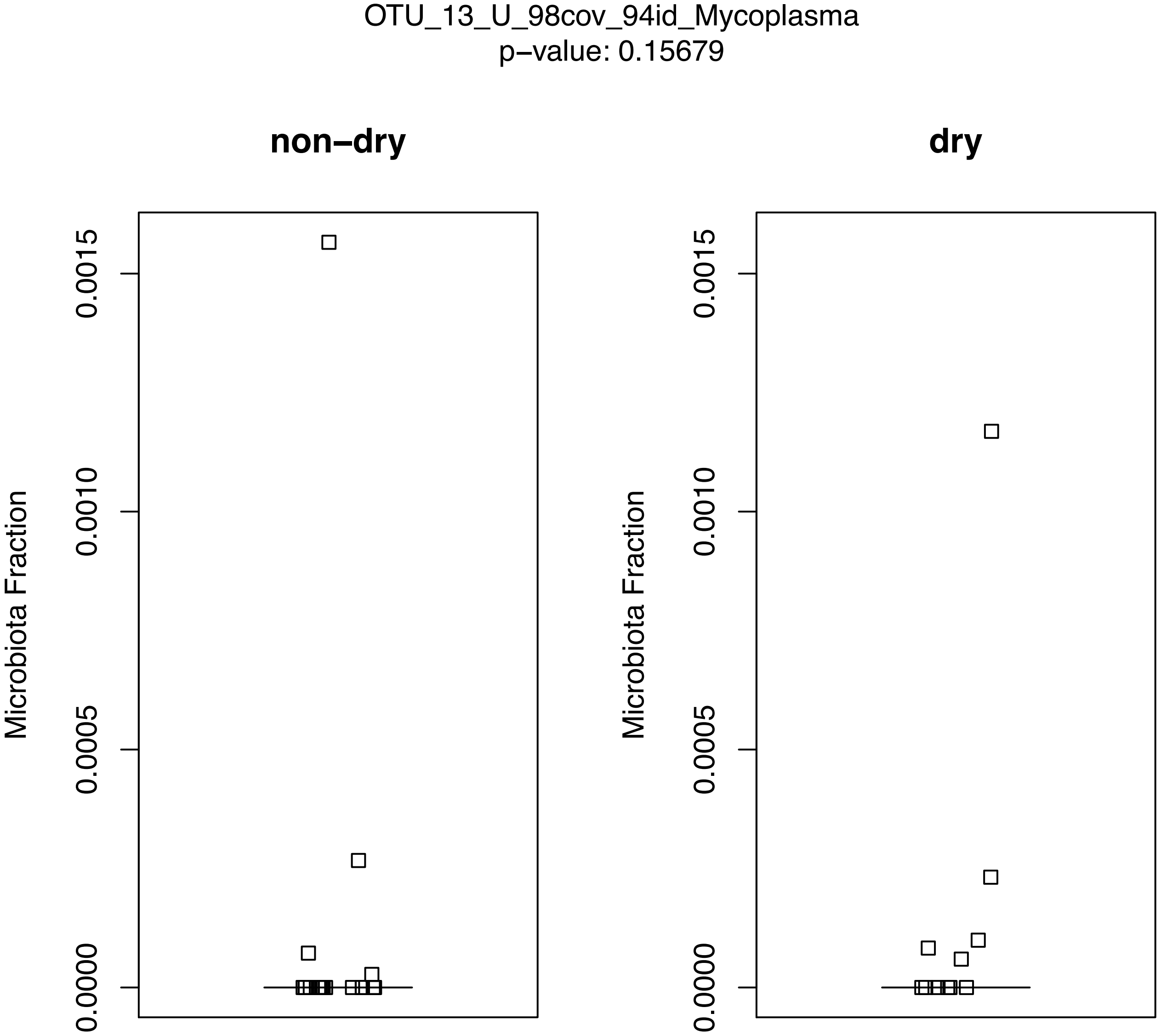

OTU_13_U_98cov_94id_Mycoplasma
p−value: 0.15679
non−dry
dry
Microbiota Fraction
0.0000
0.0005
0.0010
0.0015

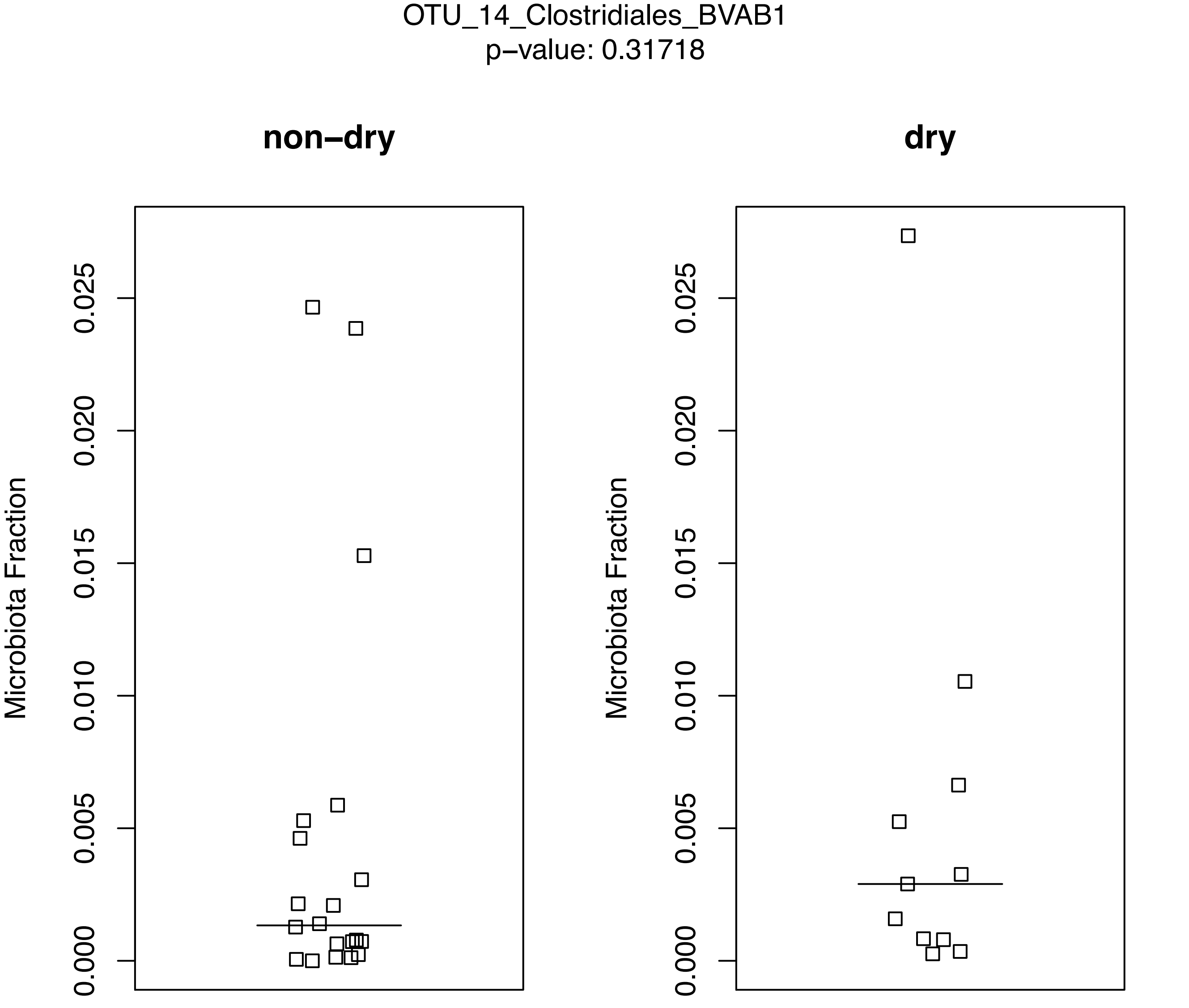

OTU_14_Clostridiales_BVAB1
p–value: 0.31718
non–dry
dry
Microbiota Fraction
0.000
0.005
0.010
0.015
0.020
0.025
Microbiota Fraction
0.000
0.005
0.010
0.015
0.020
0.025

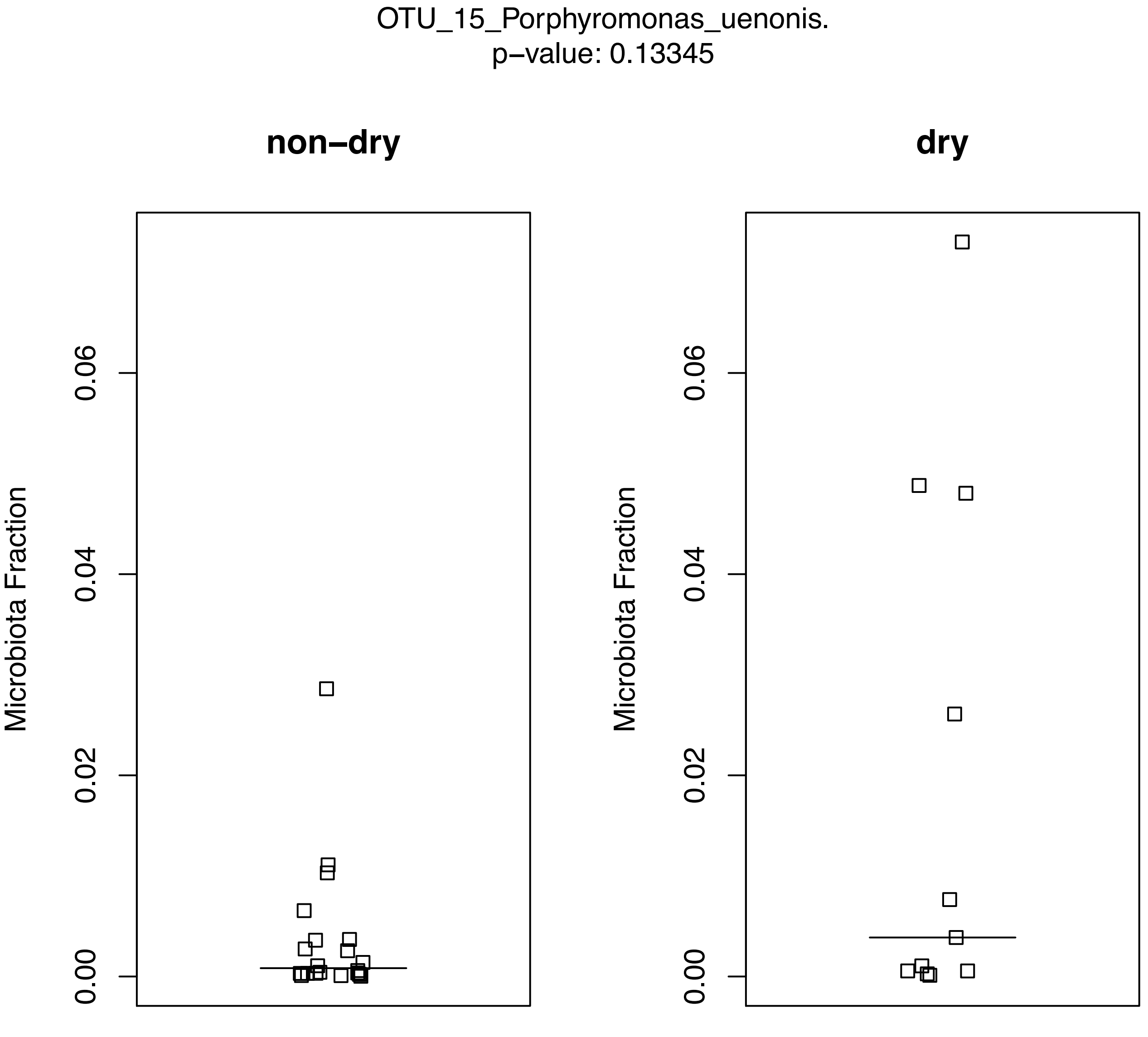
OTU_15_Porphyromonas_uenonis.
p−value: 0.13345
non−dry
dry
Microbiota Fraction
0.00
0.02
0.04
0.06
Microbiota Fraction
0.00
0.02
0.04
0.06

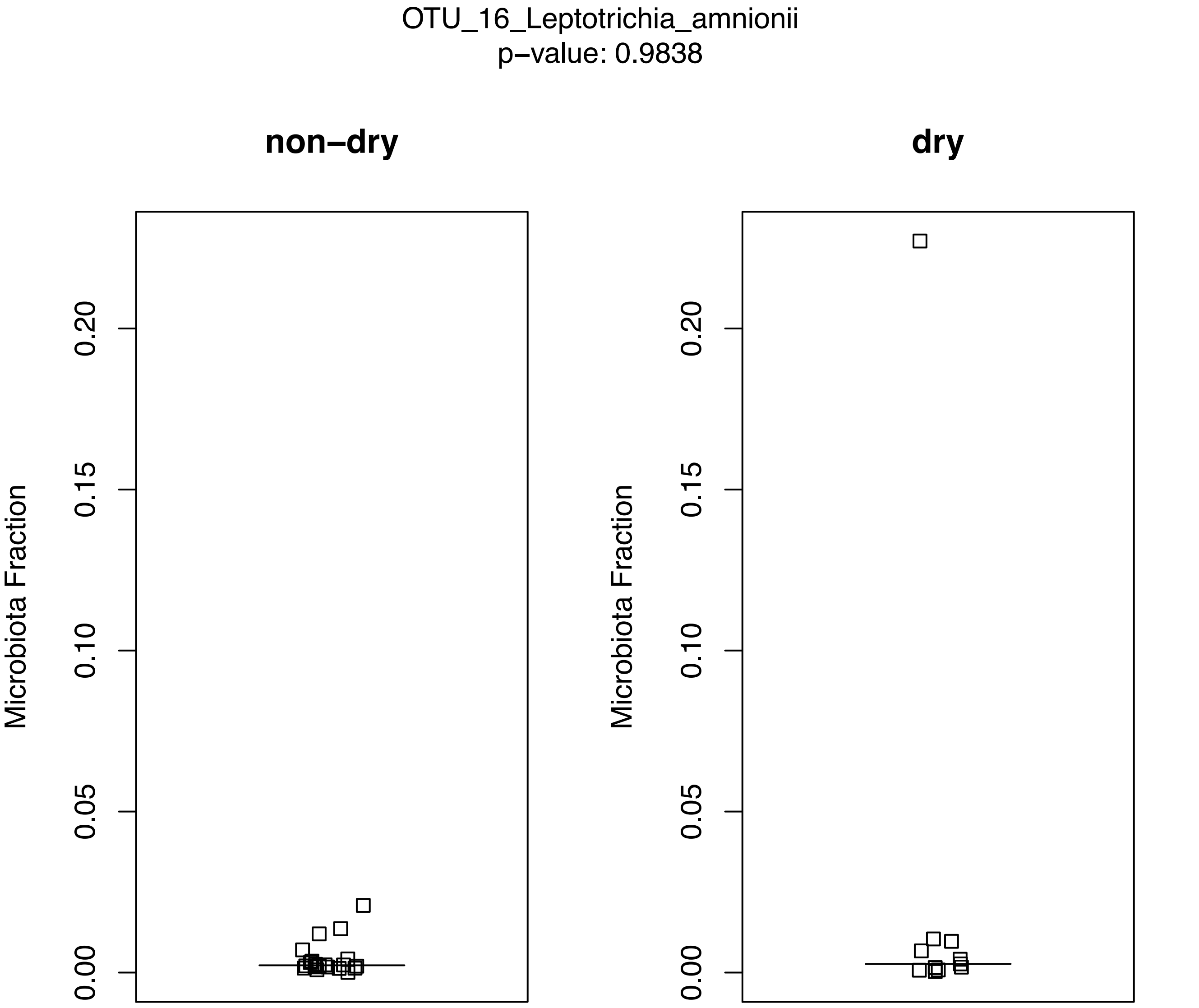
OTU_16_Leptotrichia_amnionii
p−value: 0.9838
non−dry
dry
Microbiota Fraction
0.00
0.05
0.10
0.15
0.20
Microbiota Fraction
0.00
0.05
0.10
0.15
0.20

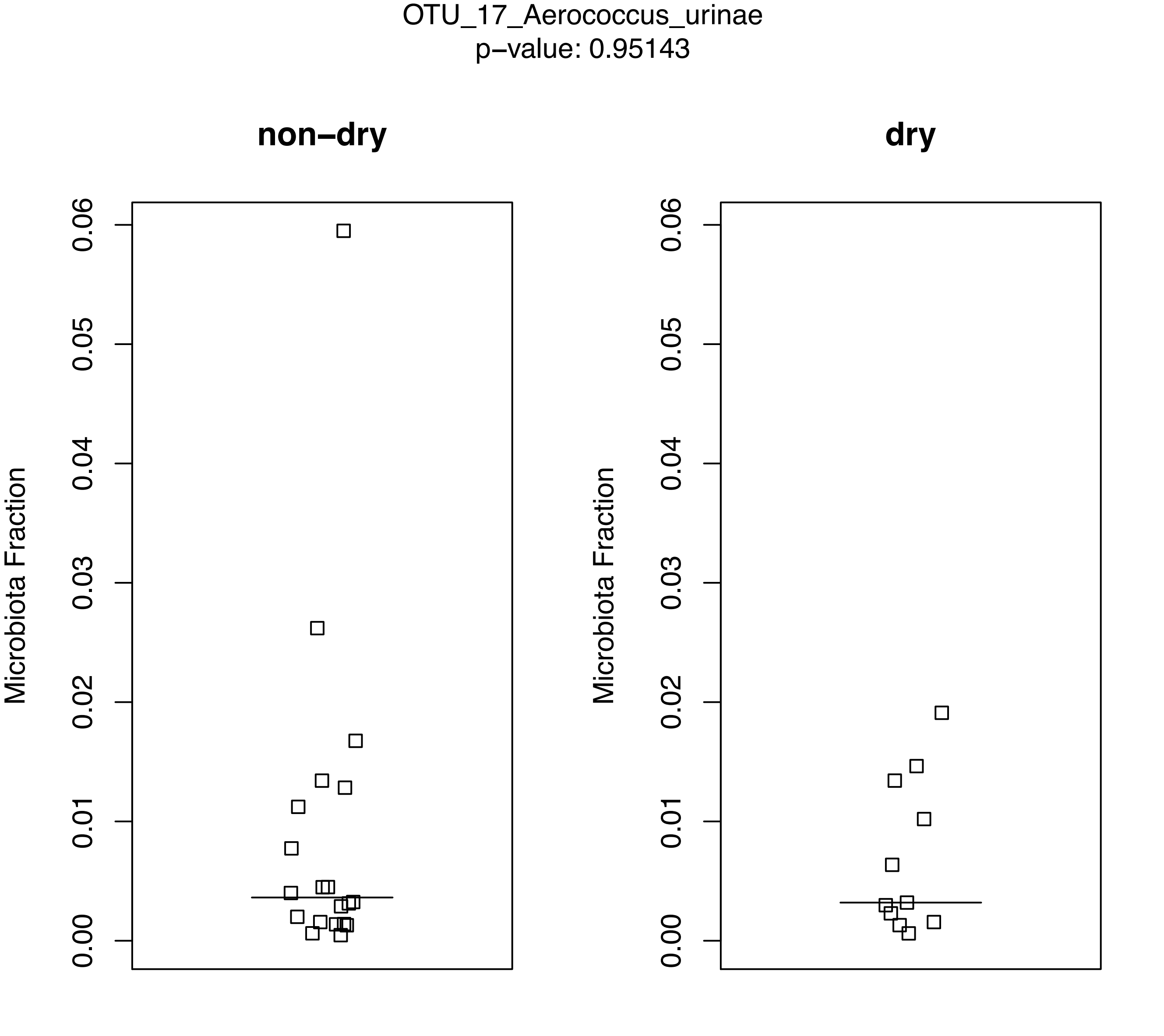
OTU_17_Aerococcus_urinae
p−value: 0.95143
non−dry
dry
Microbiota Fraction
0.00
0.01
0.02
0.03
0.04
0.05
0.06
Microbiota Fraction
0.00
0.01
0.02
0.03
0.04
0.05
0.06

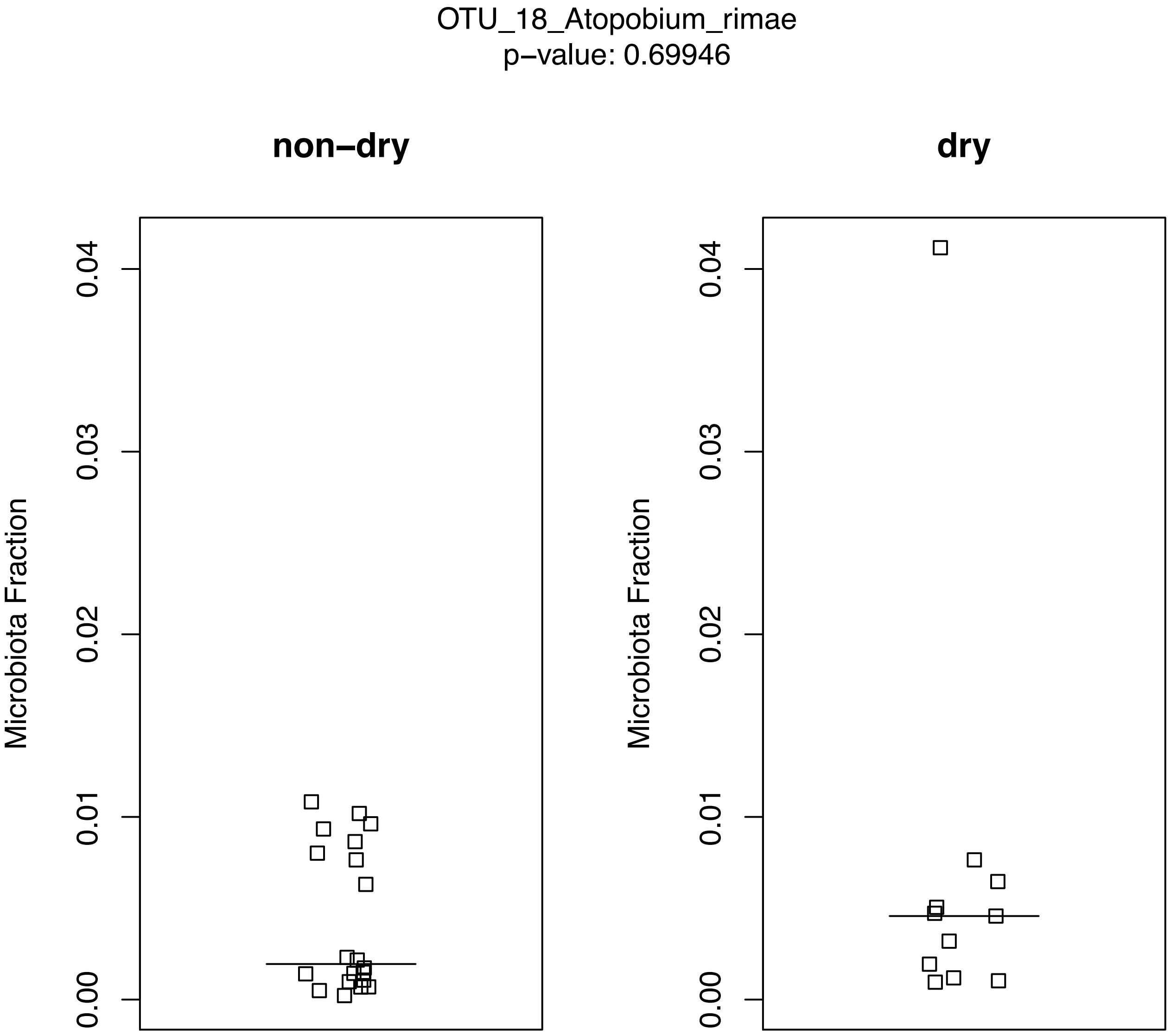
OTU_18_Atopobium_rimae
p−value: 0.69946
non−dry
dry
Microbiota Fraction
0.00
0.01
0.02
0.03
0.04
Microbiota Fraction
0.00
0.01
0.02
0.03
0.04

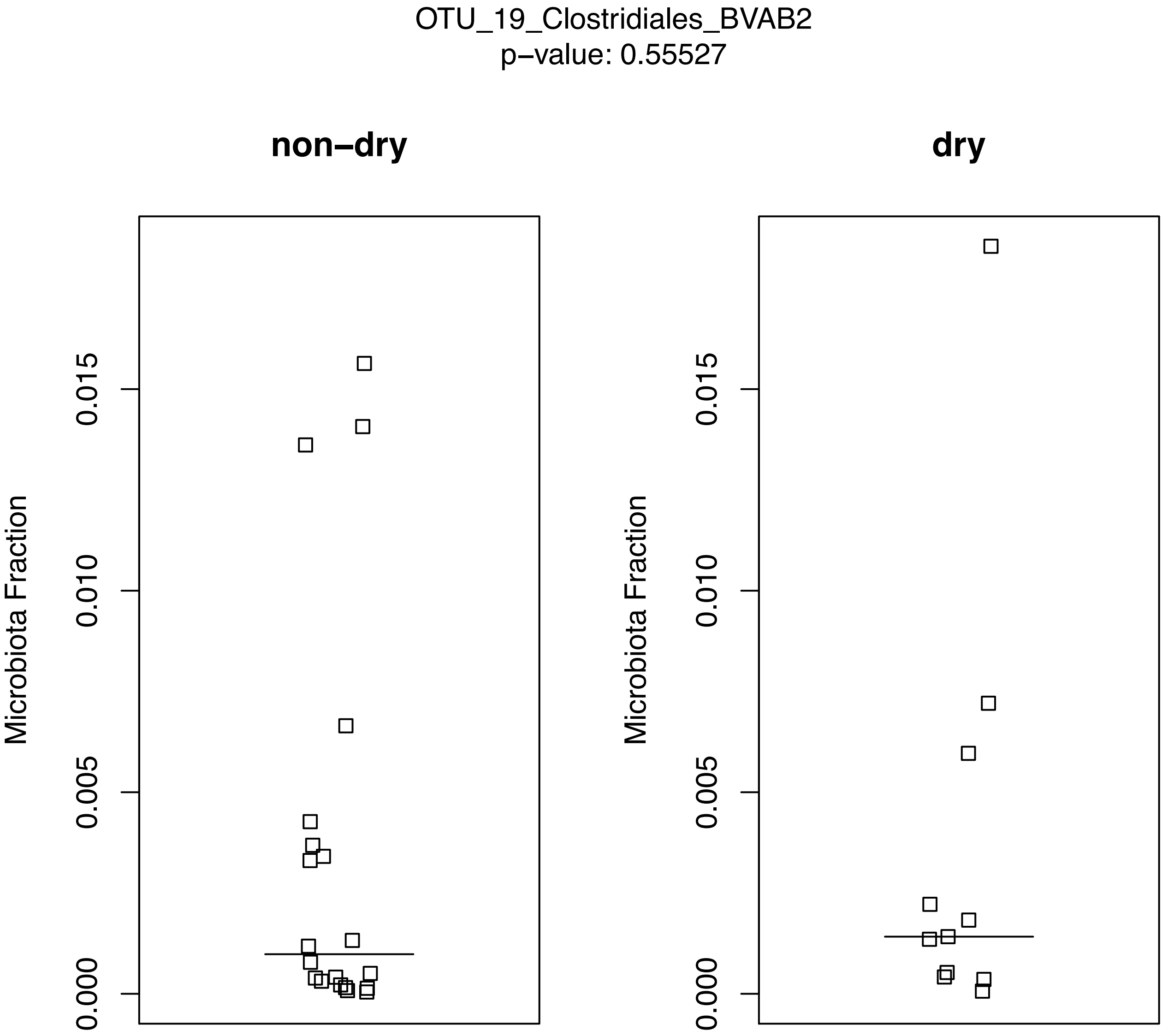

OTU_19_Clostridiales_BVAB2
p−value: 0.55527
non−dry
dry
Microbiota Fraction
0.000
0.005
0.010
0.015

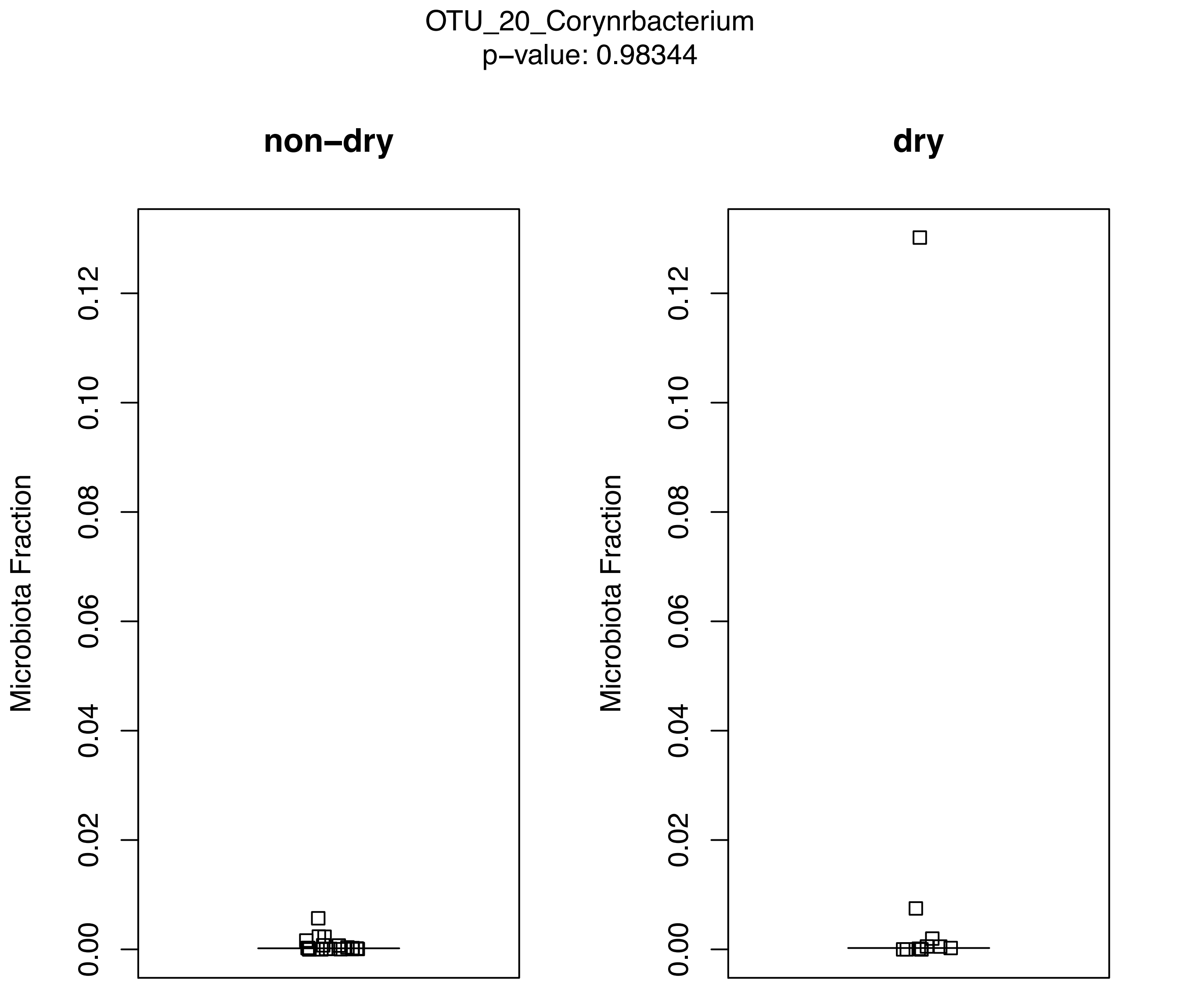
OTU_20_Corynrbacterium
p−value: 0.98344
non−dry
dry
Microbiota Fraction
0.00
0.02
0.04
0.06
0.08
0.10
0.12
Microbiota Fraction
0.00
0.02
0.04
0.06
0.08
0.10
0.12

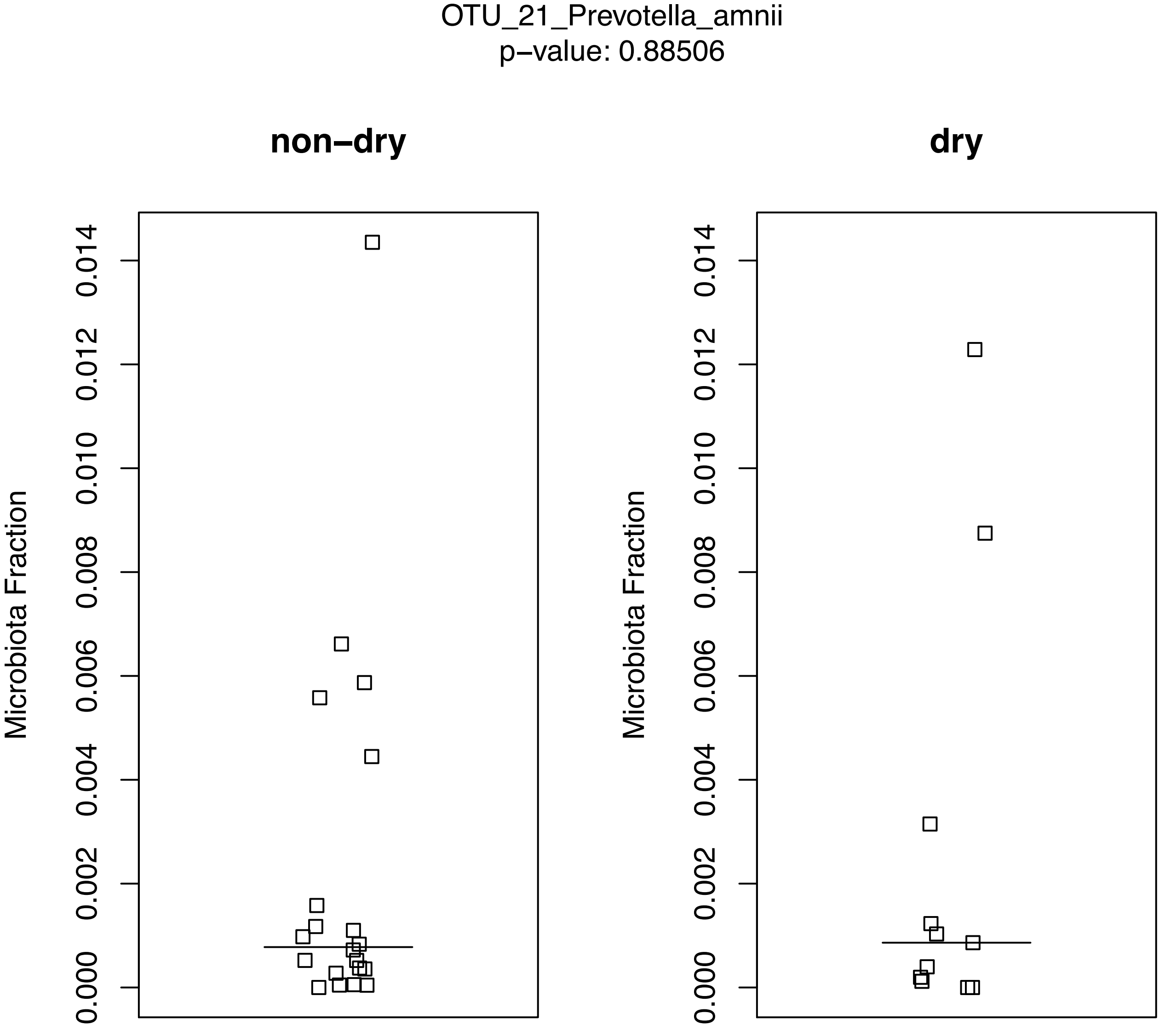
OTU_21_Prevotella_amnii
p−value: 0.88506
non−dry
dry
Microbiota Fraction
0.000
0.002
0.004
0.006
0.008
0.010
0.012
0.014

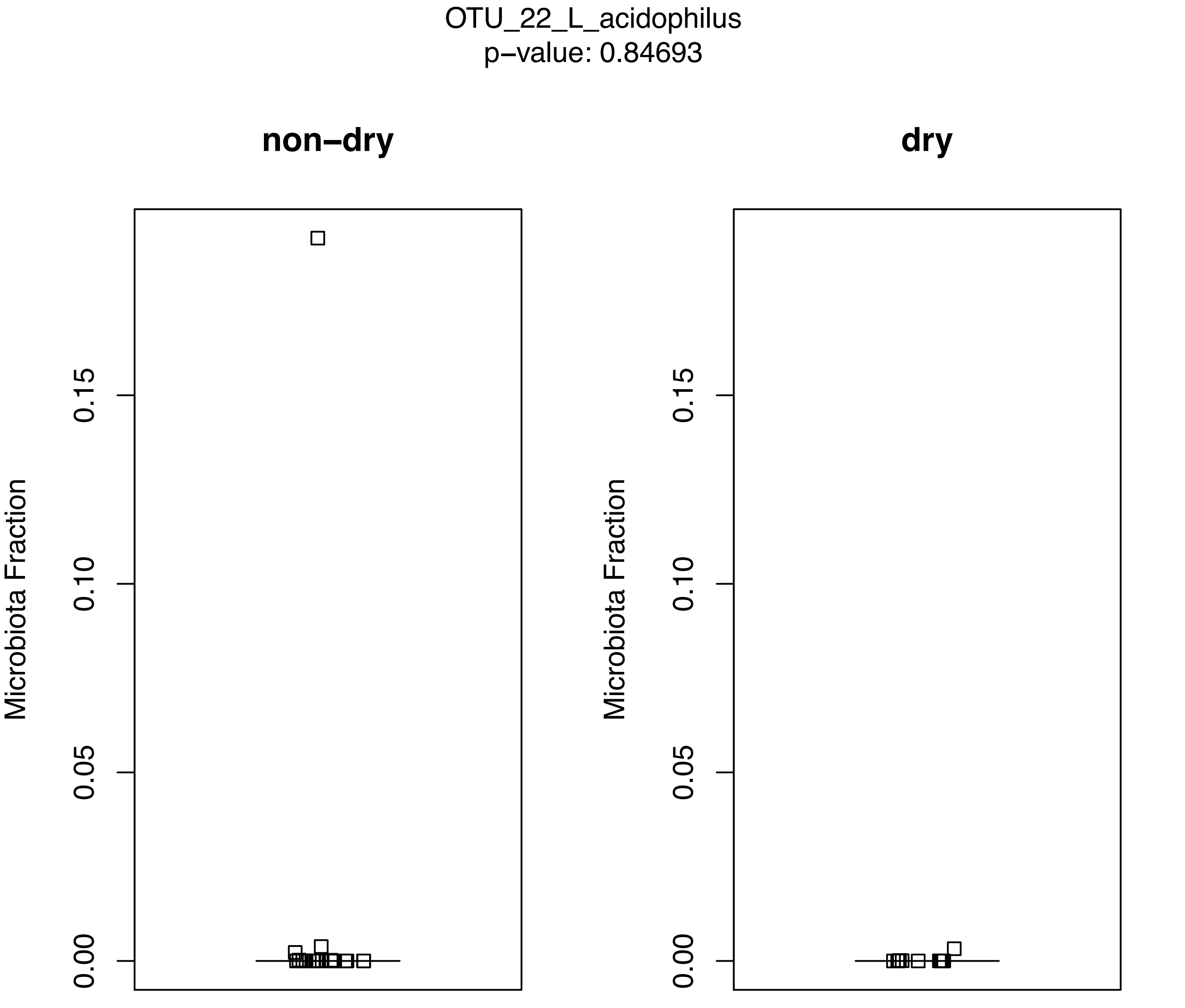

OTU_22_L_acidophilus
p−value: 0.84693
non−dry
dry
Microbiota Fraction
0.00
0.05
0.10
0.15
Microbiota Fraction
0.00
0.05
0.10
0.15

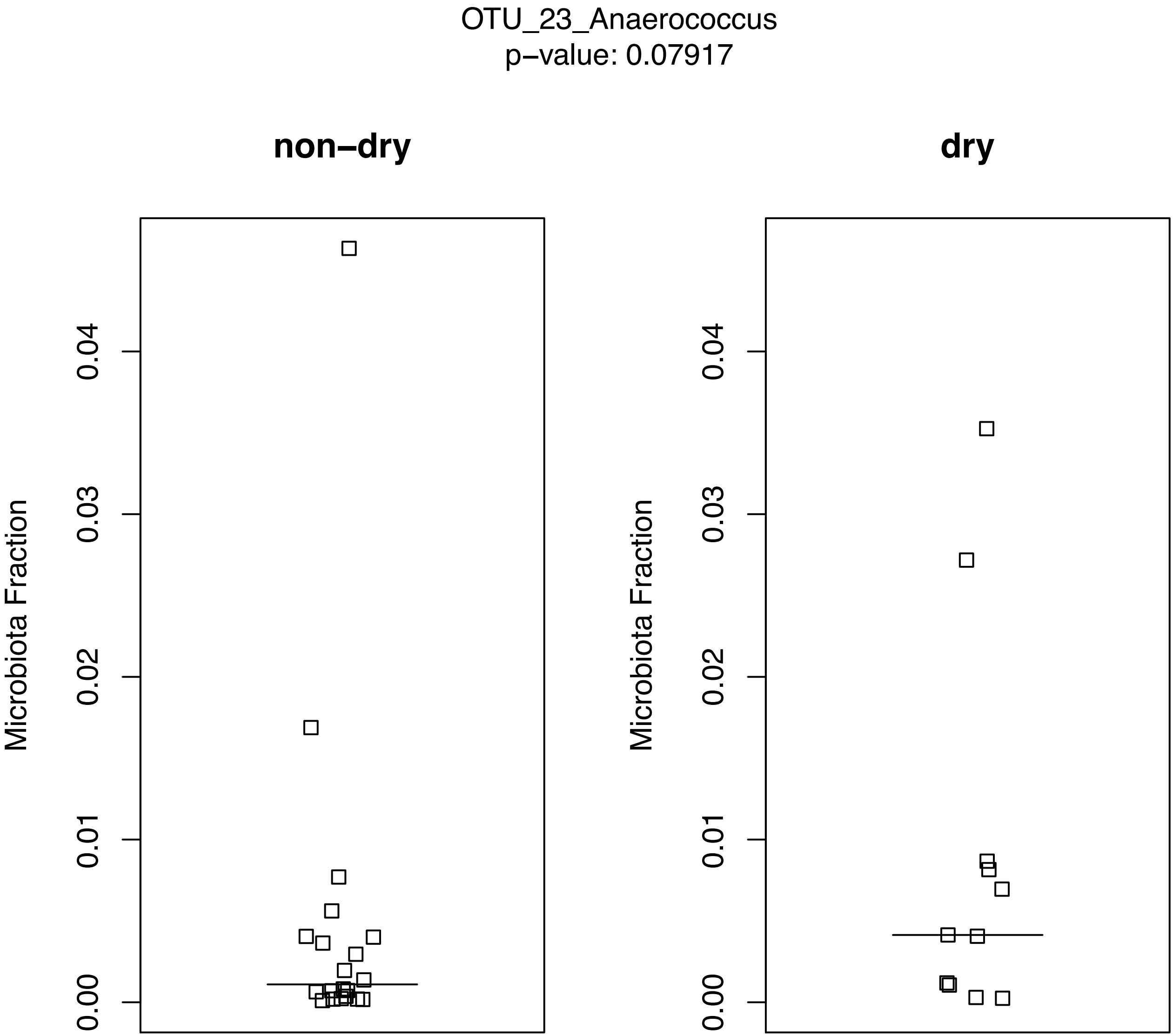
OTU_23_Anaerococcus
p−value: 0.07917
non−dry
dry
Microbiota Fraction
Microbiota Fraction
0.00
0.01
0.02
0.03
0.04
0.00
0.01
0.02
0.03
0.04

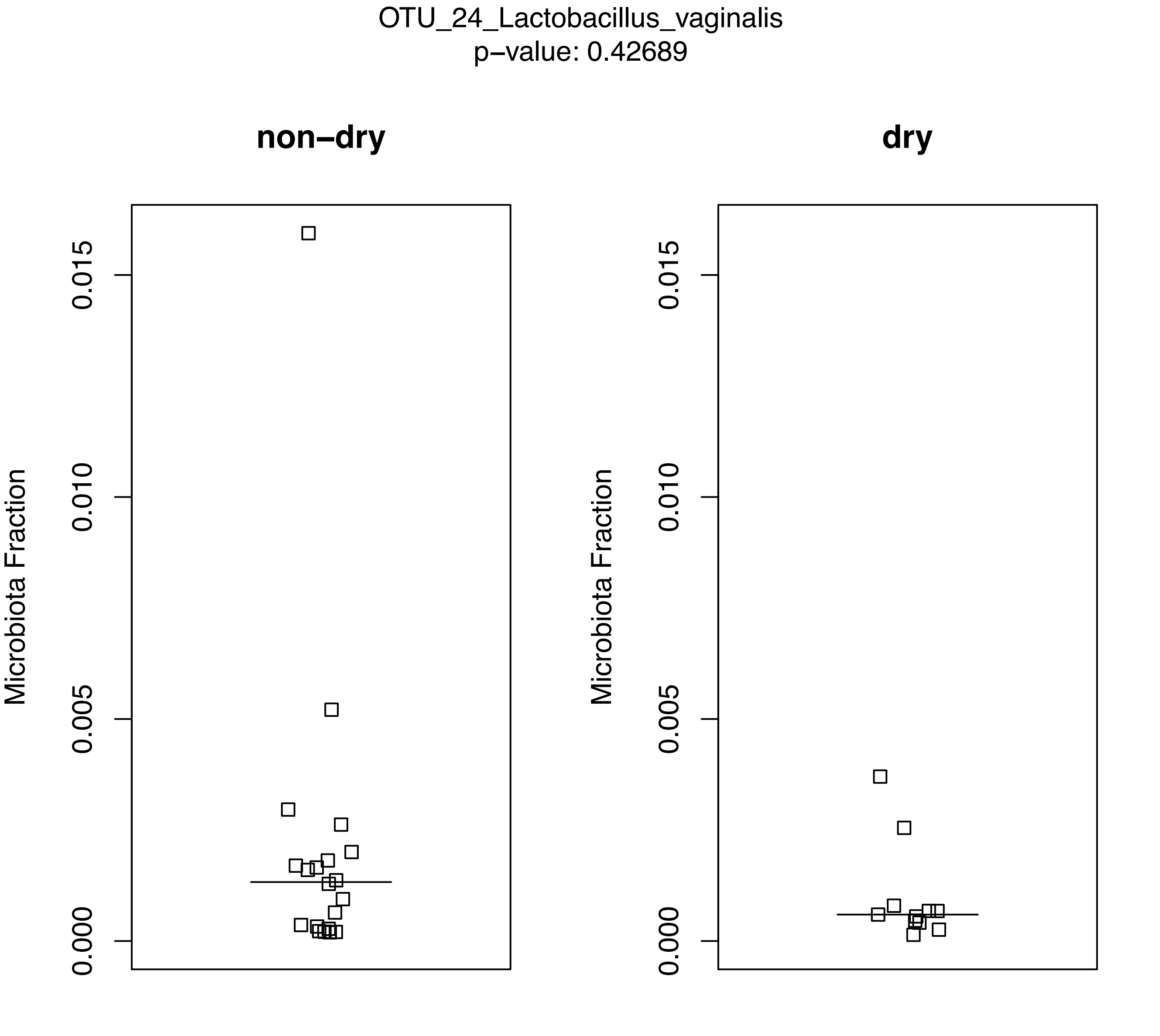

OTU_24_Lactobacillus_vaginalis
p−value: 0.42689
non−dry
dry
Microbiota Fraction
0.000
0.005
0.010
0.015

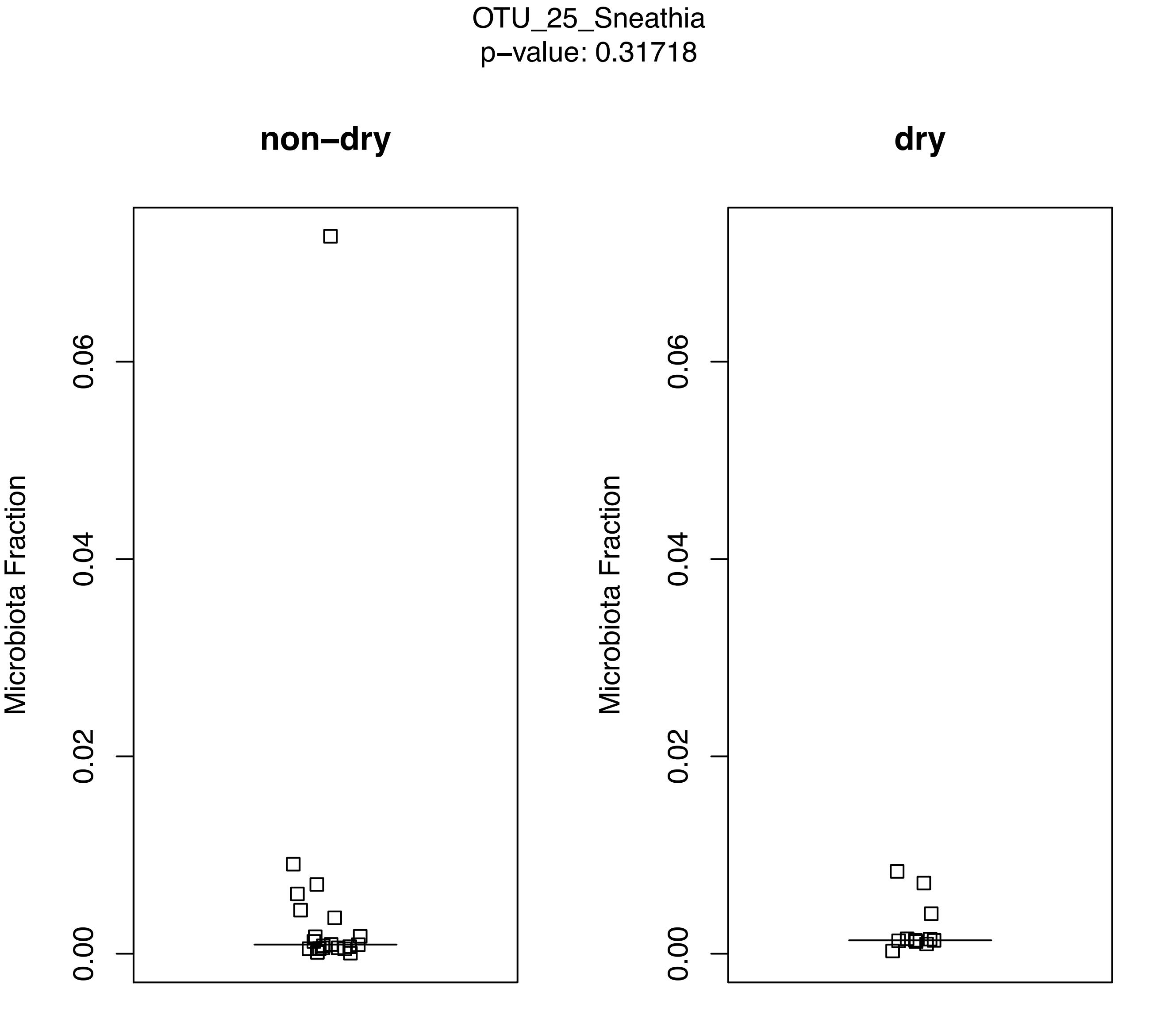
OTU_25_Sneathia
p−value: 0.31718
non−dry
dry
Microbiota Fraction
0.00
0.02
0.04
0.06

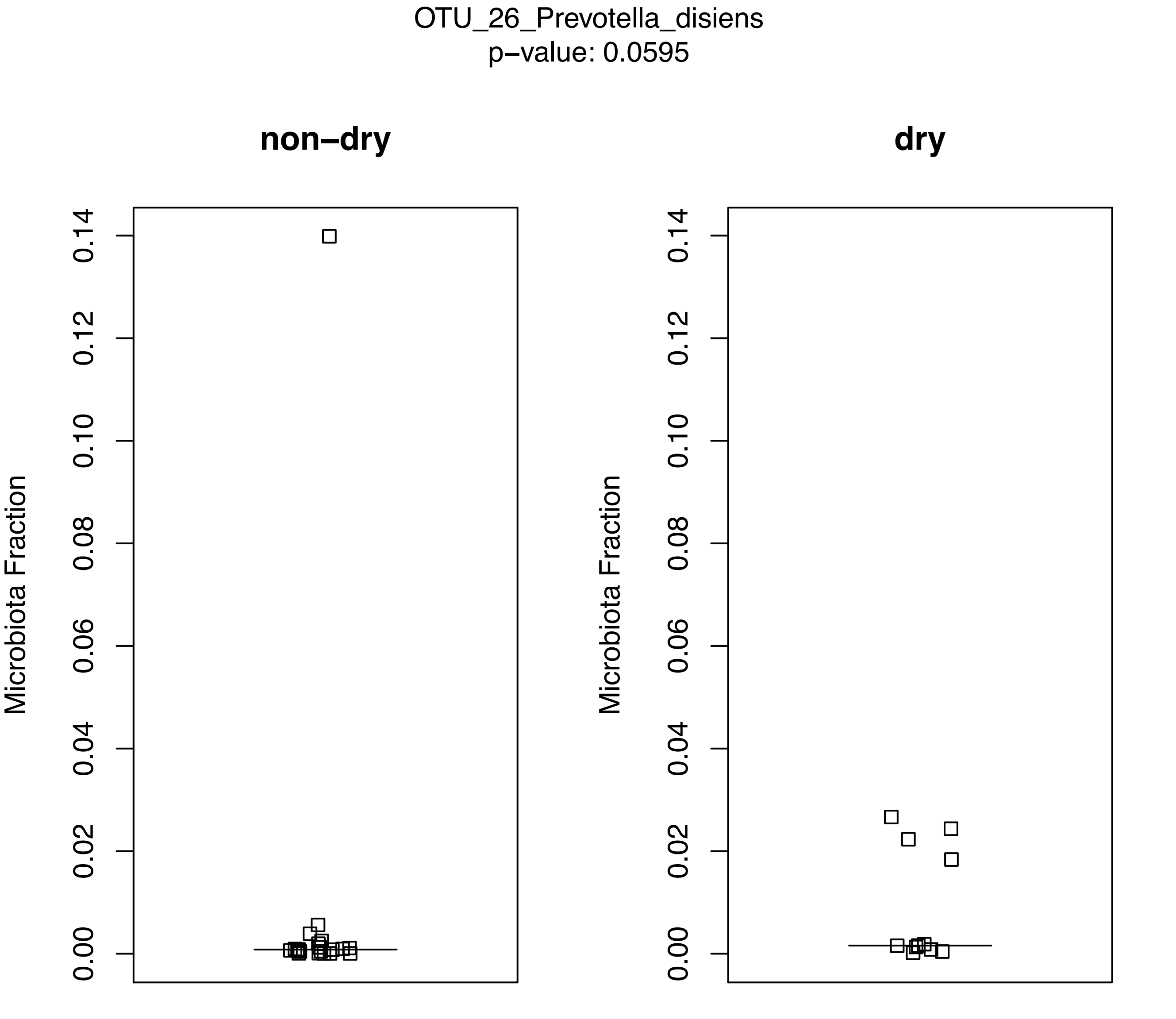
OTU_26_Prevotella_disiens
p−value: 0.0595
non−dry
dry
Microbiota Fraction
0.00
0.02
0.04
0.06
0.08
0.10
0.12
0.14

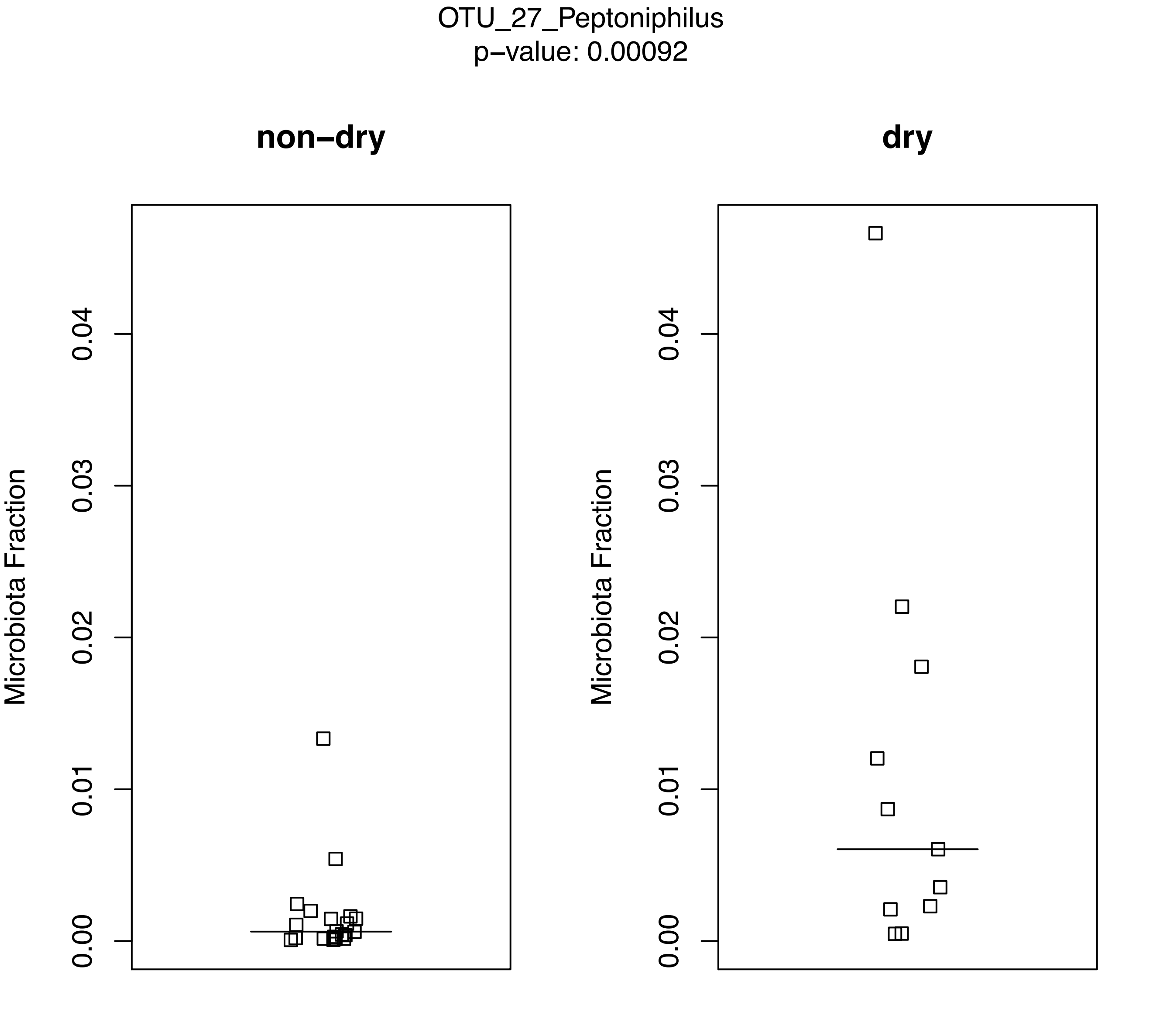
OTU_27_Peptoniphilus
p–value: 0.00092
non–dry
dry
Microbiota Fraction
0.00
0.01
0.02
0.03
0.04
Microbiota Fraction
0.00
0.01
0.02
0.03
0.04

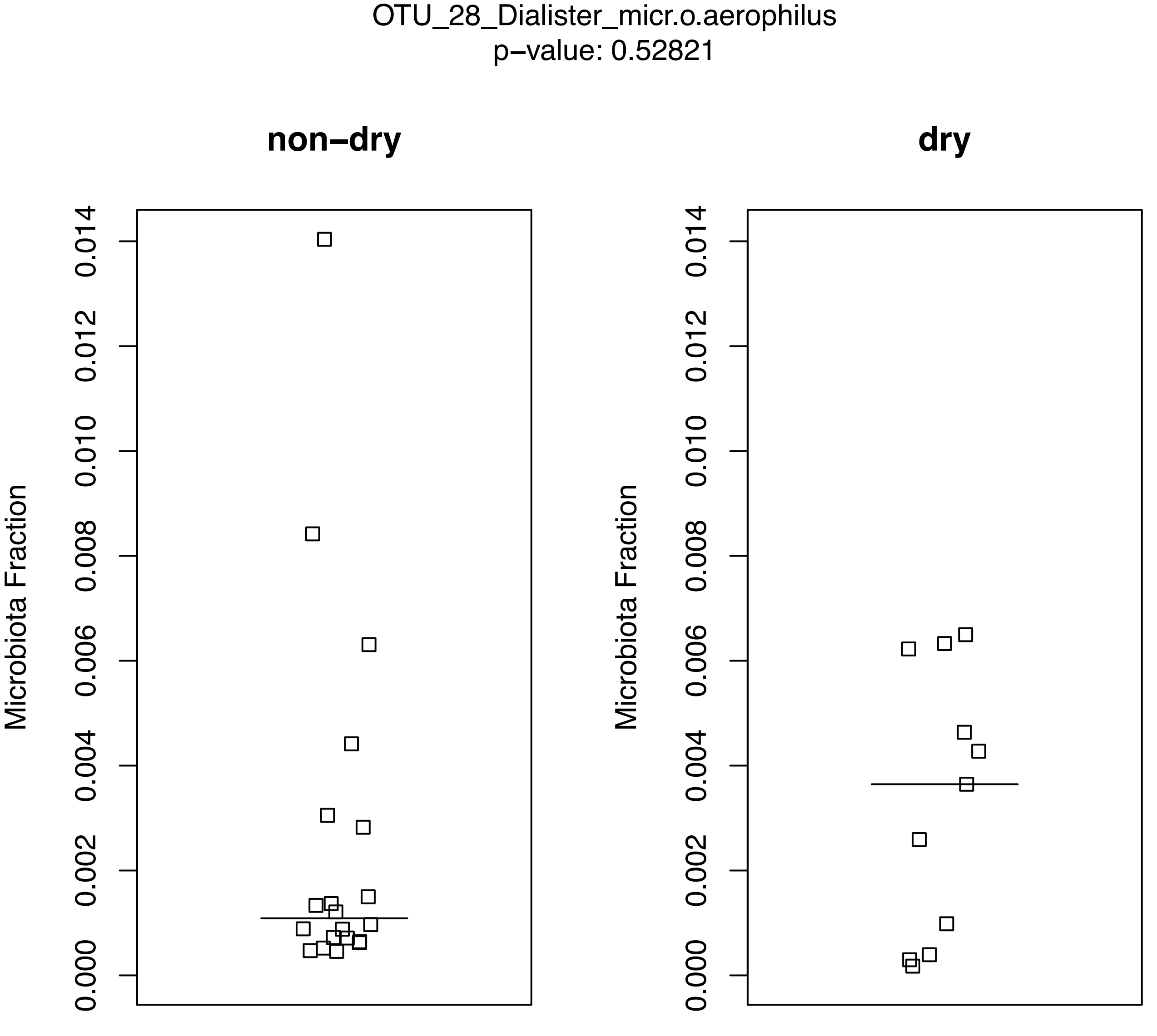

OTU_28_Dialister_micr.o.aerophilus
p−value: 0.52821
non−dry
dry
Microbiota Fraction
Microbiota Fraction
0.000
0.002
0.004
0.006
0.008
0.010
0.012
0.014

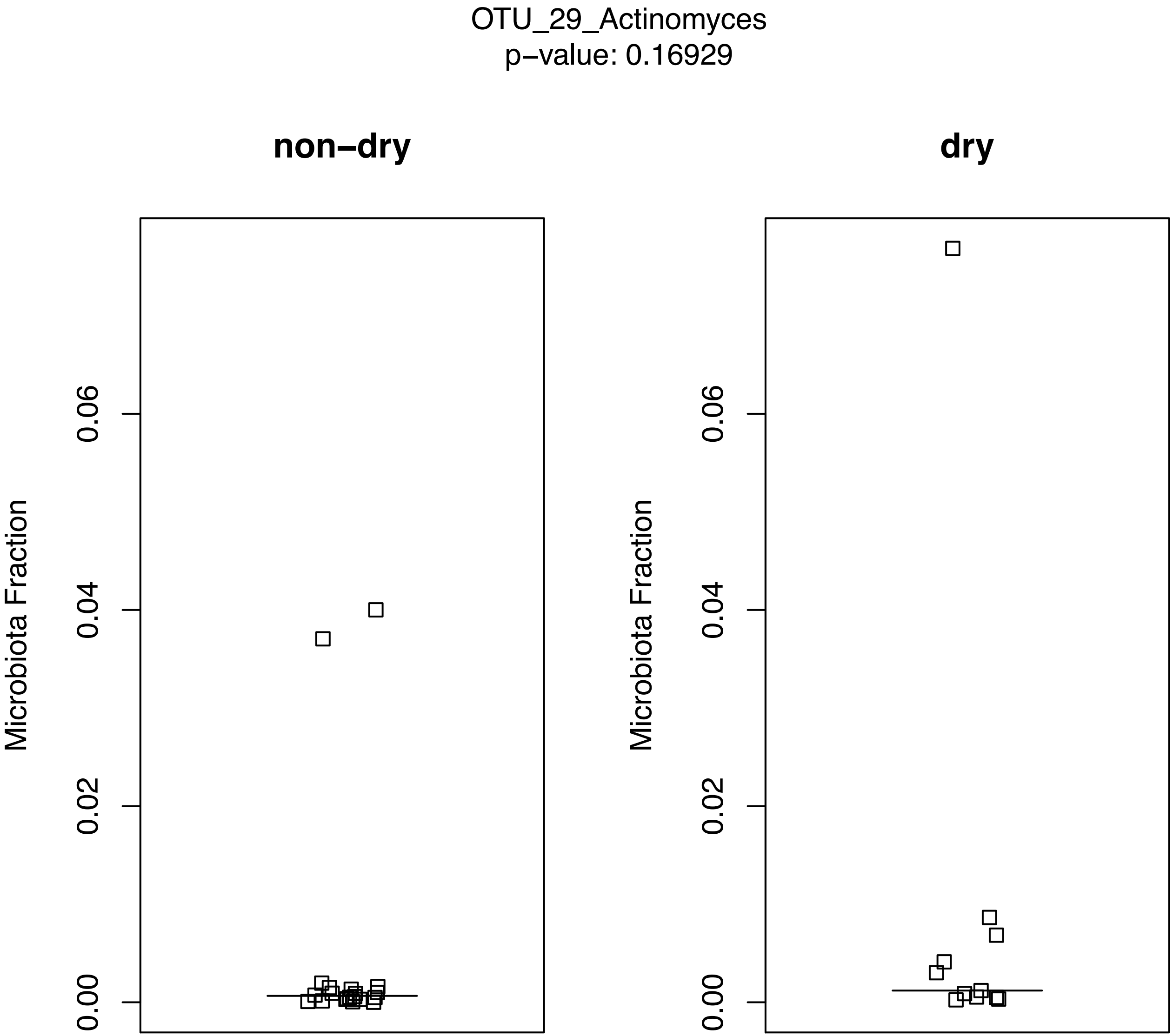
OTU_29_Actinomyces
p−value: 0.16929
non−dry
dry
Microbiota Fraction
0.00
0.02
0.04
0.06
Microbiota Fraction
0.00
0.02
0.04
0.06

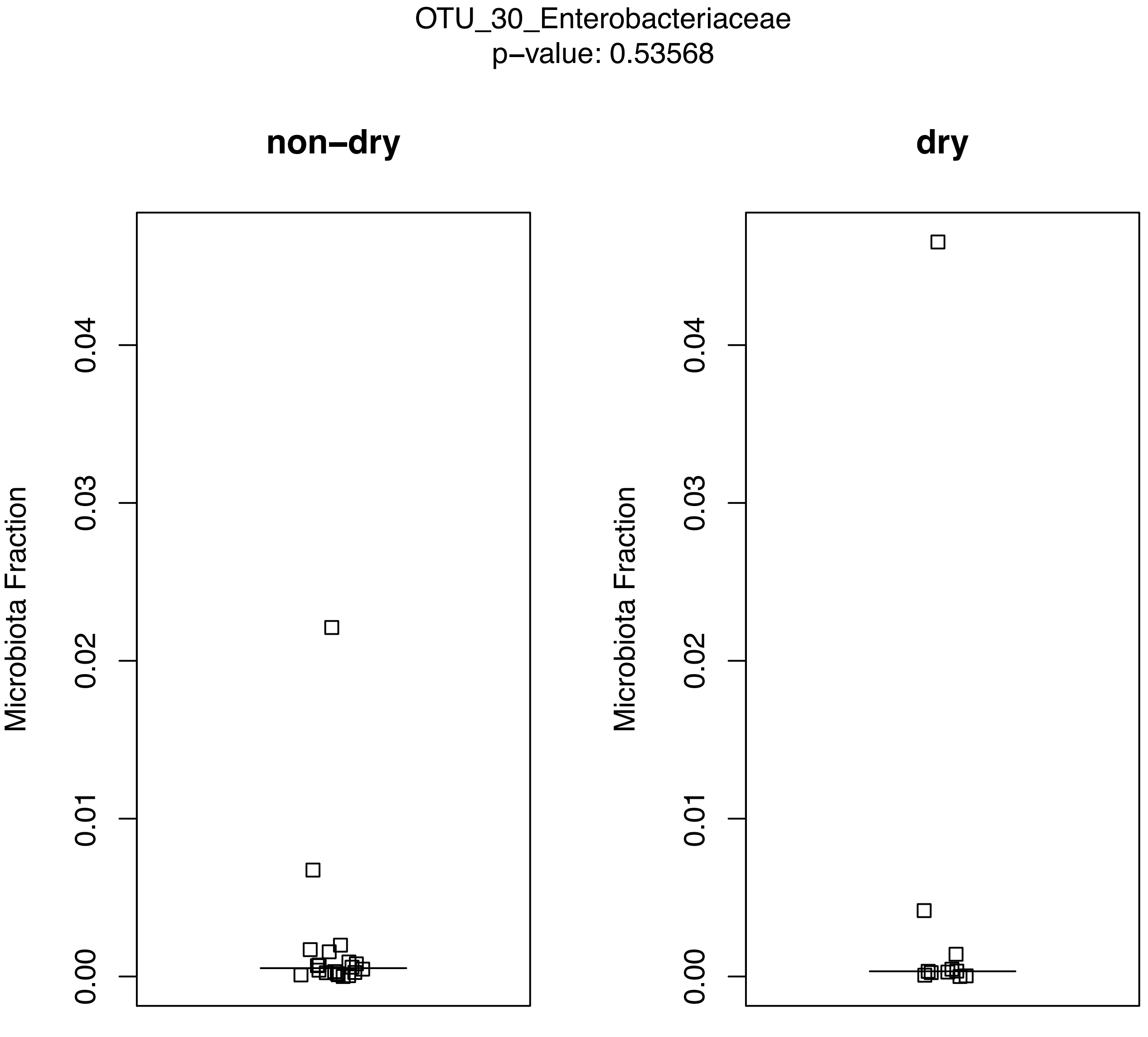

OTU_30_Enterobacteriaceae
p−value: 0.53568
non−dry
dry
Microbiota Fraction
0.00
0.01
0.02
0.03
0.04

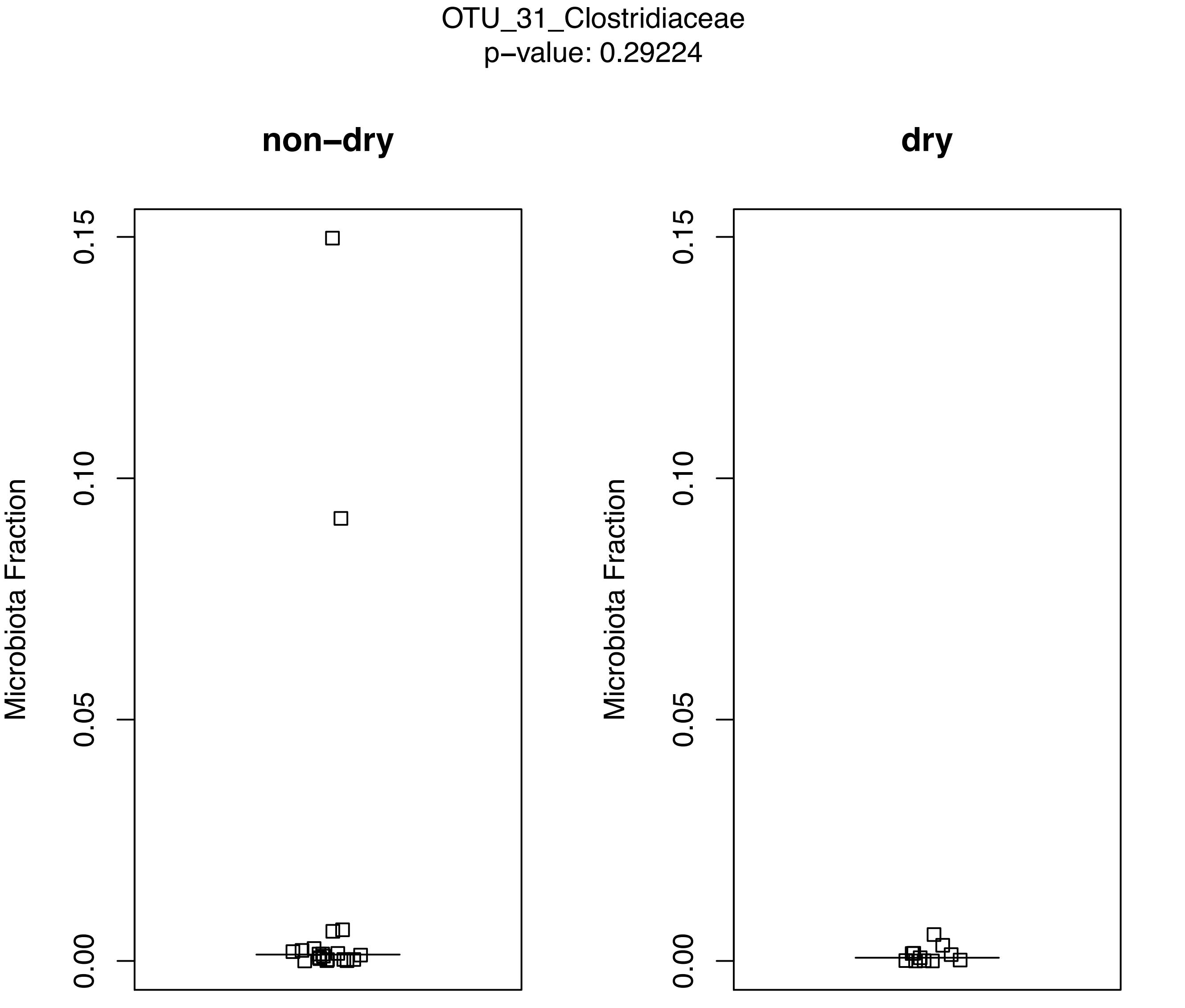

OTU_31_Clostridiaceae
p−value: 0.29224
non−dry
dry
Microbiota Fraction
0.00
0.05
0.10
0.15

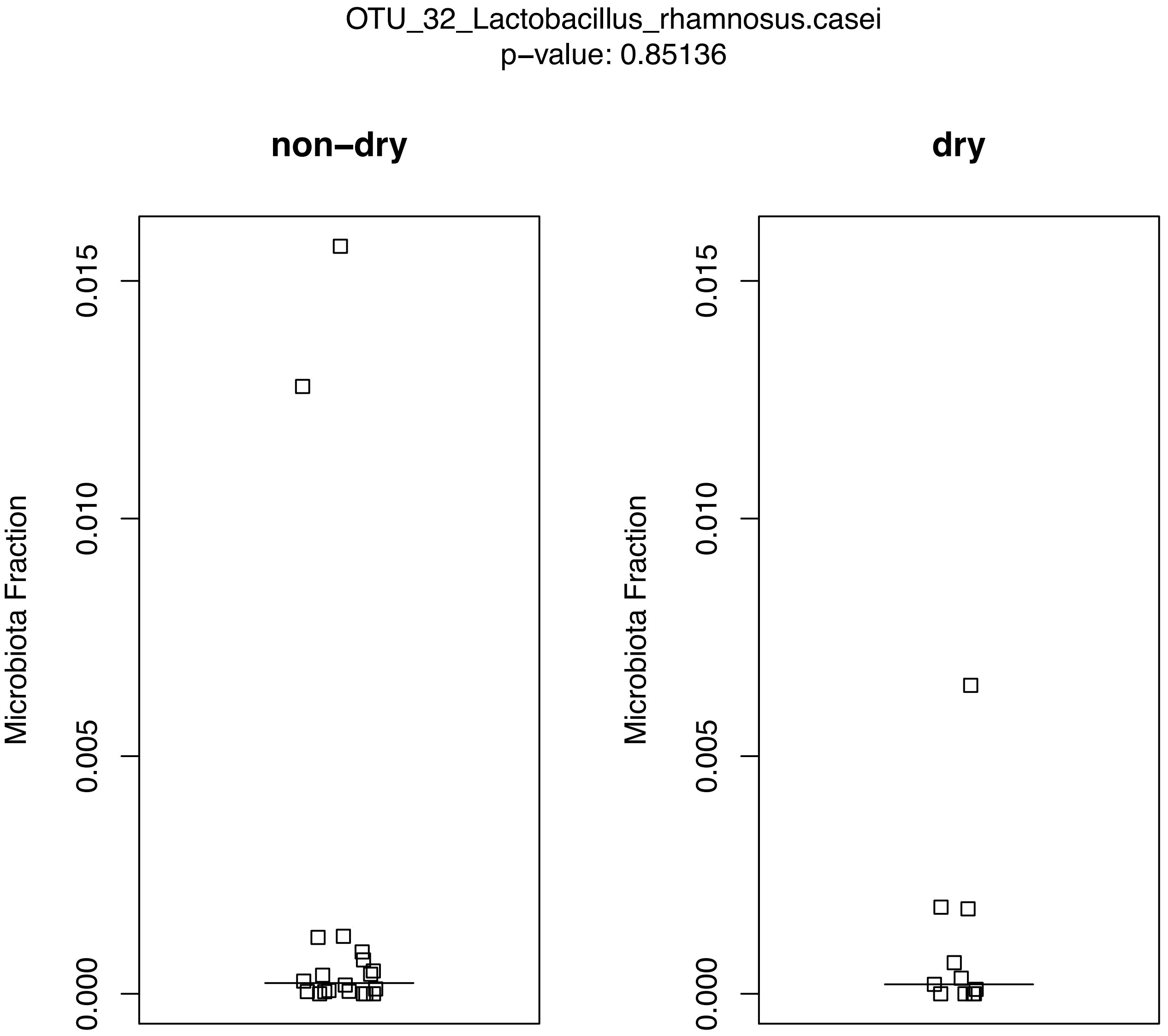

OTU_32_Lactobacillus_rhamnosus.casei
p−value: 0.85136
non−dry
dry
Microbiota Fraction
0.000
0.005
0.010
0.015

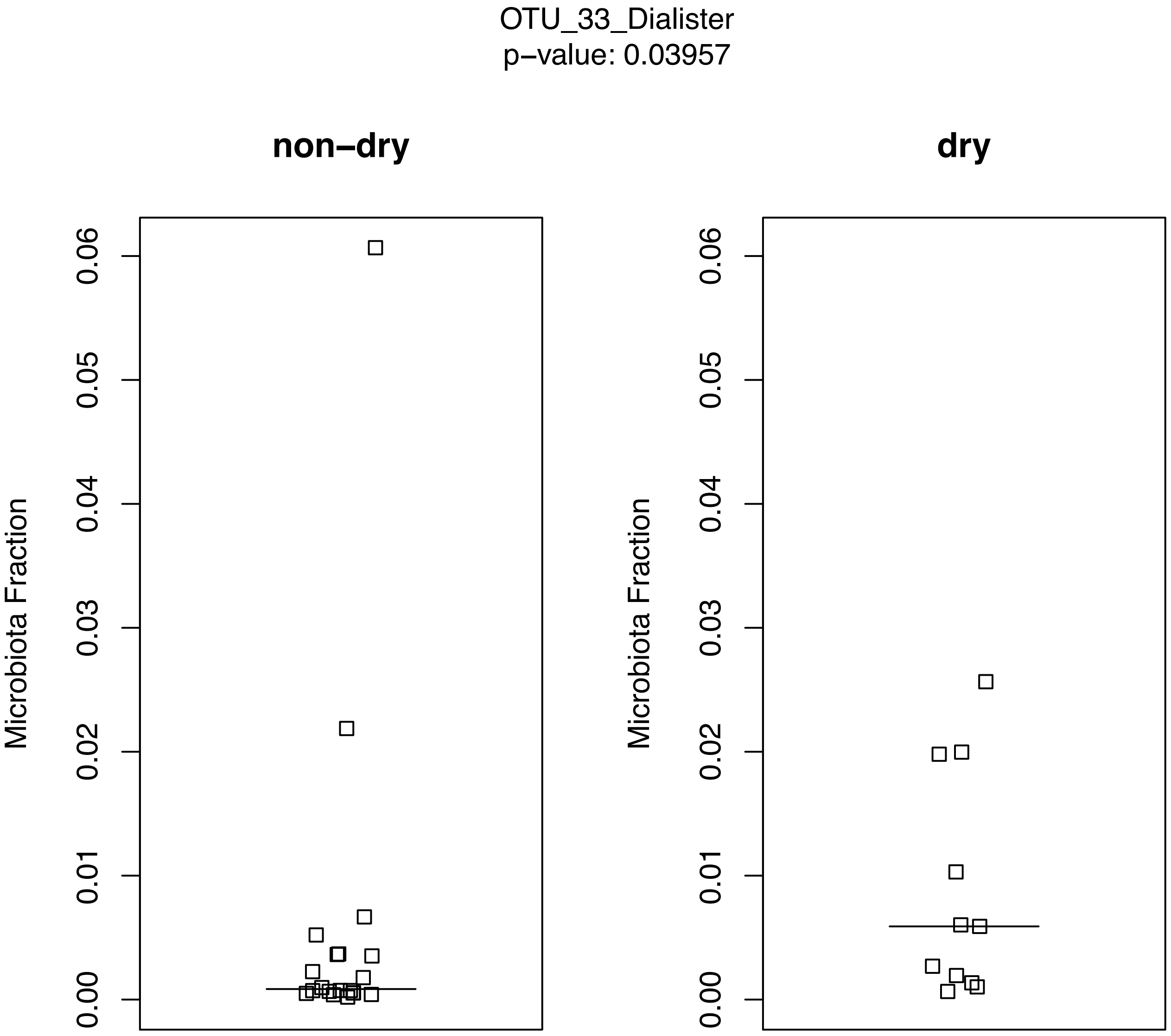
OTU_33_Dialister
p–value: 0.03957
non–dry
dry
Microbiota Fraction
0.00
0.01
0.02
0.03
0.04
0.05
0.06
Microbiota Fraction
0.00
0.01
0.02
0.03
0.04
0.05
0.06

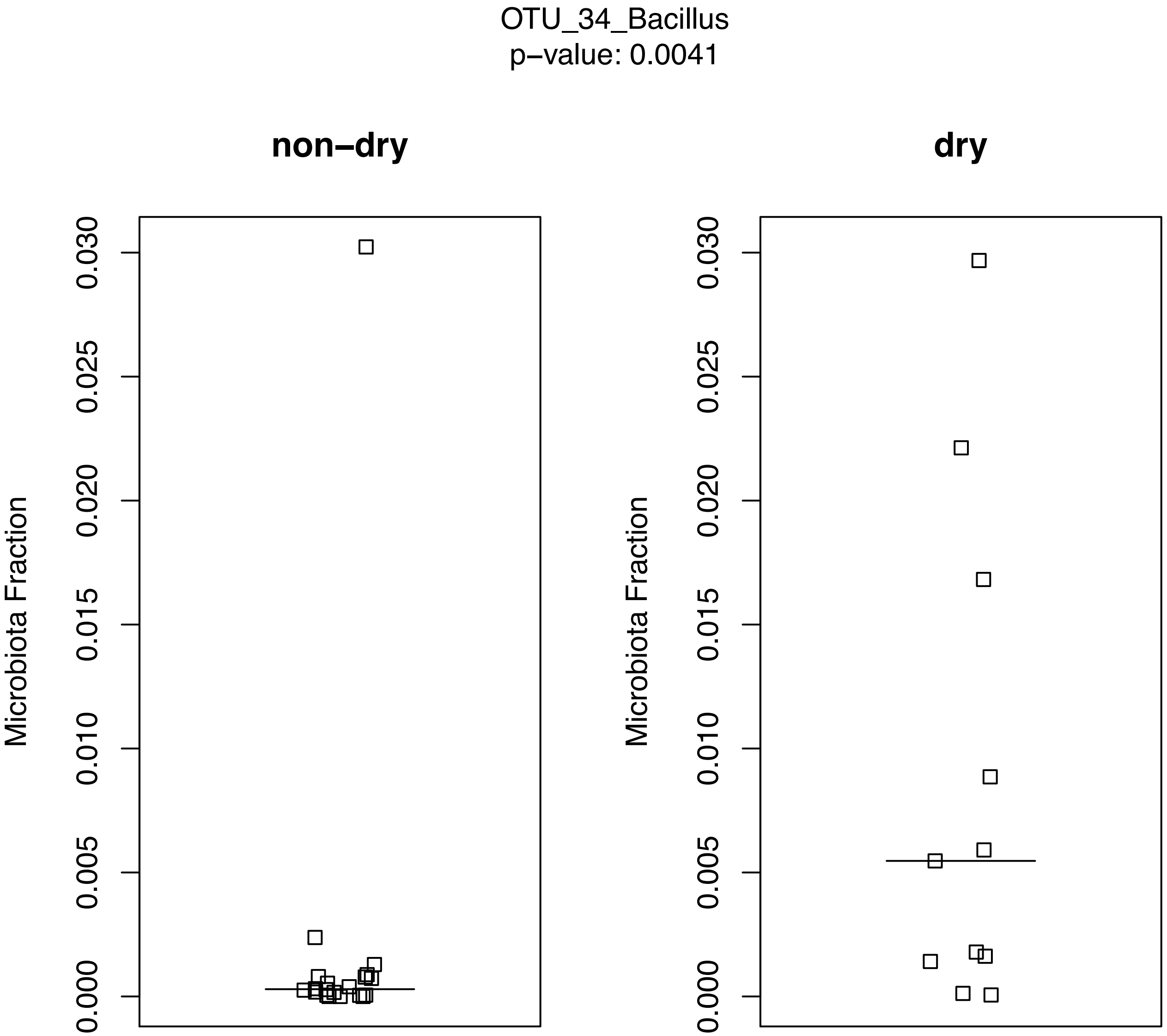

OTU_34_Bacillus
p−value: 0.0041
non−dry
dry
Microbiota Fraction
0.000
0.005
0.010
0.015
0.020
0.025
0.030

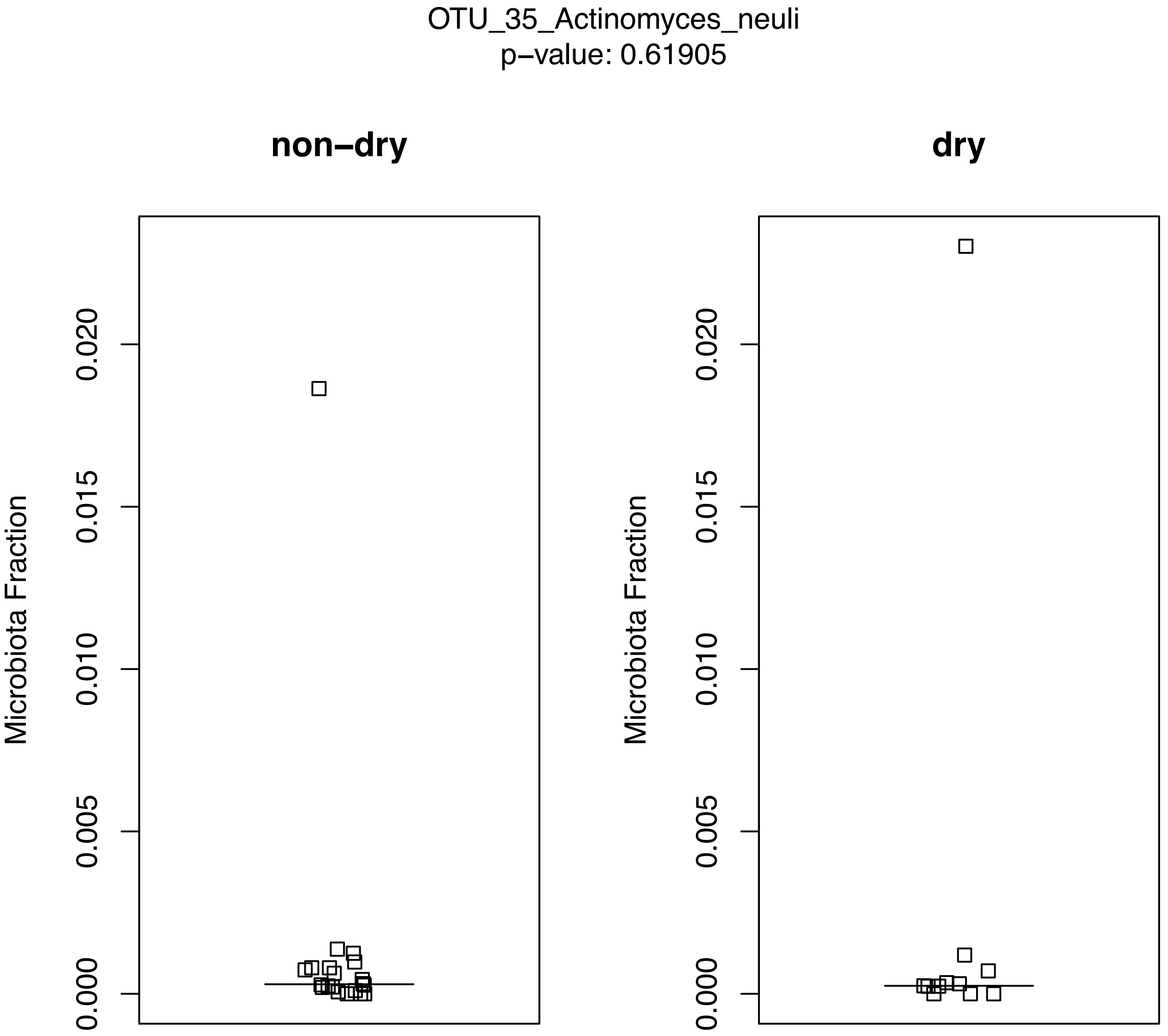
OTU_35_Actinomyces_neuli
p−value: 0.61905
non−dry
dry
Microbiota Fraction
Microbiota Fraction
0.000
0.005
0.010
0.015
0.020
0.000
0.005
0.010
0.015
0.020

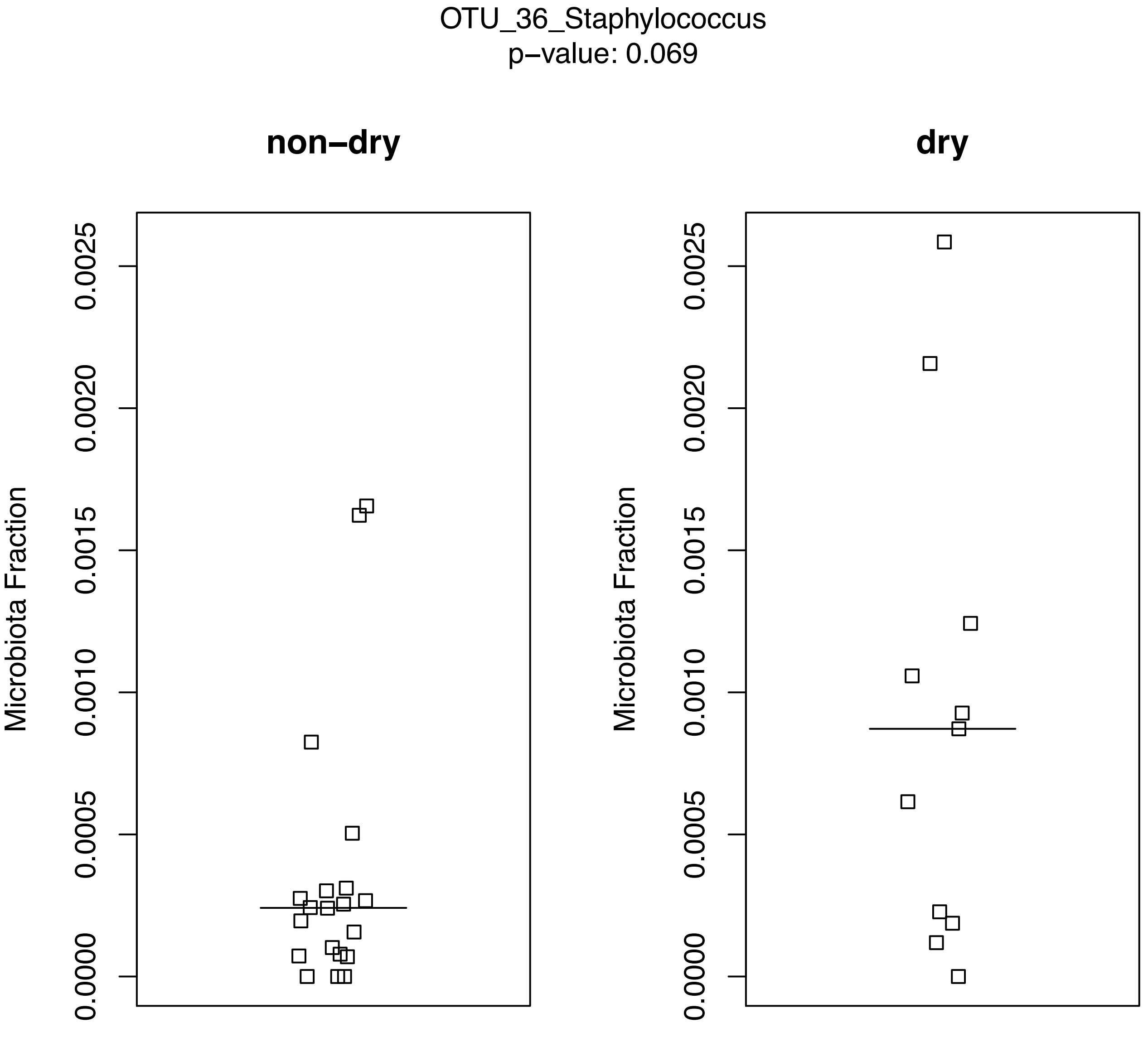
OTU_36_Staphylococcus
p−value: 0.069
non−dry
dry
Microbiota Fraction
0.0000
0.0005
0.0010
0.0015
0.0020
0.0025
Microbiota Fraction
0.0000
0.0005
0.0010
0.0015
0.0020
0.0025

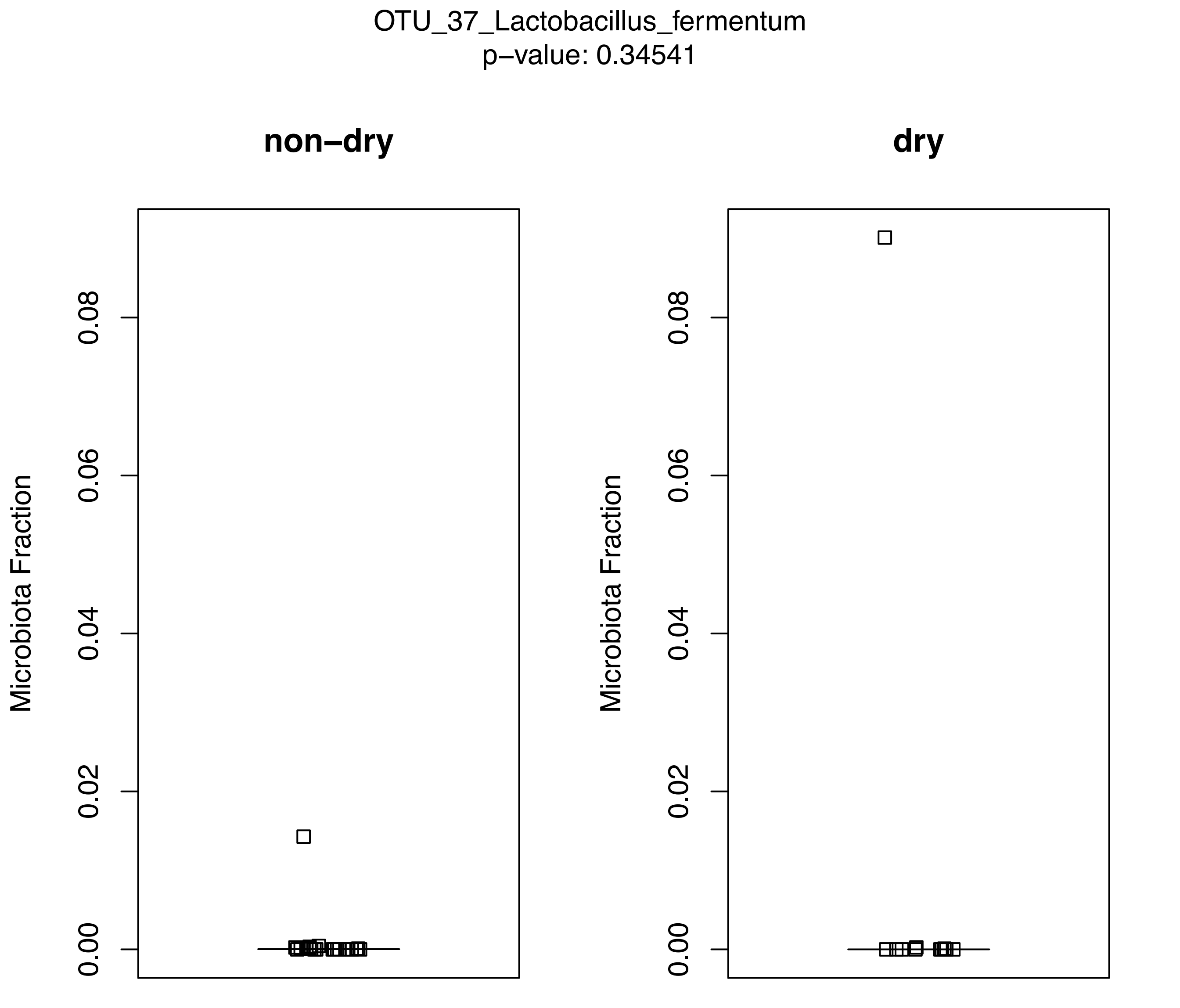

OTU_37_Lactobacillus_fermentum
p−value: 0.34541
non−dry
dry
Microbiota Fraction
0.00
0.02
0.04
0.06
0.08

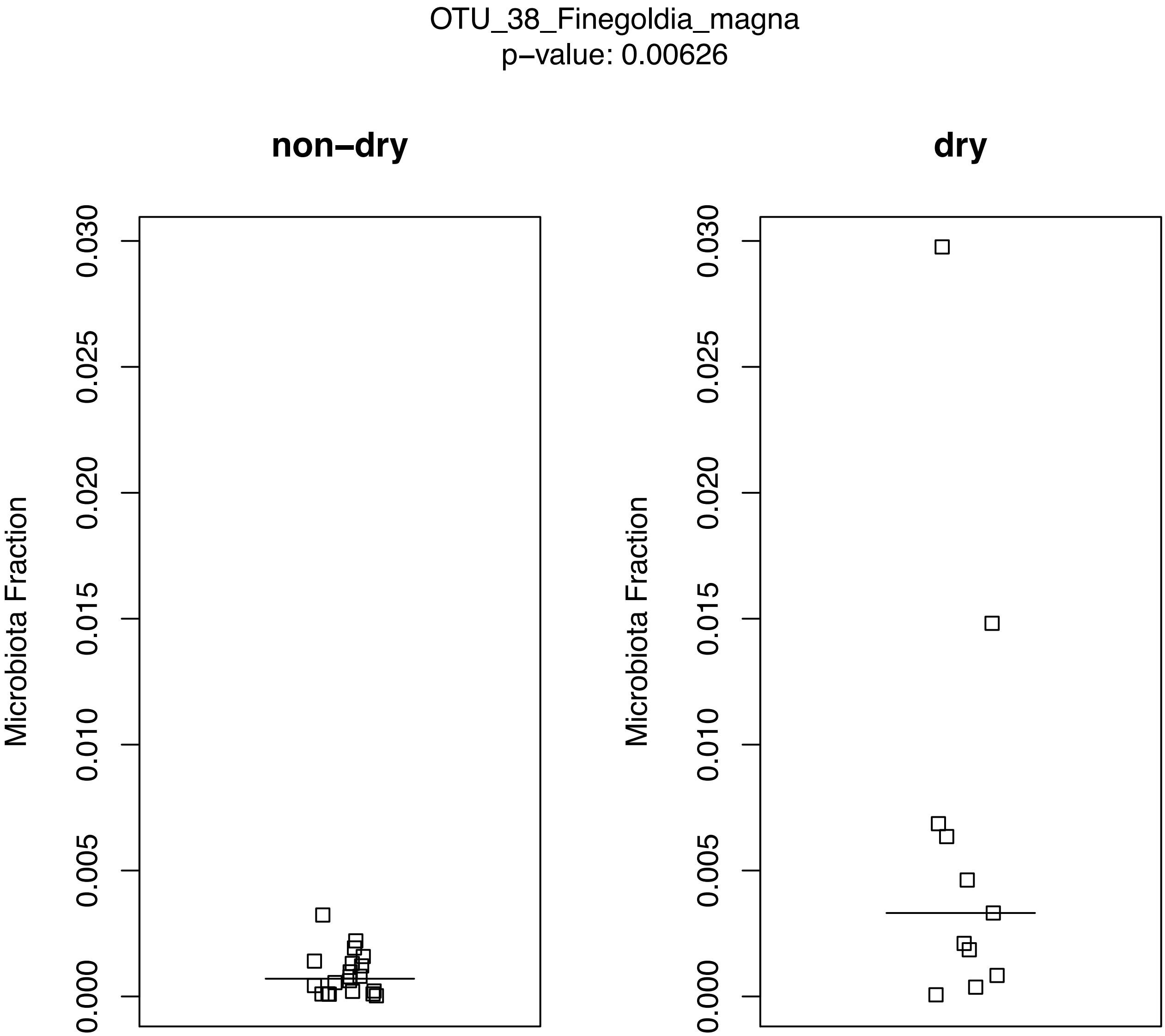
OTU_38_Finegoldia_magna
p−value: 0.00626
non−dry
dry
Microbiota Fraction
Microbiota Fraction
0.000
0.005
0.010
0.015
0.020
0.025
0.030
0.000
0.005
0.010
0.015
0.020
0.025
0.030

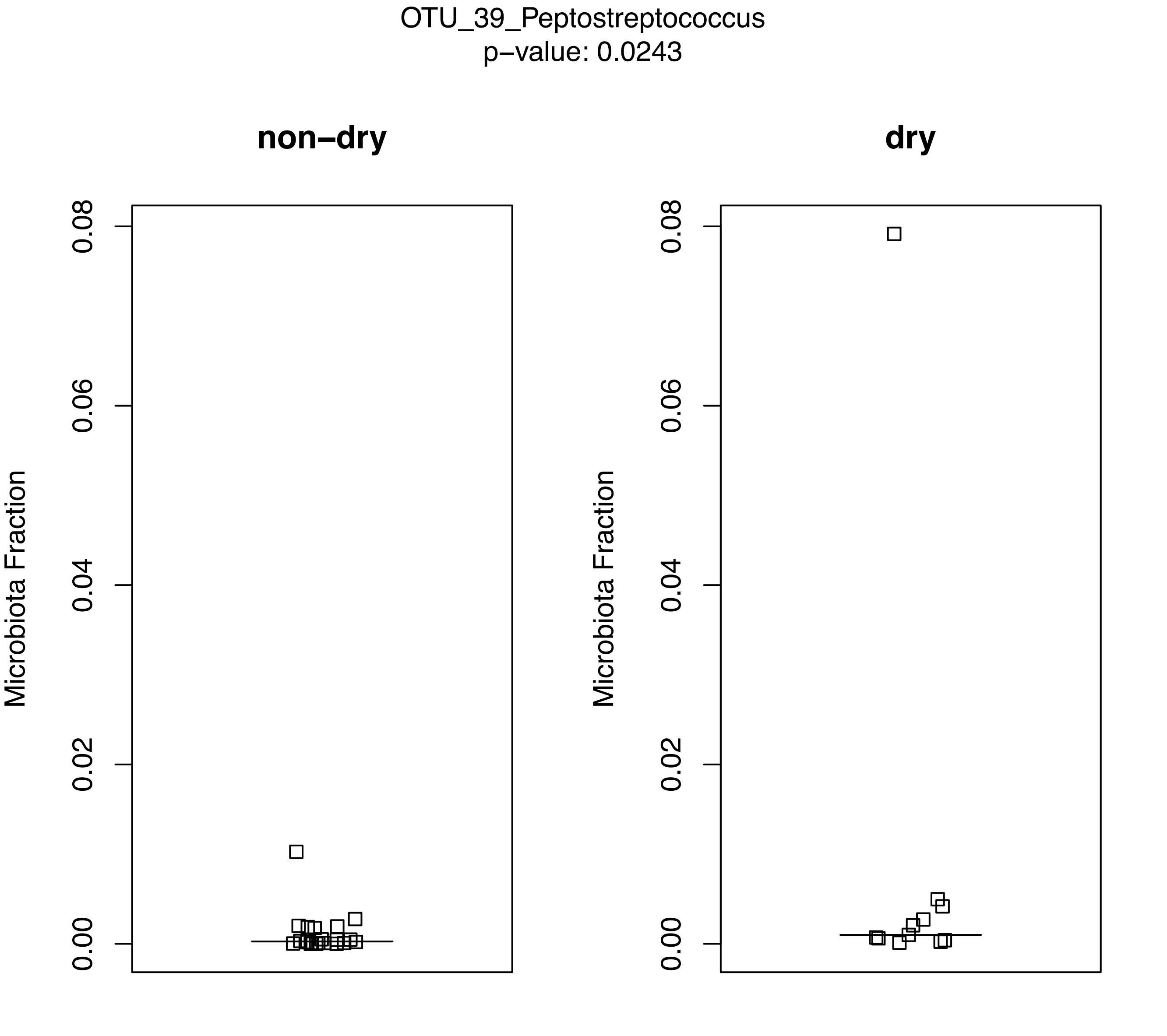

OTU_39_Peptostreptococcus
p–value: 0.0243
non–dry
dry
Microbiota Fraction
0.00
0.02
0.04
0.06
0.08

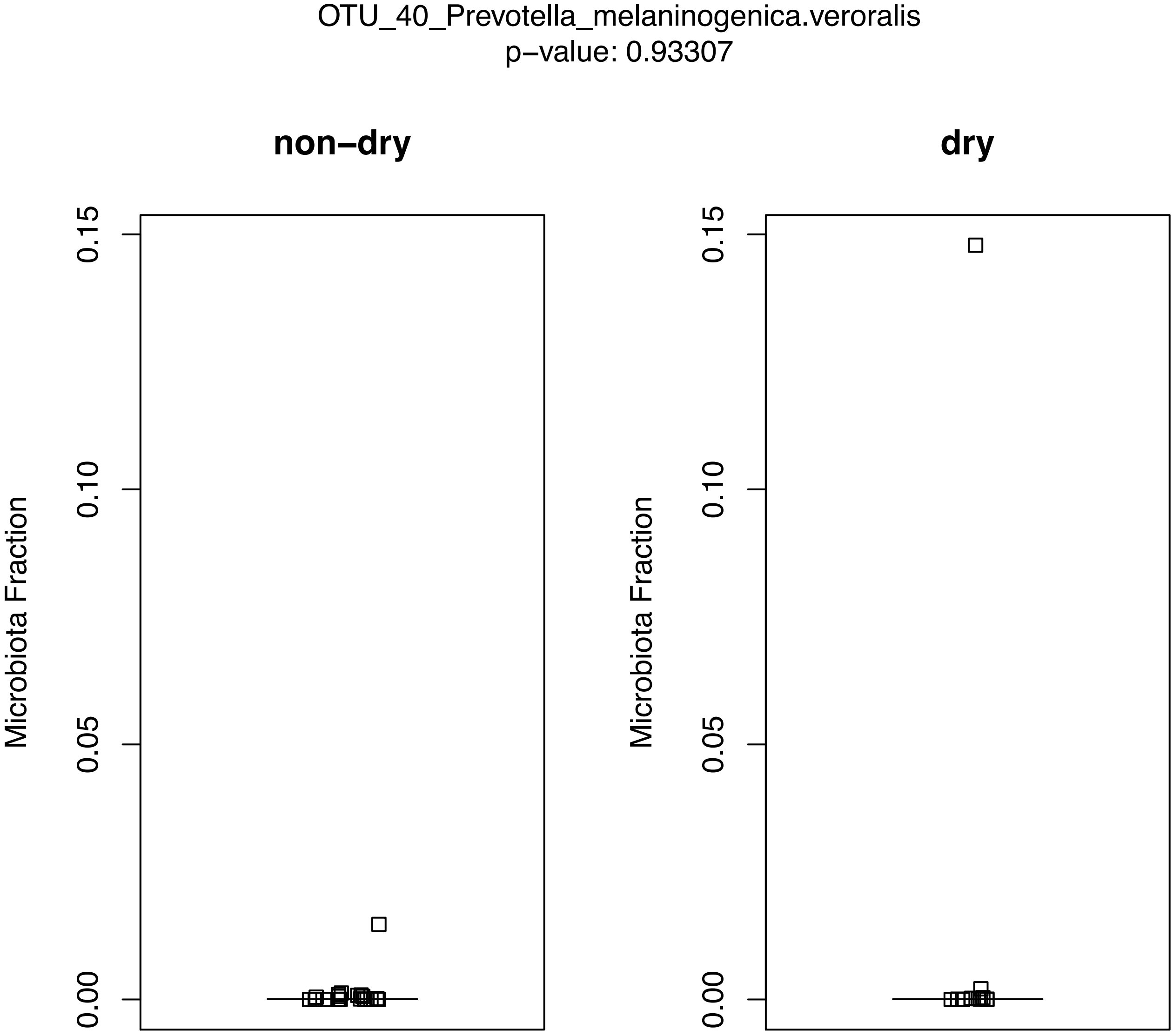
OTU_40_Prevotella_melaninogenica.veroralis
p−value: 0.93307
non−dry
dry
Microbiota Fraction
0.00
0.05
0.10
0.15
Microbiota Fraction
0.00
0.05
0.10
0.15

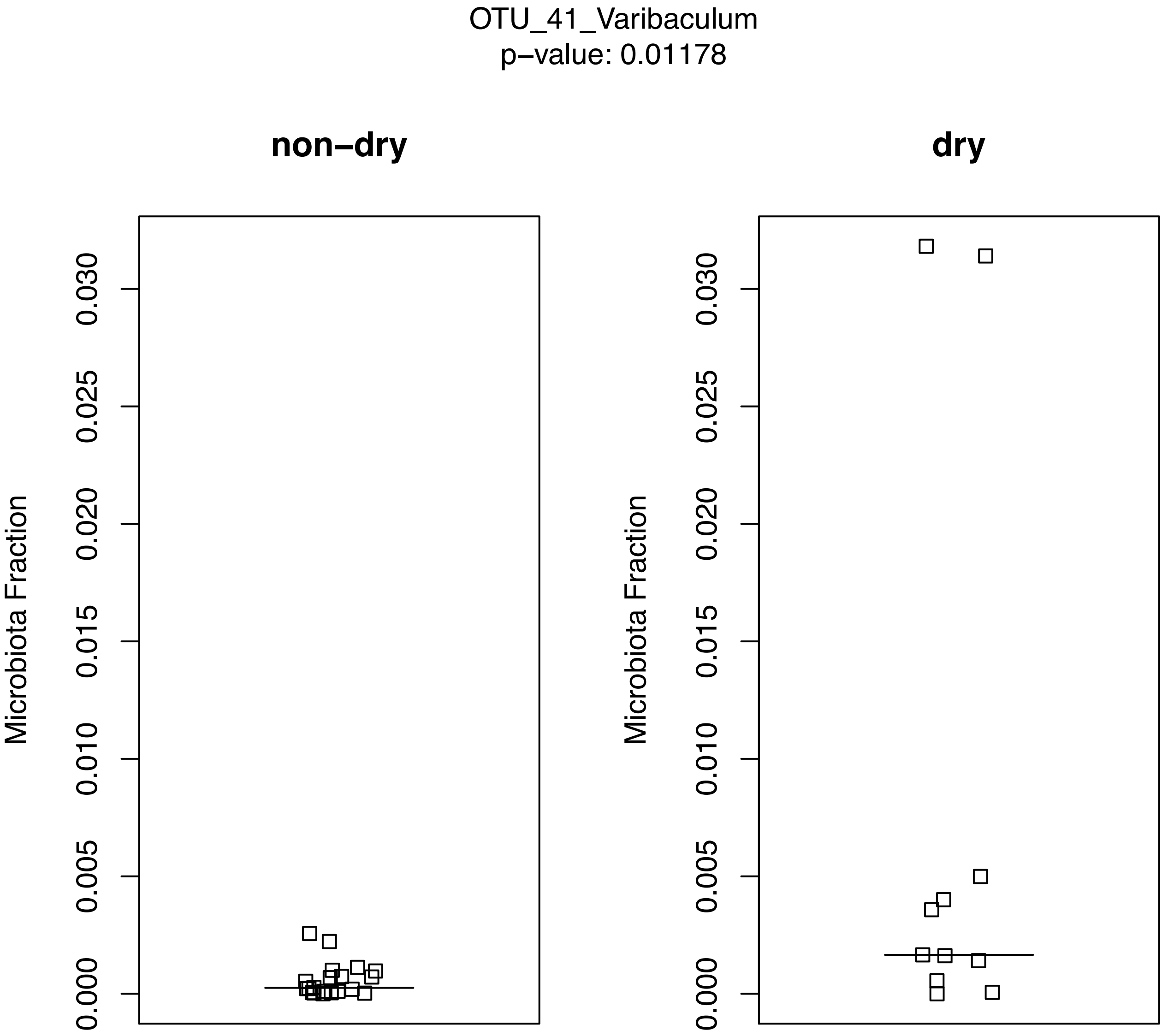

OTU_41_Varibaculum
p−value: 0.01178
non−dry
dry
Microbiota Fraction
0.000
0.005
0.010
0.015
0.020
0.025
0.030

OTU_42_Parvimonas_micra
p−value: 0.17962

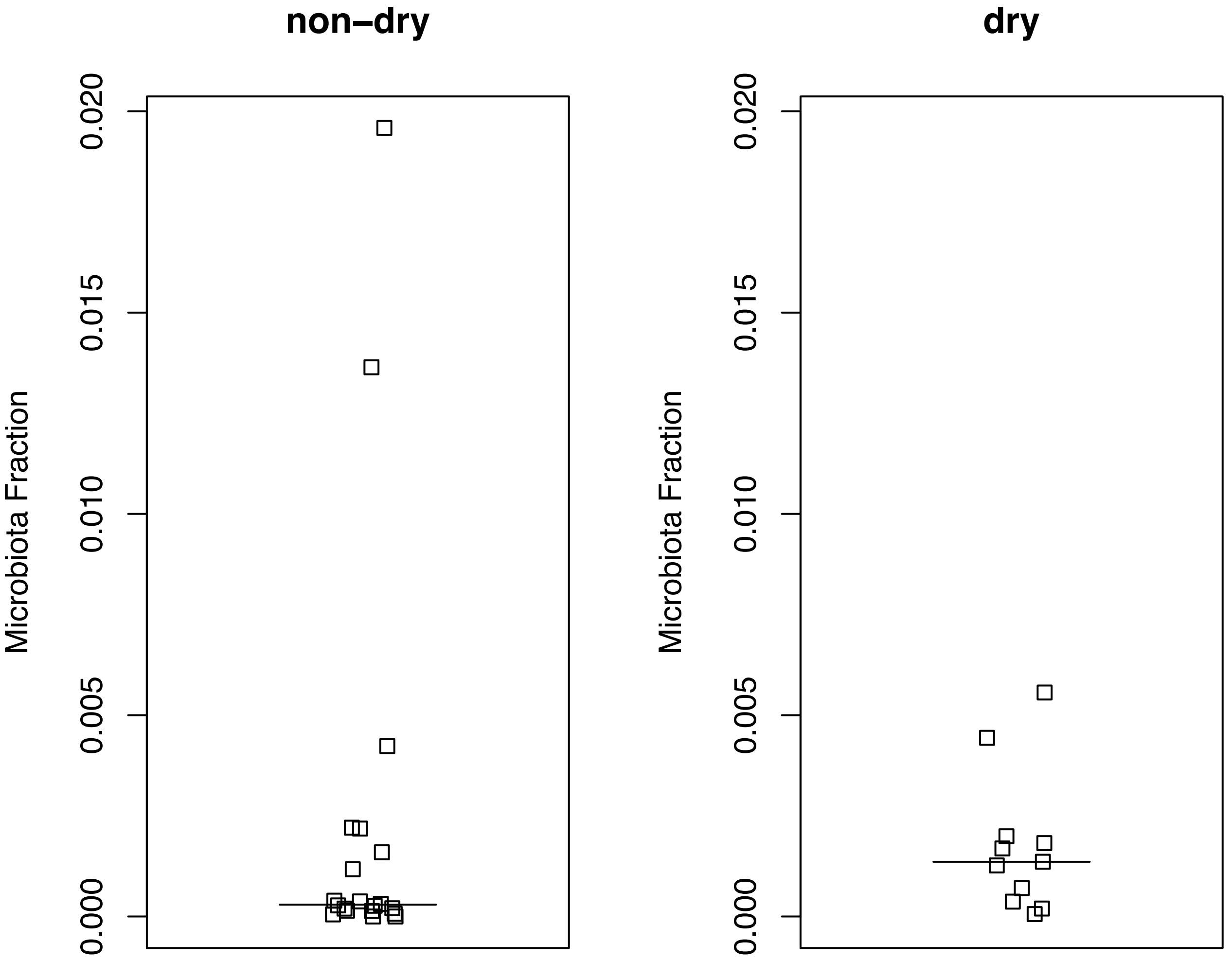

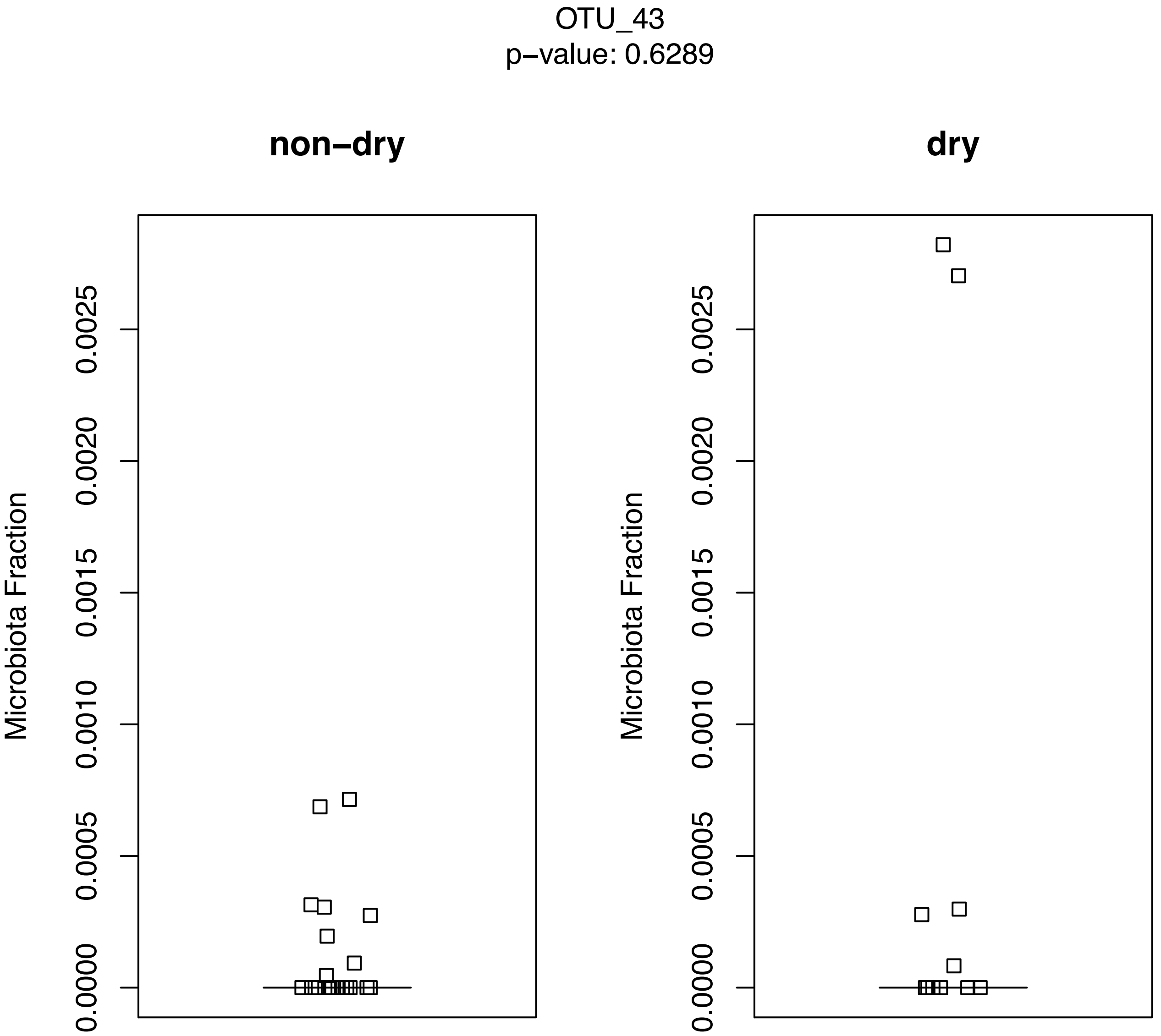

OTU_43
p−value: 0.6289
non−dry
dry
Microbiota Fraction
0.0000
0.0005
0.0010
0.0015
0.0020
0.0025
Microbiota Fraction
0.0000
0.0005
0.0010
0.0015
0.0020
0.0025

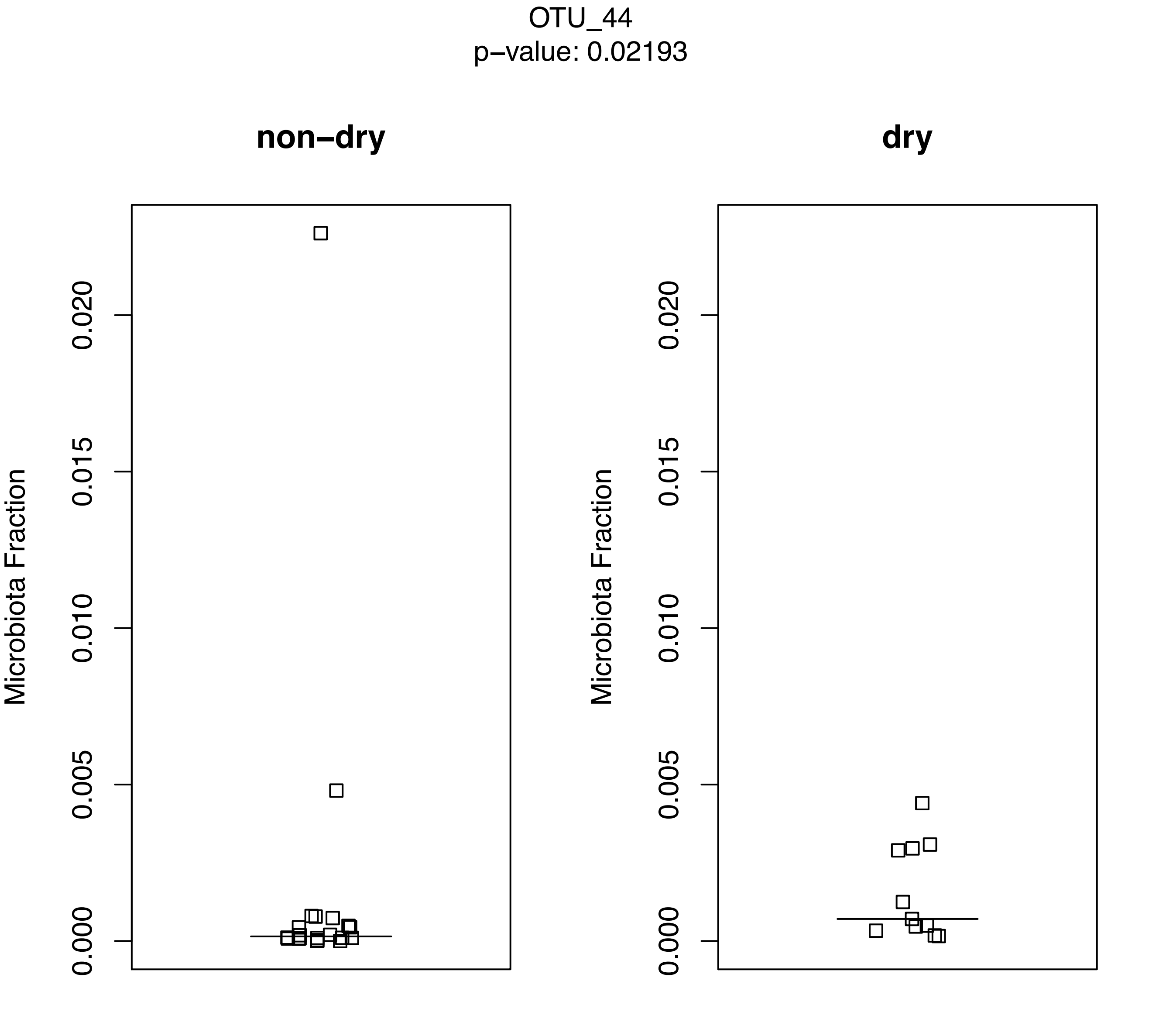

OTU_44
p−value: 0.02193
non−dry
dry
Microbiota Fraction
0.000
0.005
0.010
0.015
0.020

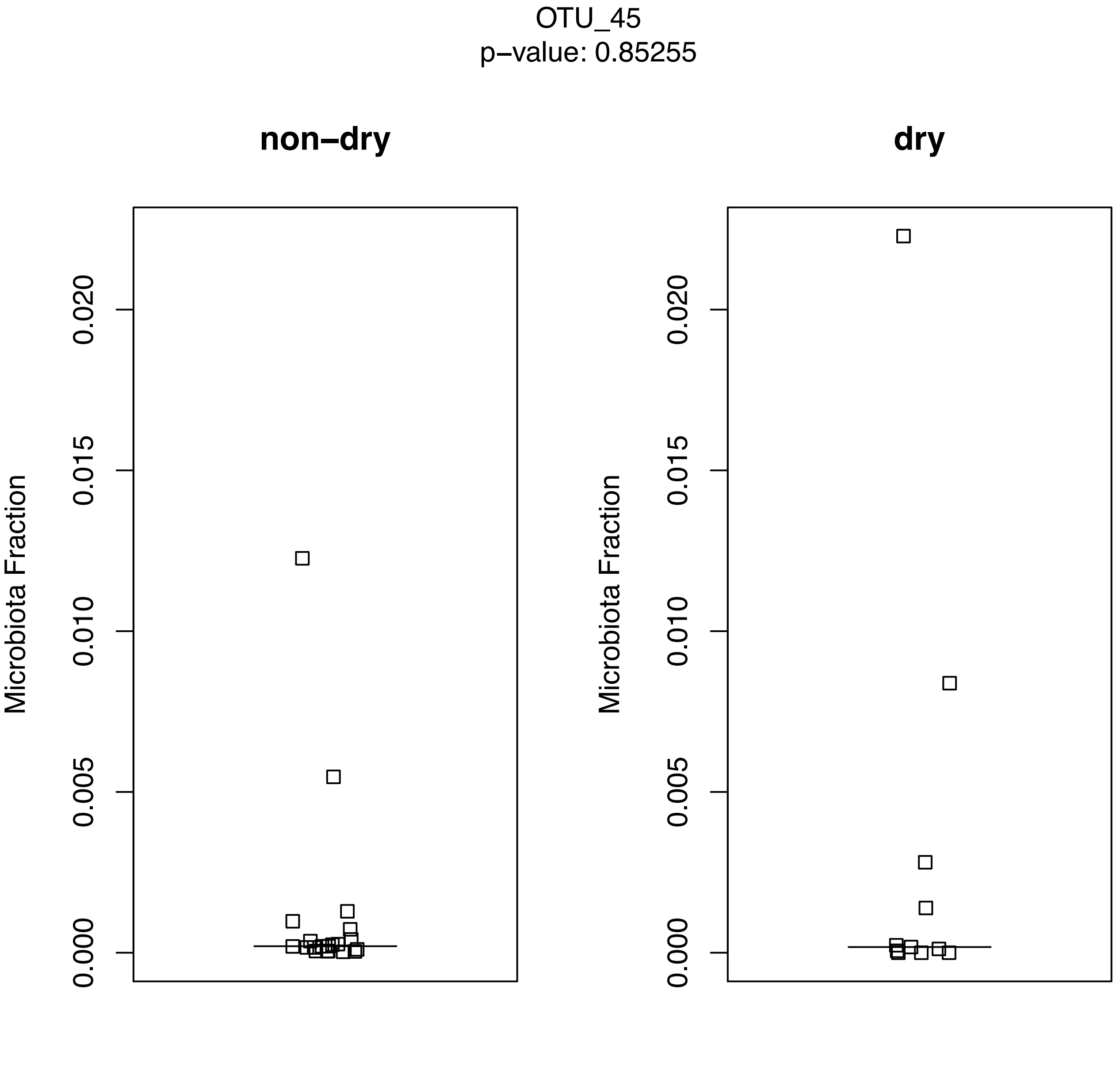
OTU_45
p−value: 0.85255
non−dry
dry
Microbiota Fraction
0.000
0.005
0.010
0.015
0.020
Microbiota Fraction
0.000
0.005
0.010
0.015
0.020

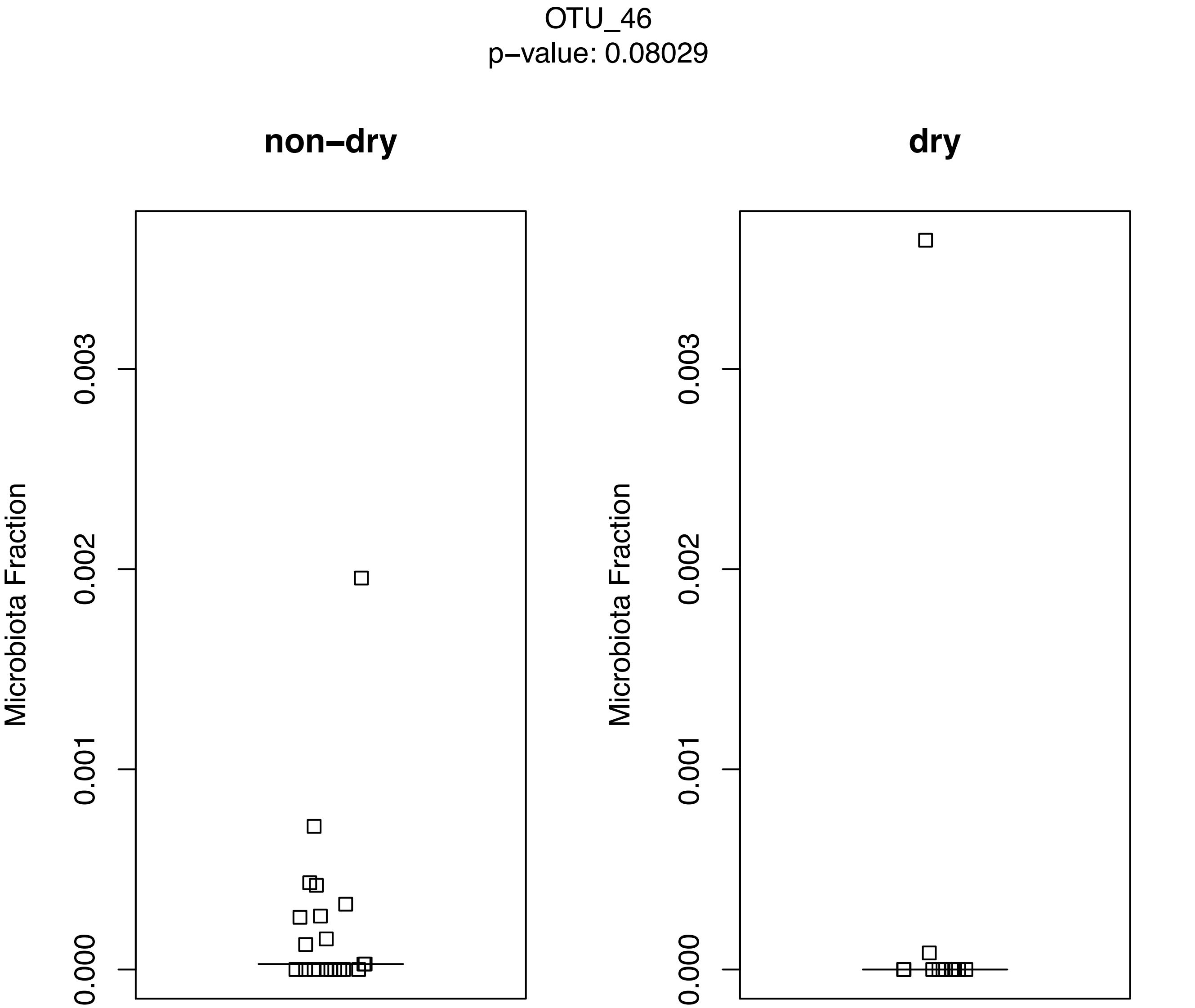
OTU_46
p−value: 0.08029
non−dry
dry
Microbiota Fraction
0.000
0.001
0.002
0.003
Microbiota Fraction
0.000
0.001
0.002
0.003

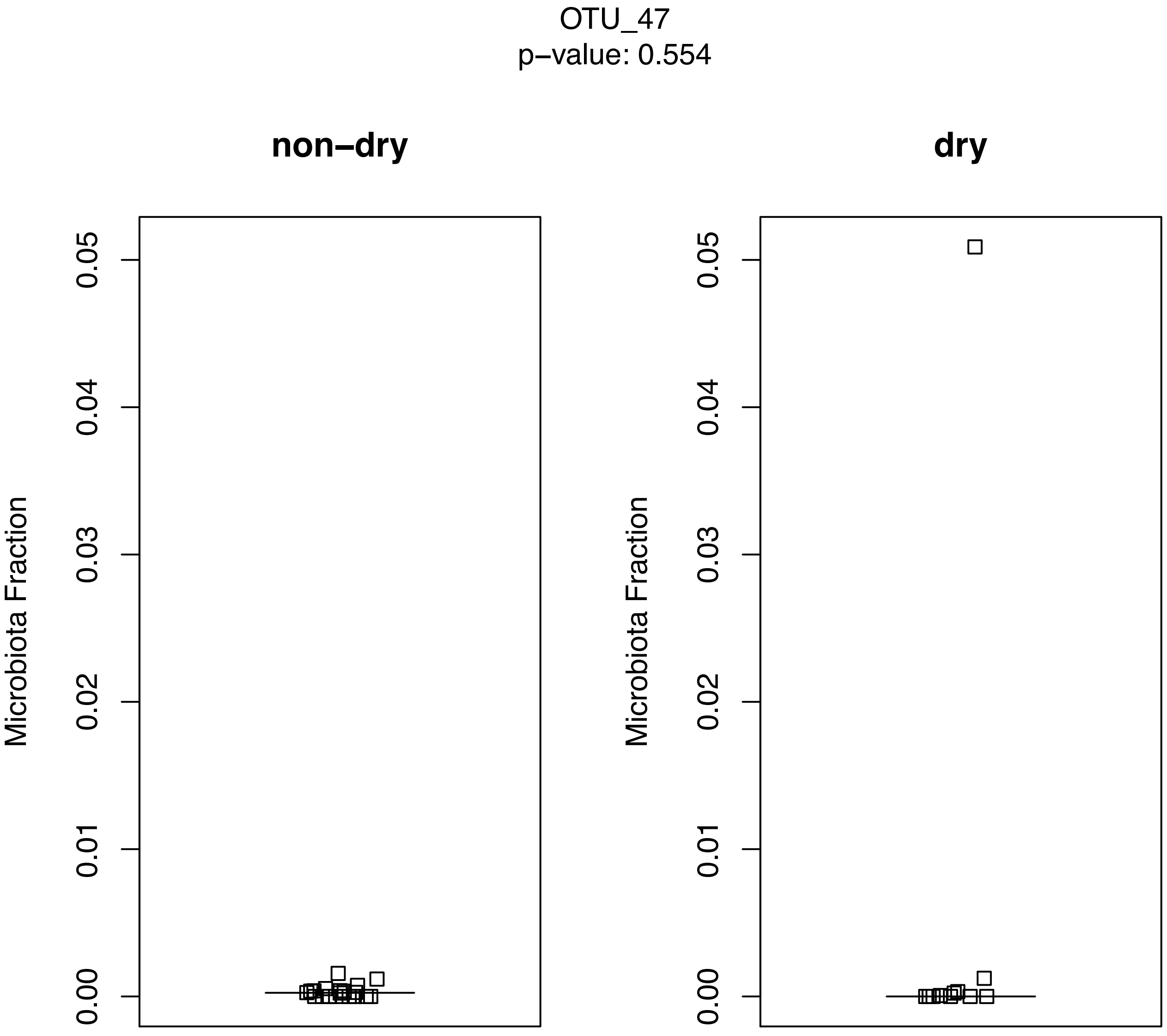

OTU_47
p−value: 0.554
non−dry
dry
Microbiota Fraction
0.00
0.01
0.02
0.03
0.04
0.05

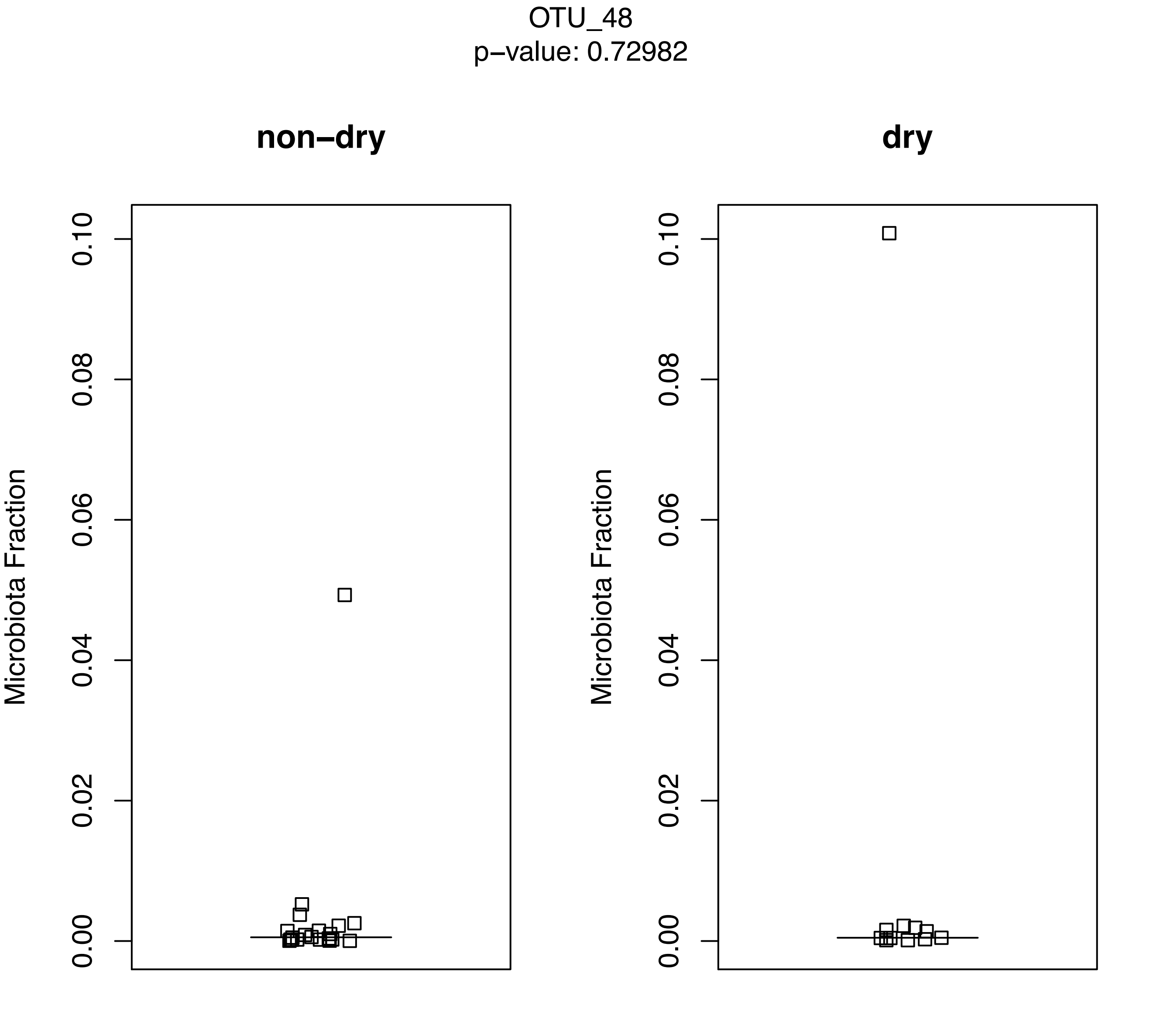
OTU_48
p–value: 0.72982
non–dry
dry
Microbiota Fraction
Microbiota Fraction
0.00
0.02
0.04
0.06
0.08
0.10

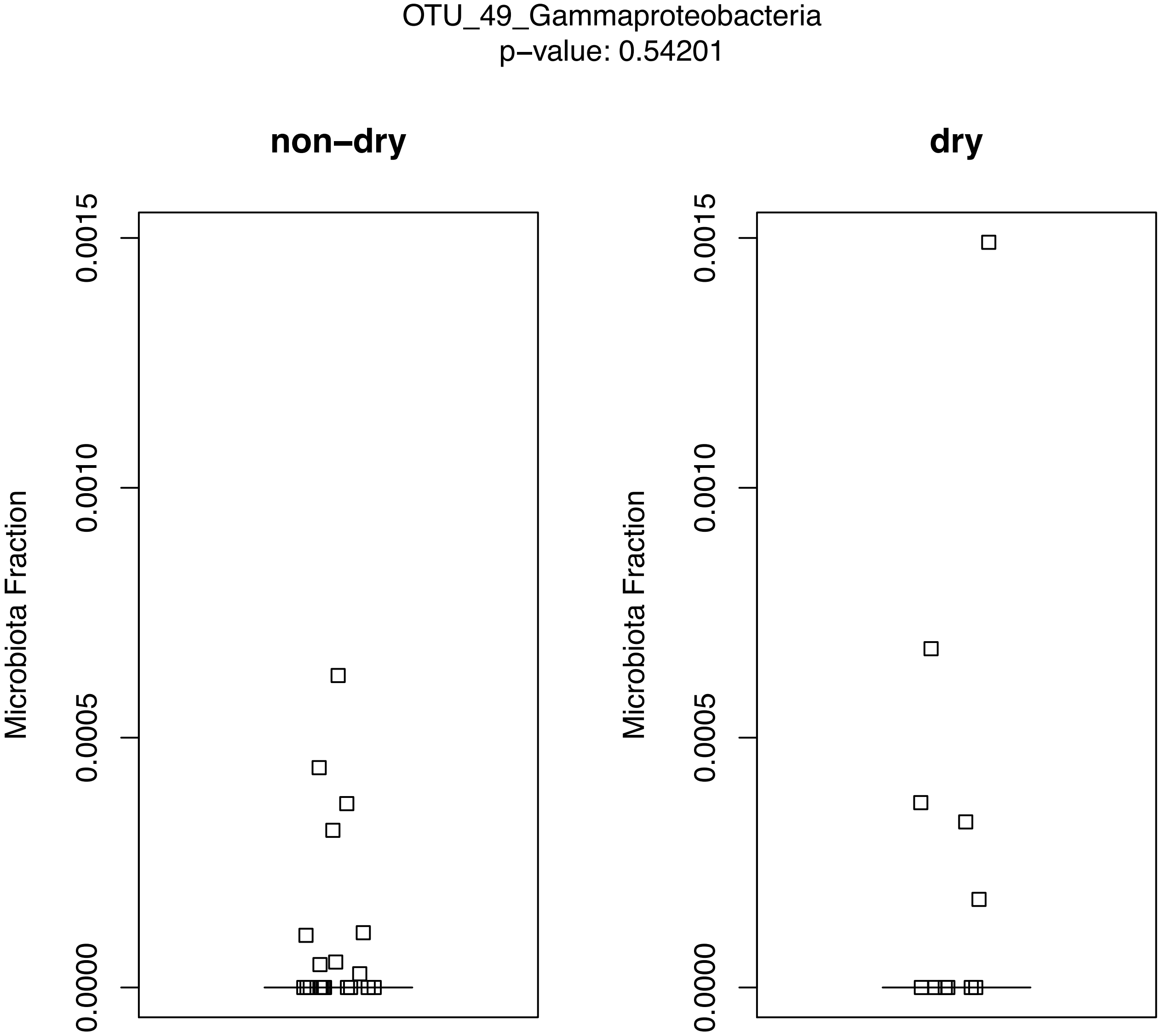

OTU_49_Gammaproteobacteria
p−value: 0.54201
non−dry
dry
Microbiota Fraction
0.0000
0.0005
0.0010
0.0015

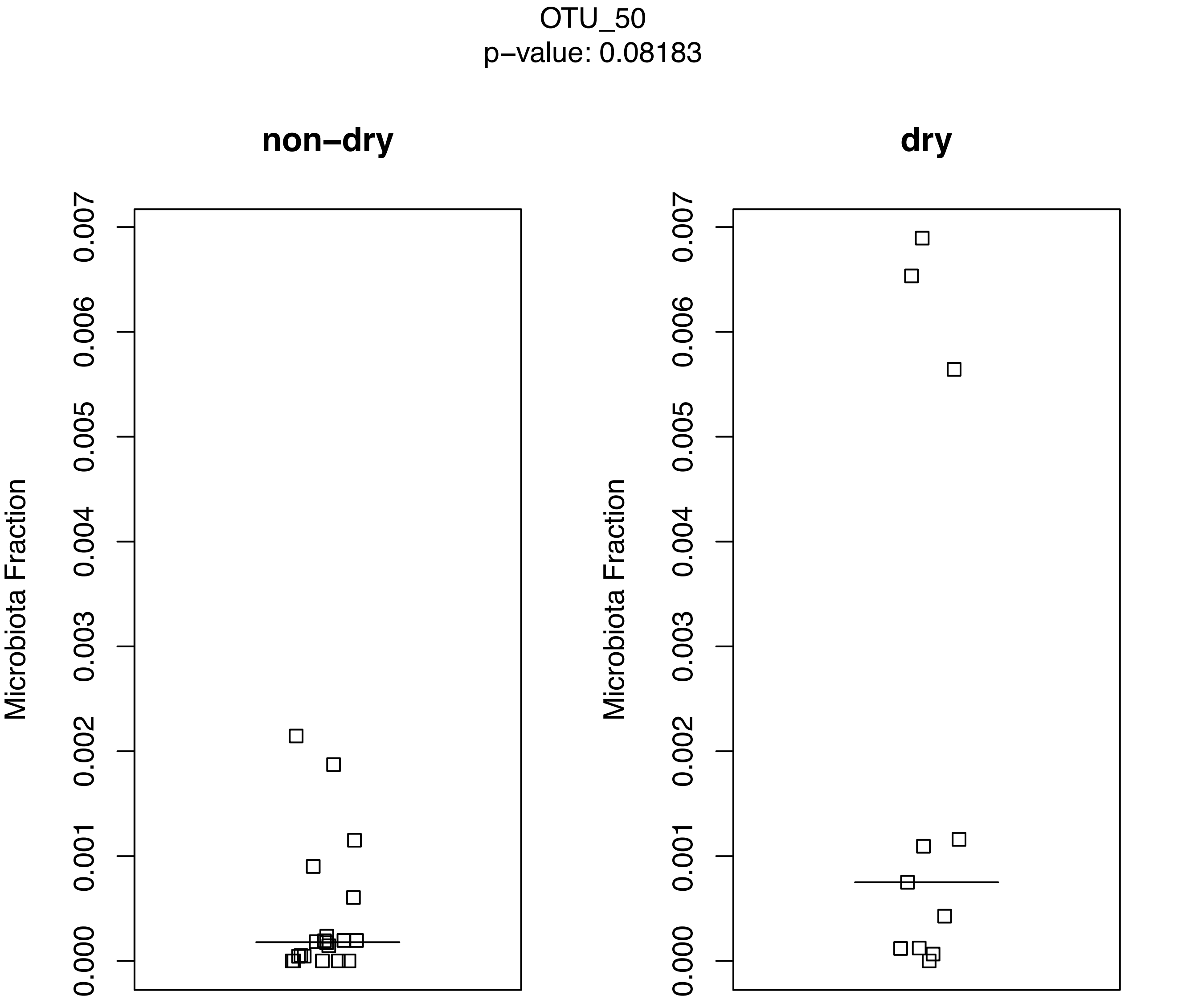
OTU_50
p–value: 0.08183
non–dry
dry
Microbiota Fraction
Microbiota Fraction
0.000
0.001
0.002
0.003
0.004
0.005
0.006
0.007

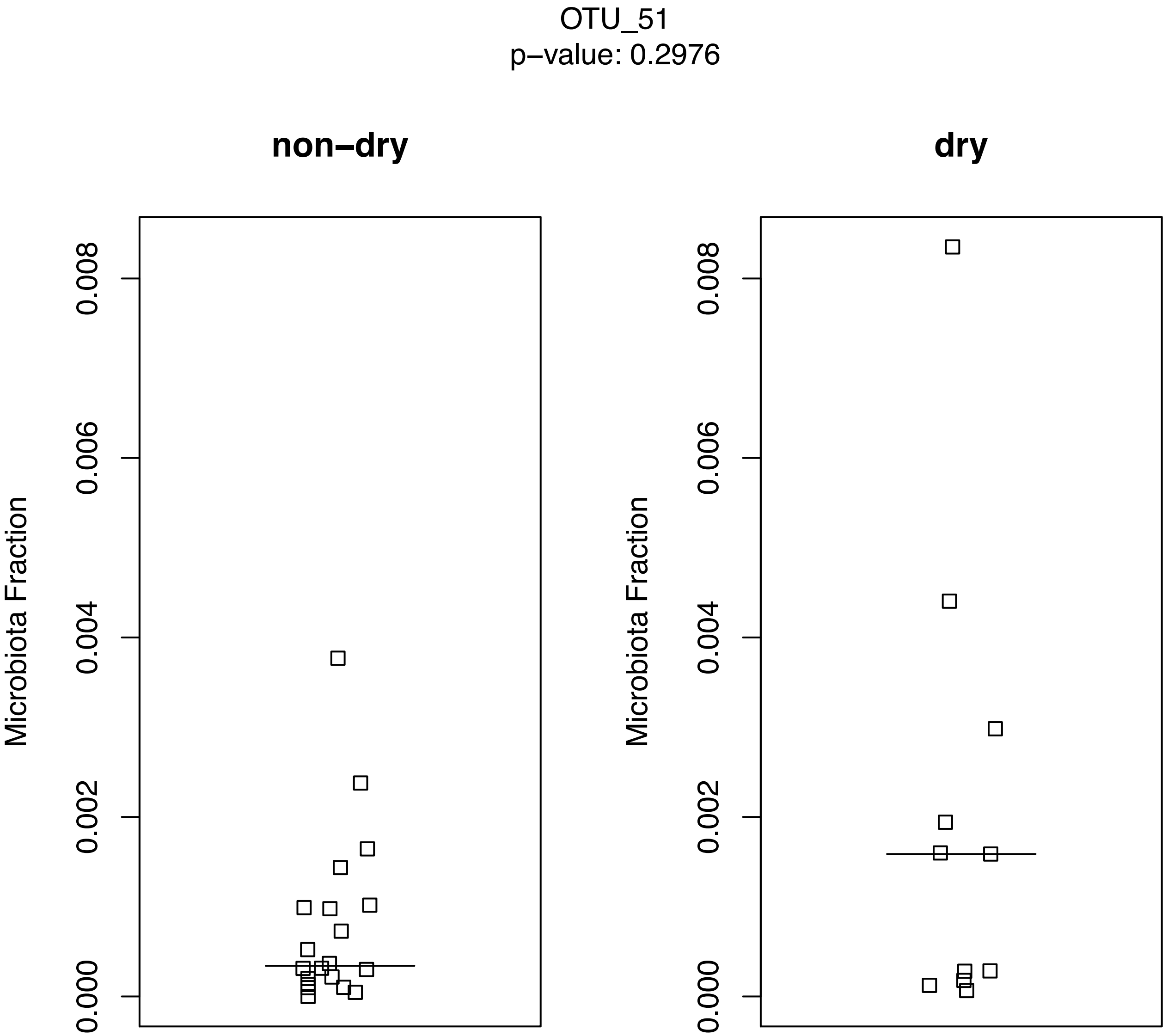

OTU_51
p−value: 0.2976
non−dry
dry
Microbiota Fraction
0.000
0.002
0.004
0.006
0.008

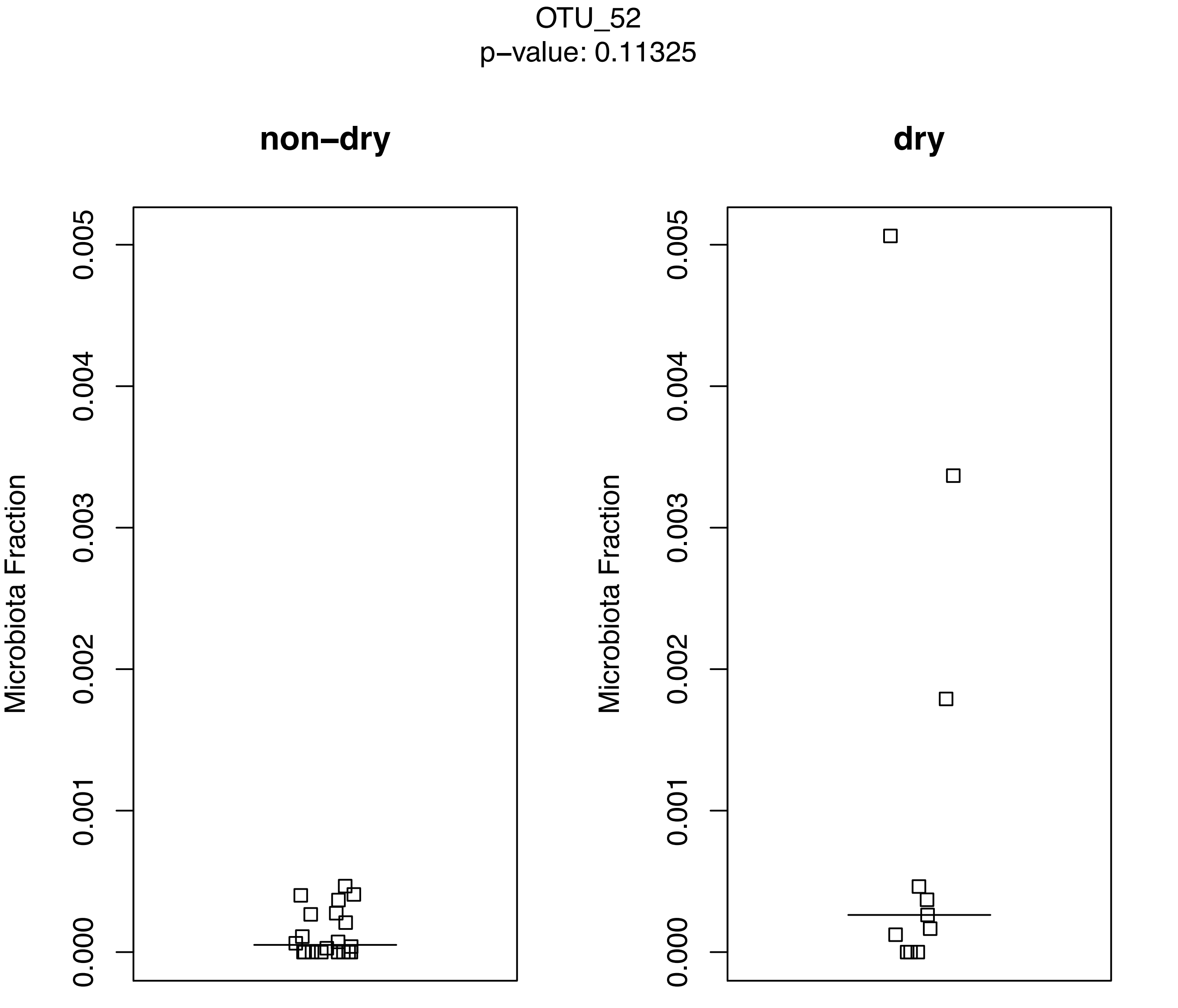
OTU_52
p−value: 0.11325
non−dry
dry
Microbiota Fraction
0.000
0.001
0.002
0.003
0.004
0.005

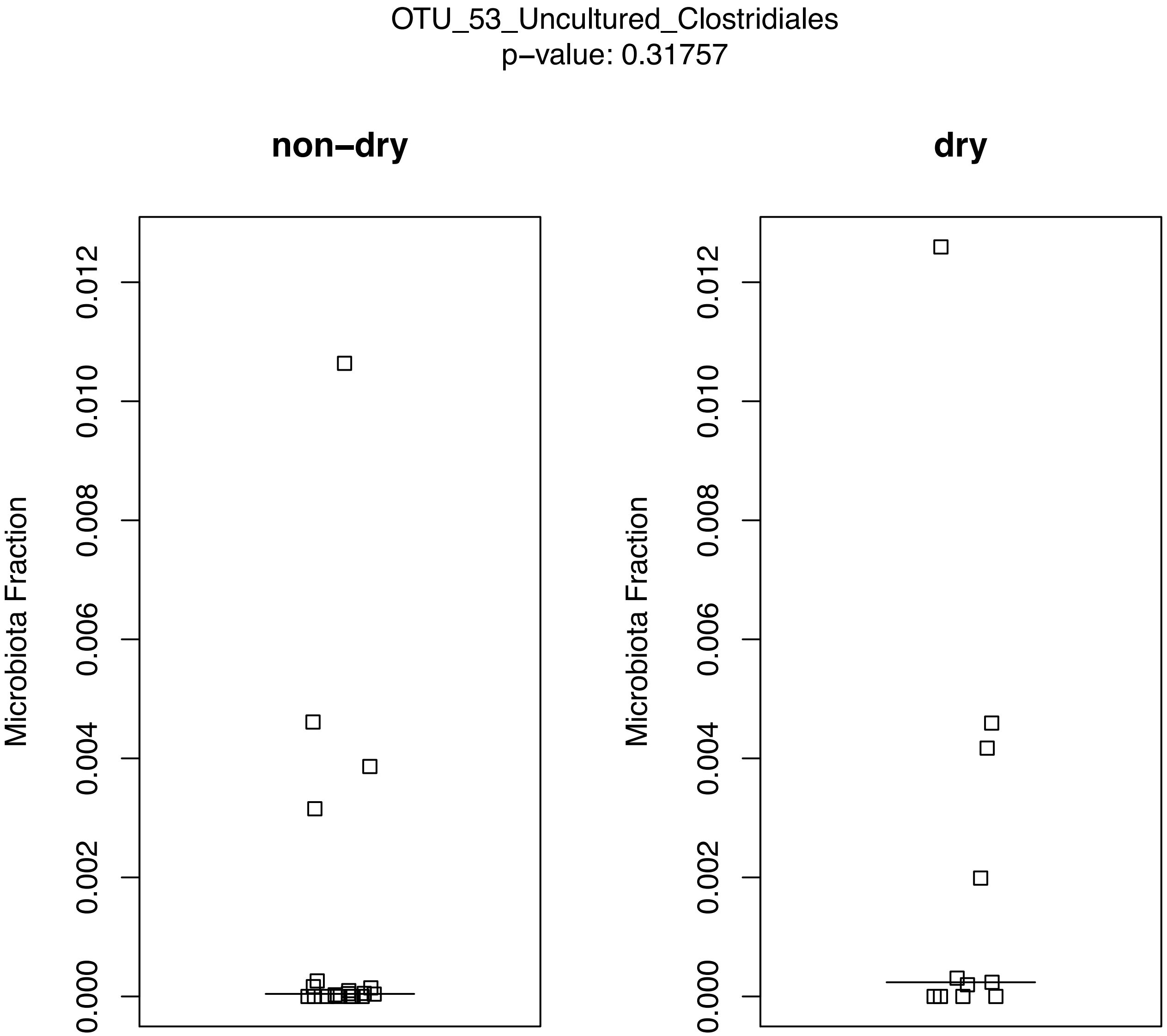

OTU_53_Uncultured_Clostridiales
p−value: 0.31757
non−dry
dry
Microbiota Fraction
Microbiota Fraction
0.000
0.002
0.004
0.006
0.008
0.010
0.012
0.000
0.002
0.004
0.006
0.008
0.010
0.012

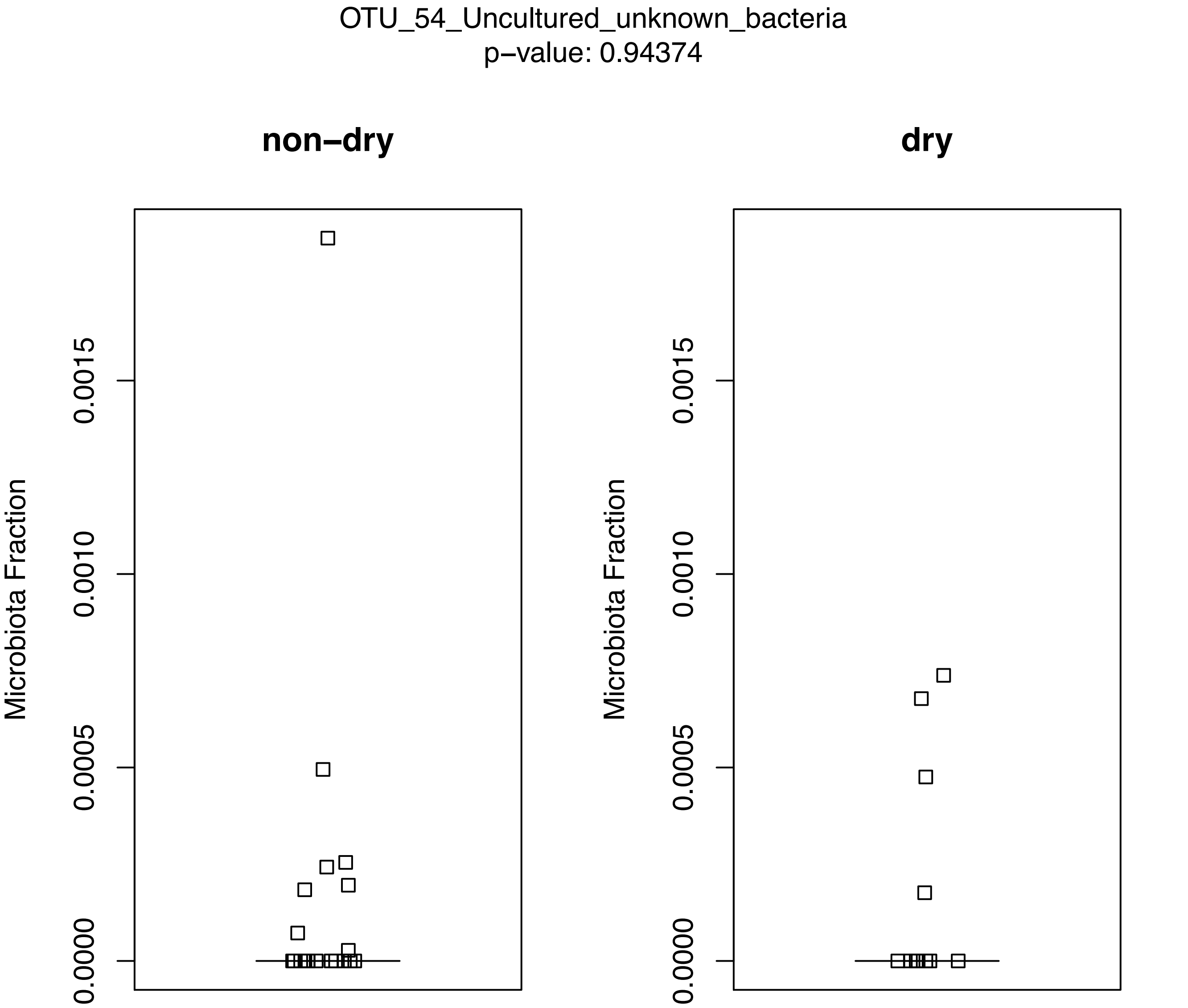
OTU_54_Uncultured_unknown_bacteria
p−value: 0.94374
non−dry
dry
Microbiota Fraction
0.0000
0.0005
0.0010
0.0015
Microbiota Fraction
0.0000
0.0005
0.0010
0.0015

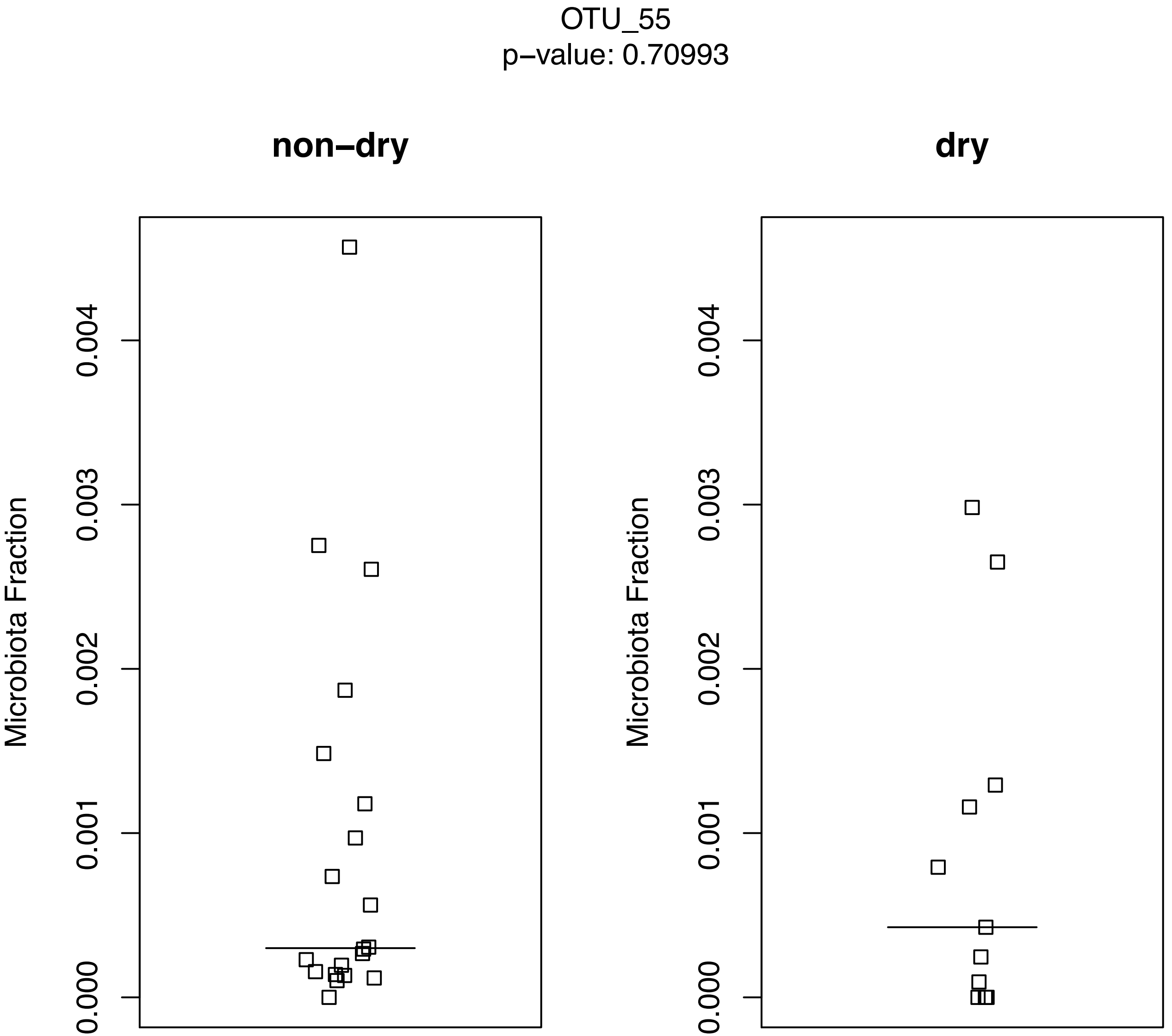
OTU_55
p−value: 0.70993
non−dry
dry
Microbiota Fraction
0.000
0.001
0.002
0.003
0.004
Microbiota Fraction
0.000
0.001
0.002
0.003
0.004

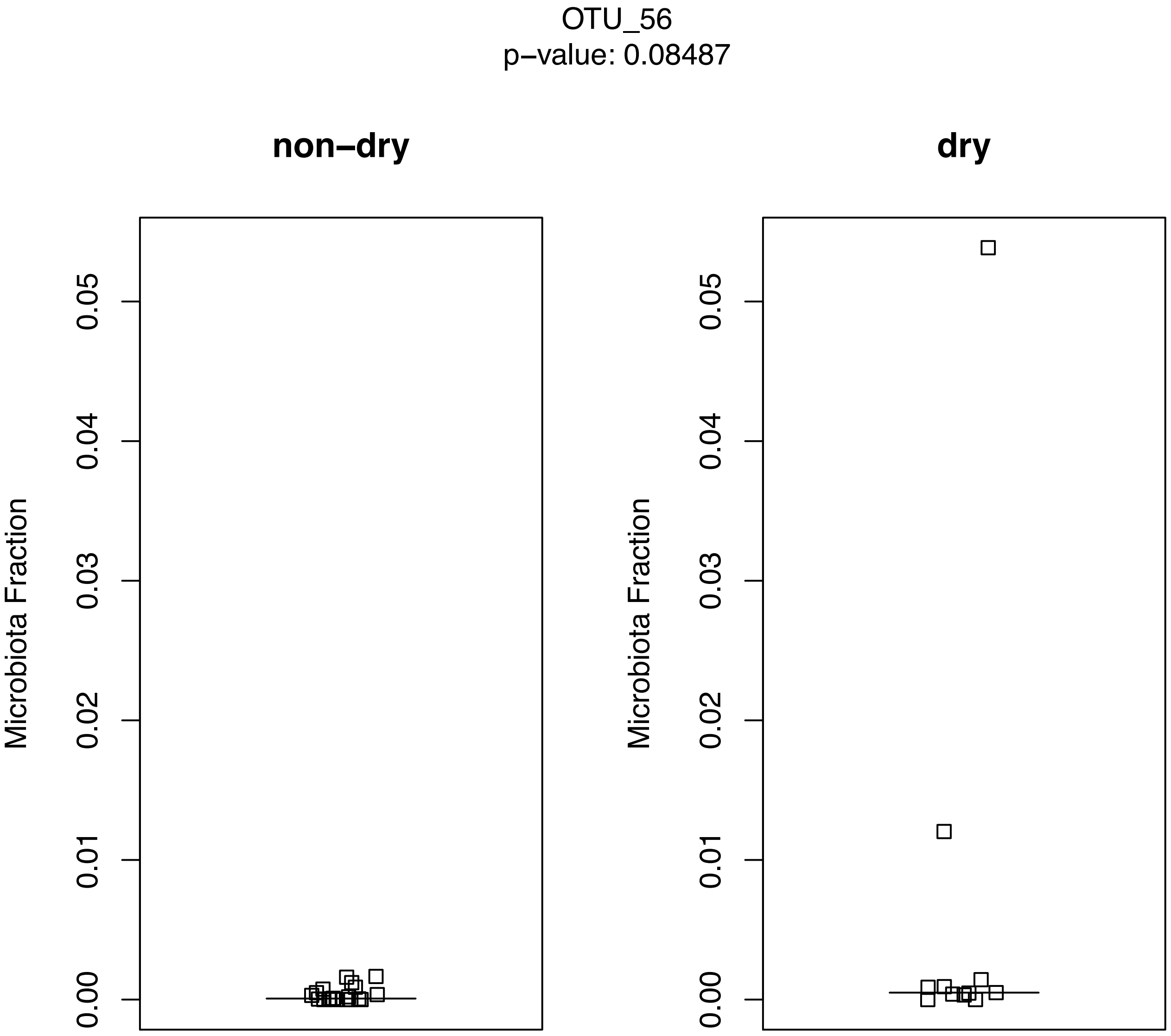

OTU_56
p−value: 0.08487
non−dry
dry
Microbiota Fraction
0.00
0.01
0.02
0.03
0.04
0.05

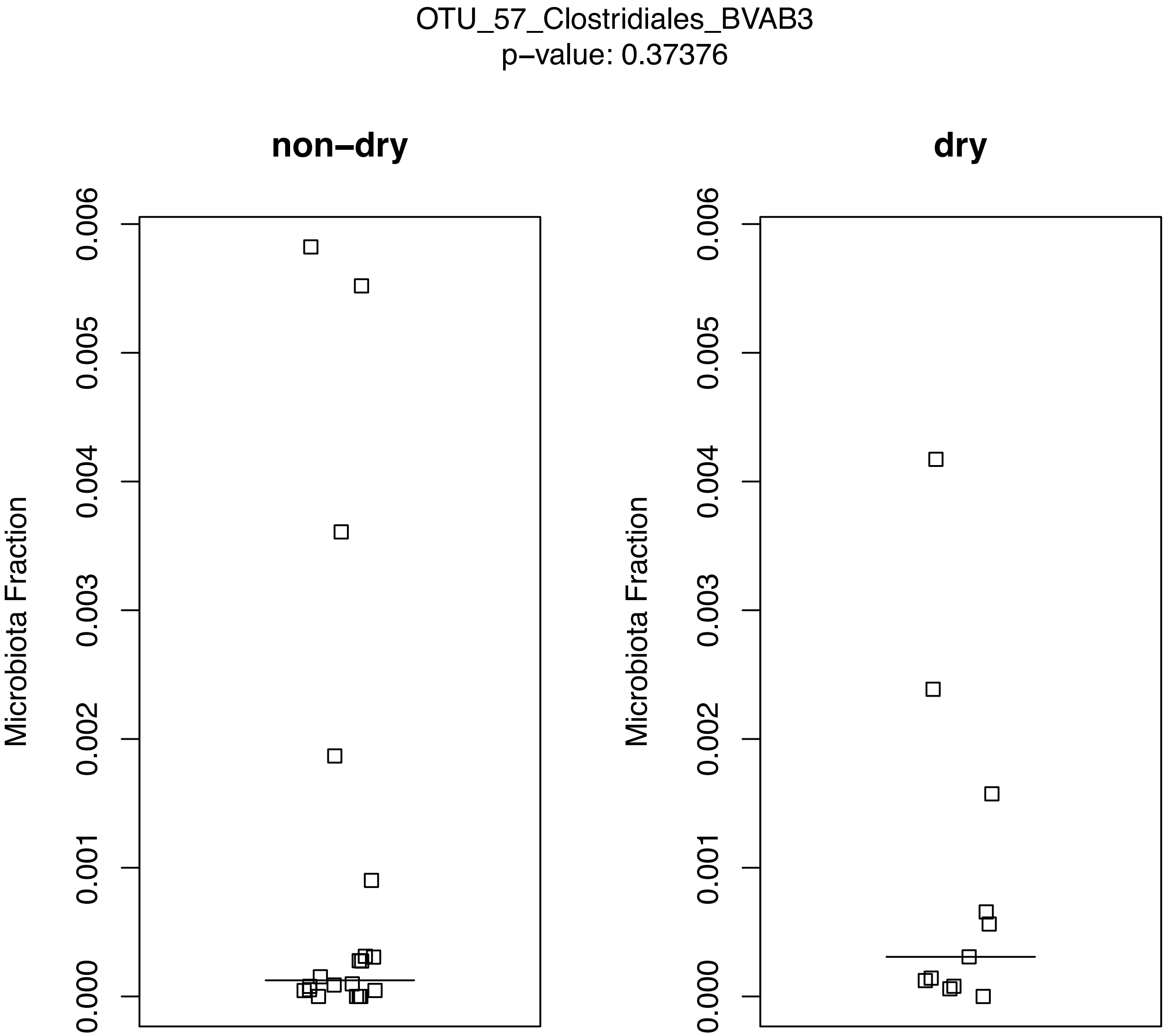
OTU_57_Clostridiales_BVAB3
p–value: 0.37376
non–dry
dry
Microbiota Fraction
Microbiota Fraction
0.000
0.001
0.002
0.003
0.004
0.005
0.006

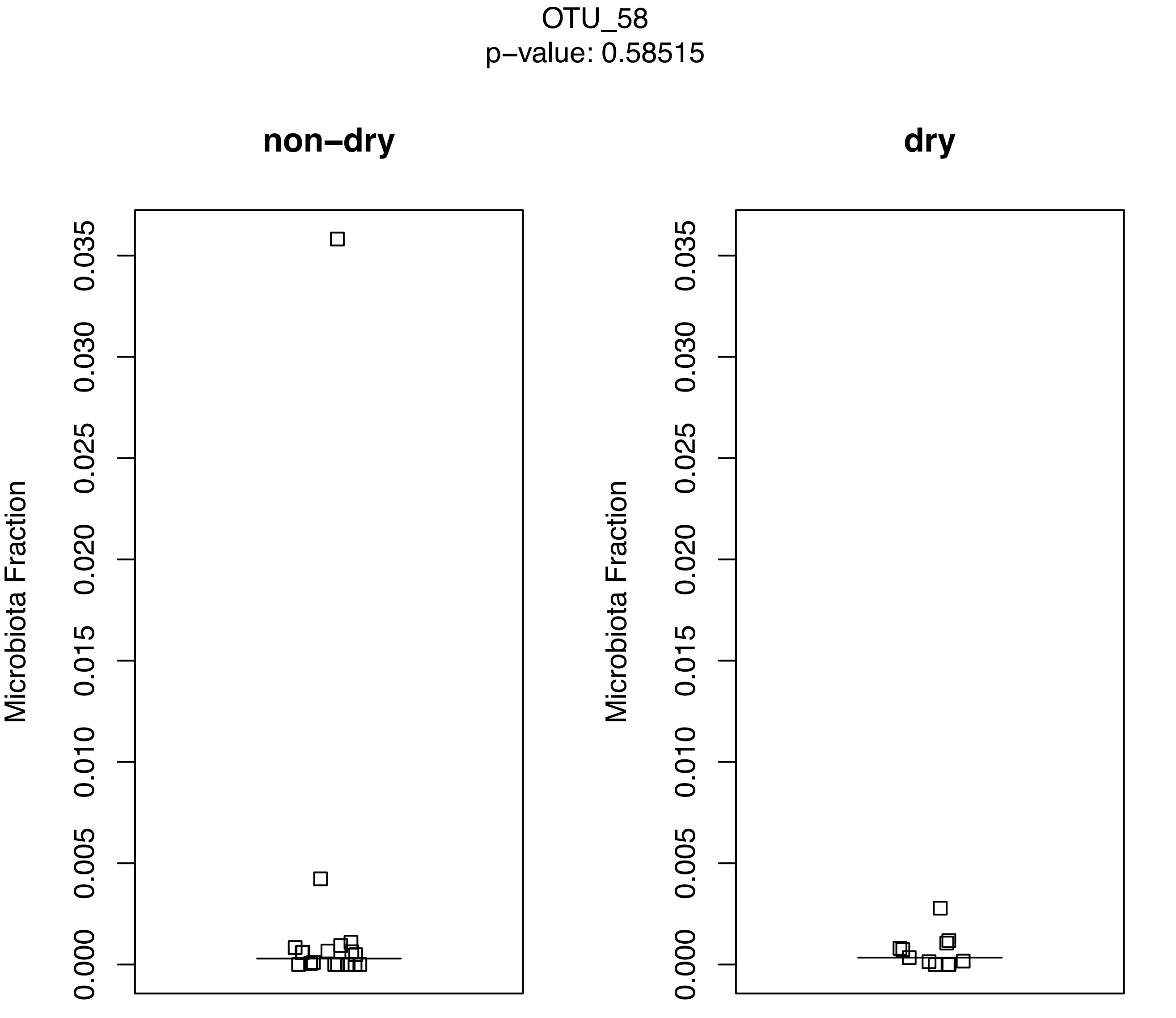

OTU_58
p−value: 0.58515
non−dry
dry
Microbiota Fraction
0.000
0.005
0.010
0.015
0.020
0.025
0.030
0.035

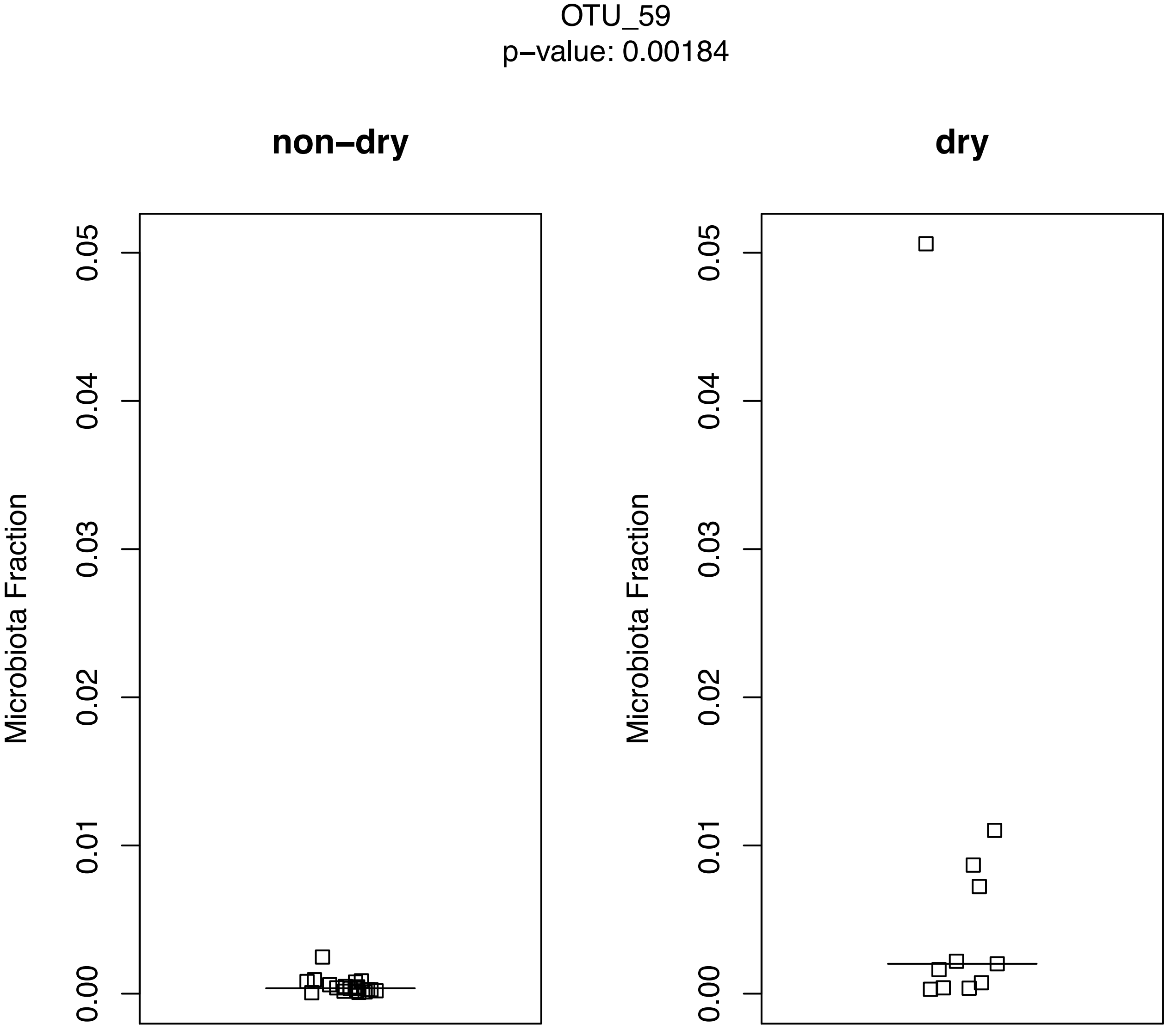

OTU_59
p−value: 0.00184
non−dry
dry
Microbiota Fraction
0.00
0.01
0.02
0.03
0.04
0.05
Microbiota Fraction
0.00
0.01
0.02
0.03
0.04
0.05

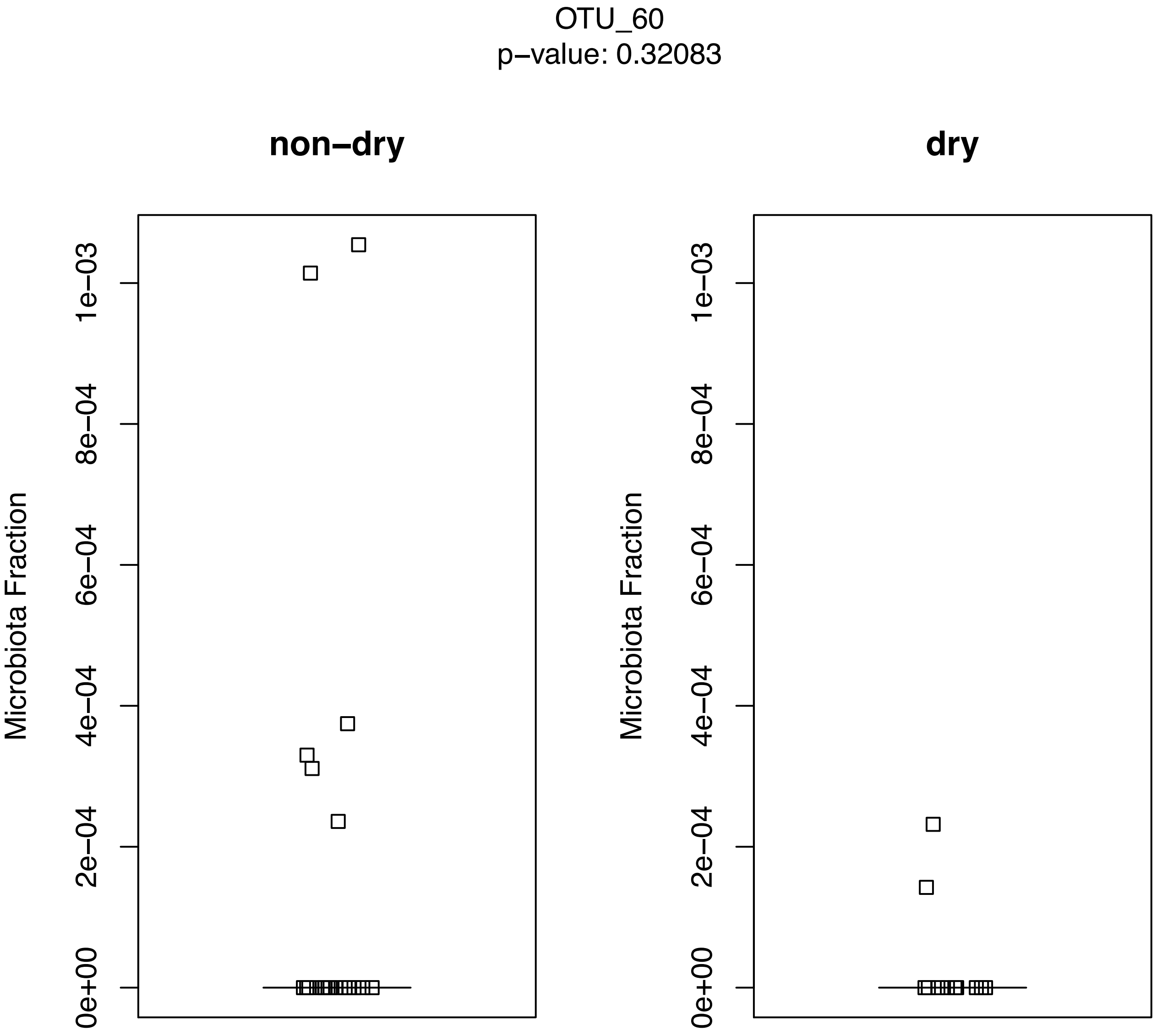

OTU_60
p–value: 0.32083
non–dry
dry
Microbiota Fraction
0e+00
2e–04
4e–04
6e–04
8e–04
1e–03

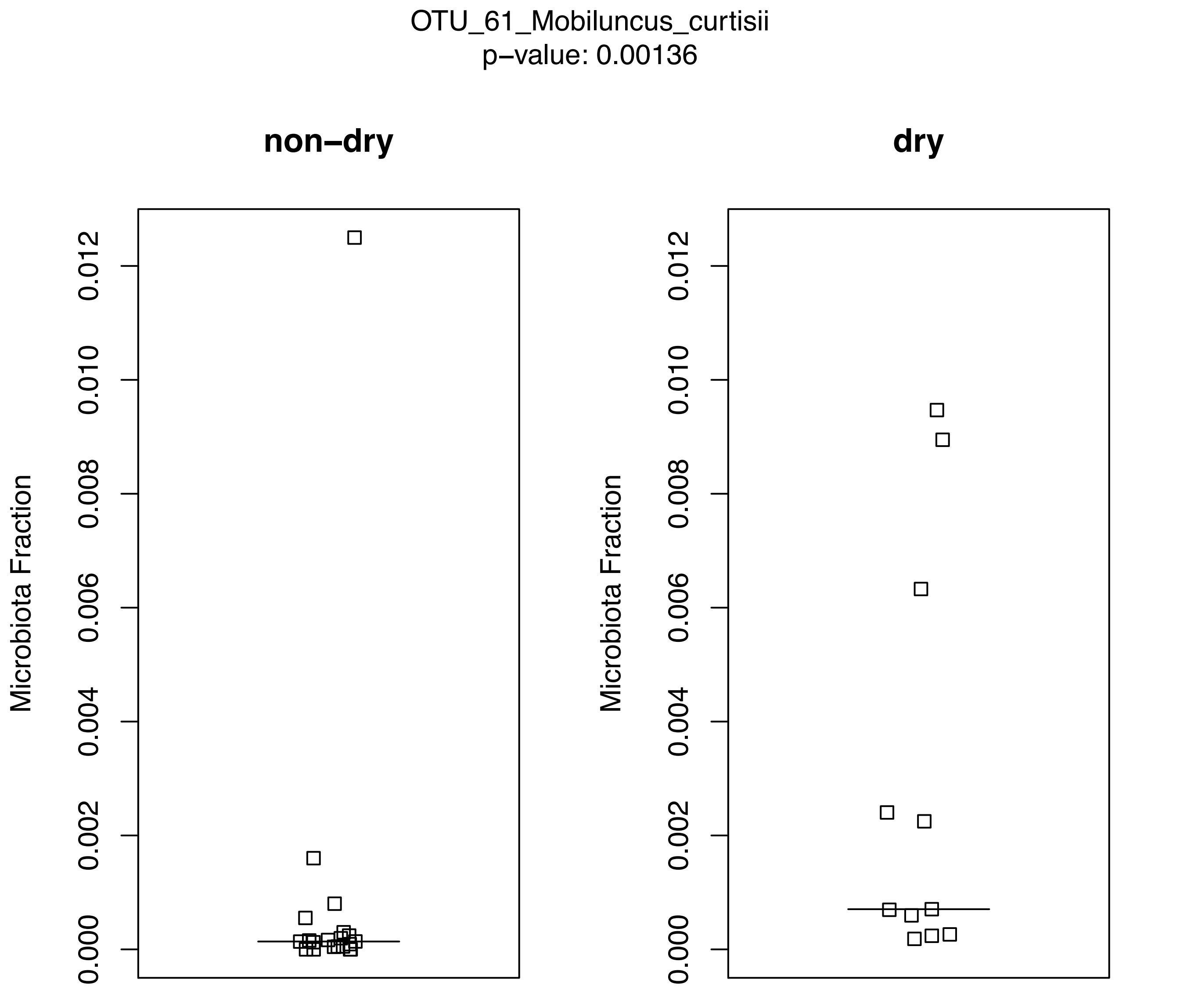

OTU_61_Mobiluncus_curtisii
p−value: 0.00136
non−dry
dry
Microbiota Fraction
0.000
0.002
0.004
0.006
0.008
0.010
0.012
Microbiota Fraction
0.000
0.002
0.004
0.006
0.008
0.010
0.012

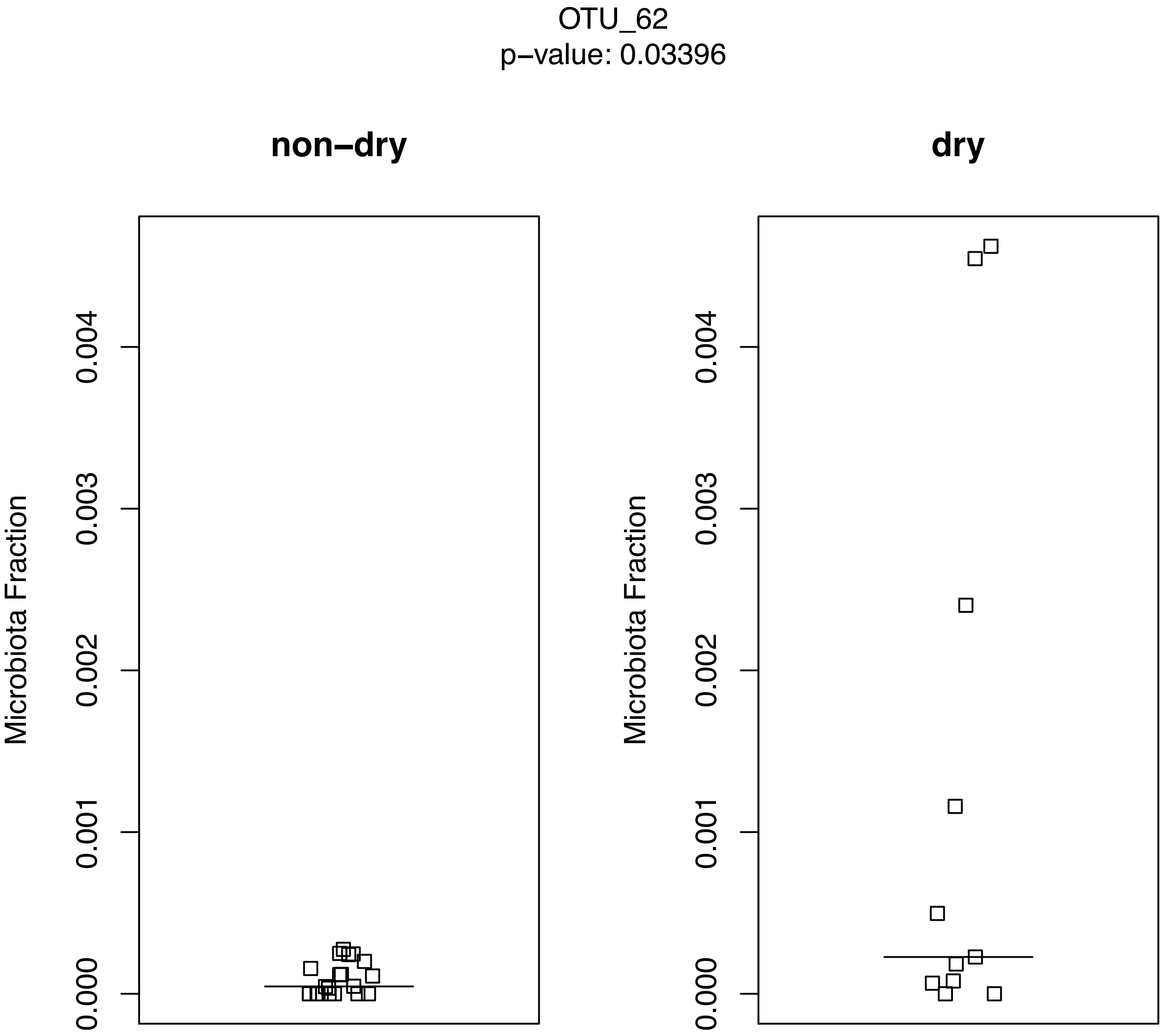
OTU_62
p−value: 0.03396
non−dry
dry
Microbiota Fraction
0.000
0.001
0.002
0.003
0.004
Microbiota Fraction
0.000
0.001
0.002
0.003
0.004

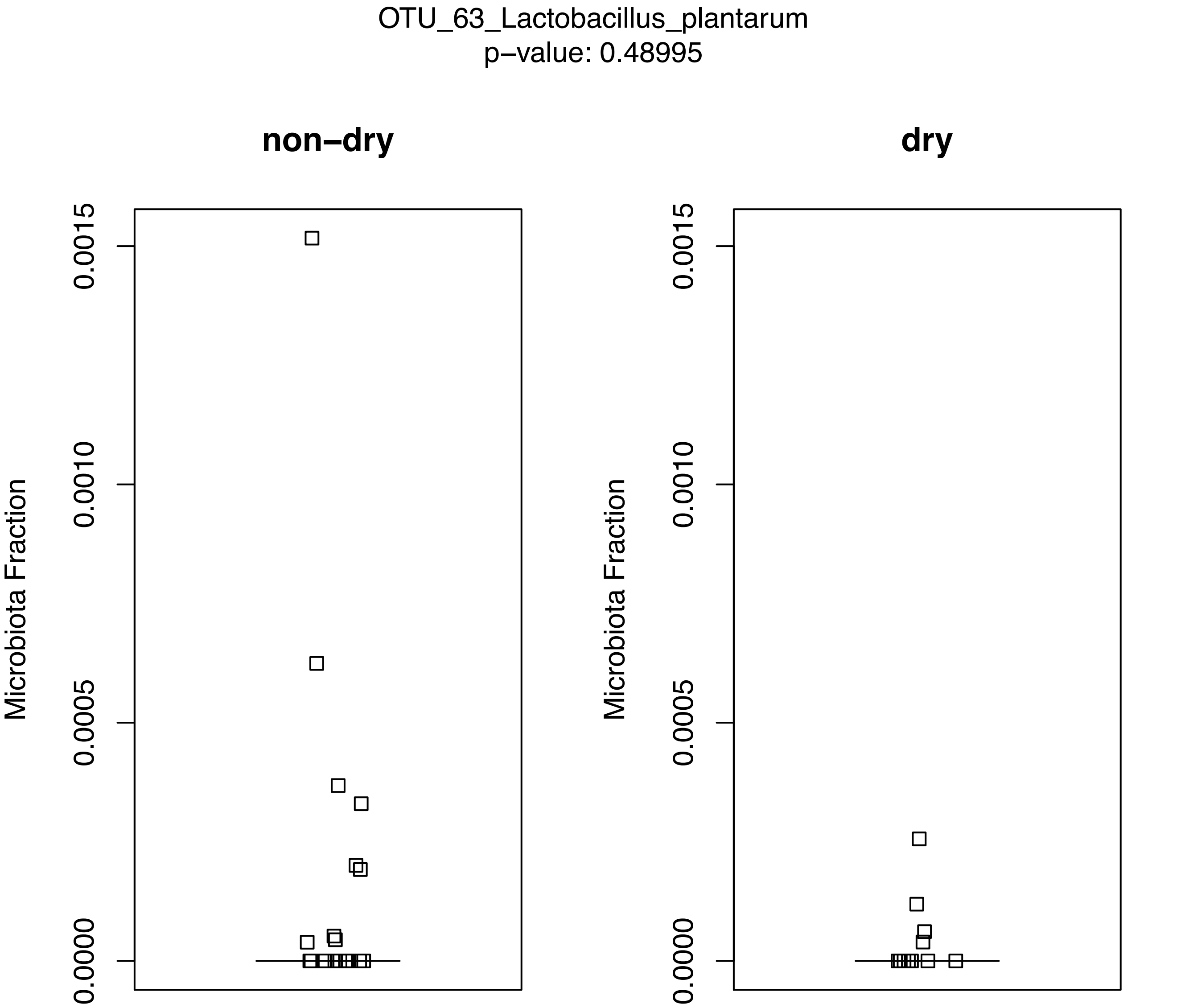

OTU_63_Lactobacillus_plantarum
p–value: 0.48995
non–dry
dry
Microbiota Fraction
0.0000
0.0005
0.0010
0.0015
Microbiota Fraction
0.0000
0.0005
0.0010
0.0015

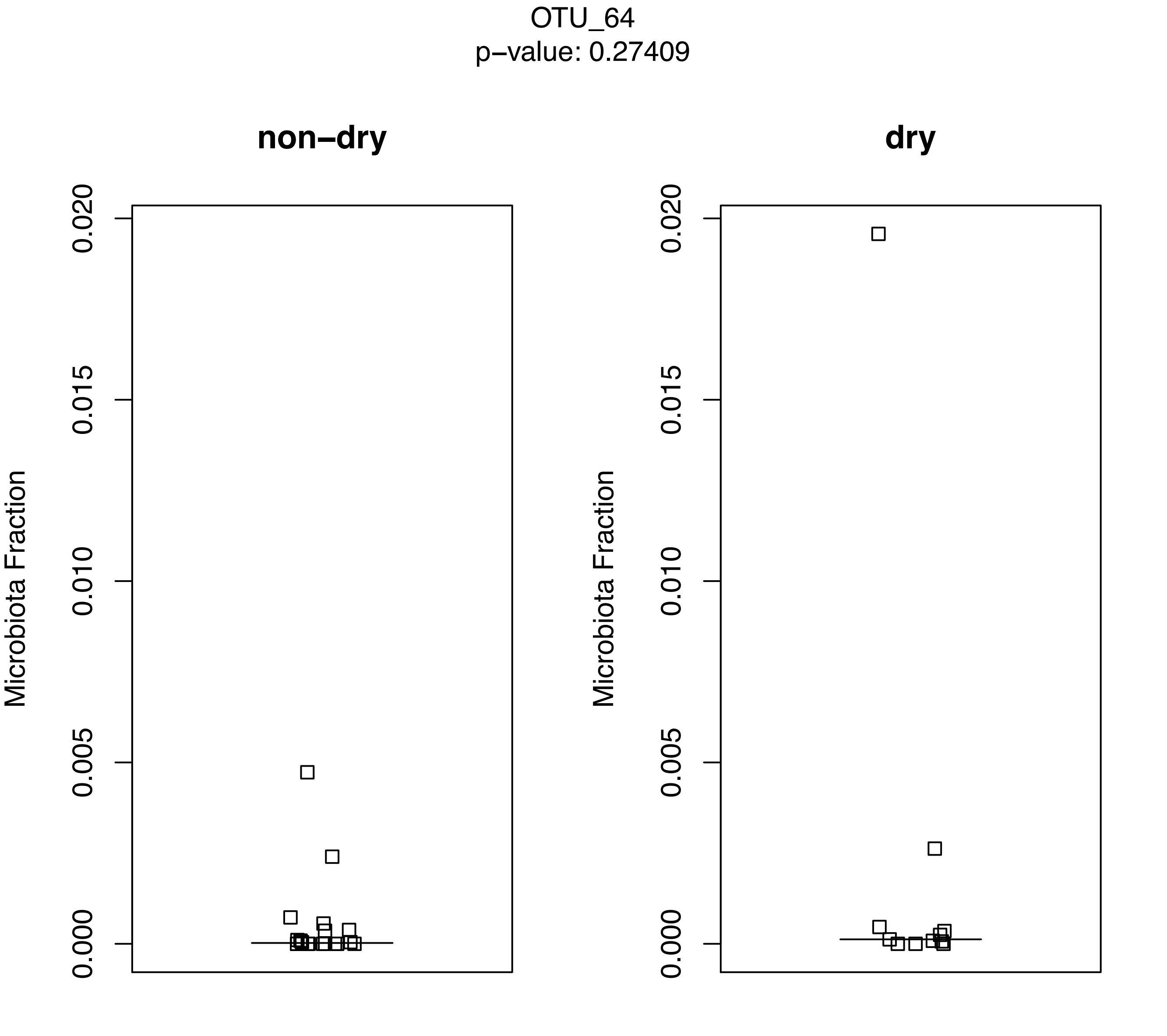
OTU_64
p−value: 0.27409
non−dry
dry
Microbiota Fraction
0.000
0.005
0.010
0.015
0.020

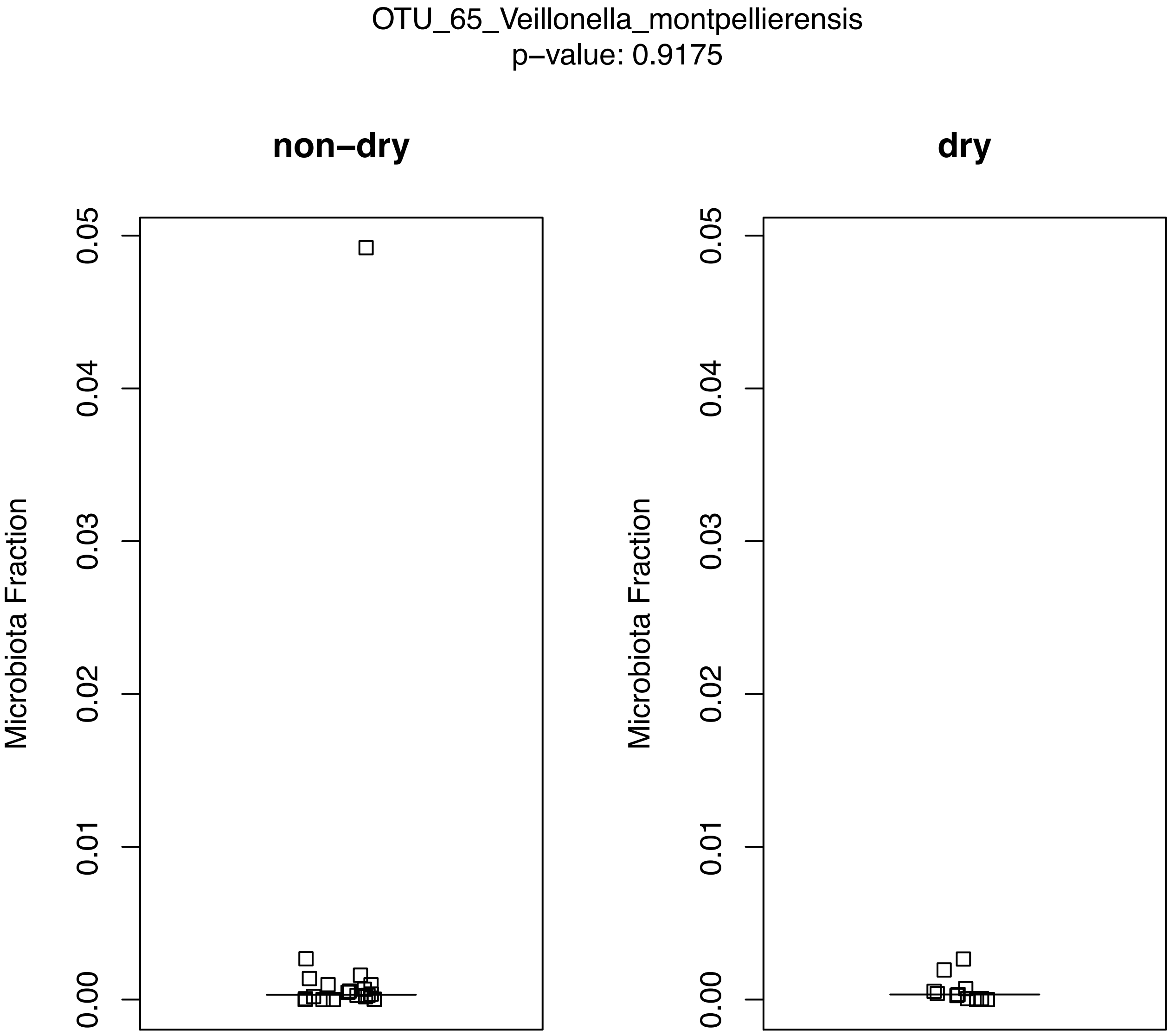
OTU_65_Veillonella_montpellierensis
p−value: 0.9175
non−dry
dry
Microbiota Fraction
0.00
0.01
0.02
0.03
0.04
0.05

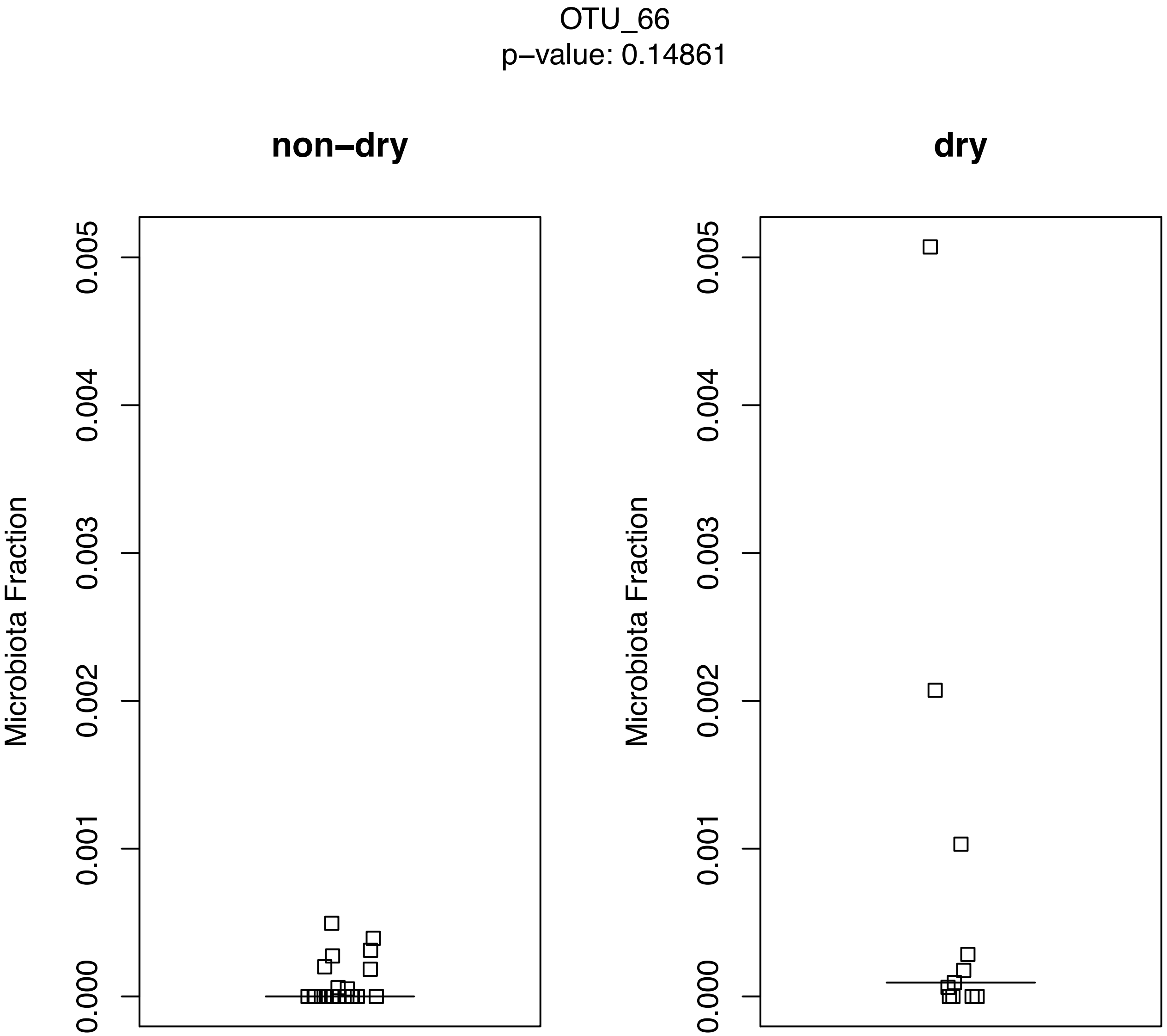
OTU_66
p−value: 0.14861
non−dry
dry
Microbiota Fraction
0.000
0.001
0.002
0.003
0.004
0.005
Microbiota Fraction
0.000
0.001
0.002
0.003
0.004
0.005

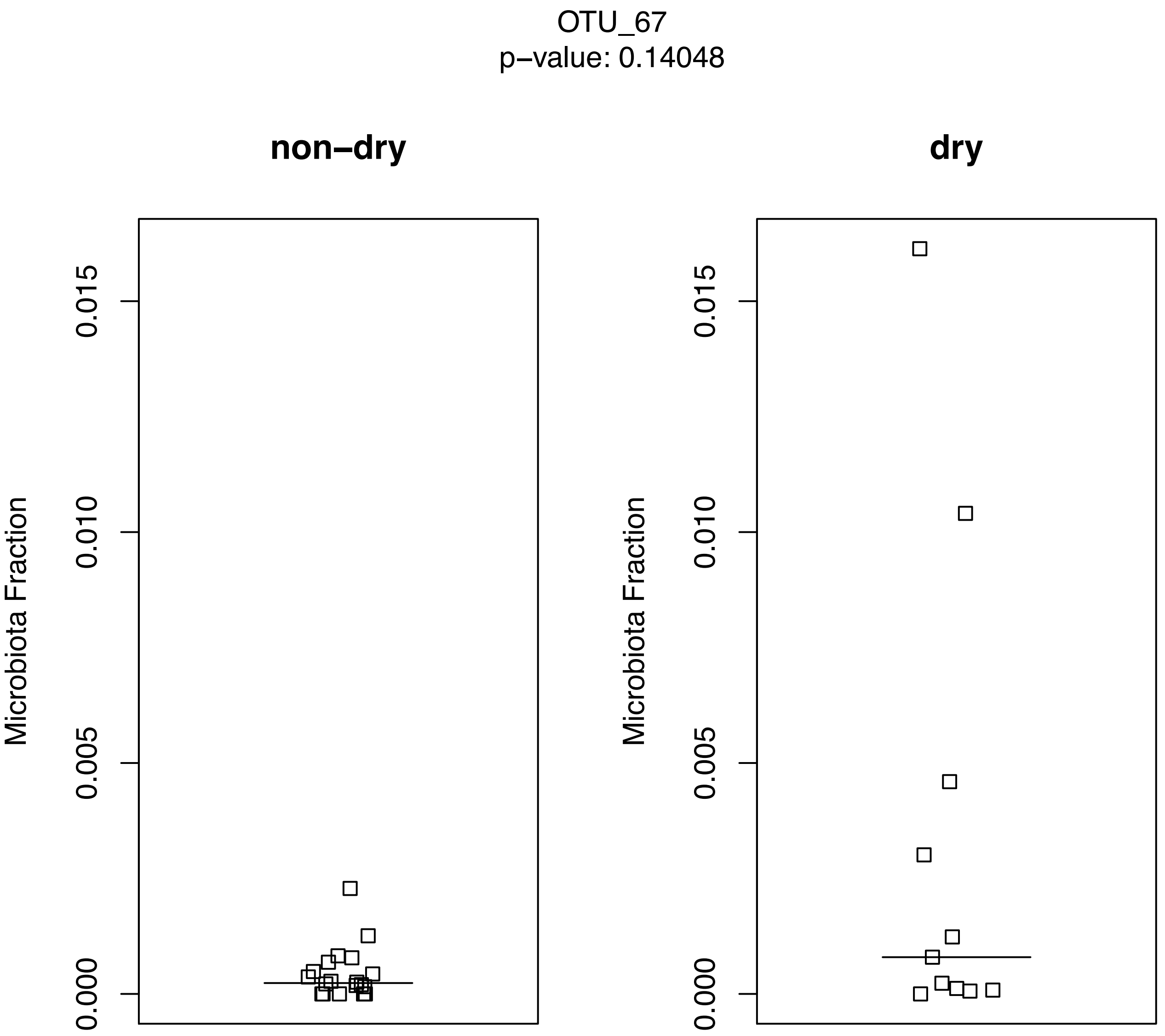
OTU_67
p−value: 0.14048
non−dry
dry
Microbiota Fraction
0.000
0.005
0.010
0.015
Microbiota Fraction
0.000
0.005
0.010
0.015

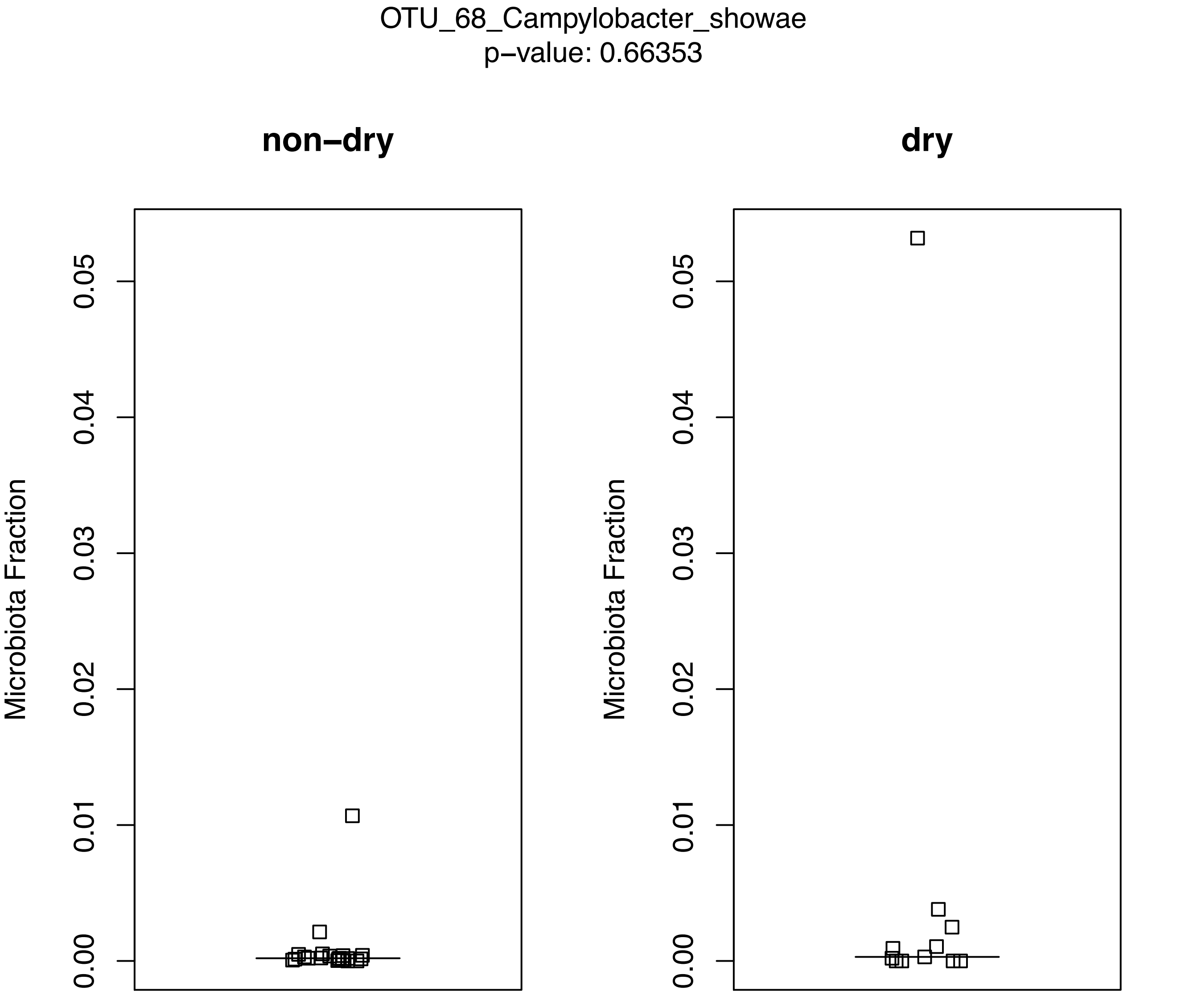
OTU_68_Campylobacter_showae
p–value: 0.66353
non–dry
dry
Microbiota Fraction
0.00
0.01
0.02
0.03
0.04
0.05
Microbiota Fraction
0.00
0.01
0.02
0.03
0.04
0.05

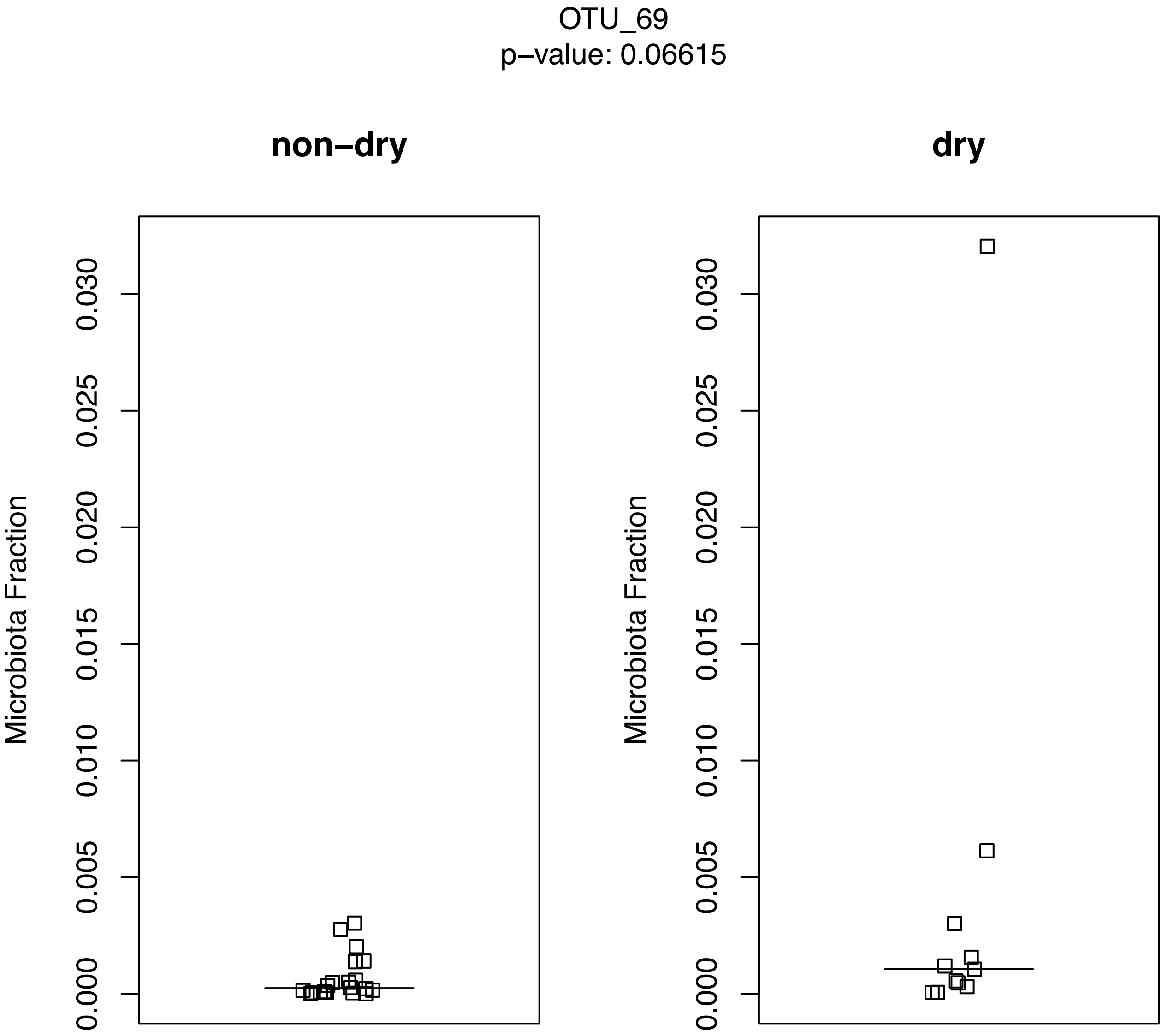

OTU_69
p−value: 0.06615
non−dry
dry
Microbiota Fraction
Microbiota Fraction
0.000
0.005
0.010
0.015
0.020
0.025
0.030
0.000
0.005
0.010
0.015
0.020
0.025
0.030

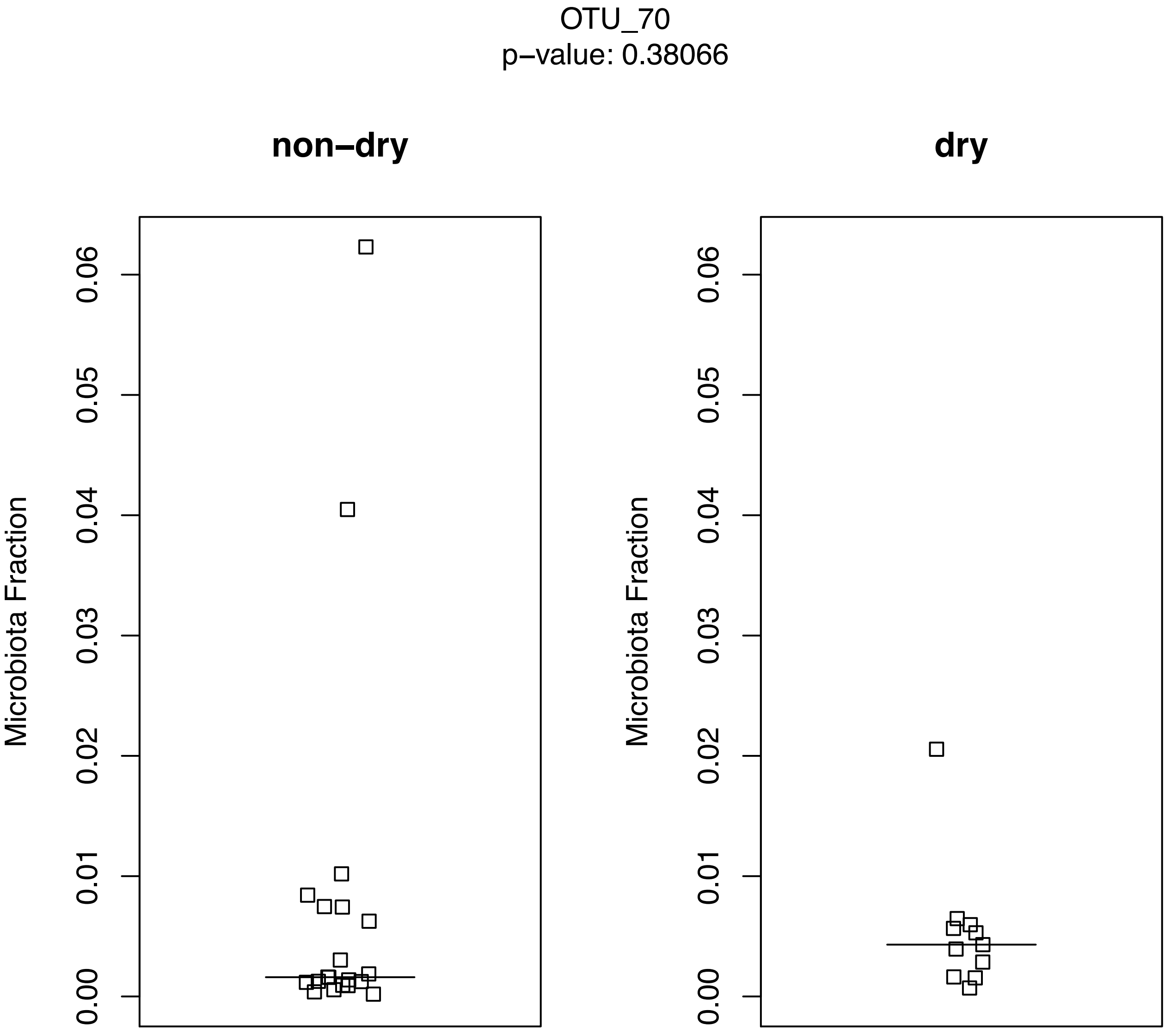

OTU_70
p–value: 0.38066
non–dry
dry
Microbiota Fraction
0.00
0.01
0.02
0.03
0.04
0.05
0.06

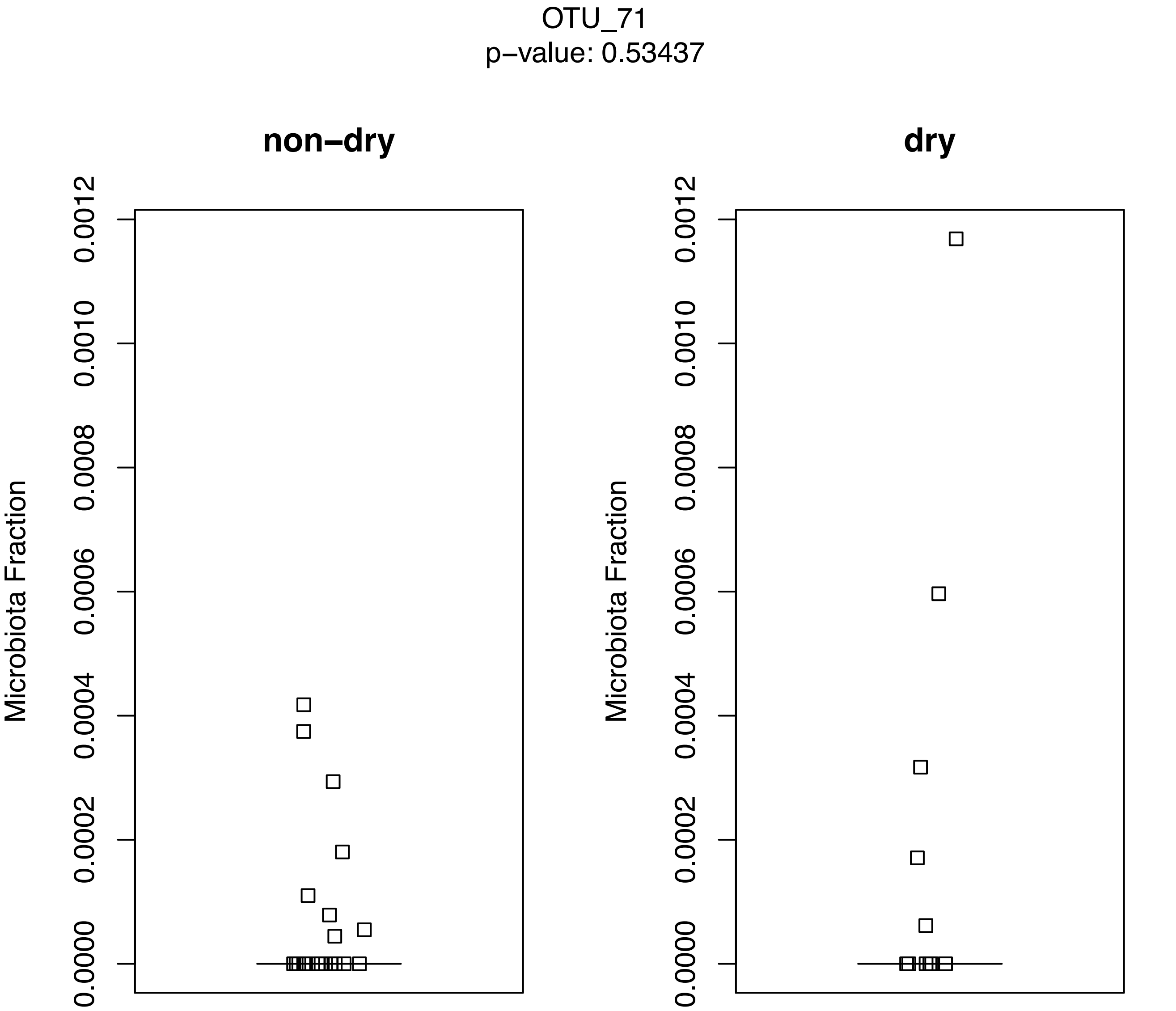
OTU_71
p−value: 0.53437
non−dry
dry
Microbiota Fraction
0.0000
0.0002
0.0004
0.0006
0.0008
0.0010
0.0012

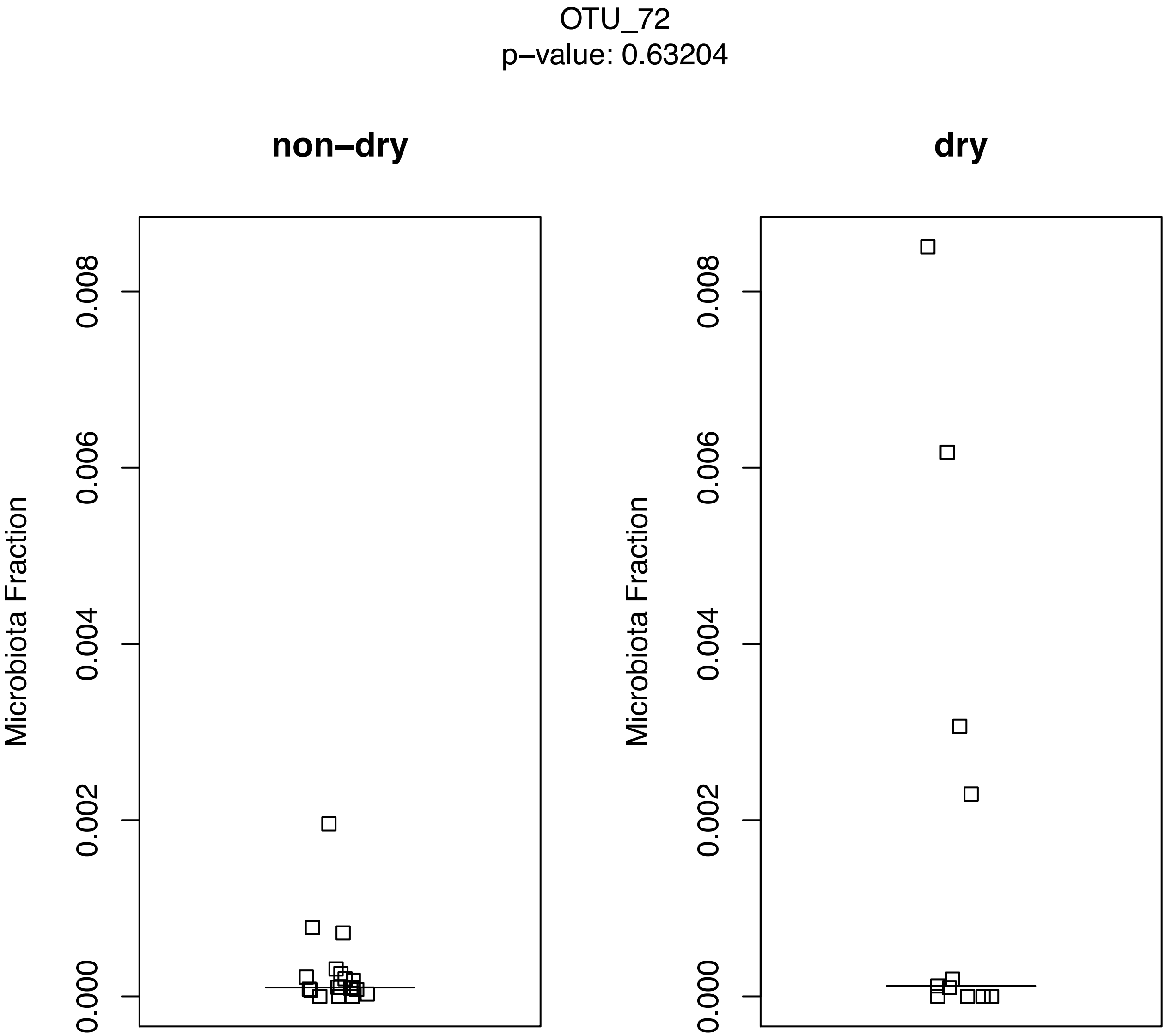
OTU_72
p−value: 0.63204
non−dry
dry
Microbiota Fraction
Microbiota Fraction
0.000
0.002
0.004
0.006
0.008

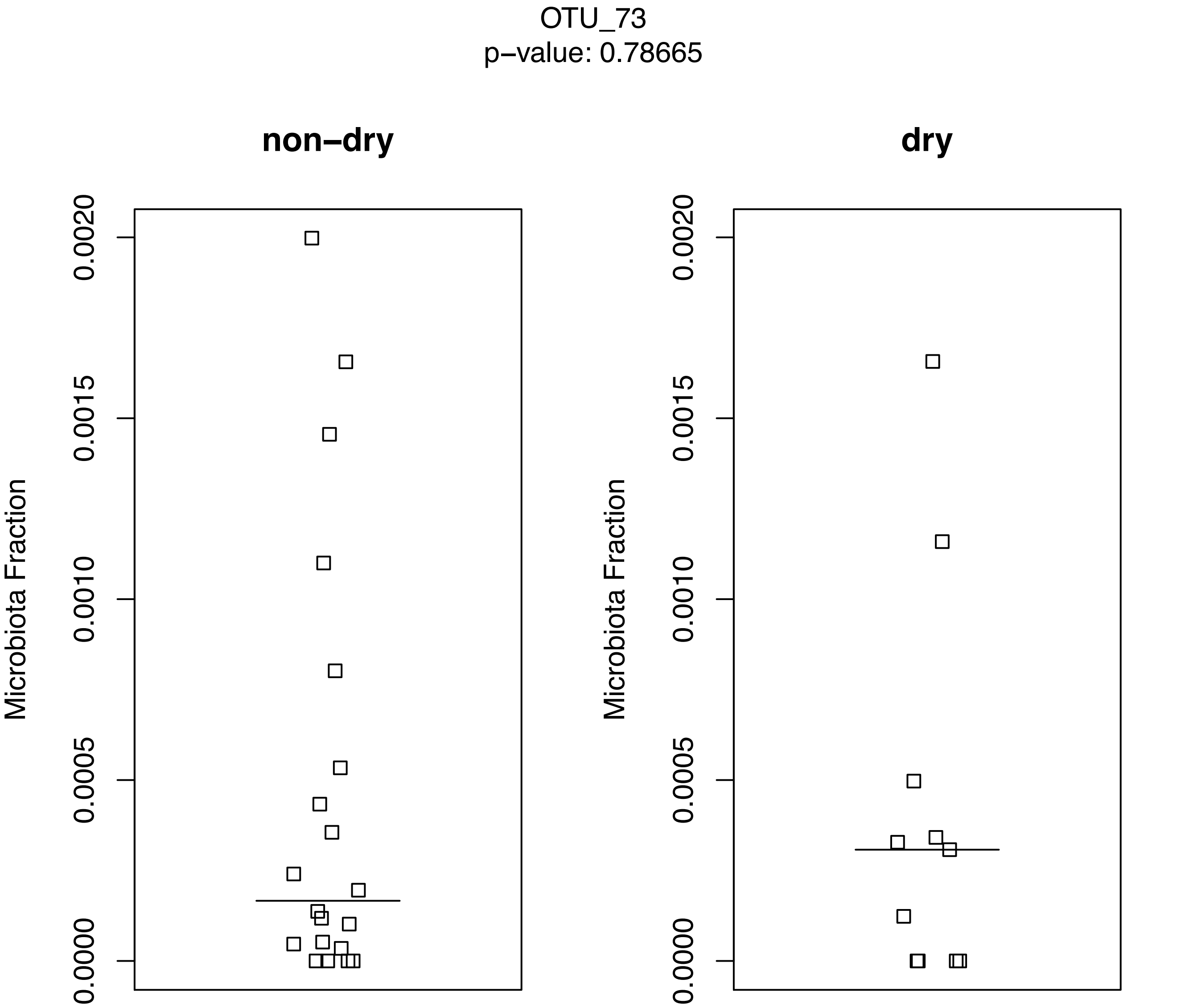
OTU_73
p−value: 0.78665
non−dry
dry
Microbiota Fraction
0.0000
0.0005
0.0010
0.0015
0.0020
Microbiota Fraction
0.0000
0.0005
0.0010
0.0015
0.0020

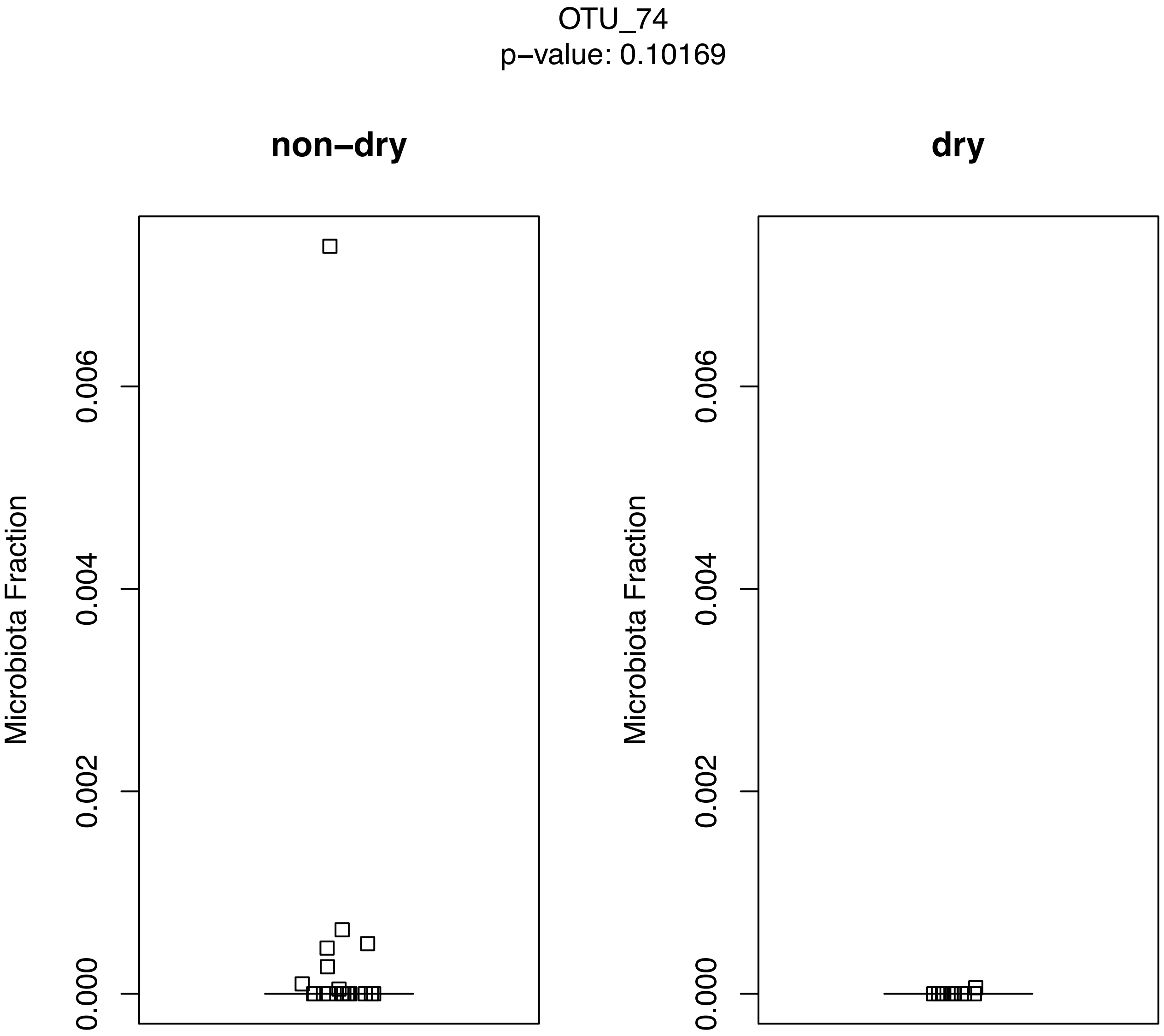
OTU_74
p−value: 0.10169
non−dry
dry
Microbiota Fraction
0.000
0.002
0.004
0.006
Microbiota Fraction
0.000
0.002
0.004
0.006

OTU_75
p–value: 0.0949

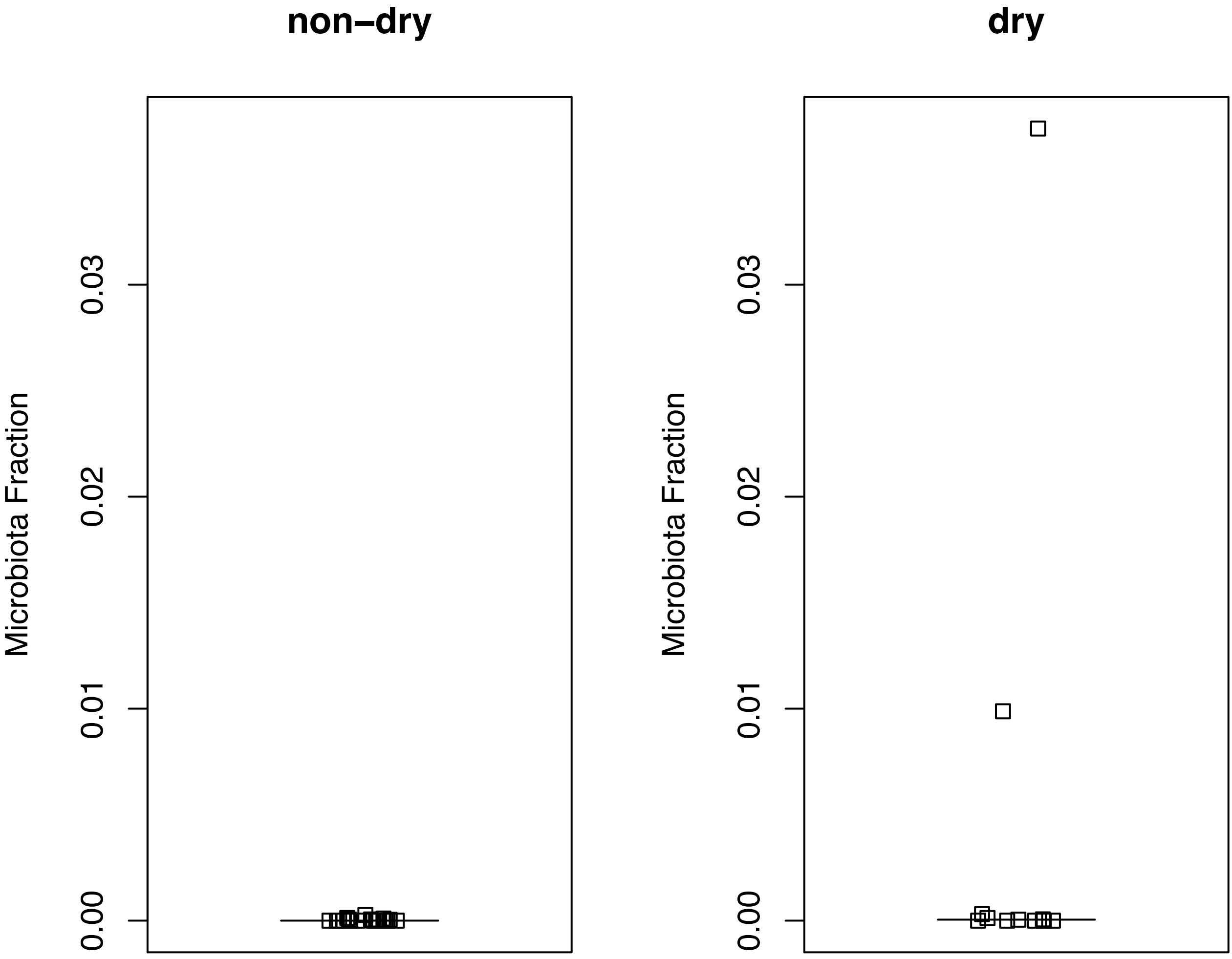

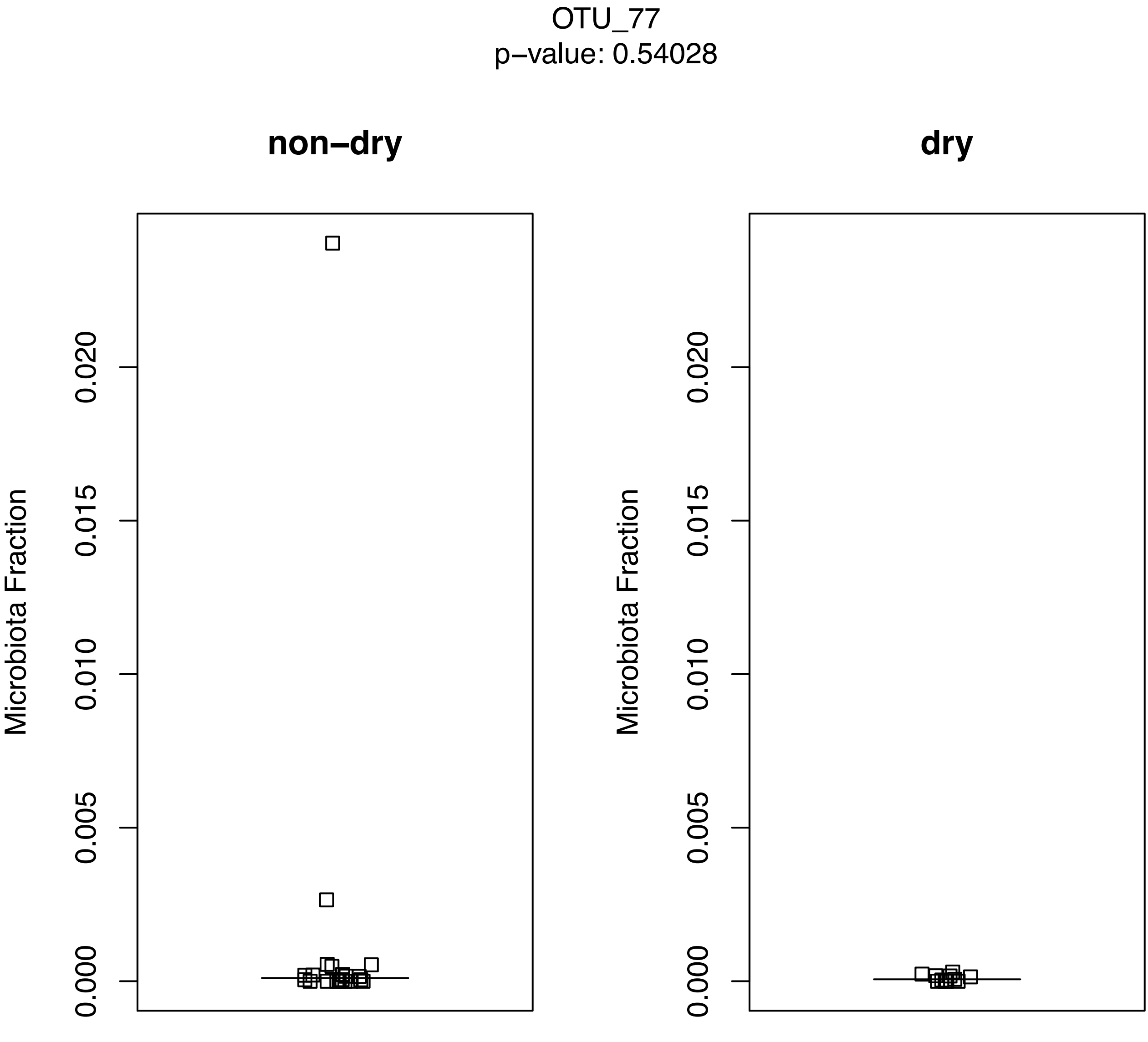
OTU_77
p−value: 0.54028
non−dry
dry
Microbiota Fraction
0.000
0.005
0.010
0.015
0.020
Microbiota Fraction
0.000
0.005
0.010
0.015
0.020

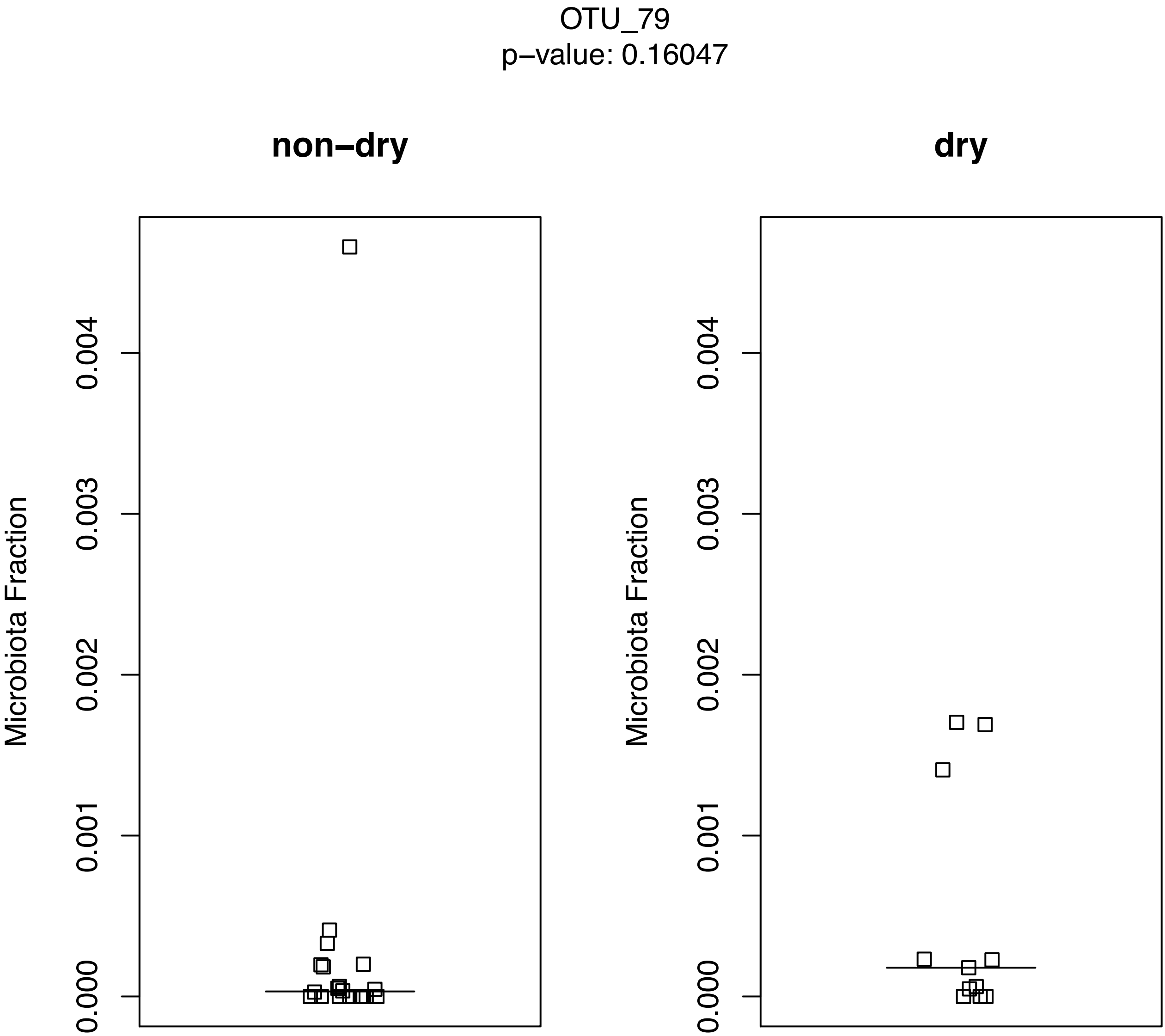
OTU_79
p−value: 0.16047
non−dry
dry
Microbiota Fraction
0.000
0.001
0.002
0.003
0.004

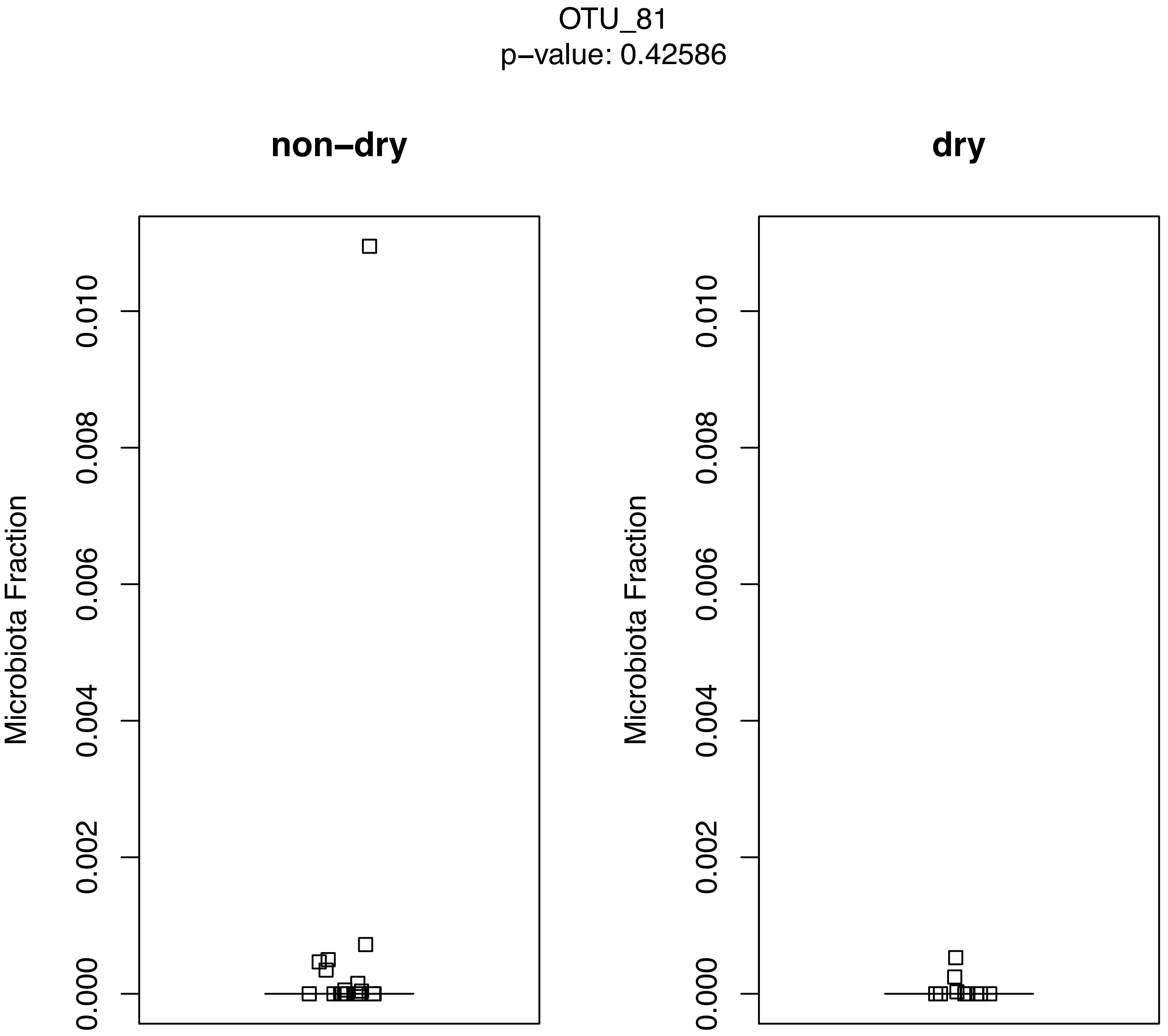
OTU_81
p−value: 0.42586
non−dry
dry
Microbiota Fraction
0.000
0.002
0.004
0.006
0.008
0.010
Microbiota Fraction
0.000
0.002
0.004
0.006
0.008
0.010

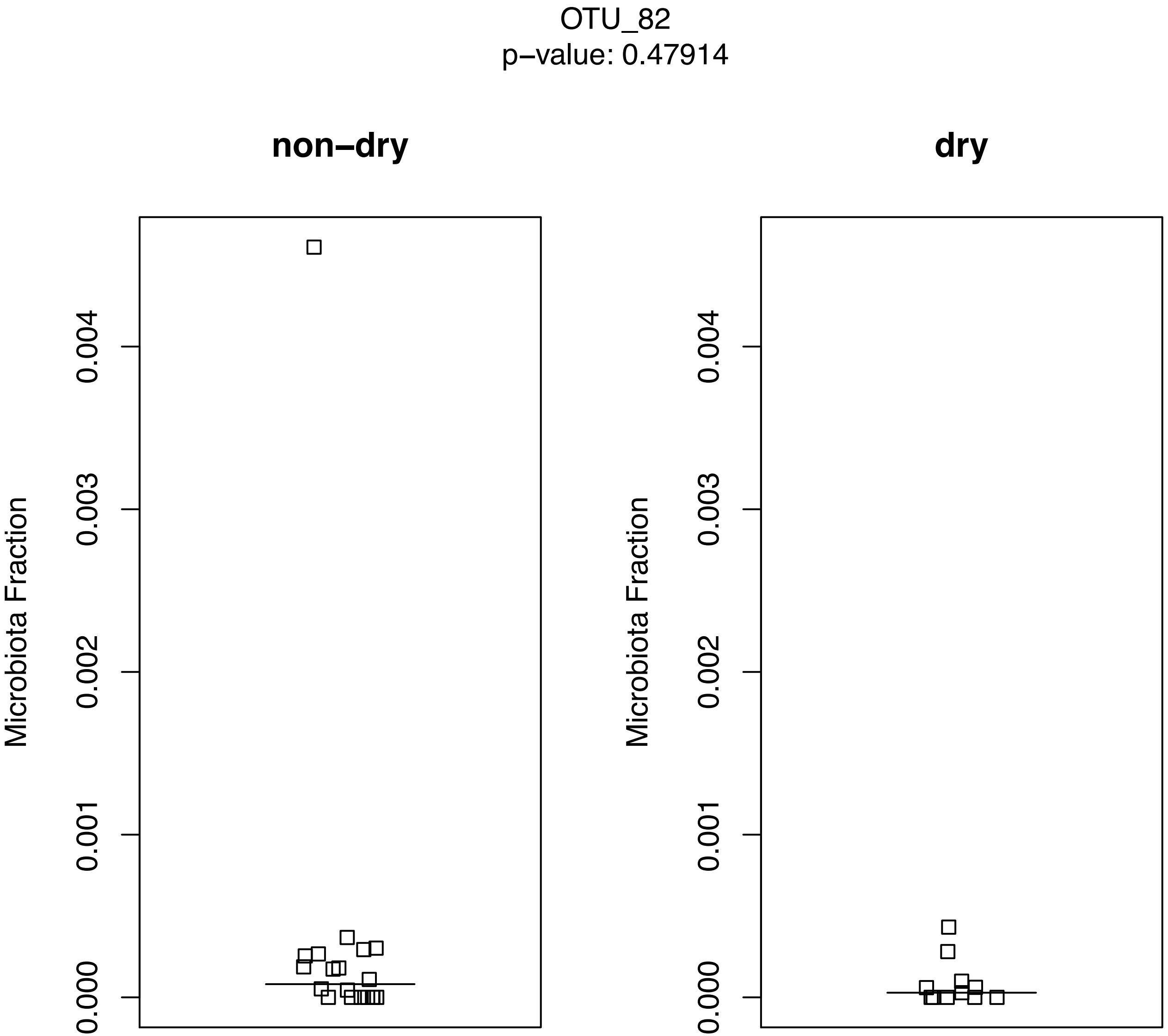
OTU_82
p−value: 0.47914
non−dry
dry
Microbiota Fraction
0.000
0.001
0.002
0.003
0.004
Microbiota Fraction
0.000
0.001
0.002
0.003
0.004

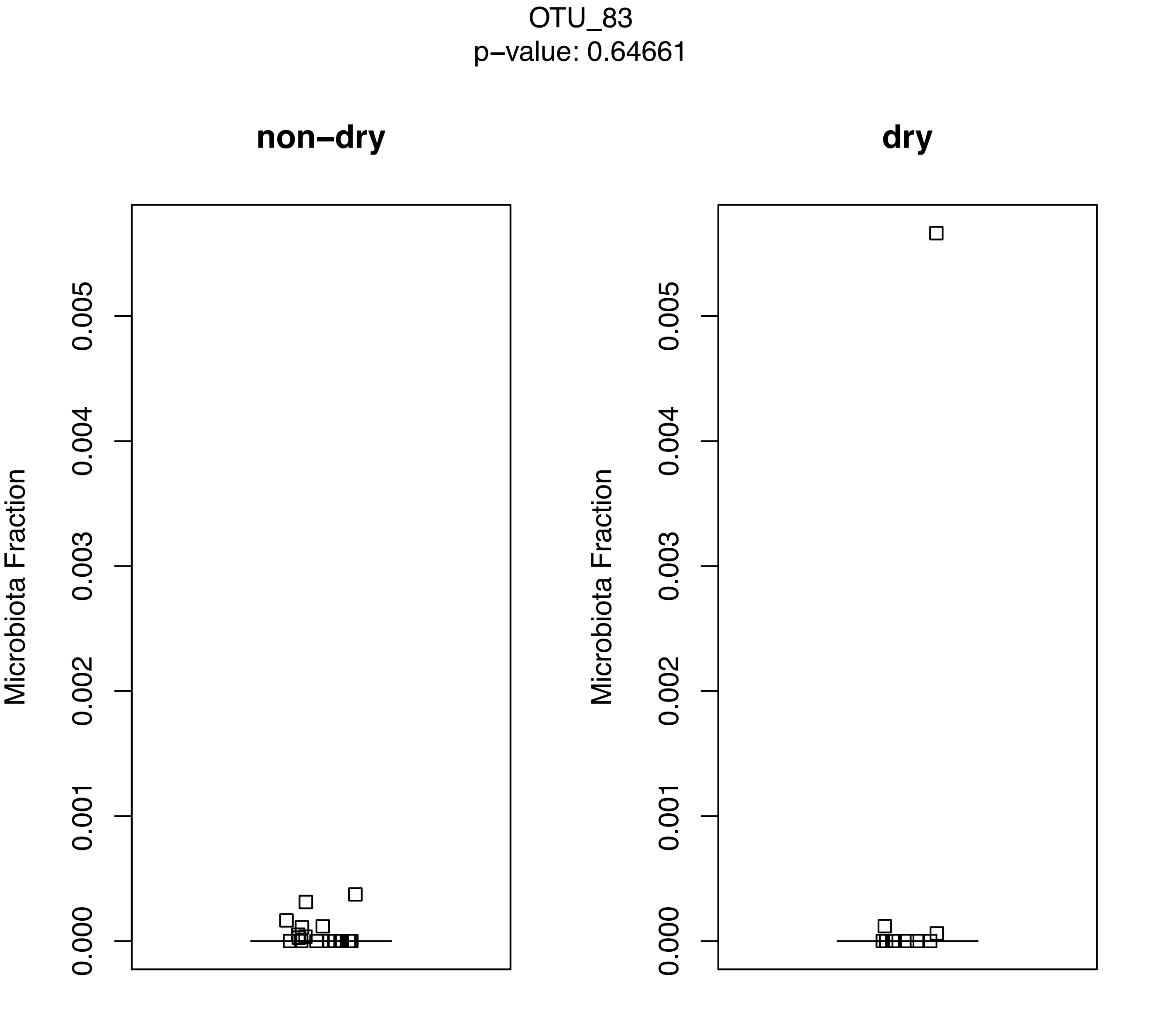

OTU_83
p−value: 0.64661
non−dry
dry
Microbiota Fraction
0.000
0.001
0.002
0.003
0.004
0.005
Microbiota Fraction
0.000
0.001
0.002
0.003
0.004
0.005

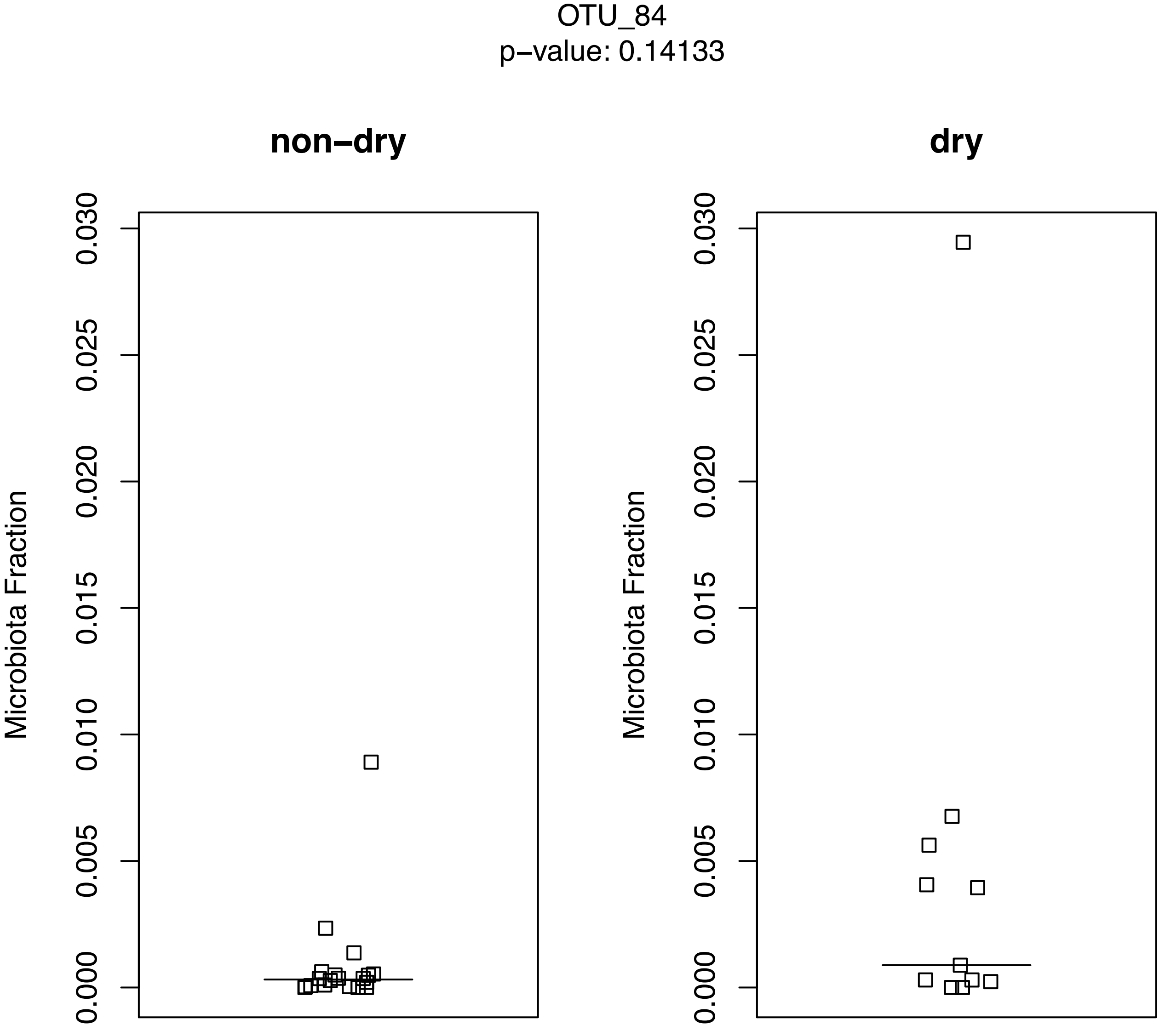
OTU_84
p−value: 0.14133
non−dry
dry
Microbiota Fraction
0.000
0.005
0.010
0.015
0.020
0.025
0.030

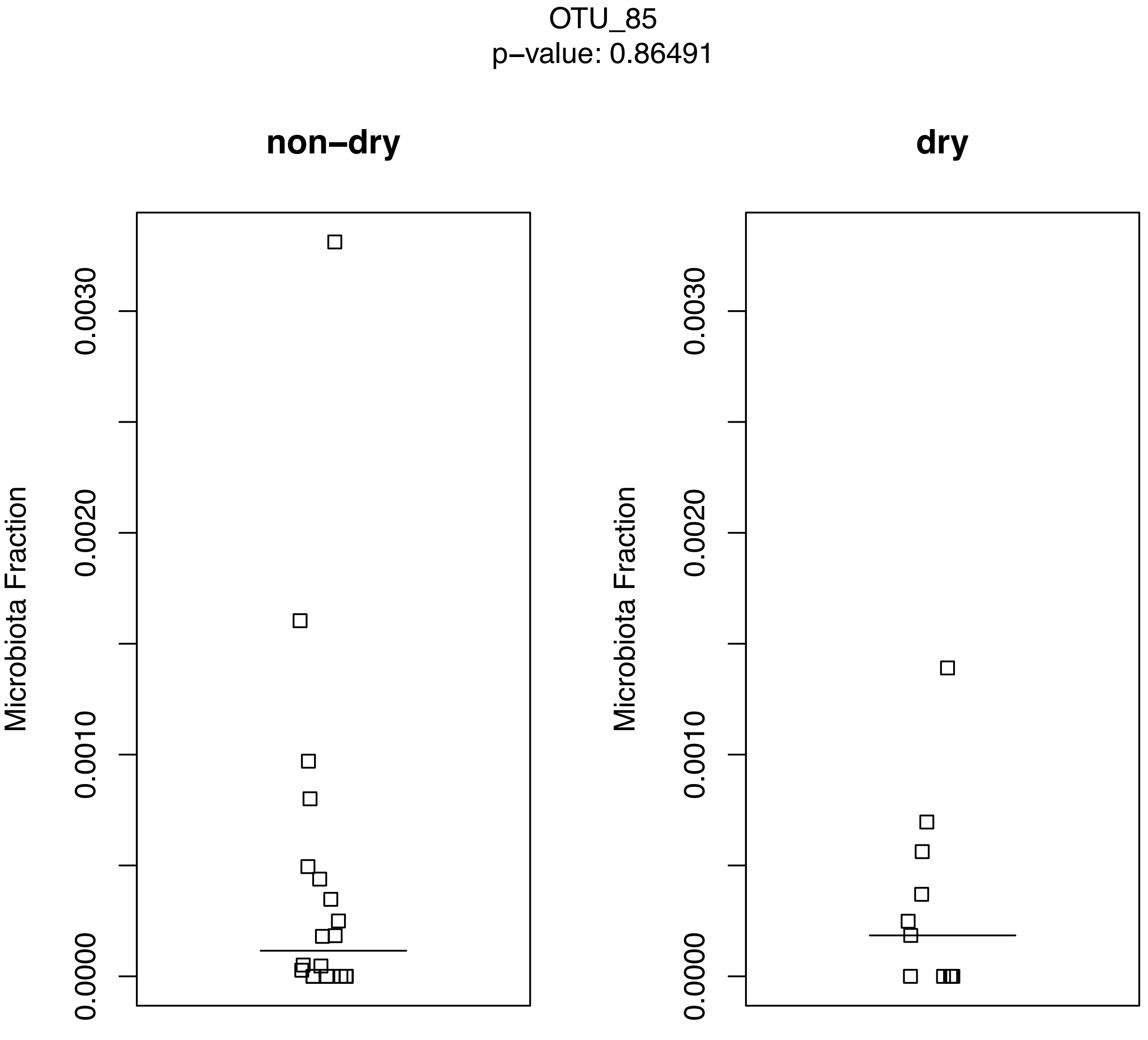

OTU_85
p−value: 0.86491
non−dry
dry
Microbiota Fraction
0.0000
0.0010
0.0020
0.0030
Microbiota Fraction
0.0000
0.0010
0.0020
0.0030

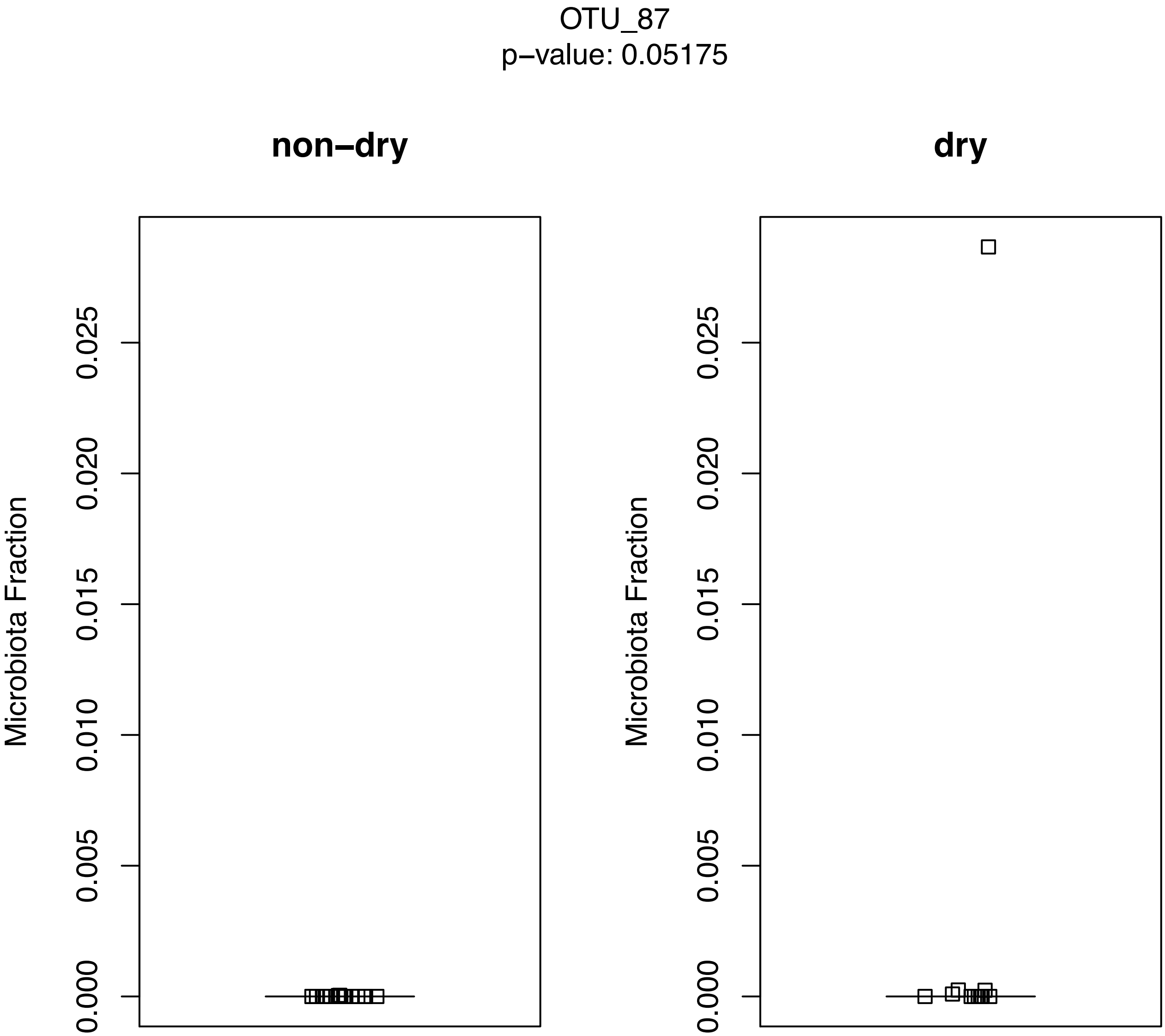
OTU_87
p−value: 0.05175
non−dry
dry
Microbiota Fraction
0.000
0.005
0.010
0.015
0.020
0.025
Microbiota Fraction
0.000
0.005
0.010
0.015
0.020
0.025

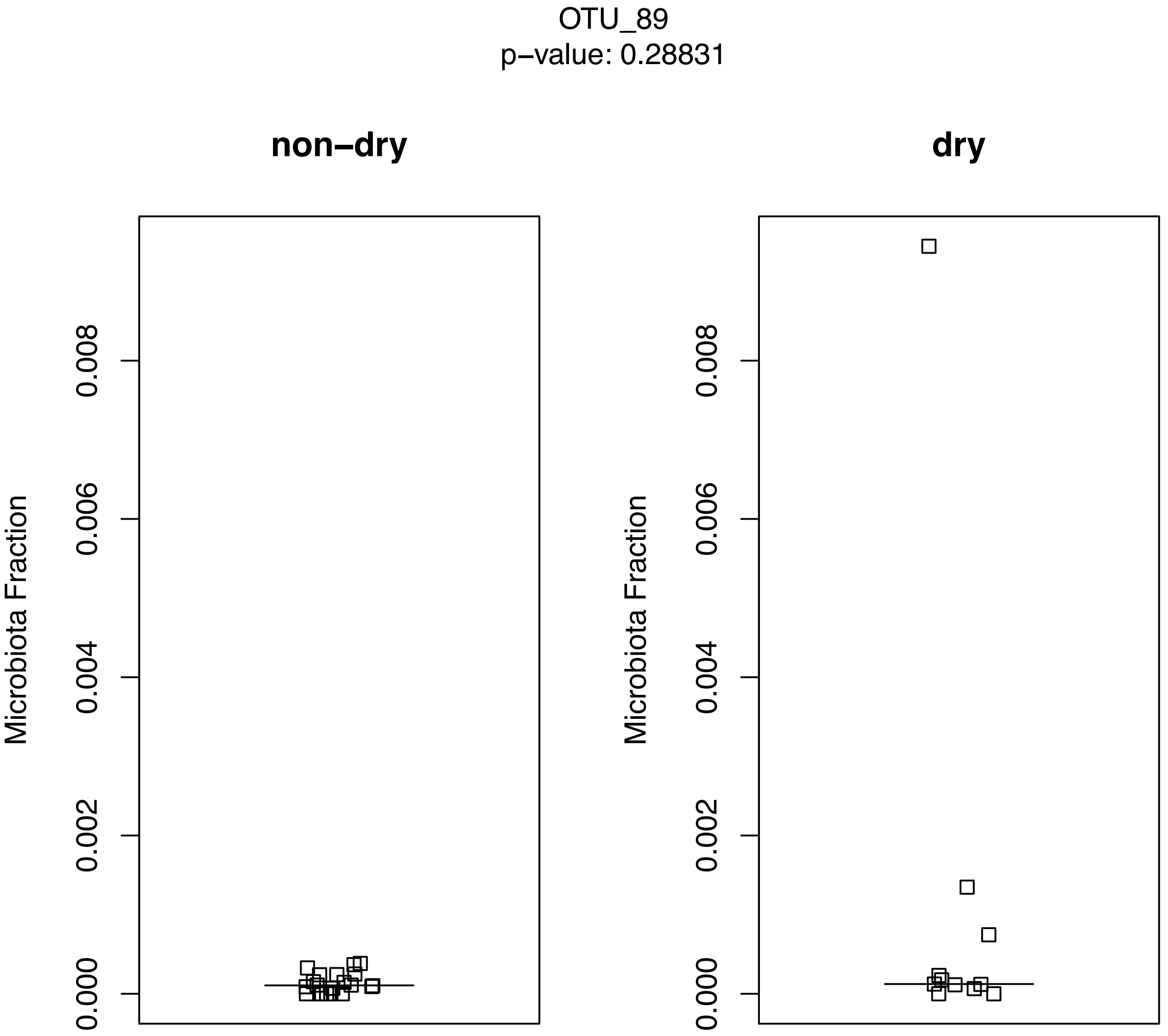

OTU_89
p−value: 0.28831
non−dry
dry
Microbiota Fraction
0.000
0.002
0.004
0.006
0.008
Microbiota Fraction
0.000
0.002
0.004
0.006
0.008

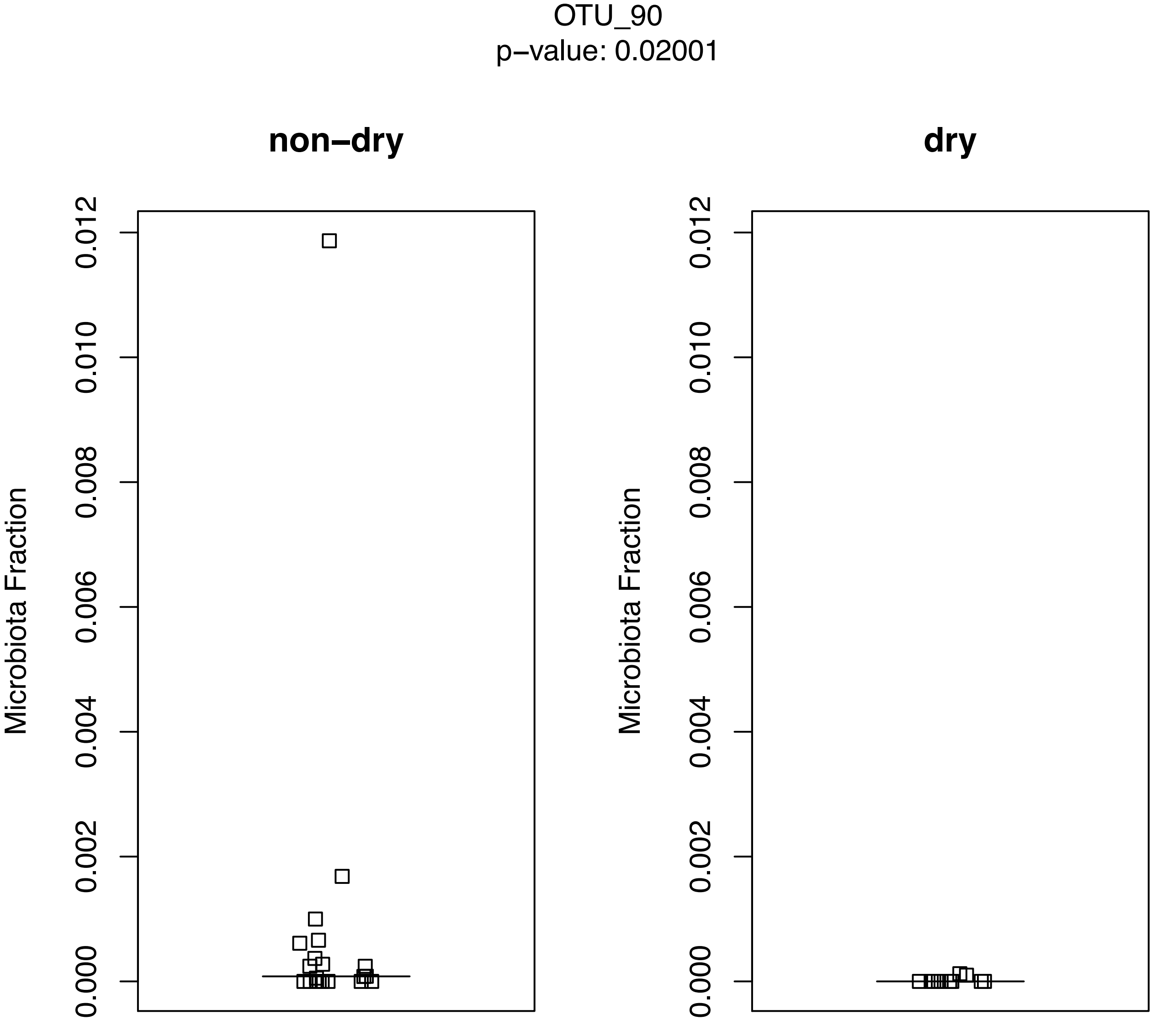
OTU_90
p−value: 0.02001
non−dry
dry
Microbiota Fraction
0.000
0.002
0.004
0.006
0.008
0.010
0.012
Microbiota Fraction
0.000
0.002
0.004
0.006
0.008
0.010
0.012

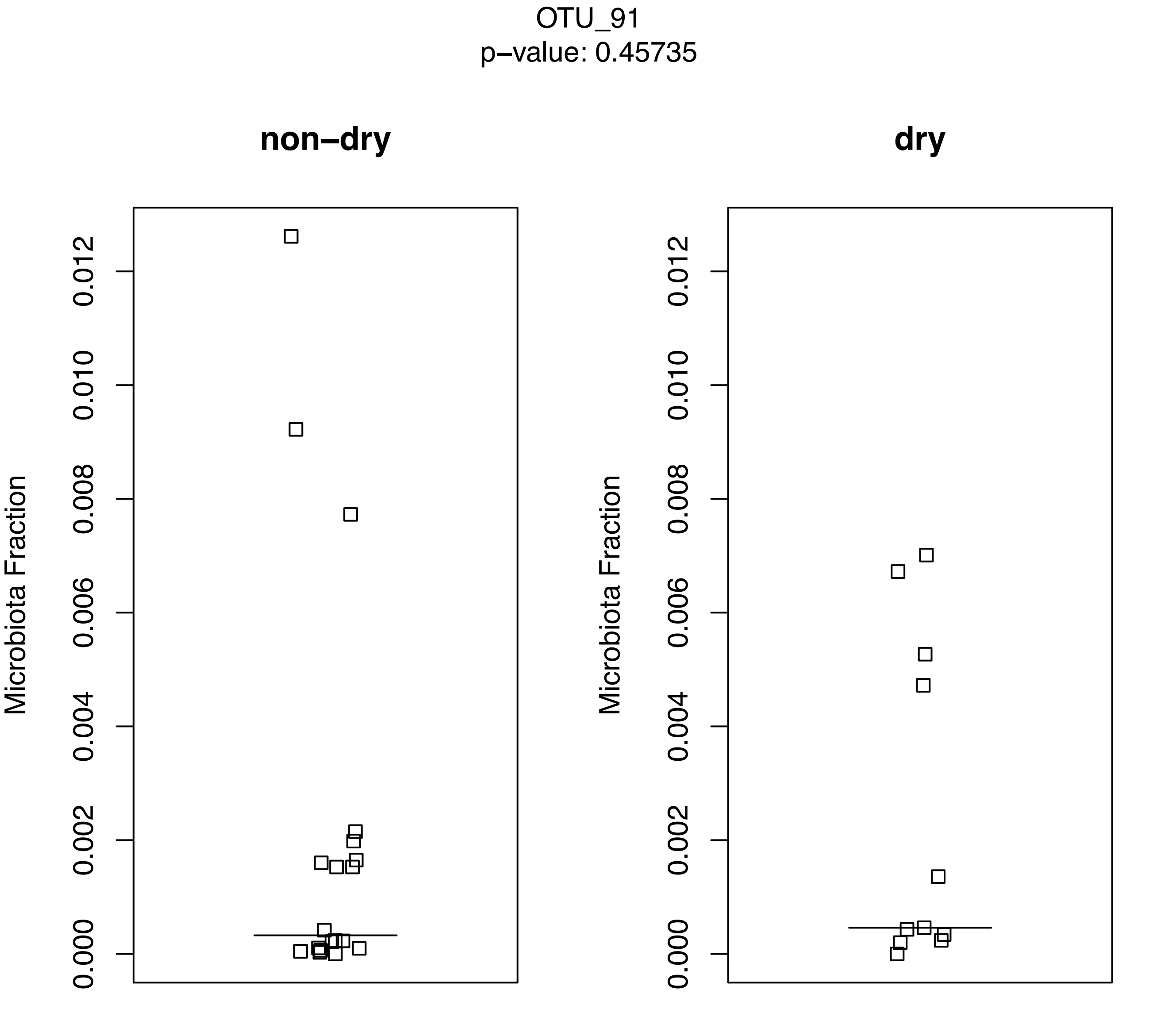

OTU_91
p−value: 0.45735
non−dry
dry
Microbiota Fraction
0.000
0.002
0.004
0.006
0.008
0.010
0.012

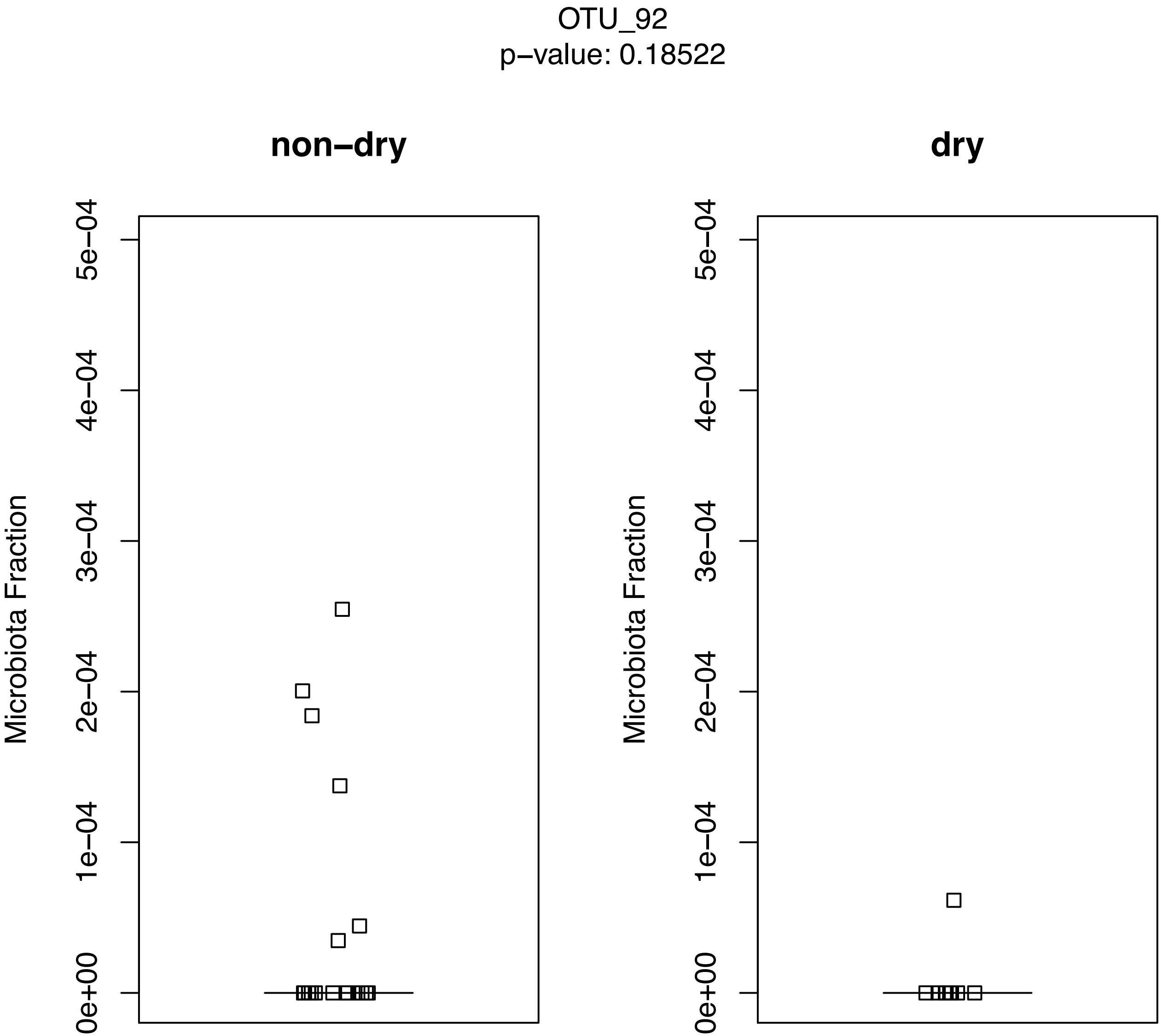
OTU_92
p−value: 0.18522
non−dry
dry
Microbiota Fraction
0e+00
1e−04
2e−04
3e−04
4e−04
5e−04

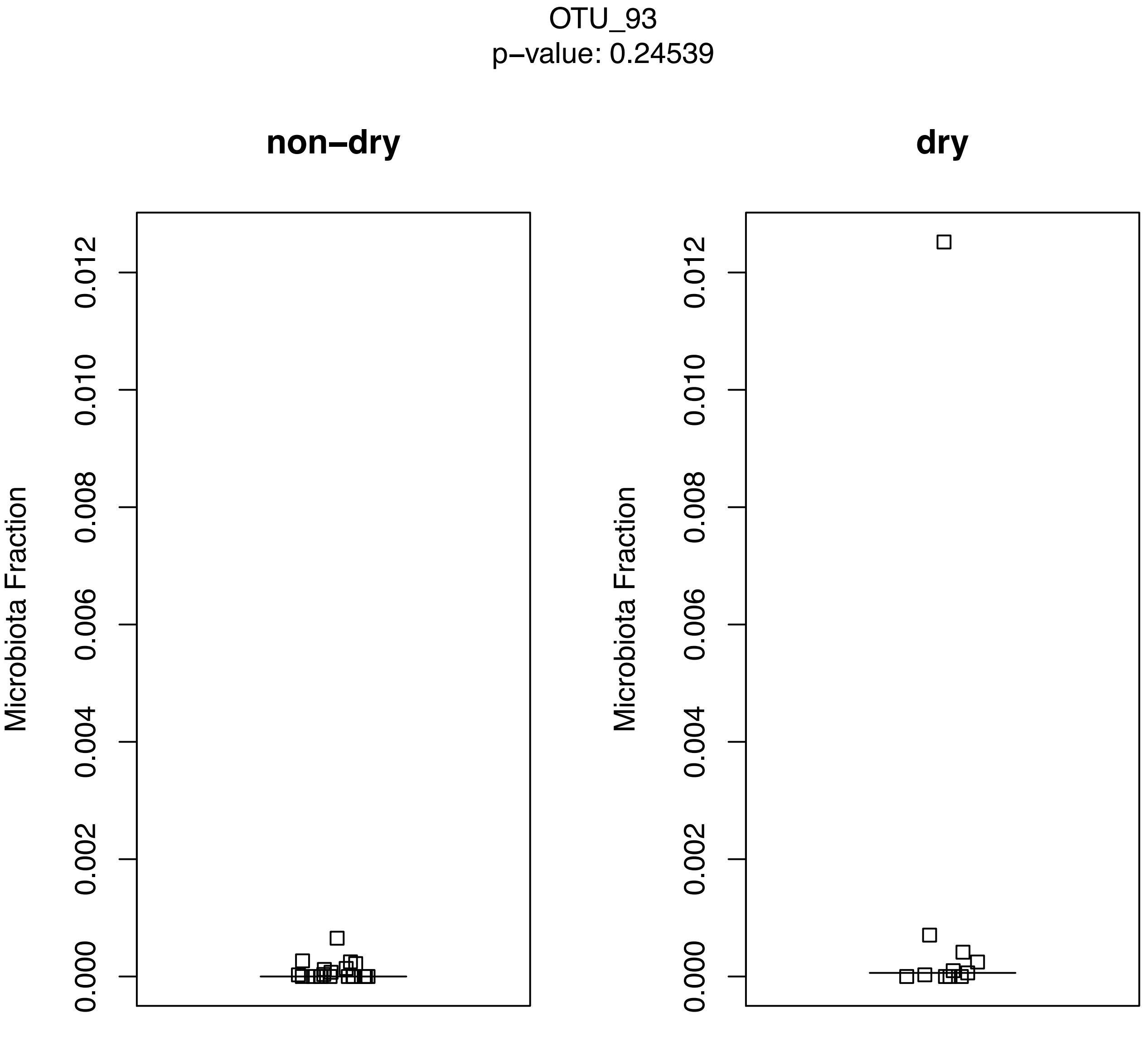
OTU_93
p–value: 0.24539
non–dry
dry
Microbiota Fraction
0.000
0.002
0.004
0.006
0.008
0.010
0.012

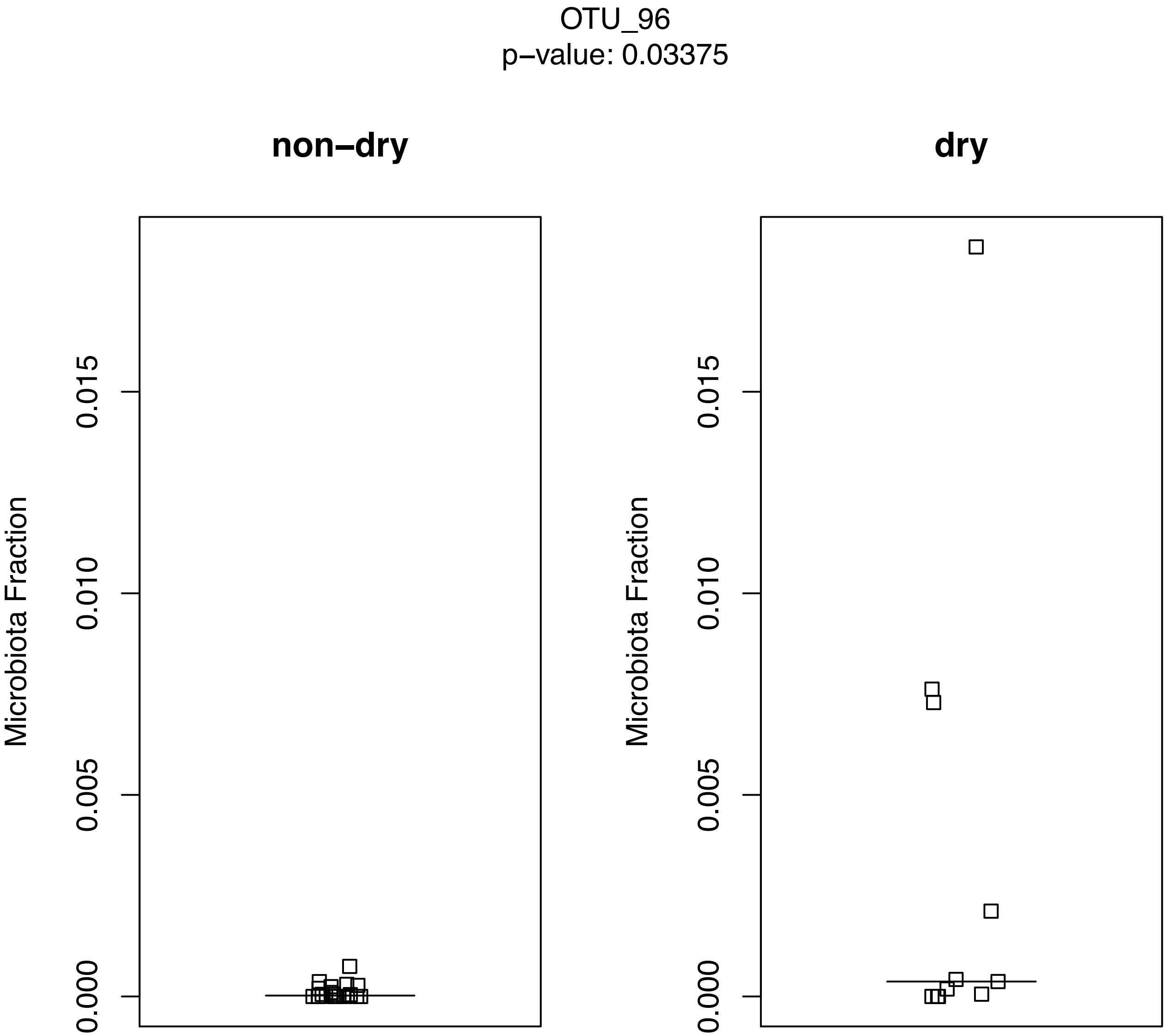

OTU_96
p−value: 0.03375
non−dry
dry
Microbiota Fraction
0.000
0.005
0.010
0.015
Microbiota Fraction
0.000
0.005
0.010
0.015

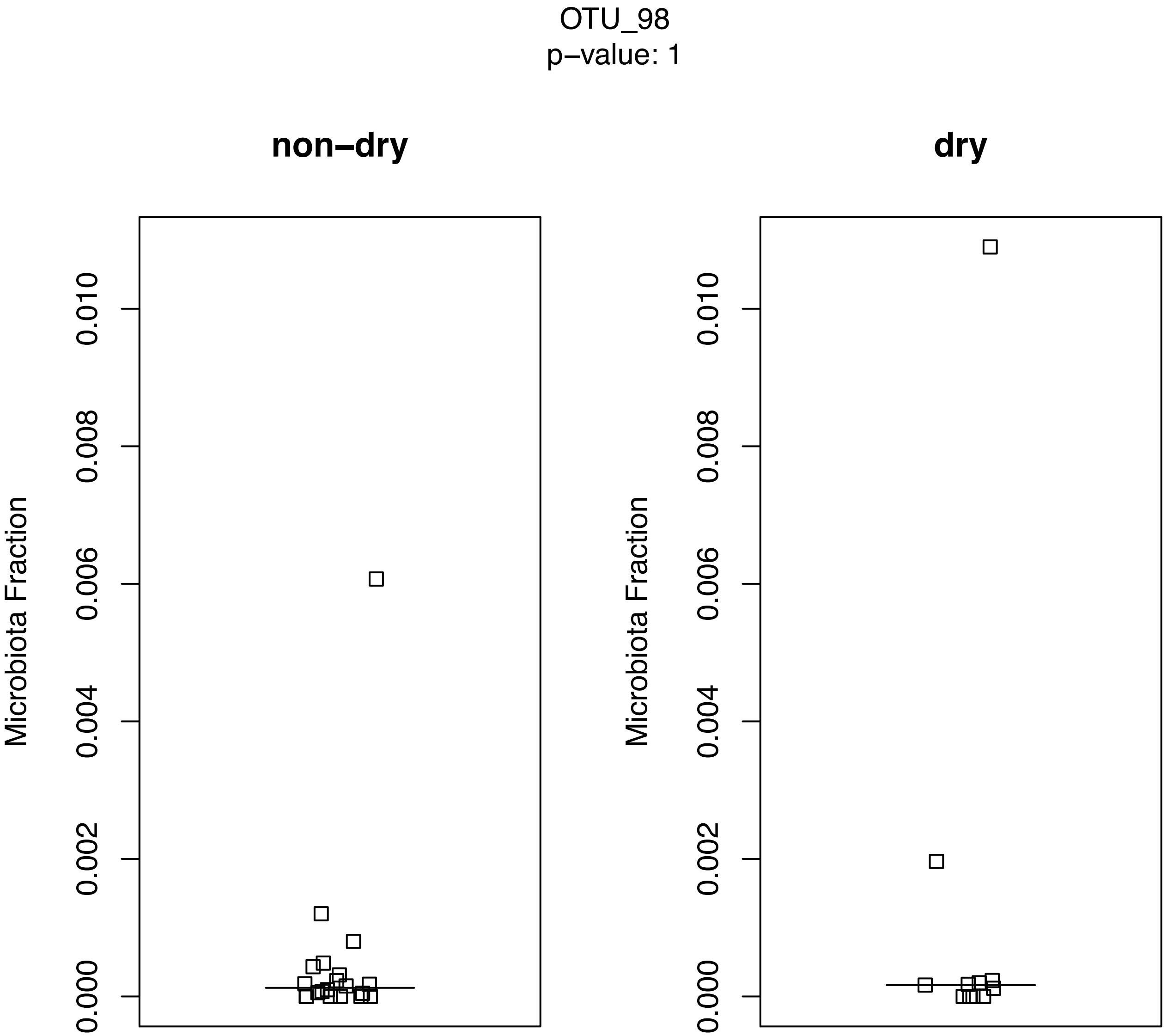

OTU_98
p−value: 1
non−dry
dry
Microbiota Fraction
Microbiota Fraction
0.000
0.002
0.004
0.006
0.008
0.010
0.000
0.002
0.004
0.006
0.008
0.010

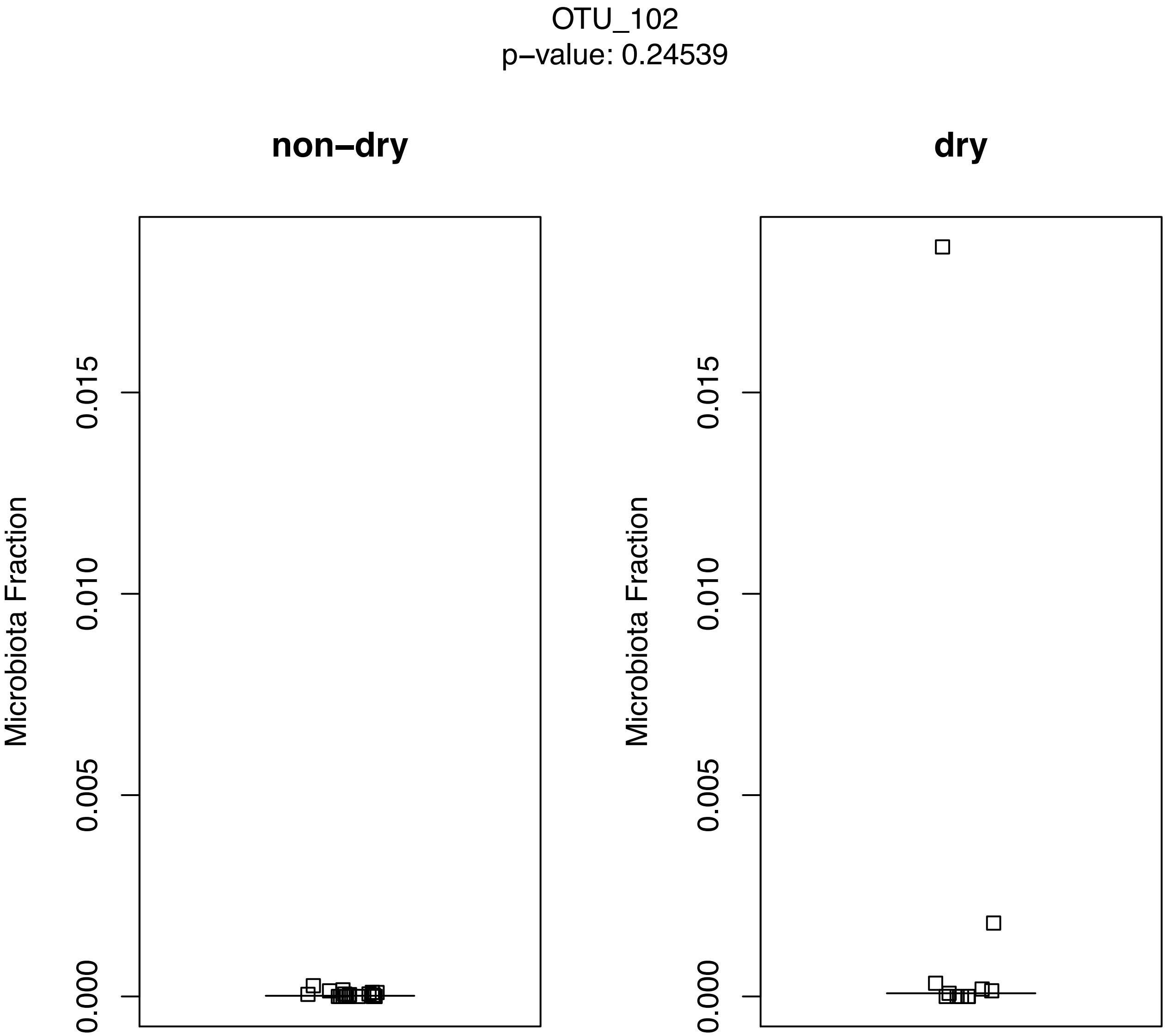

OTU_102
p−value: 0.24539
non−dry
dry
Microbiota Fraction
0.000
0.005
0.010
0.015

OTU_103
p−value: 1

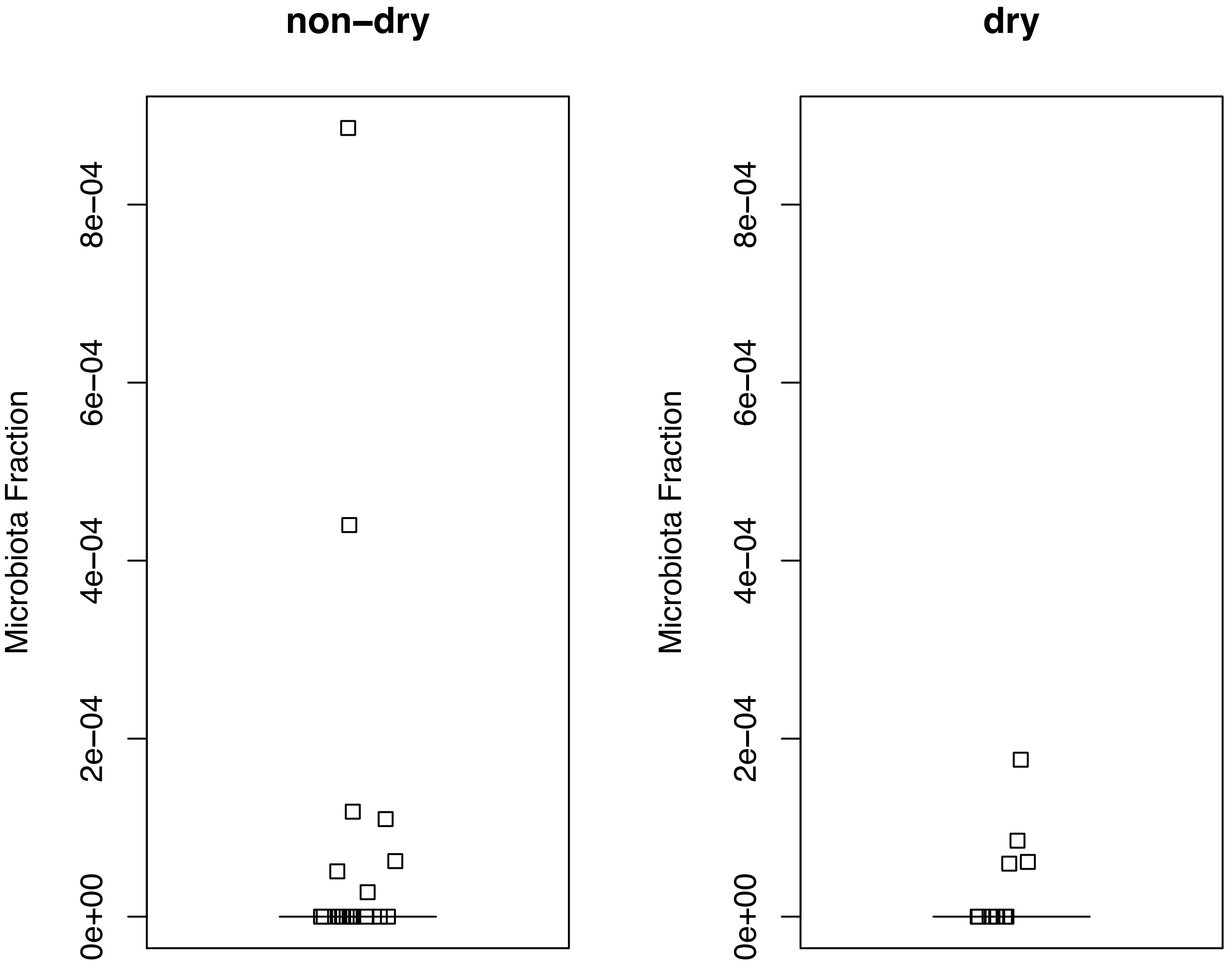

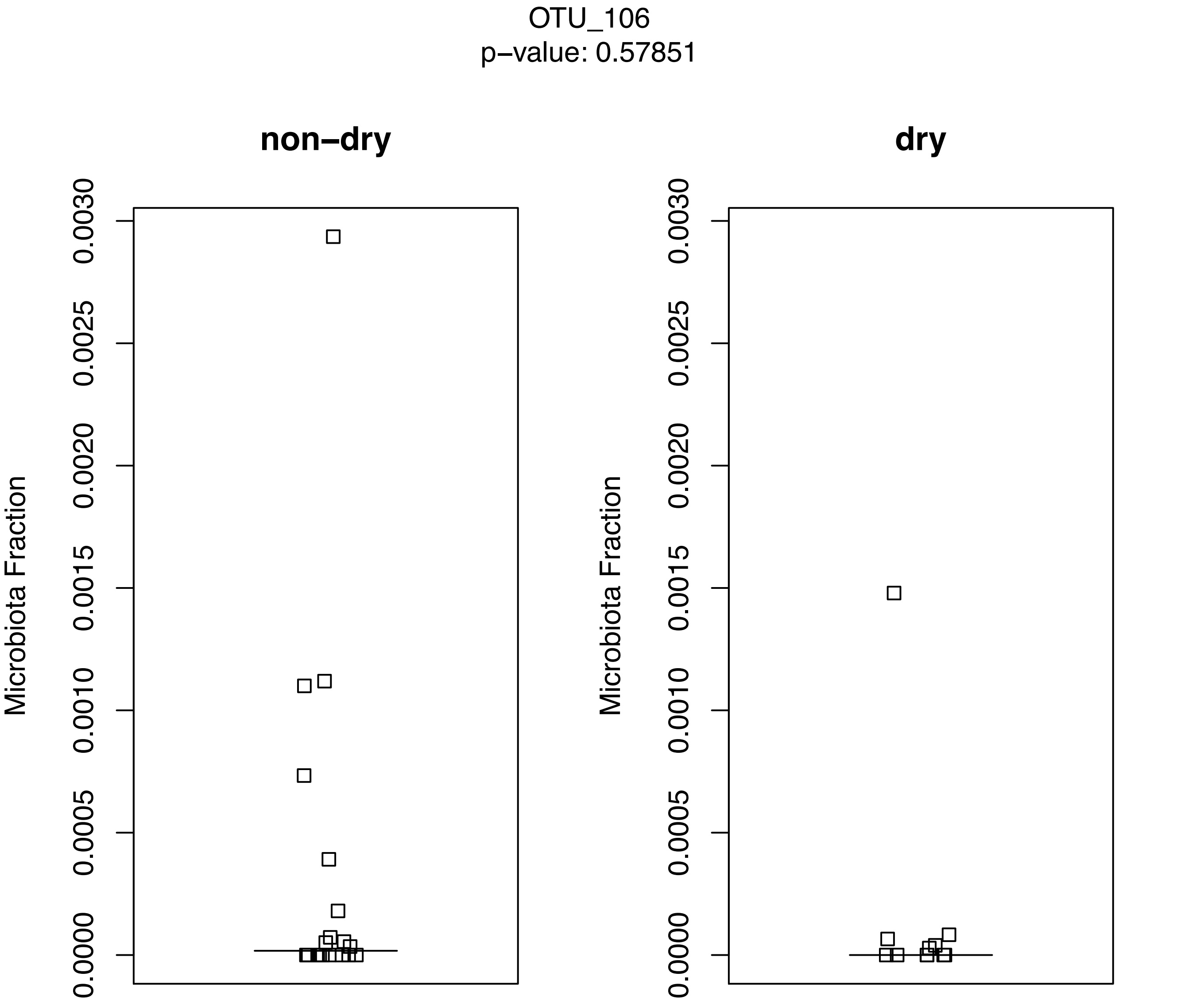
OTU_106
p−value: 0.57851
non−dry
dry
Microbiota Fraction
Microbiota Fraction
0.0000
0.0005
0.0010
0.0015
0.0020
0.0025
0.0030
0.0000
0.0005
0.0010
0.0015
0.0020
0.0025
0.0030

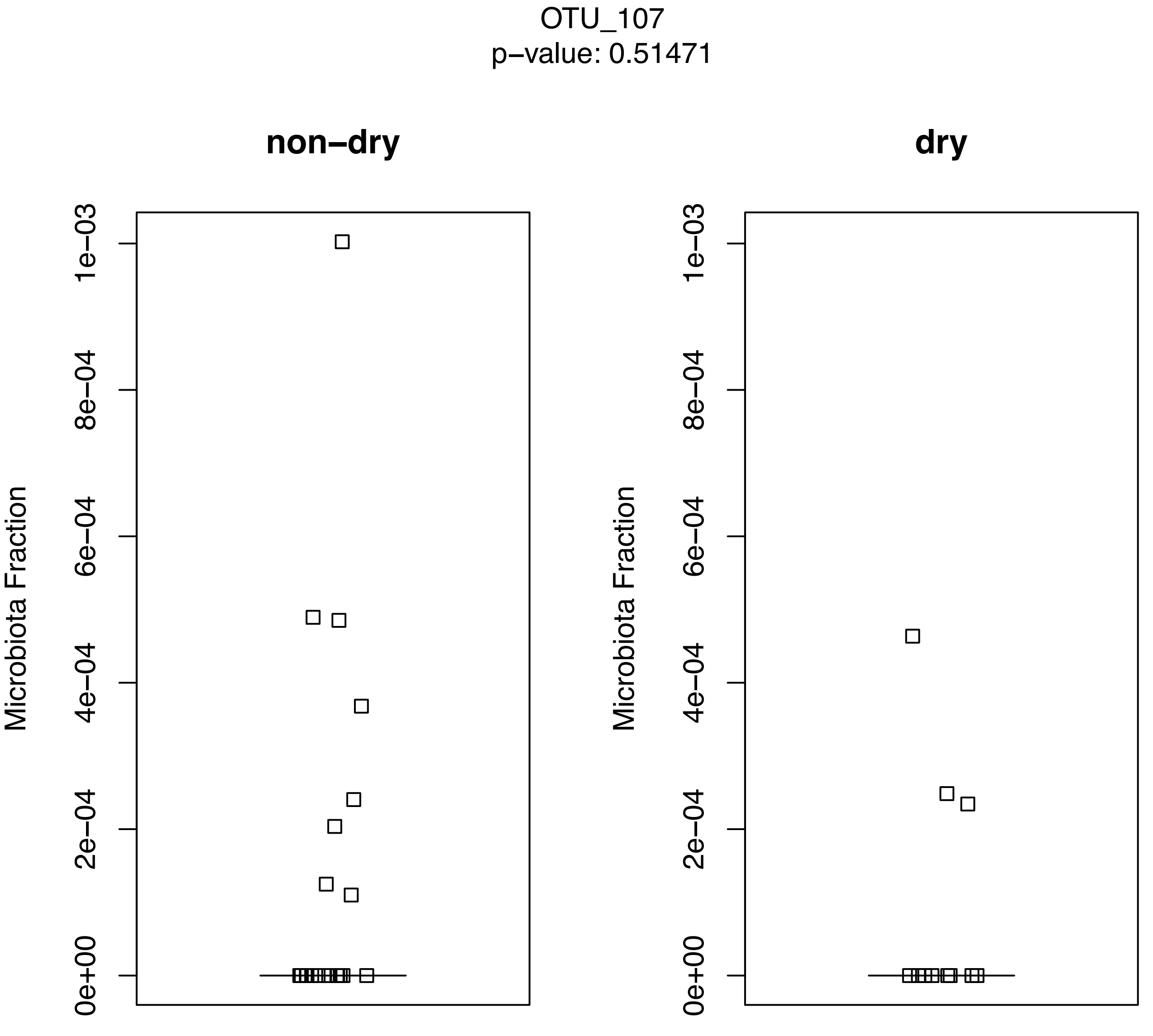
OTU_107
p−value: 0.51471
non−dry
dry
Microbiota Fraction
Microbiota Fraction
0e+00
2e−04
4e−04
6e−04
8e−04
1e−03
0e+00
2e−04
4e−04
6e−04
8e−04
1e−03

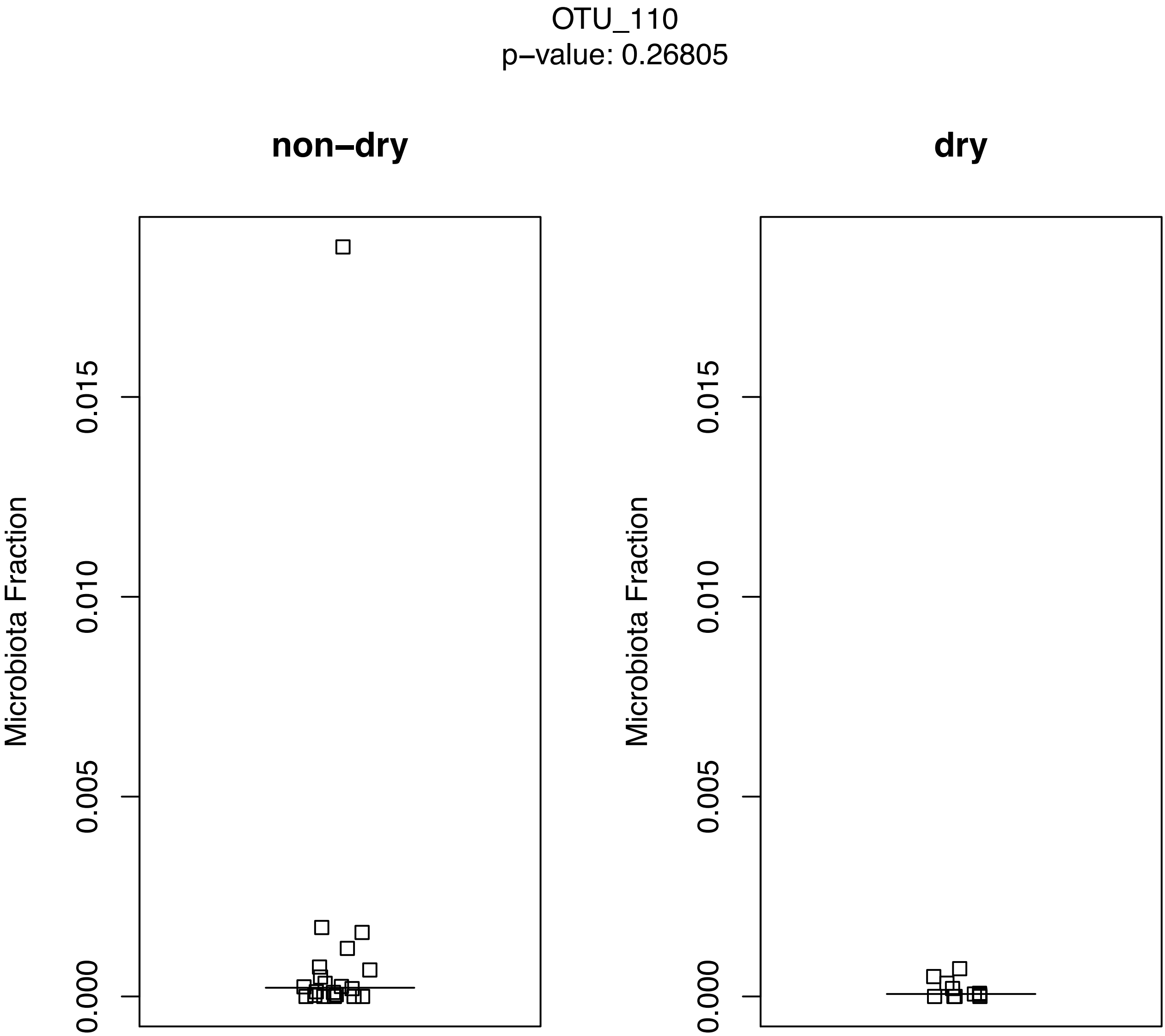

OTU_110
p−value: 0.26805
non−dry
dry
Microbiota Fraction
0.000
0.005
0.010
0.015
Microbiota Fraction
0.000
0.005
0.010
0.015

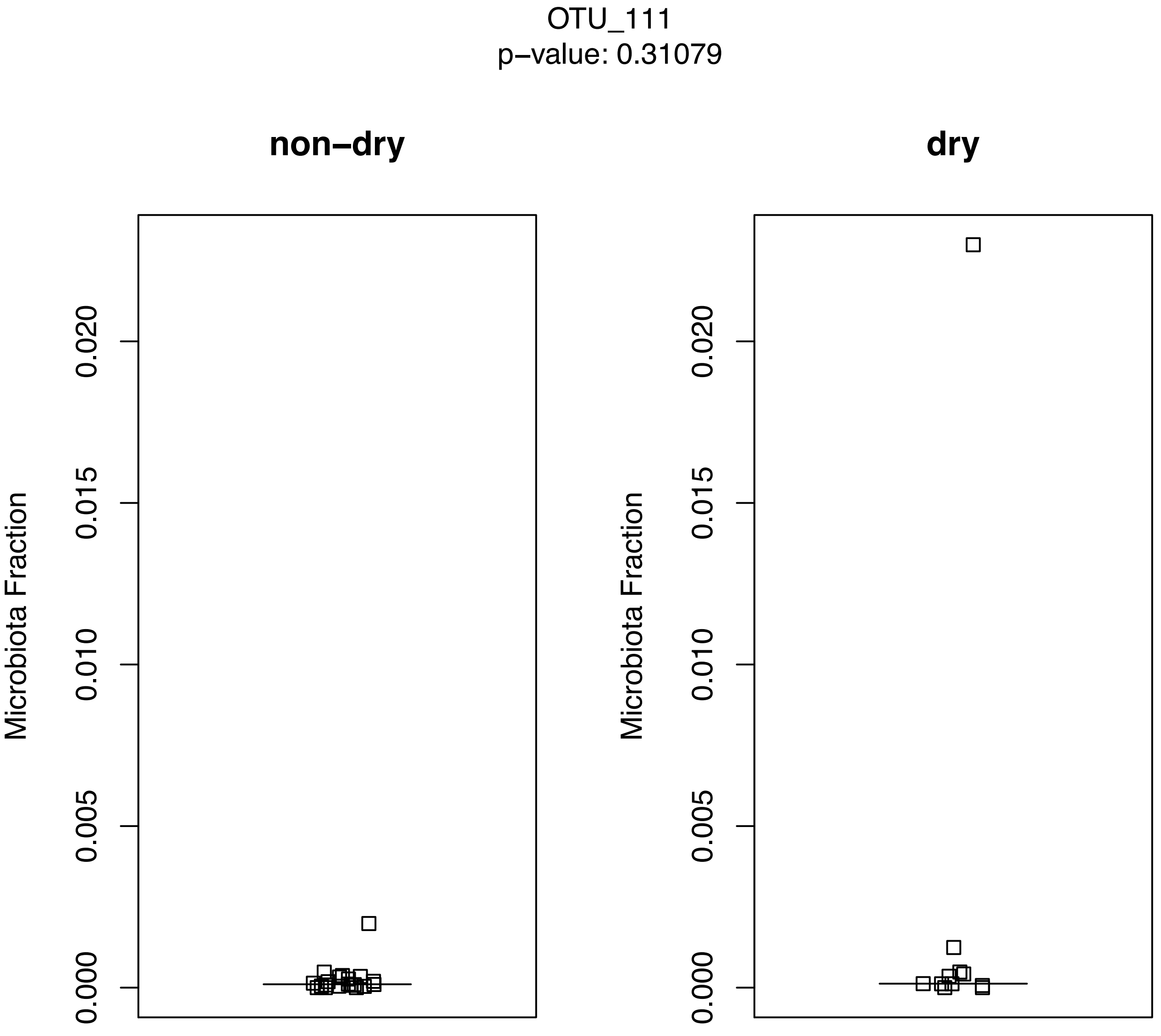

OTU_111
p–value: 0.31079
non–dry
dry
Microbiota Fraction
0.000
0.005
0.010
0.015
0.020

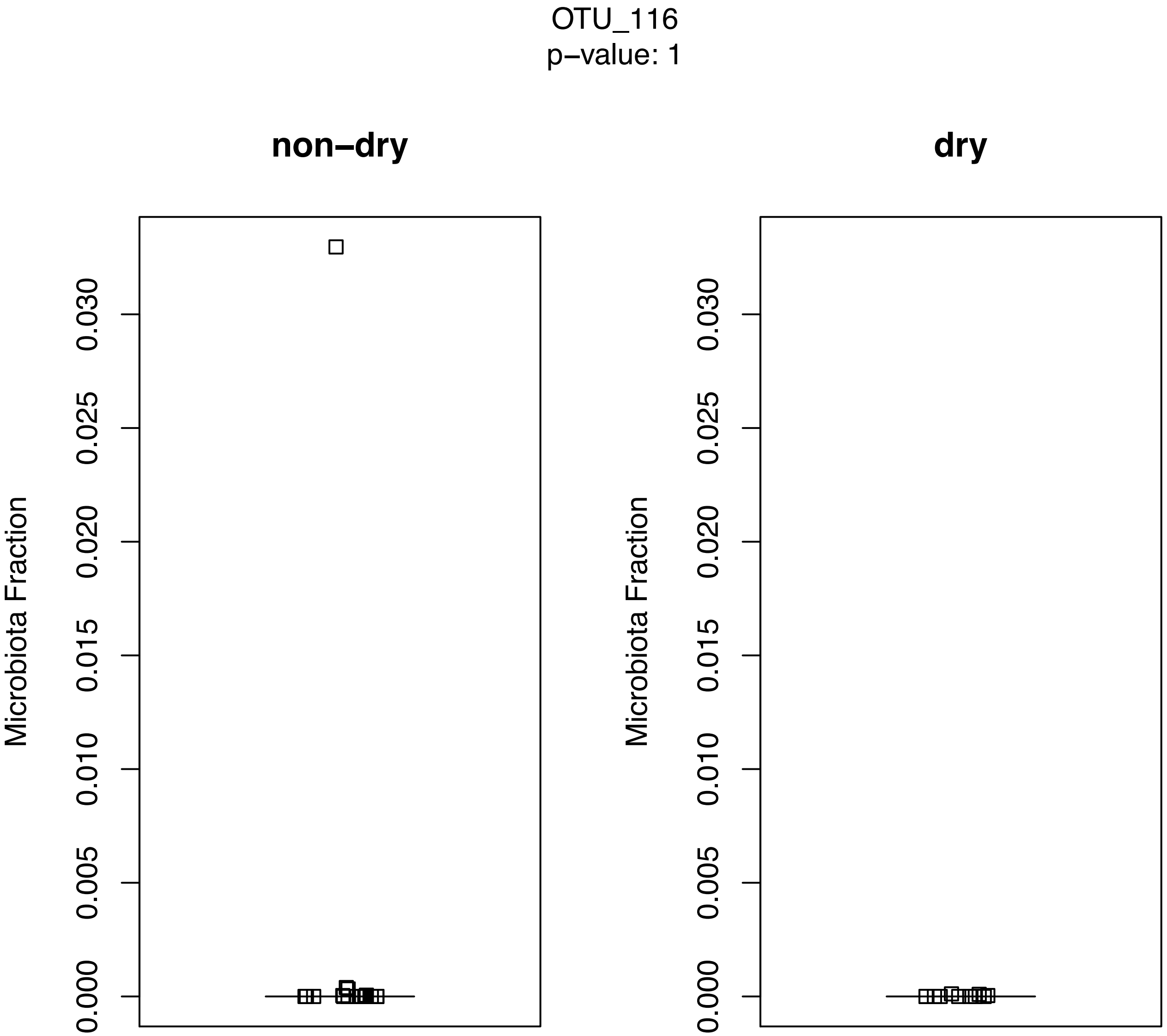

OTU_116
p−value: 1
non−dry
dry
Microbiota Fraction
0.000
0.005
0.010
0.015
0.020
0.025
0.030
Microbiota Fraction
0.000
0.005
0.010
0.015
0.020
0.025
0.030

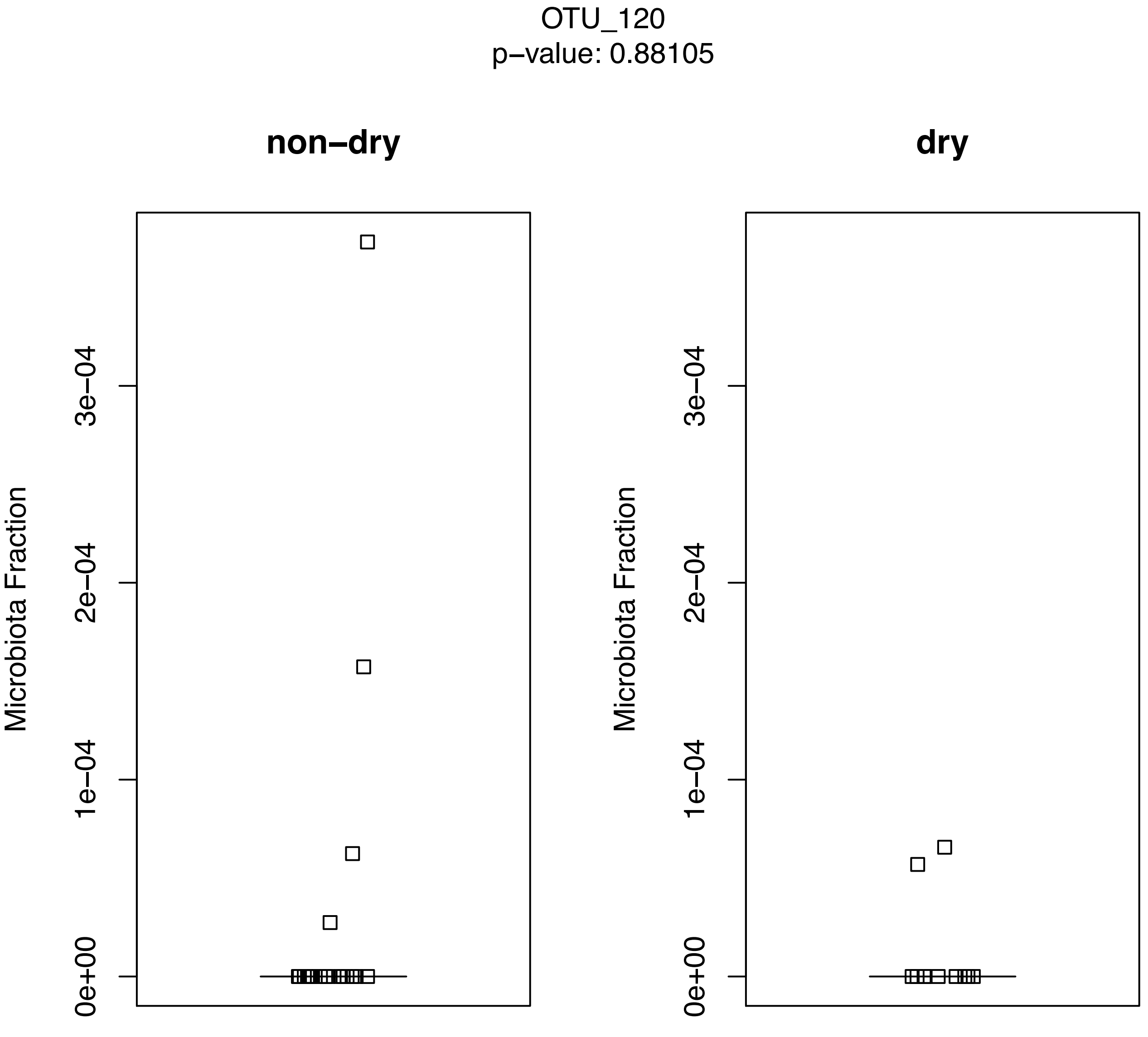

OTU_120
p−value: 0.88105
non−dry
dry
Microbiota Fraction
0e+00
1e−04
2e−04
3e−04
Microbiota Fraction
0e+00
1e−04
2e−04
3e−04

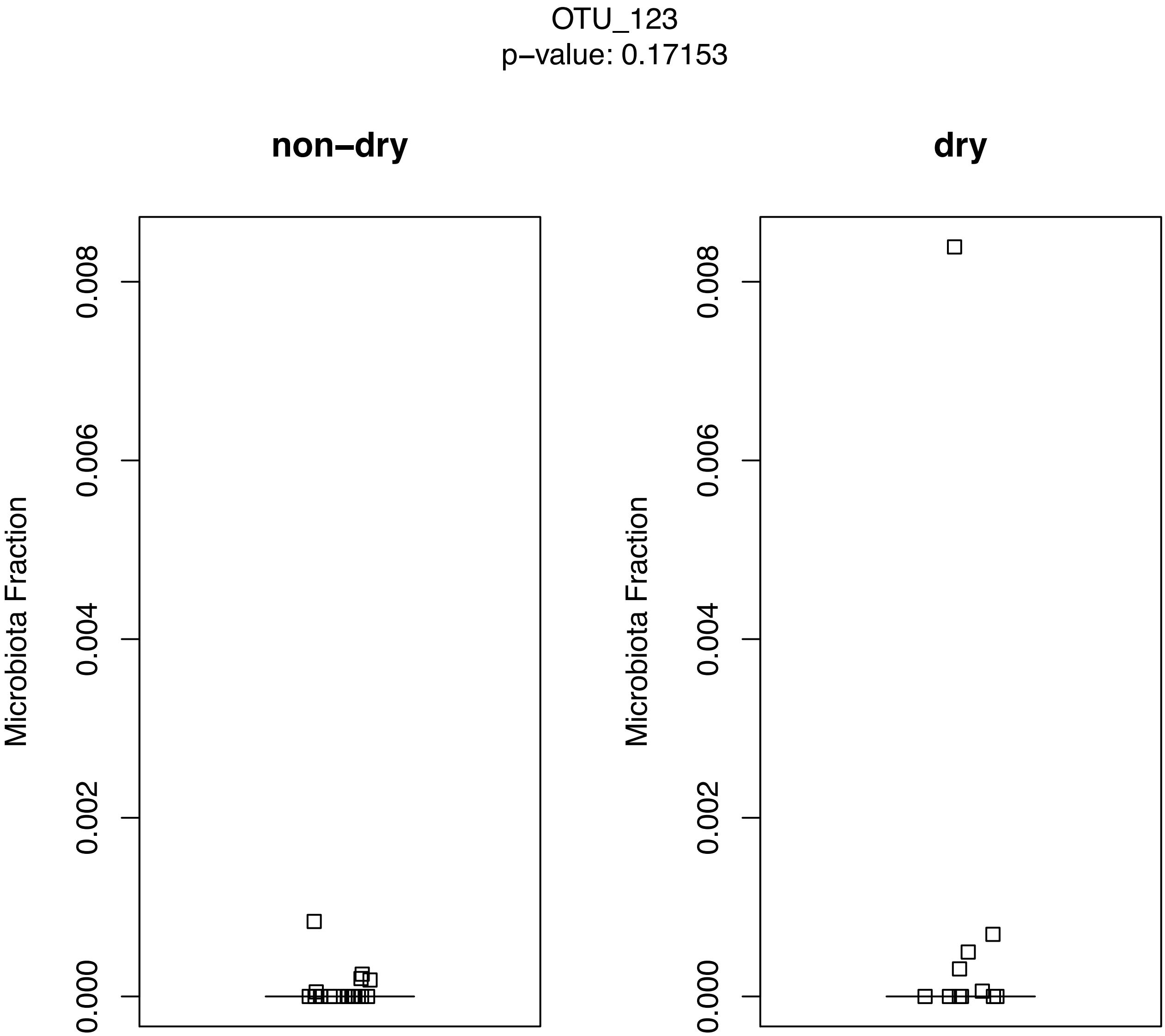
OTU_123
p−value: 0.17153
non−dry
dry
Microbiota Fraction
0.000
0.002
0.004
0.006
0.008
Microbiota Fraction
0.000
0.002
0.004
0.006
0.008

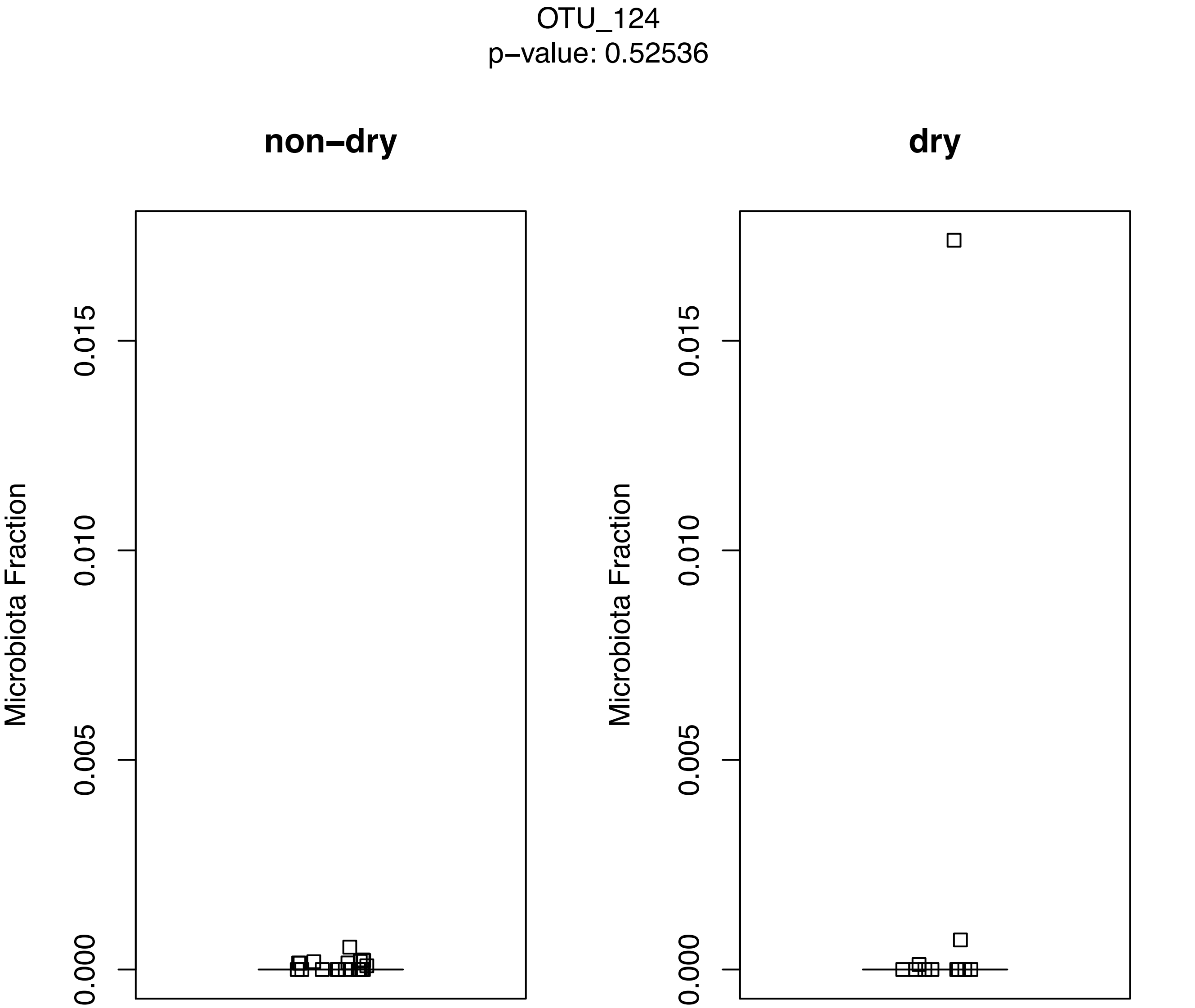
OTU_124
p−value: 0.52536
non−dry
dry
Microbiota Fraction
0.000
0.005
0.010
0.015
Microbiota Fraction
0.000
0.005
0.010
0.015

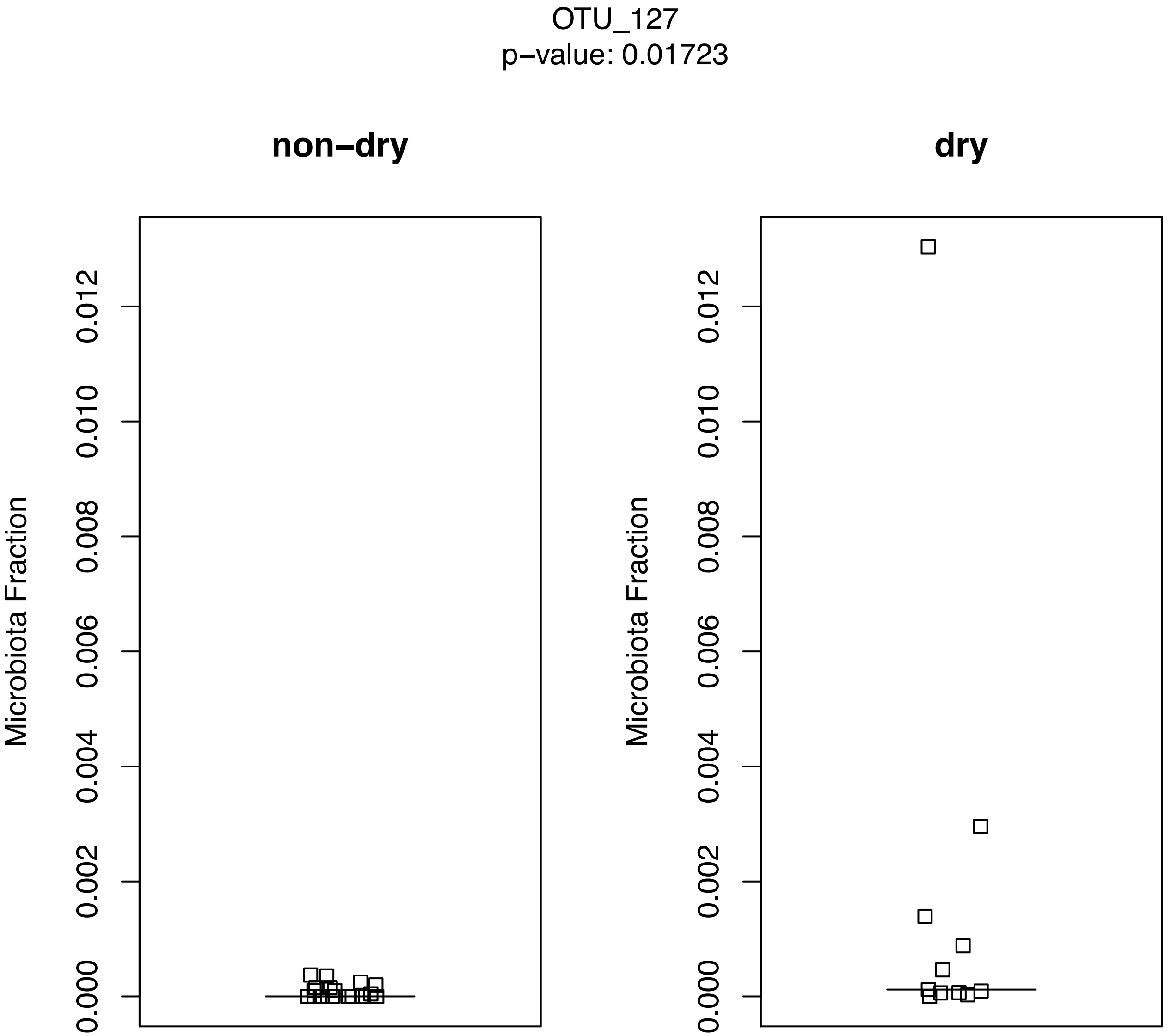
OTU_127
p−value: 0.01723
non−dry
dry
Microbiota Fraction
0.000
0.002
0.004
0.006
0.008
0.010
0.012

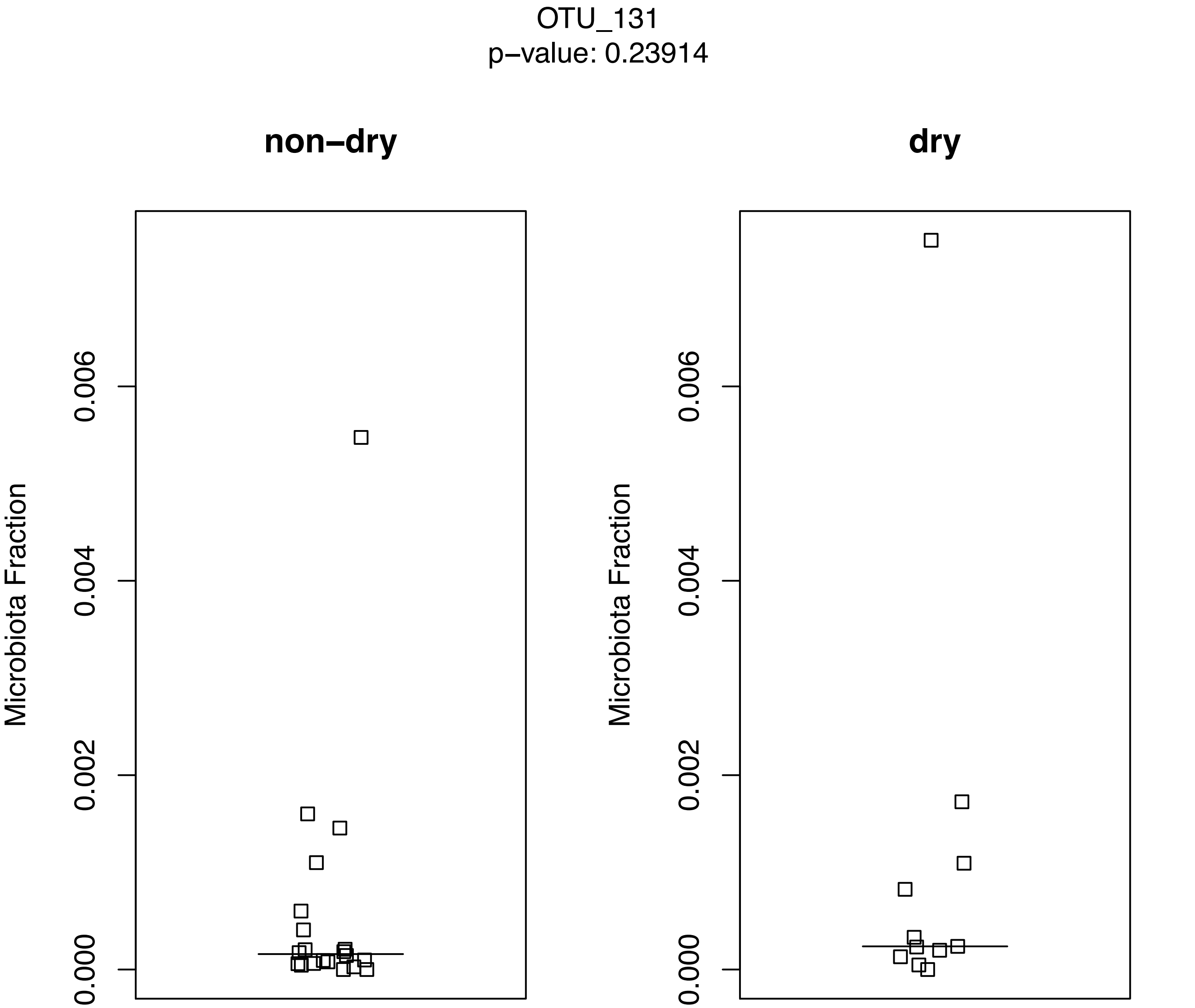
OTU_131
p–value: 0.23914
non–dry
dry
Microbiota Fraction
0.000
0.002
0.004
0.006
Microbiota Fraction
0.000
0.002
0.004
0.006

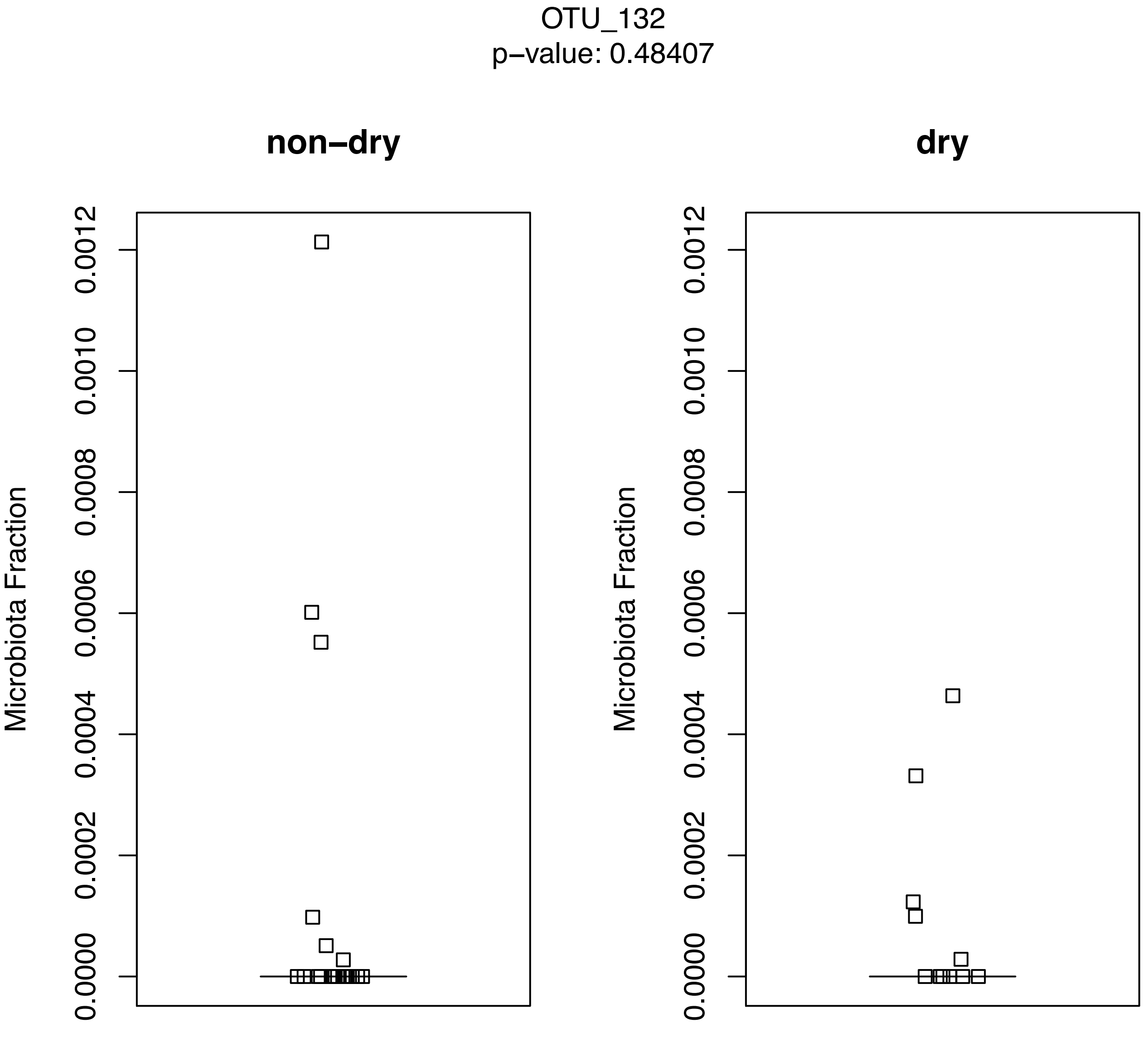
OTU_132
p−value: 0.48407
non−dry
dry
Microbiota Fraction
Microbiota Fraction
0.0000
0.0002
0.0004
0.0006
0.0008
0.0010
0.0012

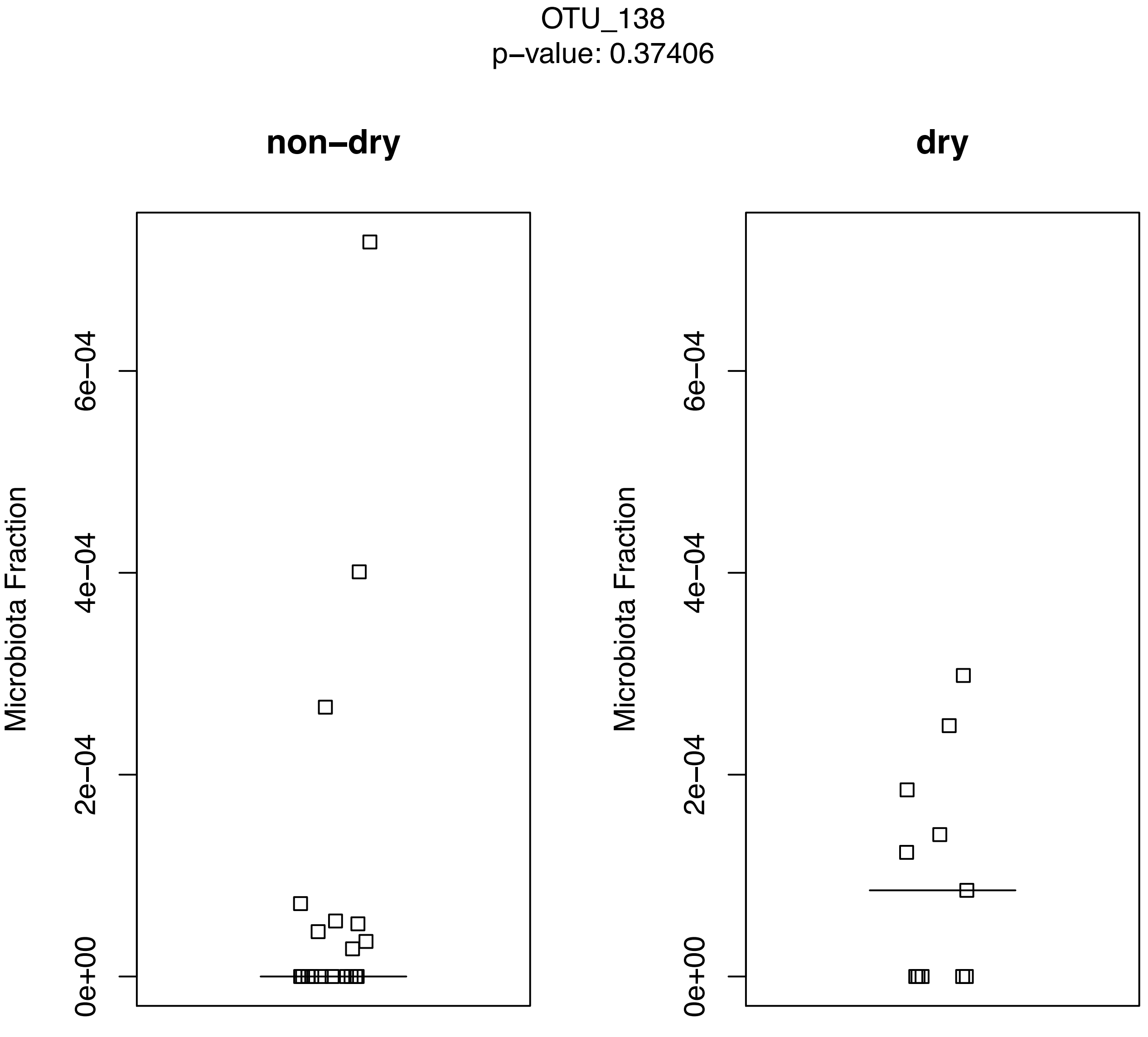

OTU_138
p−value: 0.37406
non−dry
dry
Microbiota Fraction
0e+00
2e−04
4e−04
6e−04
Microbiota Fraction
0e+00
2e−04
4e−04
6e−04

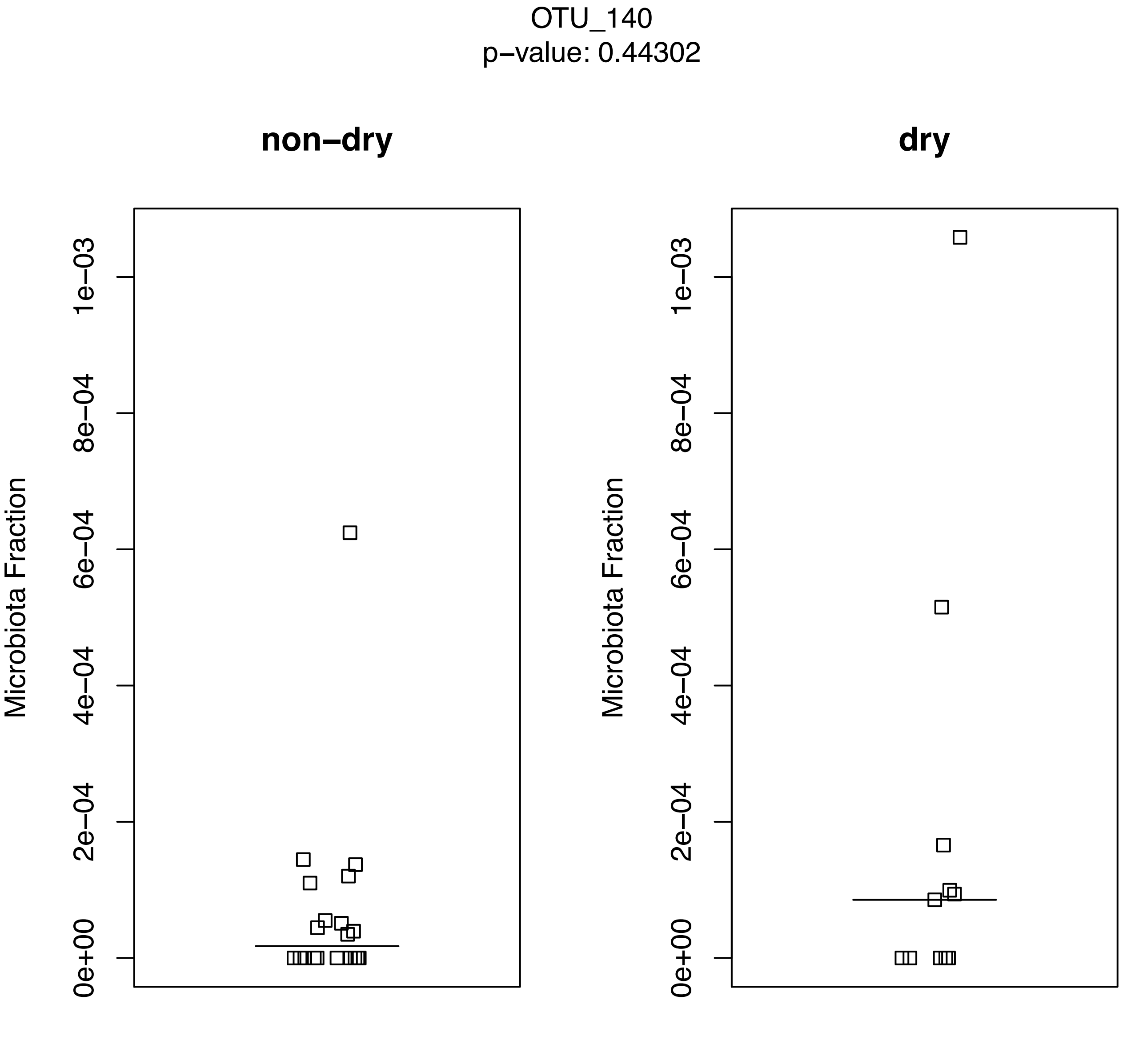

OTU_140
p−value: 0.44302
non−dry
dry
Microbiota Fraction
0e+00
2e−04
4e−04
6e−04
8e−04
1e−03

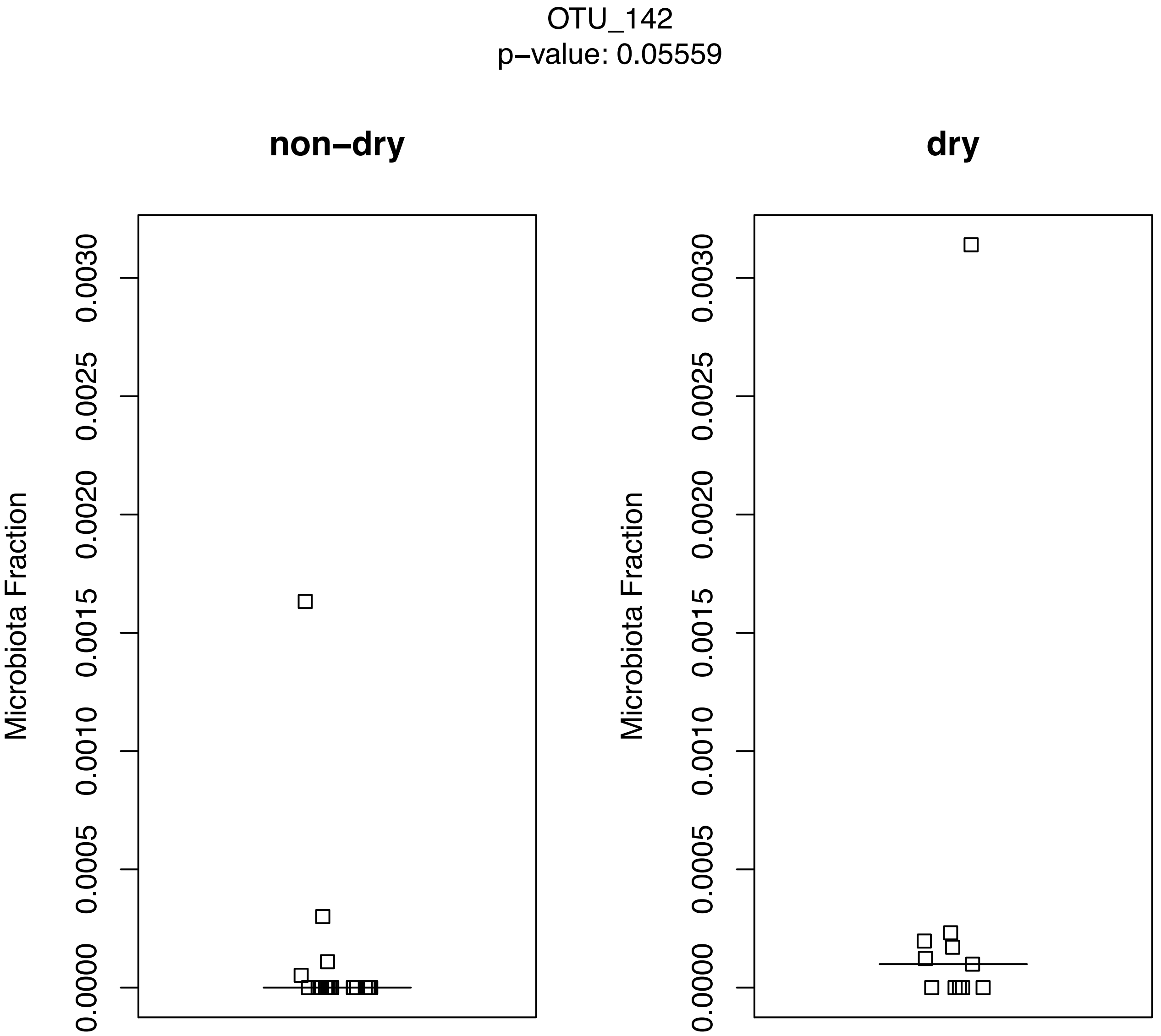
OTU_142
p−value: 0.05559
non−dry
dry
Microbiota Fraction
0.0000
0.0005
0.0010
0.0015
0.0020
0.0025
0.0030
Microbiota Fraction
0.0000
0.0005
0.0010
0.0015
0.0020
0.0025
0.0030

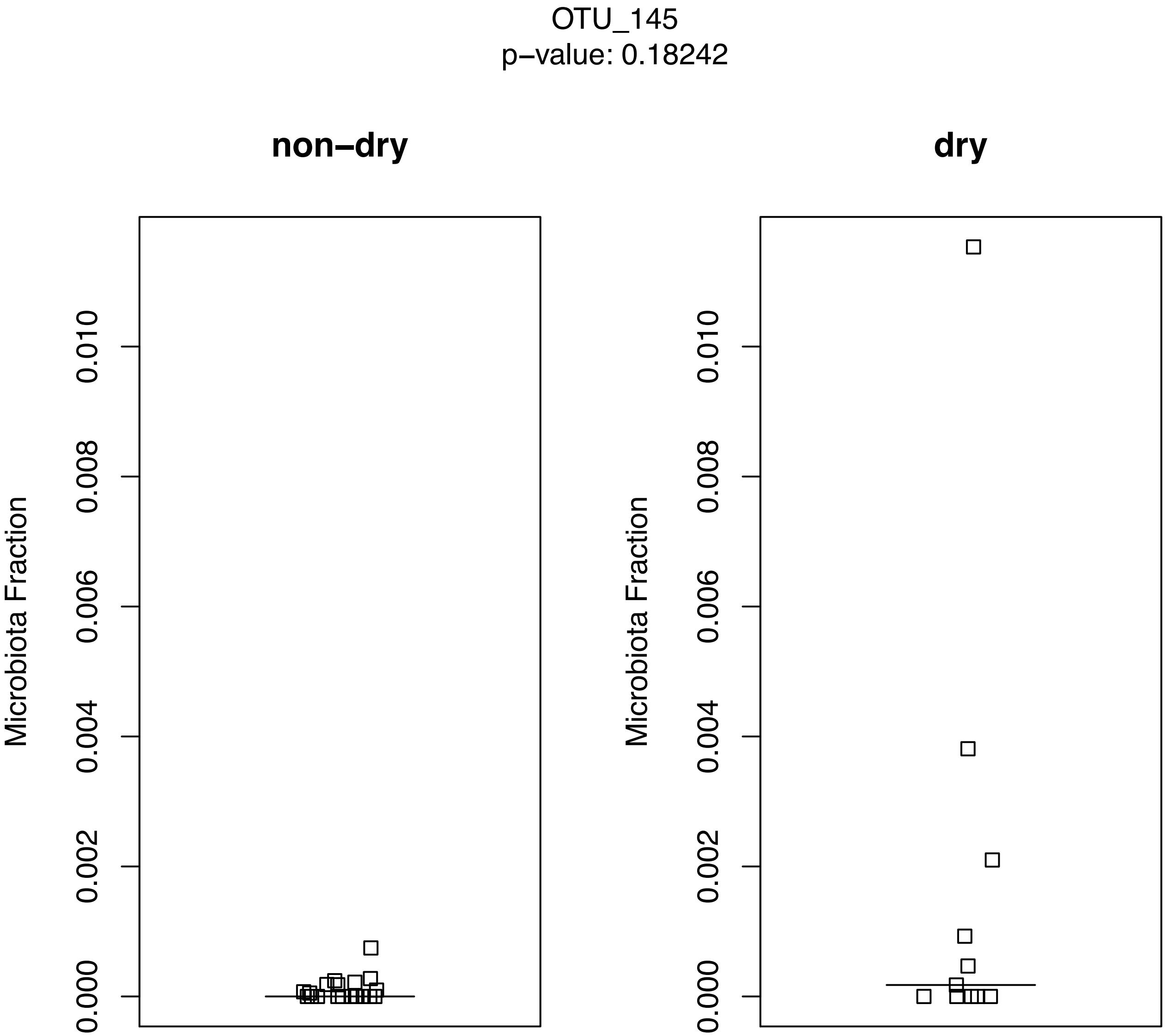

OTU_145
p−value: 0.18242
non−dry
dry
Microbiota Fraction
0.000
0.002
0.004
0.006
0.008
0.010

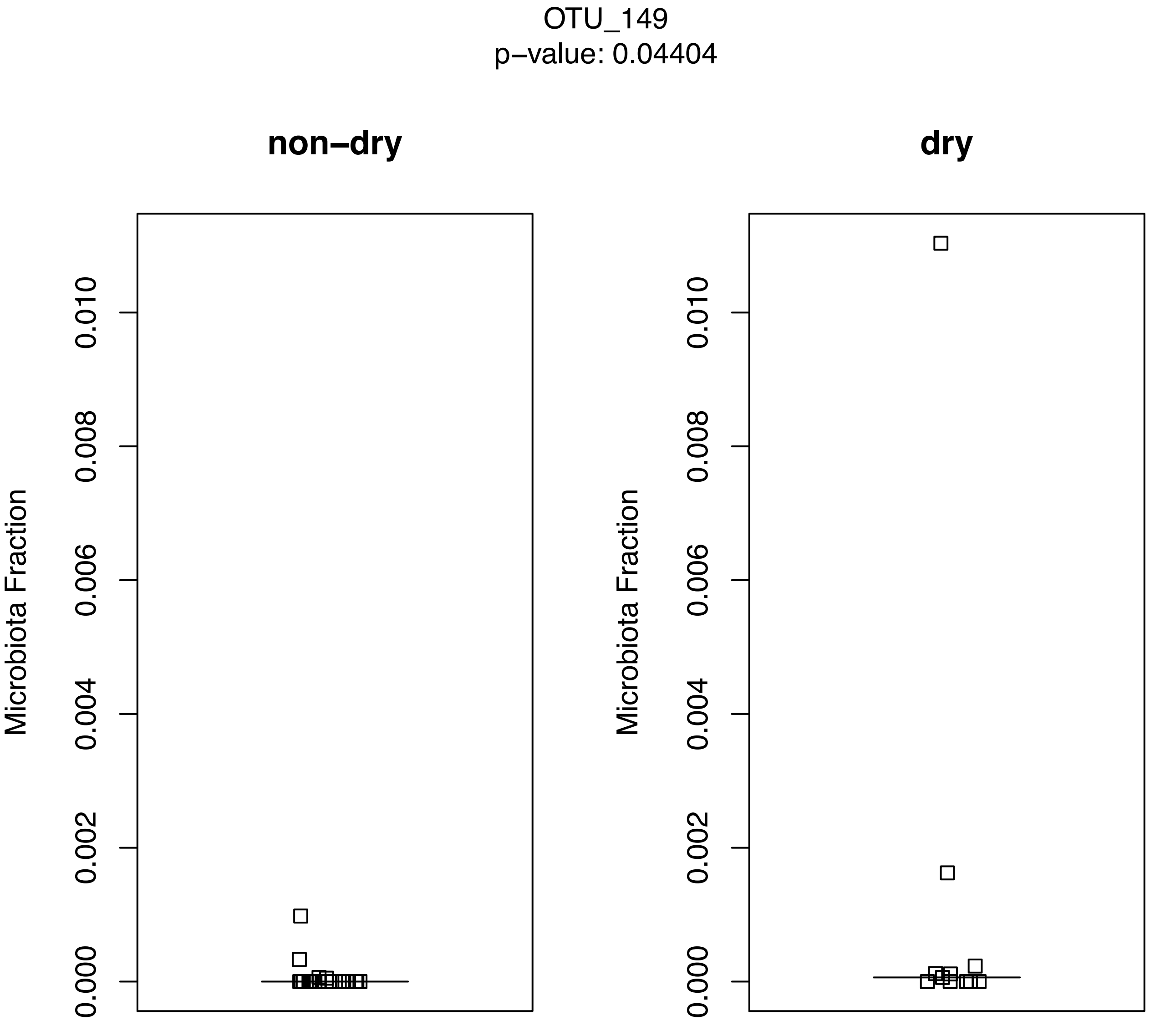

OTU_149
p−value: 0.04404
non−dry
dry
Microbiota Fraction
0.000
0.002
0.004
0.006
0.008
0.010
Microbiota Fraction
0.000
0.002
0.004
0.006
0.008
0.010

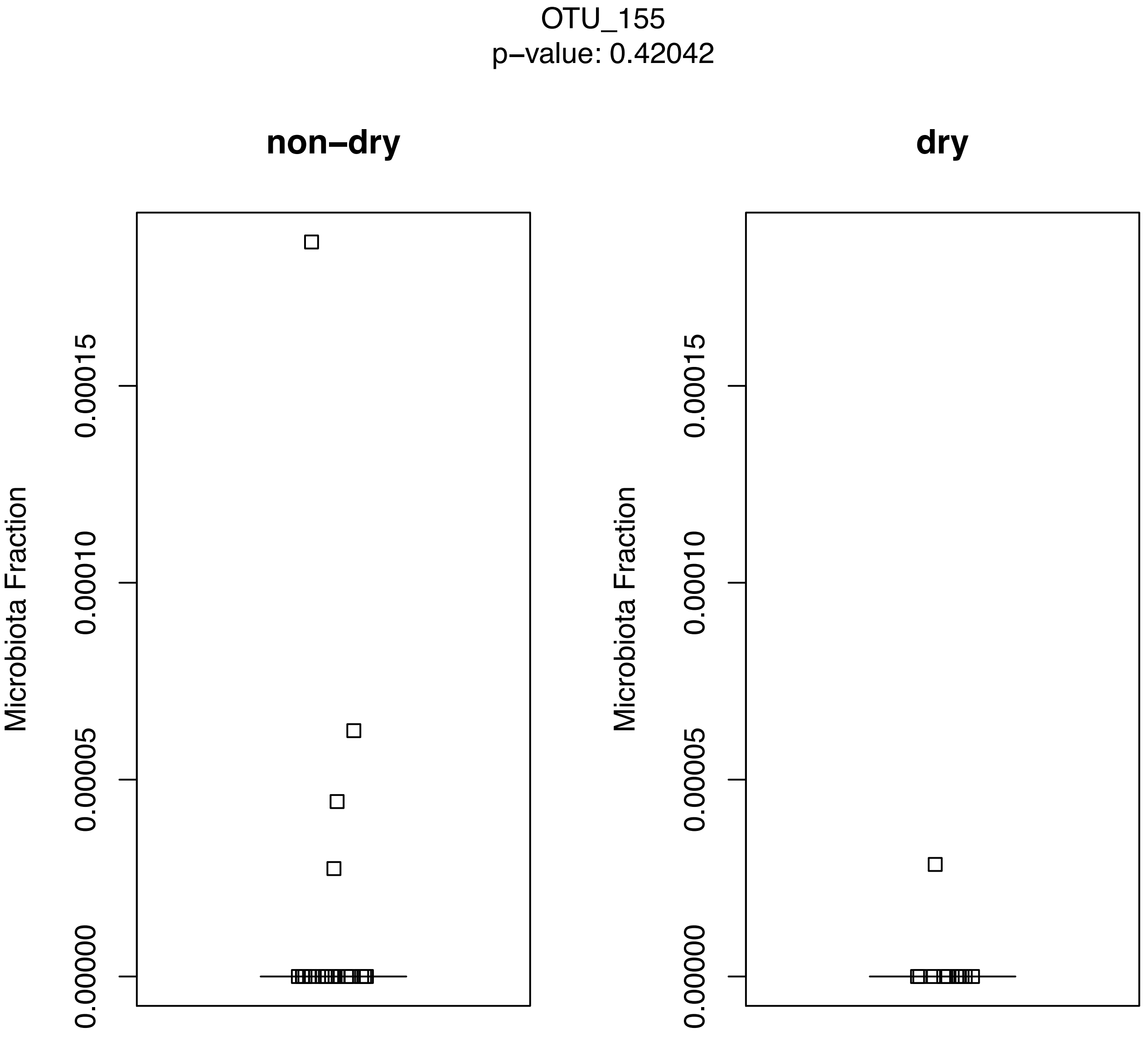
OTU_155
p−value: 0.42042
non−dry
dry
Microbiota Fraction
0.00000
0.00005
0.00010
0.00015
Microbiota Fraction
0.00000
0.00005
0.00010
0.00015

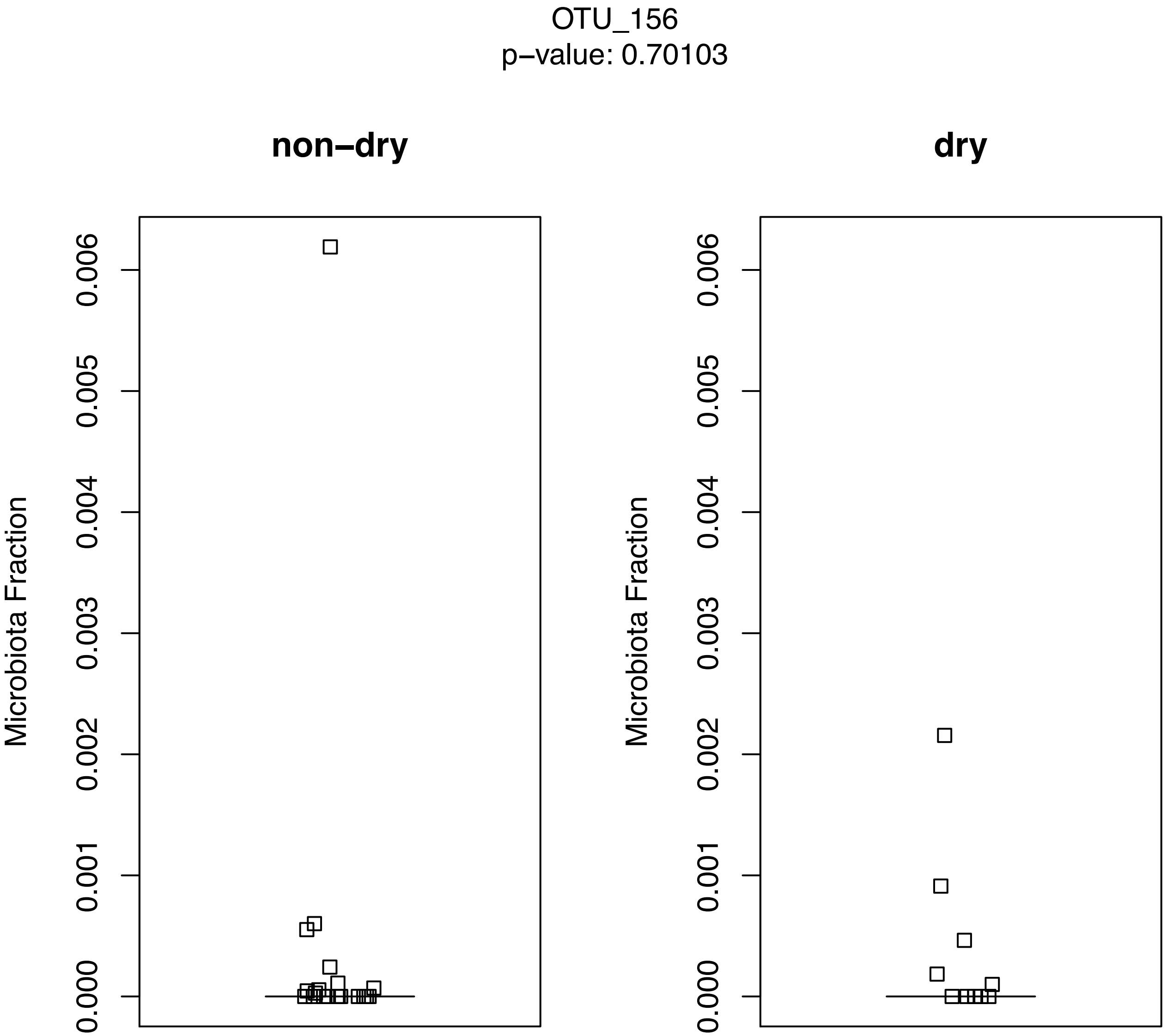

OTU_156
p−value: 0.70103
non−dry
dry
Microbiota Fraction
Microbiota Fraction
0.000
0.001
0.002
0.003
0.004
0.005
0.006

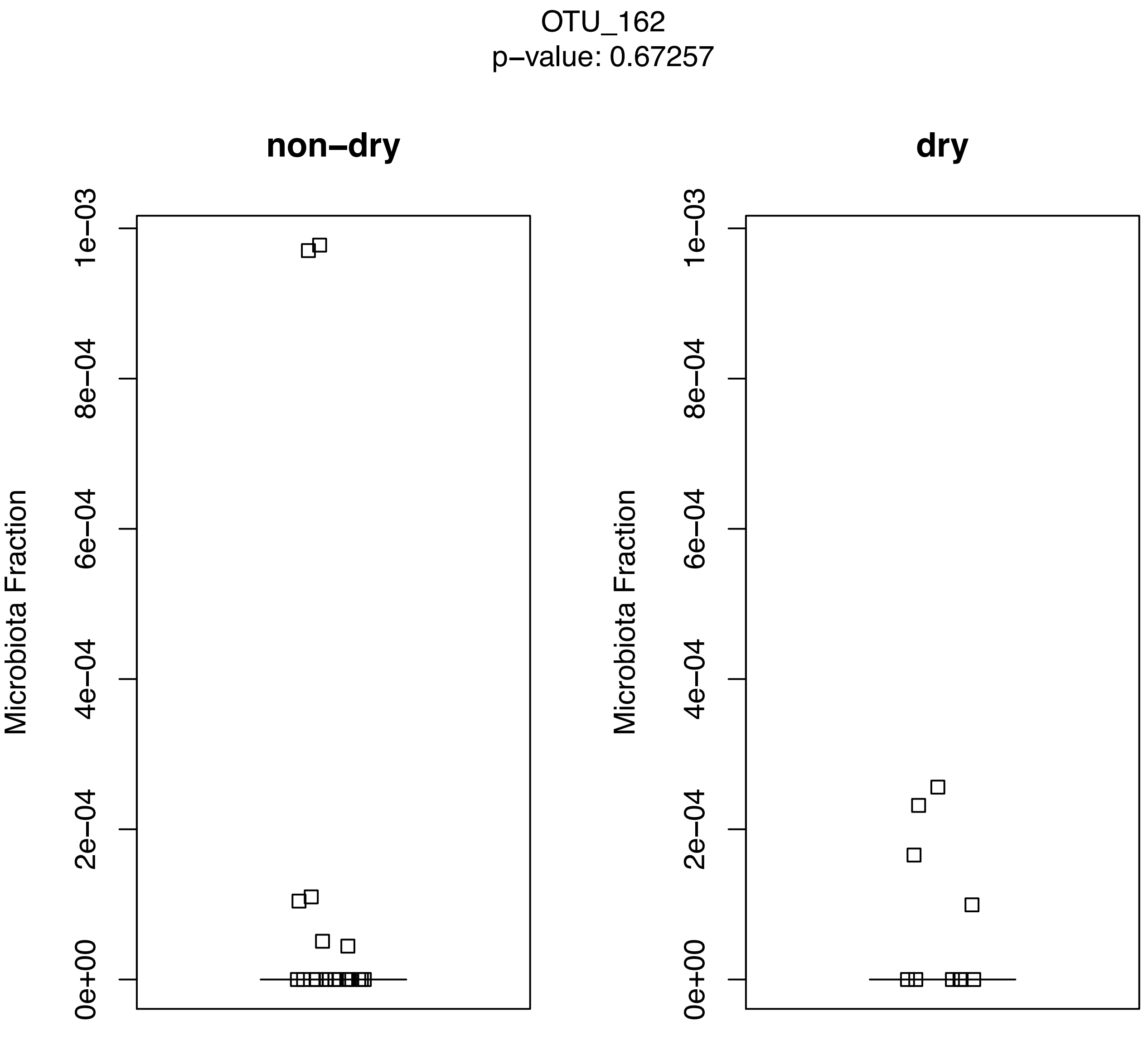

OTU_162
p−value: 0.67257
non−dry
dry
Microbiota Fraction
Microbiota Fraction
0e+00
2e−04
4e−04
6e−04
8e−04
1e−03
0e+00
2e−04
4e−04
6e−04
8e−04
1e−03

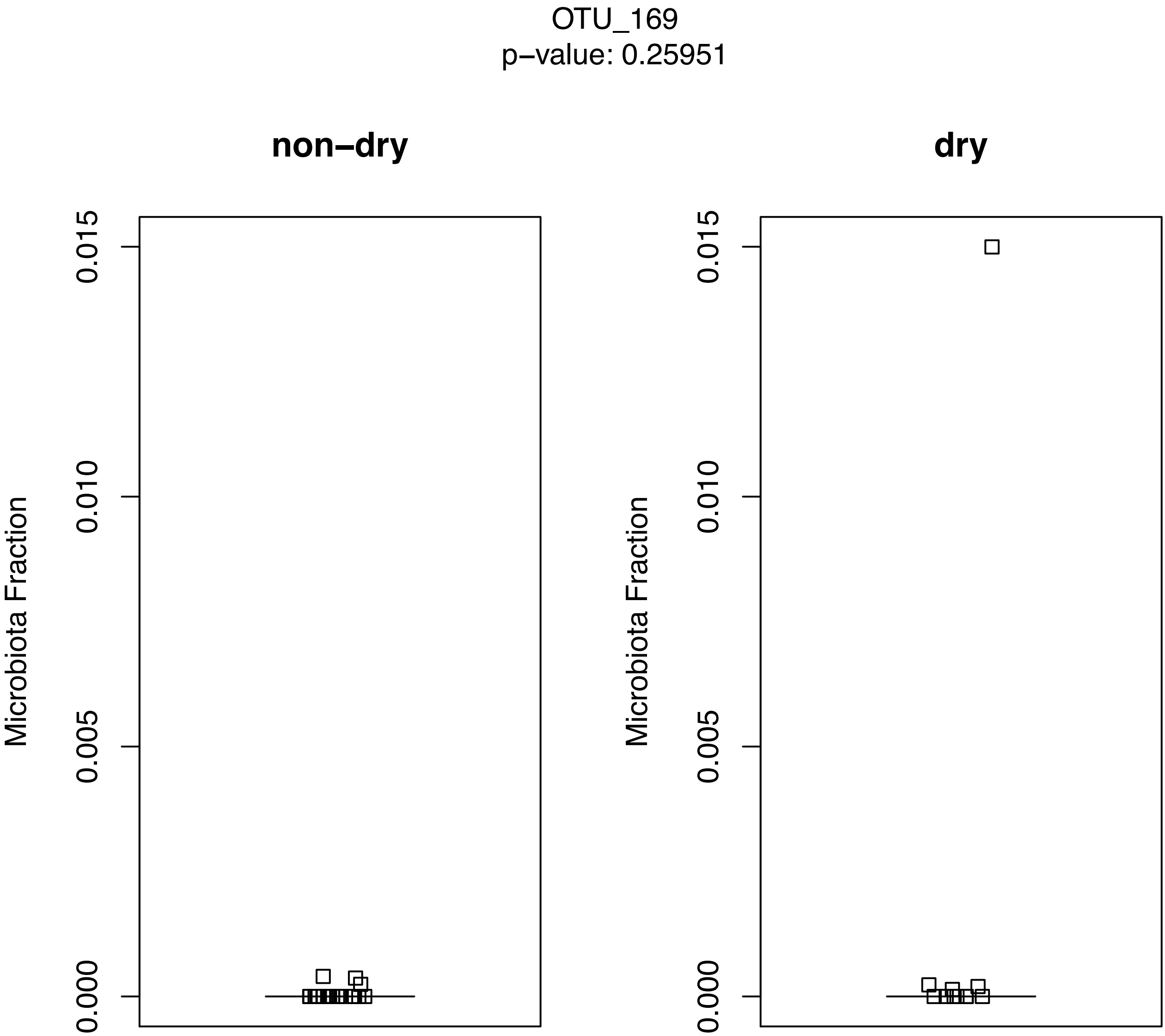

OTU_169
p−value: 0.25951
non−dry
dry
Microbiota Fraction
Microbiota Fraction
0.000
0.005
0.010
0.015
0.000
0.005
0.010
0.015

OTU_175
p−value: 0.56341

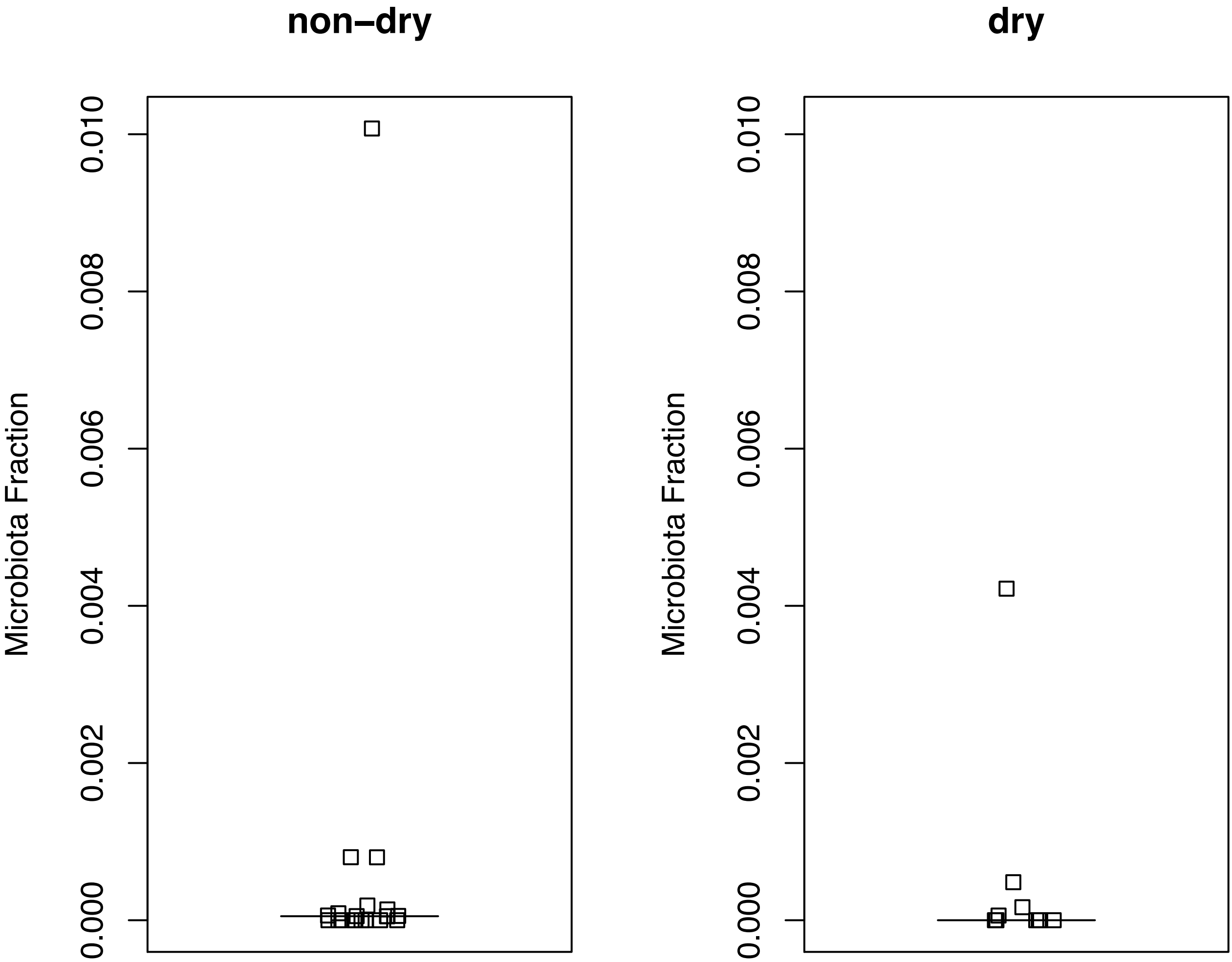

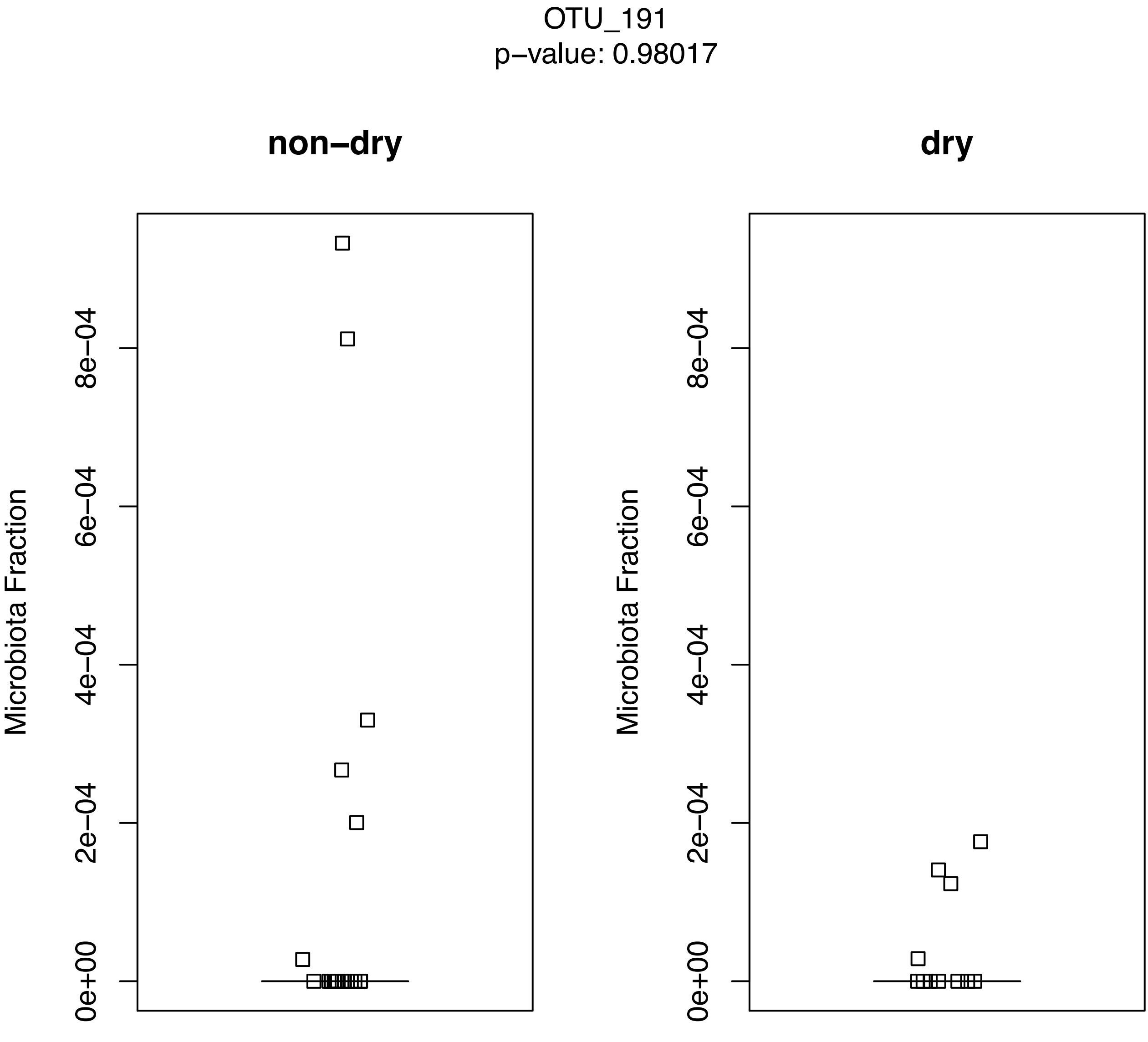
OTU_191
p−value: 0.98017
non−dry
dry
Microbiota Fraction
0e+00
2e−04
4e−04
6e−04
8e−04
Microbiota Fraction
0e+00
2e−04
4e−04
6e−04
8e−04

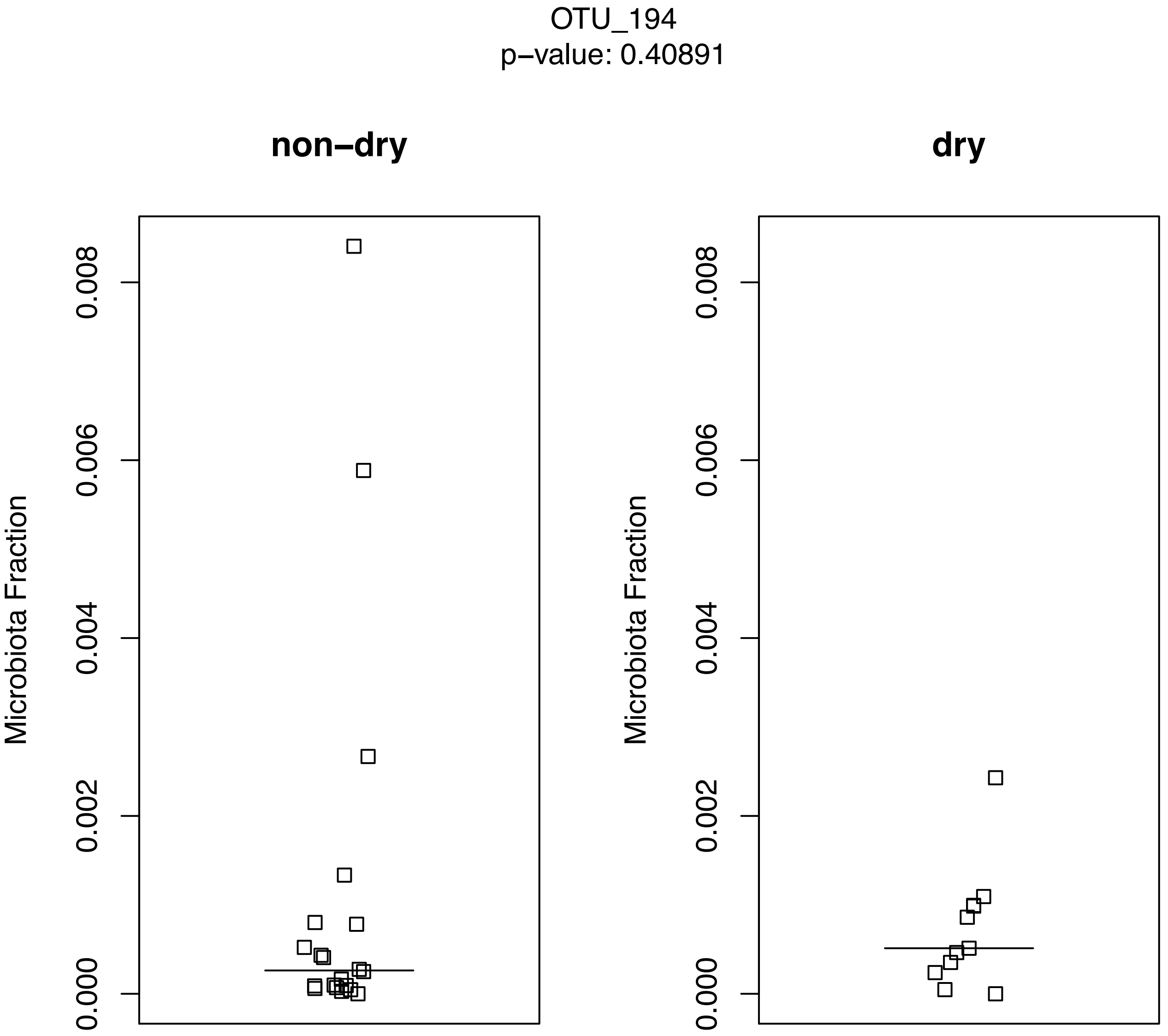
OTU_194
p−value: 0.40891
non−dry
dry
Microbiota Fraction
Microbiota Fraction
0.000
0.002
0.004
0.006
0.008
0.000
0.002
0.004
0.006
0.008

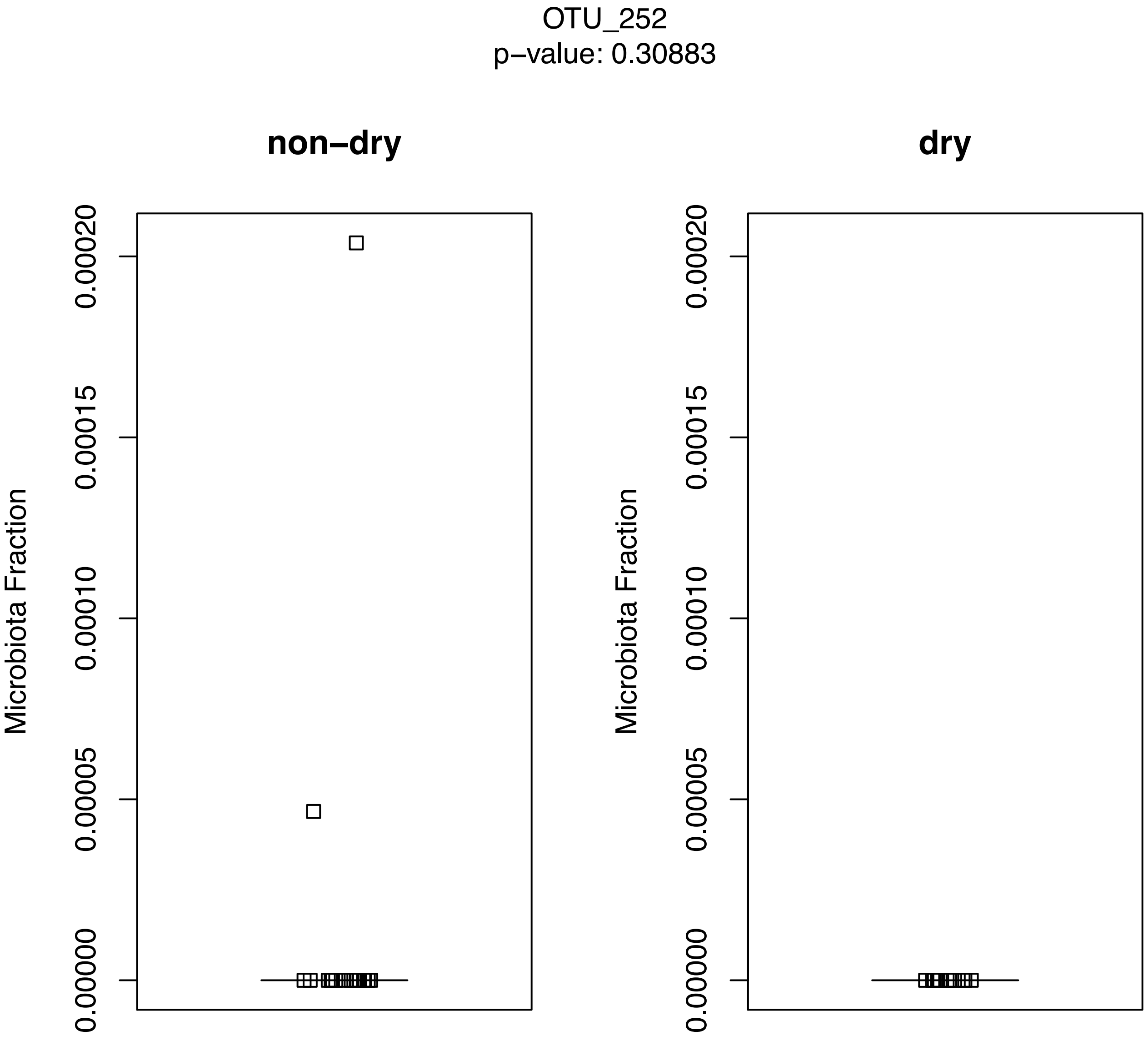
OTU_252
p−value: 0.30883
non−dry
dry
Microbiota Fraction
0.00000
0.00005
0.00010
0.00015
0.00020

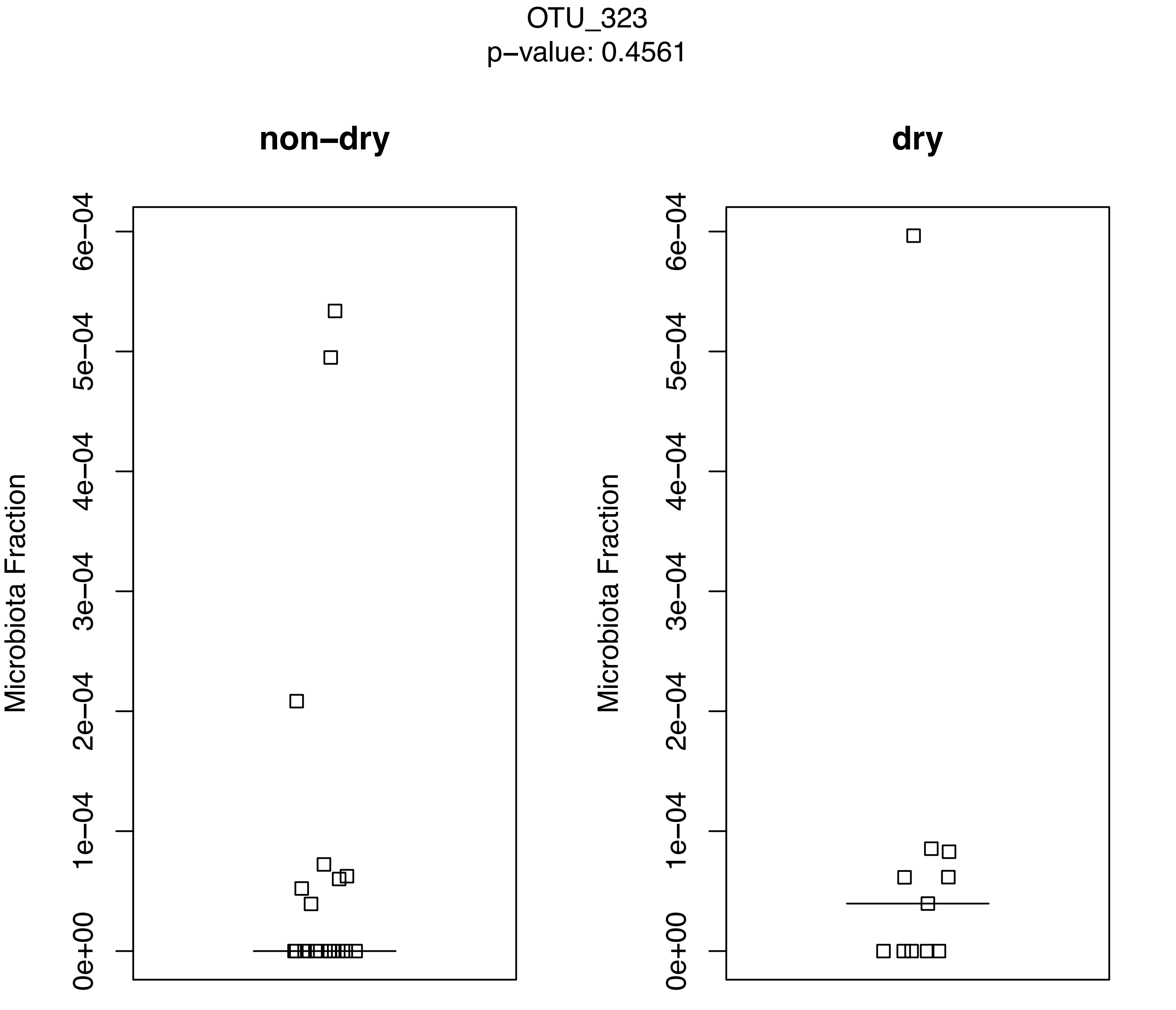

OTU_323
p–value: 0.4561
non–dry
dry
Microbiota Fraction
Microbiota Fraction
0e+00
1e−04
2e−04
3e−04
4e−04
5e−04
6e−04
0e+00
1e−04
2e−04
3e−04
4e−04
5e−04
6e−04

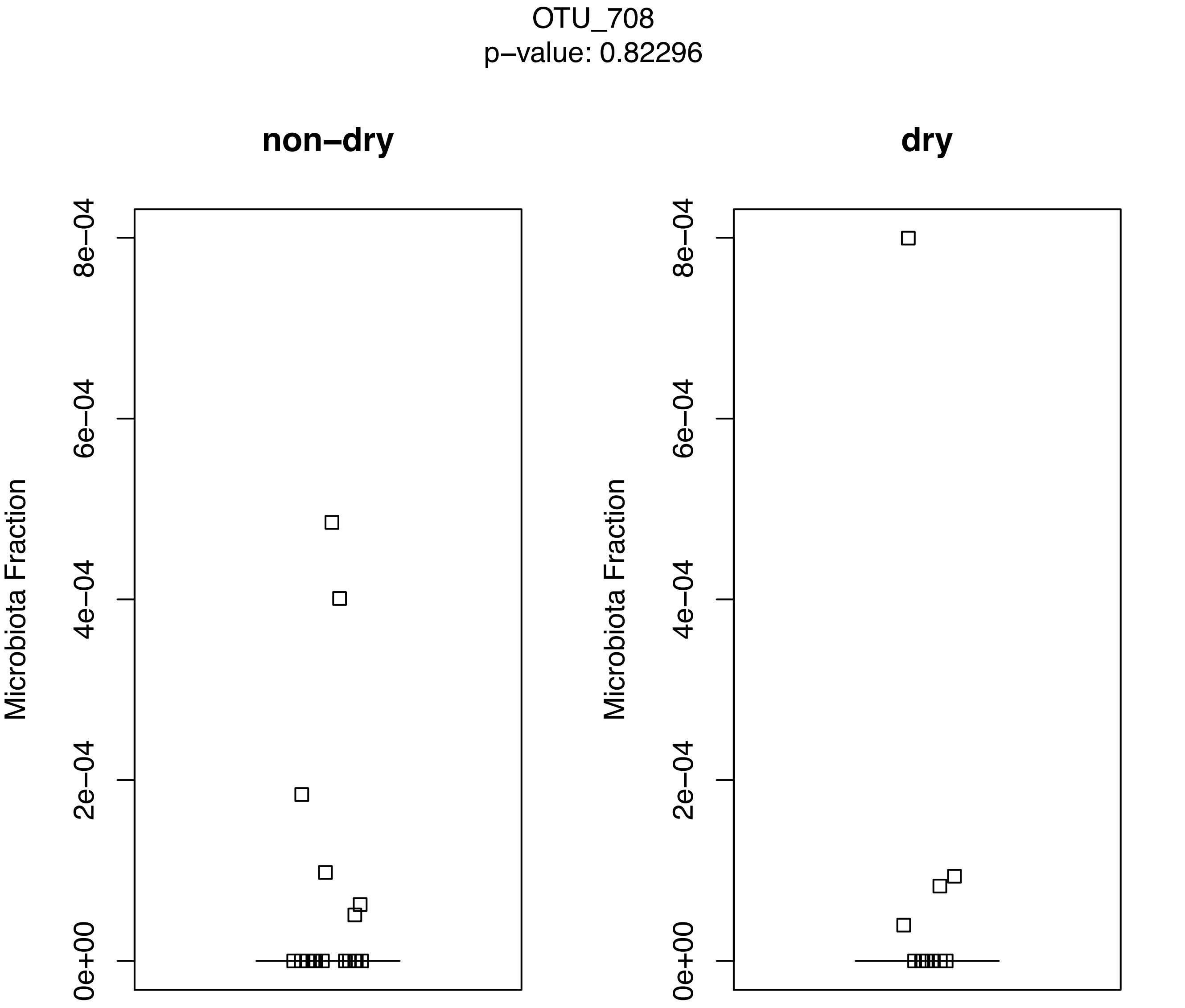

OTU_708
p−value: 0.82296
non−dry
dry
Microbiota Fraction
0e+00
2e−04
4e−04
6e−04
8e−04

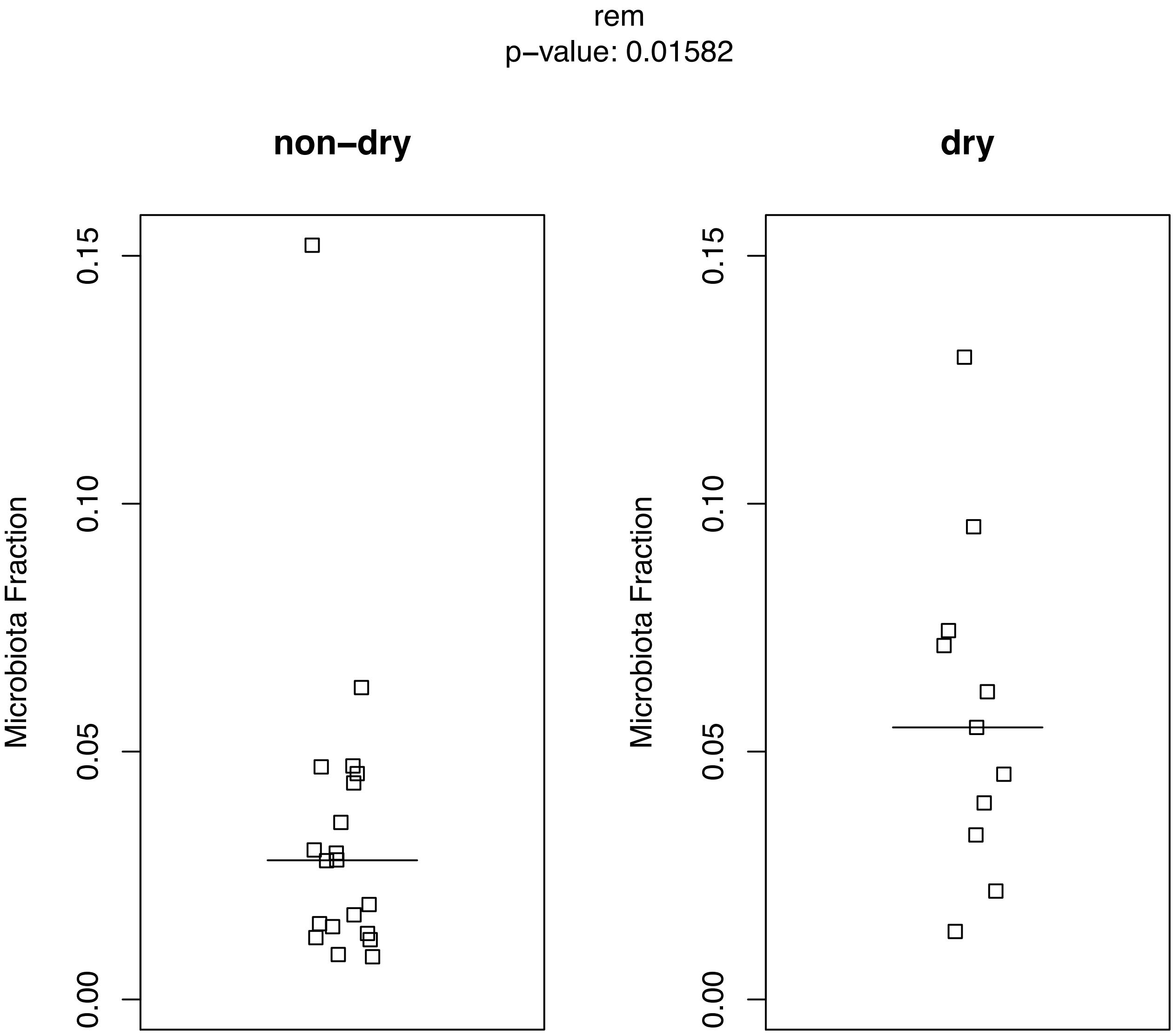
rem
p−value: 0.01582
non−dry
dry
Microbiota Fraction
Microbiota Fraction
0.00
0.05
0.10
0.15
0.00
0.05
0.10
0.15
