# Supplementary material for: Vaginal Microbiome and Epithelial Gene Array in Post-Menopausal Women with Moderate to Severe Dryness
Source: PLoS One. 2011 Nov 2;6(11):e26602. doi: 10.1371/journal.pone.0026602 (PMC3206802; doi:10.1371/journal.pone.0026602)
Supplement: Table S1 — Taxonomic assignments to OTUs at ≥1% relative abundance in any one sample. Assignments were made using the Greengenes database [32] and manually validated by BLAST [39] to the NCBI non-redundant database. (PDF) [file pone.0026602.s003.pdf]

sequence_id	greengenes prokMSA_id	greengenes Simrank id	greengenes DNAML id	greengenes DNAML columns	greengenes tax string	final name
OTU_0	103092	66.67	0.999993	29	k_Bacteria; p_Firmicutes; c_Bacilli; o_Lactobacillales; f_Lactobacillaceae; g_Lactobacillus; Unclassified; otu_1865	Lactobacillus iners
OTU_1	21096	100	0.999993	29	k_Bacteria; p_Firmicutes; c_Bacilli; o_Lactobacillales; f_Lactobacillaceae; g_Lactobacillus; s_Lactobacillus crispatus; otu_1873	Lactobacillus crispatus
OTU_2	111763	70.59	0.927641	29	k_Bacteria; p_Actinobacteria; c_Actinobacteria (class); o_Solirubrobacterales; f_Solirubrobacteraceae; Unclassified; otu_946	Gardnerella vaginalis
OTU_3	133861	100	0.999993	29	k_Bacteria; p_Actinobacteria; c_Actinobacteria (class); o_Coriobacteriales; f_Coriobacteriaceae; g_Atopobium; s_Atopobium vaginae; otu_921	Atopobium vaginae
OTU_4	55977	100	0.999993	29	k_Bacteria; p_Firmicutes; c_Bacilli; o_Lactobacillales; f_Streptococcaceae; g_Streptococcus; s_Streptococcus agalactiae; otu_1950	Streptococcus
OTU_5	265038	98.55	0.962218	28	k_Bacteria; p_Firmicutes; c_Bacilli; o_Lactobacillales; f_Lactobacillaceae; g_Lactobacillus; s_Lactobacillus jensenii; otu_1889	Lactobacillus jensenii
OTU_6	83331	100	0.999993	29	k_Bacteria; p_Bacteroidetes; c_Bacteroidia; o_Bacteroidales; f_Prevotellaceae; g_Prevotella; Unclassified; otu_1034	Prevotella timonensis
OTU_7	250493	100	0.999993	29	k_Bacteria; p_Bacteroidetes; c_Bacteroidia; o_Bacteroidales; f_Prevotellaceae; g_Prevotella; Unclassified; otu_1034	Prevotella bivia
OTU_8	103092	100	0.999993	29	k_Bacteria; p_Firmicutes; c_Bacilli; o_Lactobacillales; f_Lactobacillaceae; g_Lactobacillus; Unclassified; otu_1865	Lactobacillus gasseri/johnsonii
OTU_9	264562	36.99	0.677622	29	k_Bacteria; p_Bacteroidetes; c_Flavobacteria; o_Flavobacteriales; f_Flavobacteriaceae; g_Flavobacterium; s_Flavobacterium succinicans; otu_1109	Porphyromonas
OTU_10	161818	74.65	0.999993	29	k_Bacteria; p_Firmicutes; c_Clostridia; o_Clostridiales; f_Veillonellaceae; g_Megasphaera; Unclassified; otu_2216	Veillonellaceae dialister/megasphera
OTU_11	15488	89.86	0.999993	29	k_Bacteria; p_Firmicutes; c_Bacilli; o_Lactobacillales; f_Streptococcaceae; g_Streptococcus; s_Streptococcus anginosus; otu_1952	Streptococcus anginosus
OTU_12	161654	100	0.999993	29	k_Bacteria; p_Firmicutes; c_Bacilli; o_Lactobacillales; f_Lactobacillaceae; g_Lactobacillus; s_Lactobacillus delbrueckii; otu_1876	Lactobacillus delbrueckii spp. bulgaricus
OTU_13	15851	63.08	0.841453	28	k_Bacteria; p_Tenericutes; c_Mollicutes; o_Mycoplasmatales; f_Mycoplasmataceae; g_Mycoplasma; Unclassified; otu_4272	Mycoplasma
OTU_14	102801	49.28	0.926885	29	k_Bacteria; p_Firmicutes; c_Clostridia; o_Clostridiales; f_Clostridiaceae; g_Caminicella; otu_2004	Clostridiales BVAB1
OTU_15	33338	72.06	0.999993	29	k_Bacteria; p_Bacteroidetes; c_Bacteroidia; o_Bacteroidales; f_Porphyromonadaceae; g_Porphyromonas; s_Porphyromonas gingivalis; otu_1029	Porphyromonas
OTU_16	70784	52.17	0.795612	29	k_Bacteria; p_Proteobacteria; c_Gammaproteobacteria; o_Oceanospirillales; f_Halomonadaceae; g_Halomonas; s_Halomonas muralis; otu_3763	Leptotrichia amnionii
OTU_17	15072	100	0.999993	29	k_Bacteria; p_Firmicutes; c_Bacilli; o_Lactobacillales; f_Aerococcaceae; g_Aerococcus; s_Aerococcus urinae; otu_1810	Aerococcus urinae
OTU_18	11384	100	0.963831	29	k_Bacteria; p_Actinobacteria; c_Actinobacteria (class); o_Coriobacteriales; f_Coriobacteriaceae; g_Atopobium; s_Atopobium rimae; otu_920	Atopobium rimae
OTU_19	176207	57.35	0.962741	29	k_Bacteria; p_Firmicutes; c_Bacilli; o_Bacillales; f_Bacillaceae; g_Bacillus; Unclassified; otu_1570	Clostridiales BVAB2
OTU_20	100589	48.1	0.963962	29	k_Bacteria; p_Actinobacteria; c_Actinobacteria (class); o_Actinomycetales; f_Corynebacteriaceae; g_Corynebacterium; s_Brevibacterium stationis; otu_341	Corynebacterium
OTU_21	291490	56.52	0.999993	29	k_Bacteria; p_Bacteroidetes; c_Bacteroidia; o_Bacteroidales; f_Prevotellaceae; g_Prevotella; Unclassified; otu_1034	Prevotella amnii
OTU_22	249686	100	0.999993	29	k_Bacteria; p_Firmicutes; c_Bacilli; o_Lactobacillales; f_Lactobacillaceae; g_Lactobacillus; s_Lactobacillus acidophilus; otu_1867	Lactobacillus acidophilus
OTU_23	14284	82.19	0.999993	29	k_Bacteria; p_Firmicutes; c_Clostridia; o_Clostridiales; f_Clostridiales Family XI. Incertae Sedis; g_Anaerococcus; Unclassified; otu_2044	Anaerococcus
OTU_24	292080	98.55	0.925635	28	k_Bacteria; p_Firmicutes; c_Bacilli; o_Lactobacillales; f_Lactobacillaceae; g_Lactobacillus; Unclassified; otu_1865	Lactobacillus vaginalis
OTU_25	110646	50.72	0.847761	29	k_Bacteria; p_Firmicutes; c_Clostridia; o_Clostridiales; f_Heliobacteriaceae; g_Heliobacterium; Unclassified; otu_2084	Sneathia
OTU_26	69910	67.65	0.928004	29	k_Bacteria; p_Bacteroidetes; c_Bacteroidia; o_Bacteroidales; f_Prevotellaceae; g_Prevotella; Unclassified; otu_1034	Prevotella disiens
OTU_27	183158	100	0.963962	29	k_Bacteria; p_Firmicutes; c_Clostridia; o_Clostridiales; f_Clostridiales Family XI. Incertae Sedis; g_Peptoniphilus; s_Peptoniphilus asaccharolyticus; otu_2055	Peptoniphilus
OTU_28	109876	100	0.999993	29	k_Bacteria; p_Firmicutes; c_Clostridia; o_Clostridiales; f_Veillonellaceae; g_Dialister; s_Dialister micraerophilus; otu_2212	Dialister microaerophilus
OTU_29	45427	76.47	0.999993	29	k_Bacteria; p_Actinobacteria; c_Actinobacteria (class); o_Actinomycetales; f_Actinomycetaceae; g_Actinomyces; s_Actinomyces odontolyticus; otu_286	Actinomyces odontolyticus
OTU_30	9676	100	0.999993	29	k_Bacteria; p_Proteobacteria; c_Gammaproteobacteria; o_Enterobacteriales; f_Enterobacteriaceae; Unclassified; otu_3529	Enterobacteriaceae
OTU_31	288039	100	0.999993	29	k_Bacteria; p_Firmicutes; c_Clostridia; o_Clostridiales; f_Clostridiaceae; Unclassified; otu_1999	Clostridiaceae
OTU_32	107851	100	0.999993	29	k_Bacteria; p_Firmicutes; c_Bacilli; o_Lactobacillales; f_Lactobacillaceae; g_Lactobacillus; Unclassified; otu_1865	Lactobacillus rhamnosus/casei
OTU_33	109501	70.42	0.964936	29	k_Bacteria; p_Firmicutes; c_Clostridia; o_Clostridiales; f_Veillonellaceae; g_Dialister; s_Dialister micraerophilus; otu_2212	Dialister
OTU_34	253383	42.47	0.793475	29	k_Bacteria; p_Firmicutes; c_Bacilli; o_Bacillales; f_Bacillaceae; g_Bacillus; s_Bacillus agaradhaerens; otu_1572	Bacillus
OTU_35	140250	100	0.999993	29	k_Bacteria; p_Actinobacteria; c_Actinobacteria (class); o_Actinomycetales; f_Actinomycetaceae; g_Actinomyces; s_Actinomyces neuii; otu_285	Actinomyces neuii
OTU_36	105160	100	0.999993	29	k_Bacteria; p_Firmicutes; c_Bacilli; o_Bacillales; f_Staphylococcaceae; g_Staphylococcus; Unclassified; otu_1772	Staphylococcus
OTU_37	107871	100	0.999993	29	k_Bacteria; p_Firmicutes; c_Bacilli; o_Lactobacillales; f_Lactobacillaceae; g_Lactobacillus; s_Lactobacillus fermentum; otu_1879	Lactobacillus fermentum
OTU_38	81082	100	0.963962	29	k_Bacteria; p_Firmicutes; c_Clostridia; o_Clostridiales; f_Clostridiales Family XI. Incertae Sedis; g_Finegoldia; Unclassified; otu_2049	Finegoldia magna
OTU_39	158230	68.12	0.843924	29	k_Bacteria; p_Firmicutes; c_Clostridia; o_Clostridiales; f_Ruminococcaceae; Unclassified; otu_2159	Peprostreptococcus
OTU_40	111568	100	0.999993	29	k_Bacteria; p_Bacteroidetes; c_Bacteroidia; o_Bacteroidales; f_Prevotellaceae; g_Prevotella; s_Prevotella histicola; otu_1039	Prevotella
OTU_41	109997	85.07	0.999993	29	k_Bacteria; p_Actinobacteria; c_Actinobacteria (class); o_Actinomycetales; f_Actinomycetaceae; g_Varibaculum; s_Varibaculum cambriense; otu_298	Varibaculum
OTU_42	83408	100	0.999993	29	k_Bacteria; p_Firmicutes; c_Clostridia; o_Clostridiales; f_Clostridiales Family XI. Incertae Sedis; g_; s_Parvimonas micra; otu_2043	Parvimonas micra
OTU_43	15369	100	0.999993	29	k_Bacteria; p_Firmicutes; c_Bacilli; o_Lactobacillales; f_Streptococcaceae; g_Streptococcus; Unclassified; otu_1949	Streptococcus
OTU_44	175062	100	0.999993	29	k_Bacteria; p_Firmicutes; c_Clostridia; o_Clostridiales; f_Clostridiales Family XI. Incertae Sedis; g_Anaerococcus; s_Anaerococcus hydrogenalis; otu_2045	Anaerococcus
OTU_45	44642	43.84	0.926725	29	k_Bacteria; p_Firmicutes; c_Bacilli; o_Lactobacillales; f_Leuconostocaceae; g_Oenococcus; s_Oenococcus oeni; otu_1936	OTU_45
OTU_46	2196	89.71	0.999993	29	k_Bacteria; p_Bacteroidetes; c_Bacteroidia; o_Bacteroidales; f_Prevotellaceae; g_Prevotella; Unclassified; otu_1034	Prevotella
OTU_47	107784	76.81	0.999993	29	k_Bacteria; p_Firmicutes; c_Bacilli; o_Lactobacillales; f_Lactobacillaceae; g_Lactobacillus; Unclassified; otu_1865	Lactobacillus
OTU_48	192814	59.7	0.928004	29	k_Bacteria; p_Bacteroidetes; c_Bacteroidia; o_Bacteroidales; f_Prevotellaceae; g_Prevotella; Unclassified; otu_1034	Prevotella
OTU_49	85281	100	0.999993	29	k_Bacteria; p_Proteobacteria; c_Gammaproteobacteria; o_Enterobacteriales; f_Enterobacteriaceae; Unclassified; otu_3529	Enterobacteriaceae
OTU_50	236238	74.63	0.963991	29	k_Bacteria; p_Actinobacteria; c_Actinobacteria (class); o_Actinomycetales; f_Corynebacteriaceae; g_Corynebacterium; s_Corynebacterium freneyi; otu_353	Corynebacterium
OTU_51	15555	100	0.999993	29	k_Bacteria; p_Firmicutes; c_Bacilli; o_Lactobacillales; f_Streptococcaceae; g_Streptococcus; Unclassified; otu_1949	Streptococcus
OTU_52	71061	68.66	0.926667	29	k_Bacteria; p_Actinobacteria; c_Actinobacteria (class); o_Actinomycetales; f_Corynebacteriaceae; g_Corynebacterium; Unclassified; otu_340	Corynebacterium
OTU_53	76547	60.29	0.926885	29	k_Bacteria; p_Firmicutes; c_Clostridia; o_Clostridiales; f_Clostridiales Family XIII. Incertae Sedis; Unclassified; otu_2066	OTU_53
OTU_54	210736	37.14	0.648903	29	k_Bacteria; p_Firmicutes; c_Clostridia; o_Clostridiales; f_Peptostreptococcaceae; g_Tepidibacter; otu_2158	OTU_54
OTU_55	290474	86.96	0.926958	29	k_Bacteria; p_Firmicutes; c_Clostridia; o_Clostridiales; f_Lachnospiraceae; Unclassified; otu_2087	OTU_55
OTU_56	198874	72.6	0.925868	29	k_Bacteria; p_Firmicutes; c_Clostridia; o_Clostridiales; f_Clostridiales Family XI. Incertae Sedis; g_Anaerococcus; Unclassified; otu_2044	Anaerococcus
OTU_57	22422	53.62	0.924066	28	k_Bacteria; p_Tenericutes; c_Erysipelotrichi; o_Erysipelotrichales; f_Erysipelotrichaceae; g_Bulleidia; s_Solobacterium moorei; otu_4200	Clostridiales BVAB3
OTU_58	217912	100	0.964471	29	k_Bacteria; p_Firmicutes; c_Bacilli; o_Bacillales; f_Staphylococcaceae; g_Staphylococcus; Unclassified; otu_1772	Staphylococcus
OTU_59	69866	100	0.999993	29	k_Bacteria; p_Actinobacteria; c_Actinobacteria (class); o_Actinomycetales; f_Corynebacteriaceae; g_Corynebacterium; s_Corynebacterium riegelii; otu_366	Corynebacterium
OTU_60	217968	83.1	0.883064	28	k_Bacteria; p_Proteobacteria; c_Alphaproteobacteria; o_Rhodospirillales; f_Acetobacteraceae; g_Roseomonas; Unclassified; otu_2786	Roseomonas
OTU_61	78008	100	0.999993	29	k_Bacteria; p_Actinobacteria; c_Actinobacteria (class); o_Actinomycetales; f_Actinomycetaceae; g_Mobiluncus; s_Mobiluncus curtisii; otu_295	Mobiluncus curtisii
OTU_62	137182	100	0.999993	29	k_Bacteria; p_Firmicutes; c_Clostridia; o_Clostridiales; f_Clostridiales Family XI. Incertae Sedis; g_Anaerococcus; Unclassified; otu_2044	Anaerococcus
OTU_63	106977	100	0.999993	29	k_Bacteria; p_Firmicutes; c_Bacilli; o_Lactobacillales; f_Lactobacillaceae; g_Lactobacillus; Unclassified; otu_1865	Lactobacillus plantarum
OTU_64	111587	100	0.999993	28	k_Bacteria; p_Actinobacteria; c_Actinobacteria (class); o_Bifidobacteriales; f_Bifidobacteriaceae; g_Alloscardovia; s_Alloscardovia omnicolens; otu_895	Alloscardovia omnicolens
OTU_65	96612	100	0.999993	29	k_Bacteria; p_Firmicutes; c_Clostridia; o_Clostridiales; f_Veillonellaceae; g_Veillonella; s_Veillonella montpellierensis; otu_2232	Veillonella montpellierensis
OTU_66	105312	100	0.963991	29	k_Bacteria; p_Actinobacteria; c_Actinobacteria (class); o_Actinomycetales; f_Mycobacteriaceae; g_Mycobacterium; Unclassified; otu_578	Mycobacterium
OTU_67	109876	75.71	0.96357	28	k_Bacteria; p_Firmicutes; c_Clostridia; o_Clostridiales; f_Veillonellaceae; g_Dialister; s_Dialister micraerophilus; otu_2212	Dialister
OTU_68	11212	100	0.999993	29	k_Bacteria; p_Proteobacteria; c_Epsilonproteobacteria; o_Campylobacterales; f_Campylobacteraceae; g_Campylobacter; s_Campylobacter hominis; otu_3326	Campylobacter
OTU_69	103712	91.67	0.999993	29	k_Bacteria; p_Fusobacteria; c_Fusobacteria (class); o_Fusobacteriales; f_Fusobacteriaceae; g_Fusobacterium; Unclassified; otu_2287	Fusobacterium
OTU_70	128363	91.18	0.886974	29	k_Bacteria; p_Actinobacteria; c_Actinobacteria (class); o_Bifidobacteriales; f_Bifidobacteriaceae; g_Gardnerella; s_Gardnerella vaginalis; otu_911	Gardnerella vaginalis
OTU_71	100234	100	0.999993	29	k_Bacteria; p_Proteobacteria; c_Gammaproteobacteria; o_Enterobacteriales; f_Enterobacteriaceae; Unclassified; otu_3529	Enterobacteriaceae
OTU_72	110305	47.37	0.926289	29	k_Bacteria; p_Actinobacteria; c_Actinobacteria (class); o_Actinomycetales; Unclassified; otu_271	OTU_72
OTU_73	108033	40.79	0.965154	29	k_Bacteria; p_Firmicutes; c_Bacilli; o_Lactobacillales; f_Streptococcaceae; g_Lactococcus; s_Lactococcus lactis; otu_1947	OTU_73
OTU_74	348164	43.94	0.607436	28	k_Bacteria; p_Proteobacteria; c_Gammaproteobacteria; o_Vibrionales; f_Vibrionaceae; g_Photobacterium; Unclassified; otu_3973	Proteobacteria
OTU_75	222105	69.12	0.999993	29	k_Bacteria; p_Actinobacteria; c_Actinobacteria (class); o_Actinomycetales; f_Corynebacteriaceae; g_Corynebacterium; Unclassified; otu_340	Corynebacterium
OTU_77	138357	79.71	0.964733	29	k_Bacteria; p_Firmicutes; c_Bacilli; o_Lactobacillales; f_Lactobacillaceae; g_Lactobacillus; s_Lactobacillus coleohominis; otu_1872	Lactobacillus
OTU_79	128356	100	0.999993	29	k_Bacteria; p_Firmicutes; c_Clostridia; o_Clostridiales; f_Clostridiales Family XI. Incertae Sedis; g_Anaerococcus; s_Anaerococcus hydrogenalis; otu_2045	Anaerococcus hydrogenalis
OTU_81	23588	100	0.999993	27	k_Bacteria; p_Firmicutes; c_Clostridia; o_Clostridiales; f_Ruminococcaceae; g_Ruminococcus; Unclassified; otu_2178	Ruminococcus
OTU_82	214782	100	0.999993	29	k_Bacteria; p_Actinobacteria; c_Actinobacteria (class); o_Bifidobacteriales; f_Bifidobacteriaceae; g_Bifidobacterium; s_Bifidobacterium bifidum; otu_899	Bifidobacterium bifidum
OTU_83	77836	100	0.999993	29	k_Bacteria; p_Proteobacteria; c_Alphaproteobacteria; o_Rhizobiales; f_Methylobacteriaceae; g_Methylobacterium; s_Methylobacterium adhaesivum; otu_2553	Methylobacterium
OTU_84	192451	78.57	0.999993	29	k_Bacteria; p_Firmicutes; c_Clostridia; o_Clostridiales; f_Clostridiales Family XI. Incertae Sedis; g_Peptoniphilus; s_Peptoniphilus asaccharolyticus; otu_2055	Peptoniphilus
OTU_85	94580	100	0.999993	29	k_Bacteria; p_Actinobacteria; c_Actinobacteria (class); o_Actinomycetales; f_Actinomycetaceae; g_Mobiluncus; s_Mobiluncus mulieris; otu_296	Mobiluncus mulieris
OTU_87	219668	76.71	0.999993	29	k_Bacteria; p_Firmicutes; c_Clostridia; o_Clostridiales; f_Clostridiales Family XI. Incertae Sedis; g_Anaerococcus; Unclassified; otu_2044	Anaerococcus
OTU_89	18812	100	0.999993	29	k_Bacteria; p_Actinobacteria; c_Actinobacteria (class); o_Actinomycetales; f_Propionibacteriaceae; g_Propionibacterium; s_Propionibacterium acnes; otu_720	Propionibacterium
OTU_90	103661	100	0.999993	29	k_Bacteria; p_Firmicutes; c_Clostridia; o_Clostridiales; f_Lachnospiraceae; g_Roseburia; s_Eubacterium rectale; otu_2131	Eubacterium rectale
OTU_91	111763	63.24	0.927641	29	k_Bacteria; p_Actinobacteria; c_Actinobacteria (class); o_Solirubrobacterales; f_Solirubrobacteraceae; Unclassified; otu_946	Gardnerella vaginalis
OTU_92	14257	65.22	0.962741	28	k_Bacteria; p_Firmicutes; c_Clostridia; o_Clostridiales; f_Eubacteriaceae; g_Acetobacterium; Unclassified; otu_2074	OTU_92
OTU_93	108085	57.35	0.887381	29	k_Bacteria; p_Actinobacteria; c_Actinobacteria (class); o_Actinomycetales; f_Micrococcaceae; g_Arthrobacter; Unclassified; otu_505	Actinobacteria
OTU_96	107102	100	0.999993	29	k_Bacteria; p_Proteobacteria; c_Betaproteobacteria; o_Burkholderiales; f_Burkholderiaceae; g_Ralstonia; Unclassified; otu_3005	Ralstonia
OTU_98	157133	71.64	0.999993	29	k_Bacteria; p_Actinobacteria; c_Actinobacteria (class); o_Actinomycetales; f_Intrasporangiaceae; g_Serinicoccus; otu_428	Actinomycetales
OTU_102	72264	100	0.999993	29	k_Bacteria; p_Actinobacteria; c_Actinobacteria (class); o_Actinomycetales; f_Corynebacteriaceae; g_Corynebacterium; Unclassified; otu_340	Corynebacterium
OTU_103	63560	100	0.999993	29	k_Bacteria; p_Firmicutes; c_Bacilli; o_Bacillales; f_Paenibacillaceae; g_Paenibacillus; Unclassified; otu_1688	Paenibacillus

sequence_id	greengenes prokMSA_id	greengenes Simrank id	greengenes DNAML id	greengenes DNAML columns	greengenes tax string	final name
OTU_106	298762	100	0.999993	29	k_Bacteria; p_Actinobacteria; c_Actinobacteria (class); o_Bifidobacteriales; f_Bifidobacteriaceae; g_Bifidobacterium; s_Bifidobacterium longum; otu_905	Bifidobacterium longum
OTU_107	68416	100	0.999993	29	k_Bacteria; p_Bacteroidetes; c_Bacteroidia; o_Bacteroidales; f_Prevotellaceae; g_Prevotella; Unclassified; otu_1034	Prevotella
OTU_110	14284	94.52	0.999993	29	k_Bacteria; p_Firmicutes; c_Clostridia; o_Clostridiales; f_Clostridiales Family XI. Incertae Sedis; g_Anaerococcus; Unclassified; otu_2044	Anaerococcus
OTU_111	57712	80.6	0.999993	29	k_Bacteria; p_Actinobacteria; c_Actinobacteria (class); o_Actinomycetales; f_Corynebacteriaceae; g_Corynebacterium; s_Corynebacterium aurimucosum; otu_344	Corynebacterium
OTU_116	151926	91.18	0.999993	29	k_Bacteria; p_Firmicutes; c_Clostridia; o_Clostridiales; f_Clostridiaceae; g_Clostridium; Unclassified; otu_2005	Clostridium
OTU_120	103407	68.12	0.965052	29	k_Bacteria; p_Firmicutes; c_Clostridia; o_Clostridiales; f_Lachnospiraceae; g_; Unclassified; otu_2088	OTU_120
OTU_123	368769	52.94	0.924153	28	k_Bacteria; p_Firmicutes; c_Clostridia; o_Clostridiales; f_Clostridiales Family XI. Incertae Sedis; g_Anaerococcus; s_Anaerococcus hydrogenalis; otu_2045	OTU_123
OTU_124	84459	70.31	0.926667	29	k_Bacteria; p_Actinobacteria; c_Actinobacteria (class); o_Actinomycetales; f_Corynebacteriaceae; g_Corynebacterium; s_Corynebacterium tuberculostearicum; otu_368	Actinobaculum
OTU_127	248907	92.65	0.964922	29	k_Bacteria; p_Actinobacteria; c_Actinobacteria (class); o_Actinomycetales; f_Corynebacteriaceae; g_Corynebacterium; Unclassified; otu_340	Corynebacterium
OTU_131	111763	58.82	0.927641	29	k_Bacteria; p_Actinobacteria; c_Actinobacteria (class); o_Solirubrobacterales; f_Solirubrobacteraceae; Unclassified; otu_946	Gardnerella
OTU_132	69910	60.29	0.928004	29	k_Bacteria; p_Bacteroidetes; c_Bacteroidia; o_Bacteroidales; f_Prevotellaceae; g_Prevotella; Unclassified; otu_1034	OTU_132
OTU_138	13945	43.28	0.451425	28	k_Bacteria; p_Firmicutes; c_Clostridia; o_Clostridiales; f_Lachnospiraceae; g_Butyrivibrio; Unclassified; otu_2097	OTU_138
OTU_140	90638	100	0.999993	29	k_Bacteria; p_Cyanobacteria; c_Chloroplast; o_Streptophyta; otu_1407	OTU_140
OTU_142	33338	52.24	0.999993	29	k_Bacteria; p_Bacteroidetes; c_Bacteroidia; o_Bacteroidales; f_Porphyromonadaceae; g_Porphyromonas; s_Porphyromonas gingivalis; otu_1029	Porphyromonas
OTU_145	101184	100	0.964951	29	k_Bacteria; p_Proteobacteria; c_Betaproteobacteria; o_Burkholderiales; Unclassified; otu_2913	Leptothrix
OTU_149	93286	85.92	0.926958	29	k_Bacteria; p_Proteobacteria; c_Betaproteobacteria; o_Gallionellales; f_Gallionellaceae; Unclassified; otu_3080	Betaproteobacteria
OTU_155	243807	86.96	0.964297	29	k_Bacteria; p_Firmicutes; c_Bacilli; o_Bacillales; f_Paenibacillaceae; g_Paenibacillus; Unclassified; otu_1688	Paenibacillus
OTU_156	50424	41.18	0.361167	29	k_Bacteria; p_Proteobacteria; c_Gammaproteobacteria; o_Alteromonadales; f_Alteromonadaceae; Unclassified; otu_3406	OTU_156
OTU_162	90545	65.15	0.964965	29	k_Bacteria; p_Actinobacteria; c_Actinobacteria (class); o_Actinomycetales; f_Streptomycetaceae; g_Streptomyces; Unclassified; otu_766	Actinobacteria
OTU_169	72536	72.31	0.999993	29	k_Bacteria; p_Actinobacteria; c_Actinobacteria (class); o_Actinomycetales; f_Actinomycetaceae; g_Actinomyces; Unclassified; otu_279	Actinomyces
OTU_175	14032	59.7	0.88507	29	k_Bacteria; p_Firmicutes; c_Clostridia; o_Clostridiales; f_Lachnospiraceae; g_Ruminococcus; s_Ruminococcus gnavus; otu_2134	Clostridiales
OTU_191	104204	61.19	0.843924	29	k_Bacteria; p_Firmicutes; c_Bacilli; o_Bacillales; f_Paenibacillaceae; g_Paenibacillus; s_Paenibacillus chitinolyticus; otu_1697	Peptostreptococcus
OTU_194	111763	57.35	0.841511	29	k_Bacteria; p_Actinobacteria; c_Actinobacteria (class); o_Solirubrobacterales; f_Solirubrobacteraceae; Unclassified; otu_946	Gardnerella
OTU_252	143401	37.14	0.66723	29	k_Bacteria; p_Proteobacteria; c_Gammaproteobacteria; o_Oceanospirillales; f_Alcanivoracaceae; g_Alcanivorax; s_Alcanivorax dieselolei; otu_3706	OTU_252
OTU_323	76393	58.82	0.928004	29	k_Bacteria; p_Bacteroidetes; c_Bacteroidia; o_Bacteroidales; f_Prevotellaceae; g_Prevotella; Unclassified; otu_1034	Prevotella
OTU_708	78555	64.71	0.886974	29	k_Bacteria; p_Actinobacteria; c_Actinobacteria (class); o_Bifidobacteriales; f_Bifidobacteriaceae; Unclassified; otu_894	Bifidobacteriaceae
