# Supplementary material for: Vaginal Microbiome and Epithelial Gene Array in Post-Menopausal Women with Moderate to Severe Dryness
Source: PLoS One. 2011 Nov 2;6(11):e26602. doi: 10.1371/journal.pone.0026602 (PMC3206802; doi:10.1371/journal.pone.0026602)
Supplement: Table S2 — Affymetrix read outs for 960 probe sets representing those differentially expressed by at least 2-fold. (PDF) [file pone.0026602.s004.pdf]

Column #	Transcript Cluster ID	gene_assignment	Gene Symbol	RefSeq	p-value(Group)	p-value(VVA vs. Control)	Ratio(VVA vs. Control)	Fold-Change(VVA vs. Control)	Fold-Change(VVA vs. Control) (Description)	F(Group)	SS(Group)	SS(Error)	F(Error)
25413	8109049	NM 032566 // SPINK7 // serine peptidase inhibitor, Kazal type 7 (putative) // 5q	SPINK7	NM 032566	0.000481089	0.000481089	0.0258712	-38.653	VVA down vs Control	31.9293	66.7185	16.7166	1
20498	8060432	NM 003245 // TGM3 // transglutaminase 3 (E polypeptide, protein-glutamine-gamma-	TGM3	NM 003245	4.24E-05	4.24E-05	0.0457455	-21.8601	VVA down vs Control	64.5243	47.5308	5.89308	1
18071	8036103	NM 198538 // SBSN // suprabasin // 19q13.13 // 374897 /// AY358701 // SBSN // su	SBSN	NM 198538	3.28E-05	3.28E-05	0.0477684	-20.9344	VVA down vs Control	69.2785	46.2067	5.33577	1
14926	8004221	NM 000697 // ALOX12 // arachidonate 12-lipoxygenase // 17p13.1 // 239 /// ENST00	ALOX12	NM 000697	0.000443231	0.000443231	0.050989	-19.6121	VVA down vs Control	32.7364	44.2455	10.8125	1
5092	7905515	NM 001025231 // KPRP // keratinocyte proline-rich protein // 1q21.3 // 448834 //	KPRP	NM 001025231	0.000230424	0.000230424	0.0573658	-17.432	VVA down vs Control	39.8095	40.8111	8.20128	1
10746	7961673	NM 021957 // GYS2 // glycogen synthase 2 (liver) // 12p12.2 // 2998 /// ENST0000	GYS2	NM 021957	0.00166379	0.00166379	0.0595864	-16.7823	VVA down vs Control	21.54	39.7337	14.7572	1
16529	8020724	NM 001942 // DSG1 // desmoglein 1 // 18q12.1 // 1828 /// ENST00000257192 // DSG1	DSG1	NM 001942	0.00501809	0.00501809	0.0602183	-16.6063	VVA down vs Control	14.6687	39.4371	21.5081	1
6527	7920182	NM 178435 // LCE3E // late cornified envelope 3E // 1q21.3 // 353145 /// ENST000	LCE3E	NM 178435	0.000677774	0.000677774	0.0651619	-15.3464	VVA down vs Control	28.7283	37.2534	10.374	1
6528	7920185	NM 032563 // LCE3D // late cornified envelope 3D // 1q21 // 84648 /// ENST000003	LCE3D	NM 032563	0.00327332	0.00327332	0.0684582	-14.6075	VVA down vs Control	17.1029	35.9192	16.8015	1
16618	8021594	NM 080474 // SERPINB12 // serpin peptidase inhibitor, clade B (ovalbumin), membe	SERPINB12	NM 080474	0.00141764	0.00141764	0.0708944	-14.1055	VVA down vs Control	22.7054	34.9885	12.3278	1
7565	7930760	NM 001011709 // PNLIPRP3 // pancreatic lipase-related protein 3 // 10q25.3 // 11	PNLIPRP3	NM 001011709	0.000306405	0.000306405	0.0730311	-13.6928	VVA down vs Control	36.585	34.2078	7.48017	1
18069	8036072	NM 207392 // KRTDAP // keratinocyte differentiation-associated protein // 19q13.	KRTDAP	NM 207392	0.00216392	0.00216392	0.0764524	-13.08	VVA down vs Control	19.726	33.0213	13.392	1
21449	8069790	NM 012131 // CLDN17 // claudin 17 // 21q22.11 // 26285 /// ENST00000286808 // CL	CLDN17	NM 012131	0.00198787	0.00198787	0.0776486	-12.8785	VVA down vs Control	20.2986	32.6237	12.8575	1
10903	7963491	NM 006121 // KRT1 // keratin 1 // 12q12-q13 // 3848 /// ENST00000252244 // KRT1	KRT1	NM 006121	0.00115579	0.00115579	0.0834338	-11.9855	VVA down vs Control	24.2618	30.8148	10.1608	1
27672	8130807	AL832737 // LOC441178 // hypothetical LOC441178 // 6q27 // 441178 /// AK056600 /	LOC441178	AL832737	0.00465675	0.00465675	0.0918464	-10.8877	VVA down vs Control	15.0756	28.4772	15.1116	1
27688	8130980	AL832737 // LOC441178 // hypothetical LOC441178 // 6q27 // 441178 /// AK056600 /	LOC441178	AL832737	0.00465675	0.00465675	0.0918464	-10.8877	VVA down vs Control	15.0756	28.4772	15.1116	1
27401	8127854	NM 002395 // ME1 // malic enzyme 1, NADP(+)-dependent, cytosolic // 6q12 // 4199	ME1	NM 002395	0.00132211	0.00132211	0.094627	-10.5678	VVA down vs Control	23.2281	27.7702	9.56433	1
16489	8020347	---		---	0.00170288	0.00170288	0.0974796	-10.2586	VVA down vs Control	21.3748	27.075	10.1334	1
5082	7905486	NM 019060 // CRCT1 // cysteine-rich C-terminal 1 // 1q21 // 54544 /// ENST000003	CRCT1	NM 019060	0.00385853	0.00385853	0.10203	-9.80103	VVA down vs Control	16.1344	26.0242	12.9037	1
27308	8126905	NM 006061 // CRISP3 // cysteine-rich secretory protein 3 // 6p12.3 // 10321 ///	CRISP3	NM 006061	0.0087558	0.0087558	0.102572	-9.74926	VVA down vs Control	11.8698	25.9036	17.4585	1
11010	7964687	NM 001099676 // C12orf56 // chromosome 12 open reading frame 56 // 12q14.2 // 11	C12orf56	NM 001099676	0.00115745	0.00115745	0.108742	-9.19604	VVA down vs Control	24.2506	24.5916	8.11249	1
33041	8179814	NM 025261 // LY6G6C // lymphocyte antigen 6 complex, locus G6C // 6p21.33 // 807	LY6G6C	NM 025261	0.00477655	0.00477655	0.110685	-9.03465	VVA down vs Control	14.9365	24.2006	12.9619	1
32916	8178586	NM 025261 // LY6G6C // lymphocyte antigen 6 complex, locus G6C // 6p21.33 // 807	LY6G6C	NM 025261	0.00469074	0.00469074	0.111355	-8.98032	VVA down vs Control	15.0357	24.0682	12.8059	1
27141	8125042	NM 025261 // LY6G6C // lymphocyte antigen 6 complex, locus G6C // 6p21.33 // 807	LY6G6C	NM 025261	0.00467873	0.00467873	0.117779	-8.49049	VVA down vs Control	15.0498	22.8539	12.1484	1
24016	8095723	NM 001013442 // EPGN // epithelial mitogen homolog (mouse) // 4q13.3 // 255324 /	EPGN	NM 001013442	0.00313442	0.00313442	0.117964	-8.47716	VVA down vs Control	17.365	22.8203	10.5133	1
7408	7928944	NM 004670 // PAPSS2 // 3'-phosphoadenosine 5'-phosphosulfate synthase 2 // 10q23	PAPSS2	NM 004670	0.00102947	0.00102947	0.118099	-8.46747	VVA down vs Control	25.1801	22.7959	7.24253	1
30696	8158771	NM 031426 // AIF1L // allograft inflammatory factor 1-like // 9q34.13-q34.3 // 8	AIF1L	NM 031426	0.000985012	0.000985012	0.122636	-8.1542	VVA down vs Control	25.5375	21.9985	6.89135	1
17794	8033151	NM 133492 // ACER1 // alkaline ceramidase 1 // 19p13.3 // 125981 /// ENST0000030	ACER1	NM 133492	0.000765824	0.000765824	0.123971	-8.0664	VVA down vs Control	27.6526	21.7721	6.29873	1
18543	8040340	NM 145693 // LPIN1 // lipin 1 // 2p25.1 // 23175 /// ENST00000256720 // LPIN1 //	LPIN1	NM 145693	0.000824914	0.000824914	0.125617	-7.96072	VVA down vs Control	27.014	21.4979	6.36643	1
7246	7927363	NM 001137556 // FAM25B // family with sequence similarity 25, member B // 10q11.	FAM25B	NM 001137556	0.000880751	0.000880751	0.126216	-7.92295	VVA down vs Control	26.4611	21.3994	6.4697	1
7823	7933327	NM 001137556 // FAM25B // family with sequence similarity 25, member B // 10q11.	FAM25B	NM 001137556	0.000880751	0.000880751	0.126216	-7.92295	VVA down vs Control	26.4611	21.3994	6.4697	1
7834	7933423	NM 001137556 // FAM25B // family with sequence similarity 25, member B // 10q11.	FAM25B	NM 001137556	0.000880751	0.000880751	0.126216	-7.92295	VVA down vs Control	26.4611	21.3994	6.4697	1
18978	8044548	NM 014440 // IL1F6 // interleukin 1 family, member 6 (epsilon) // 2q12-q14.1 //	IL1F6	NM 014440	0.00518785	0.00518785	0.131057	-7.63029	VVA down vs Control	14.49	20.6282	11.3889	1
6526	7920178	NM 016190 // CRNN // cornulin // 1q21 // 49860 /// ENST00000271835 // CRNN // co	CRNN	NM 016190	0.000973862	0.000973862	0.133206	-7.50716	VVA down vs Control	25.6303	20.2992	6.33602	1
7995	7934997	NM 005398 // PPP1R3C // protein phosphatase 1, regulatory (inhibitor) subunit 3C	PPP1R3C	NM 005398	0.000998305	0.000998305	0.138026	-7.24503	VVA down vs Control	25.4285	19.5898	6.16308	1
6892	7923967	NM 018566 // YOD1 // YOD1 OTU deubiquinating enzyme 1 homolog (S. cerevisiae) //	YOD1	NM 018566	0.000796014	0.000796014	0.148673	-6.72616	VVA down vs Control	27.3189	18.1471	5.31415	1
24219	8097717	NM 024605 // ARHGAP10 // Rho GTPase activating protein 10 // 4q31.23 // 79658 //	ARHGAP10	NM 024605	0.00178299	0.00178299	0.154657	-6.46592	VVA down vs Control	21.0507	17.4035	6.61396	1
10825	7962608	NM 006025 // P11 // 26 serine protease // 12q13.1 // 8909 /// ENST00000229003 //	P11	NM 006025	0.00492772	0.00492772	0.156771	-6.37873	VVA down vs Control	14.767	17.1513	9.29171	1
18532	8040249	NM 005742 // PDIA6 // protein disulfide isomerase family A, member 6 // 2p25.1 /	PDIA6	NM 005742	0.00287828	0.00287828	0.157366	-6.35463	VVA down vs Control	17.8888	17.0813	7.63889	1
16491	8020352	---		---	0.00378159	0.00378159	0.160861	-6.21656	VVA down vs Control	16.2508	16.6779	8.21023	1
23925	8094988	NM 025087 // CWH43 // cell wall biogenesis 43 C-terminal homolog (S. cerevisiae)	CWH43	NM 025087	0.00710271	0.00710271	0.161875	-6.17761	VVA down vs Control	12.8754	16.5633	10.2914	1
28236	8136200	NM 016352 // CPA4 // carboxypeptidase A4 // 7q32 // 51200 /// ENST00000222482 //	CPA4	NM 016352	0.00127454	0.00127454	0.162106	-6.1688	VVA down vs Control	23.5064	16.5374	5.62821	1
11554	7970448	NM 001110219 // GJB6 // gap junction protein, beta 6, 30kDa // 13q11-q12.1|13q12	GJB6	NM 001110219	0.0153612	0.0153612	0.163651	-6.11057	VVA down vs Control	9.42139	16.3654	13.8964	1
16005	8015376	NM 005557 // KRT16 // keratin 16 // 17q12-q21 // 3868 /// ENST00000301653 // KRT	KRT16	NM 005557	0.000970611	0.000970611	0.167524	-5.96931	VVA down vs Control	25.6576	15.9452	4.9717	1
28294	8136807				0.00114842	0.00114842	0.170199	-5.87547	VVA down vs Control	24.3118	15.6636	5.15424	1
6535	7920214	NM 001024209 // SPRR2E // small proline-rich protein 2E // 1q21-q22 // 6704 ///	SPRR2E	NM 001024209	0.0419508	0.0419508	0.170648	-5.86001	VVA down vs Control	5.84894	15.6171	21.3605	1
10909	7963555	NM 173352 // KRT78 // keratin 78 // 12q13.13 // 196374 /// ENST00000304620 // KR	KRT78	NM 173352	0.00260231	0.00260231	0.172124	-5.80976	VVA down vs Control	18.5228	15.4653	6.67945	1
24551	8100701	NM 182502 // TMPRSS11B // transmembrane protease, serine 11B // 4q13.2 // 132724	TMPRSS11B	NM 182502	0.00508889	0.00508889	0.177679	-5.62813	VVA down vs Control	14.5933	14.912	8.17472	1
11544	7970388	---		---	0.00164476	0.00164476	0.179263	-5.5784	VVA down vs Control	21.6222	14.7592	5.46077	1
19897	8053751	---		---	0.00380907	0.00380907	0.180976	-5.5256	VVA down vs Control	16.2089	14.5963	7.20412	1
30959	8161405	---		---	0.00380907	0.00380907	0.180976	-5.5256	VVA down vs Control	16.2089	14.5963	7.20412	1
21419	8069517	---		---	0.00146161	0.00146161	0.182667	-5.47445	VVA down vs Control	22.4794	14.4379	5.13818	1
27307	8126891	NM 003296 // CRISP2 // cysteine-rich secretory protein 2 // 6p21-qter // 7180 //	CRISP2	NM 003296	0.00547465	0.00547465	0.1843	-5.42593	VVA down vs Control	14.2042	14.2871	8.0467	1
13393	7987792	NM 178034 // PLA2G4D // phospholipase A2, group IVD (cytosolic) // 15q15.1 // 28	PLA2G4D	NM 178034	0.00396082	0.00396082	0.188202	-5.31345	VVA down vs Control	15.984	13.9354	6.97467	1
21247	8067864	NR 003366 // ANKRD20B // ankyrin repeat domain 20B // 2q11.1 // 729171 /// NM 03	ANKRD20B	NR 003366	0.00281465	0.00281465	0.188883	-5.29429	VVA down vs Control	18.028	13.8752	6.15716	1
6891	7923965	---		---	0.00406995	0.00406995	0.191259	-5.22852	VVA down vs Control	15.8288	13.6678	6.90779	1
23263	8088436	BC050317 // C3orf67 // chromosome 3 open reading frame 67 // 3p14.2 // 200844 //	C3orf67	BC050317	0.00575325	0.00575325	0.198263	-5.04381	VVA down vs Control	13.944	13.0799	7.50424	1
30143	8153336	NM 020427 // SLURP1 // secreted LY6/PLAUR domain containing 1 // 8q24.3 // 57152	SLURP1	NM 020427	0.00141121	0.00141121	0.199453	-5.01372	VVA down vs Control	22.7392	12.9833	4.56774	1
21416	8069505	BX648100 // C21orf15 // chromosome 21 open reading frame 15 // 21q11 // 54094	C21orf15	BX648100	0.00415016	0.00415016	0.20114	-4.97167	VVA down vs Control	15.7181	12.848	6.53924	1
24055	8096116	NM 032717 // AGPAT9 // 1-acylglycerol-3-phosphate O-acyltransferase 9 // 4q21.23	AGPAT9	NM 032717	0.0186754	0.0186754	0.204065	-4.90041	VVA down vs Control	8.65065	12.6178	11.6687	1
14899	8003889	---		---	0.0101151	0.0101151	0.205984	-4.85474	VVA down vs Control	11.207	12.4695	8.90127	1
18351	8038747	NM 145894 // KLK12 // kallikrein-related peptidase 12 // 19q13.3-q13.4 // 43849	KLK12	NM 145894	0.0494377	0.0494377	0.207233	-4.82549	VVA down vs Control	5.35112	12.3743	18.4998	1
15950	8014956	NM 021724 // NR1D1 // nuclear receptor subfamily 1, group D, member 1 // 17q11.2	NR1D1	NM 021724	0.0122801	0.0122801	0.210008	-4.76173	VVA down vs Control	10.3541	12.166	9.4	1
25410	8109001	NM 001127698 // SPINK5 // serine peptidase inhibitor, Kazal type 5 // 5q32 // 11	SPINK5	NM 001127698	0.0020074	0.0020074	0.210763	-4.74467	VVA down vs Control	20.232	12.1102	4.78852	1
16844	8023688	NM 002974 // SERPINB4 // serpin peptidase inhibitor, clade B (ovalbumin), member	SERPINB4	NM 002974	0.0352825	0.0352825	0.210992	-4.73951	VVA down vs Control	6.3984	12.0932	15.1203	1
25437	8109333	NM 002084 // GPX3 // glutathione peroxidase 3 (plasma) // 5q23 // 2878 /// ENST0	GPX3	NM 002084	0.000903262	0.000903262	0.211018	-4.73893	VVA down vs Control	26.2505	12.0913	3.68491	1
4394	7898655	NM 001785 // CDA // cytidine deaminase // 1p36.2-p35 // 978 /// ENST00000375071	CDA	NM 001785	0.00548895	0.00548895	0.213789	-4.67752	VVA down vs Control	14.1905	11.8894	6.70278	1
15691	8012309	NM 001139 // ALOX12B // arachidonate 12-lipoxygenase, 12R type // 17p13.1 // 242	ALOX12B	NM 001139	0.0062152	0.0062152	0.220394	-4.53732	VVA down vs Control	13.5456	11.425	6.74758	1
25783	8112672	AK097510 // ANKRD31 // ankyrin repeat domain 31 // 5q13.3 // 256006 /// ENST0000	ANKRD31	AK097510	0.0197081	0.0197081	0.220622	-4.53264	VVA down vs Control	8.44511	11.4094	10.8081	1
12384	7978222	NM 000359 // TGM1 // transglutaminase 1 (K polypeptide epidermal type I, protein	TGM1	NM 000359	0.0172239	0.0172239	0.22387	-4.46688	VVA down vs Control	8.96509	11.1899	9.98527	1
10970	7964285	NM 148897 // SDR9C7 // short chain dehydrogenase/reductase family 9C, member 7 /	SDR9C7	NM 148897	0.0114745	0.0114745	0.224133	-4.46164	VVA down vs Control	10.6476	11.1723	8.39422	1
30422	8155898	NM 006200 // PCSK5 // proprotein convertase subtilisin/kexin type 5 // 9q21.3 //	PCSK5	NM 006200	0.00143861	0.00143861	0.232903	-4.29364	VVA down vs Control	22.5966	10.6062	3.75497	1
28293	8136801	NR 001296 // TRY6 // trypsinogen C // 7q34 // 154754 /// NM 002770 // PRSS2 // p	TRY6	NR 001296	0.00125078	0.00125078	0.233721	-4.27861	VVA down vs Control	23.6503	10.5552	3.57042	1
9855	7952436	NM 138961 // ESAM // endothelial cell adhesion molecule // 11q24.2 // 90952 ///	ESAM	NM 138961	0.00729799	0.00729799	0.234489	-4.26459	VVA down vs Control	12.742	10.5076	6.59715	1
10177	7955736	NM 012291 // ESPL1 // extra spindle pole bodies homolog 1 (S. cerevisiae) // 12q	ESPL1	NM 012291	0.00180074	0.00180074	0.236093	-4.23563	VVA down vs Control	20.9813	10.4091	3.9689	1
6534	7920210	NM 001017418 // SPRR2B // small proline-rich protein 2B // 1q21-q22 // 6701 ///	SPRR2B	NM 001017418	0.0492788	0.0492788	0.237517	-4.21023	VVA down vs Control	5.36066	10.3225	15.4048	1
26509	8119161	NM 002648 // PIM1 // pim-1 oncogene // 6p21.2 // 5292 /// ENST00000373509 // PIM	PIM1	NM 002648	0.00213304	0.00213304	0.237734	-4.20638	VVA down vs Control	19.8221	10.3094	4.16077	1
11467	7969531	---		---	0.000832439	0.000832439	0.23862	-4.19076	VVA down vs Control	26.9368	10.2561	3.04597	1
12077	7975284	NM 152443 // RDH12 // retinol dehydrogenase 12 (all-trans/9-cis/11-cis) // 14q24	RDH12	NM 152443	0.00410204	0.00410204	0.239183	-4.1809	VVA down vs Control	15.7842	10.2224	5.18107	1
21857	8073680	---		---	0.00883239	0.00883239	0.241163	-4.14658	VVA down vs Control	11.8291	10.1049	6.83397	1
17113	8026424	NM 173483 // CYP4F22 // cytochrome P450, family 4, subfamily F, polypeptide 22 /	CYP4F22	NM 173483	0.0237175	0.0237175	0.242071	-4.13102	VVA down vs Control	7.75976	10.0516	10.3628	1
12715	7981514	NM 138420 // AHNAK2 // AHNAK nucleoprotein 2 // 14q32.33 // 113146 /// ENST00000	AHNAK2	NM 138420	0.000367291	0.000367291	0.244139	-4.09602	VVA down vs Control	34.651	9.93135	2.29288	1
24639	8101609	NM 197965 // SLC10A6 // solute carrier family 10 (sodium/bile acid cotransporter	SLC10A6	NM 197965	0.00127275	0.00127275	0.244243	-4.09429	VVA down vs Control	23.5171	9.92539	3.3764	1
16563	8021113	NM 145055 // C18orf25 // chromosome 18 open reading frame 25 // 18q21.1 // 14733	C18orf25	NM 145055	0.00159586	0.00159586	0.244772	-4.08544	VVA down vs Control	21.8389	9.89494	3.6247	1
14438	7999253	NM 002705 // PPL // periplakin // 16p13.3 // 5493 /// ENST00000345988 // PPL //	PPL	NM 002705	0.000686626	0.000686626	0.245652	-4.07079	VVA down vs Control	28.6124	9.84451	2.75251	1
23460	8090433	NM 007283 // MGLL // monoglyceride lipase // 3q21.3 // 11343 /// NM 001003794 //	MGLL	NM 007283	0.000339038	0.000339038	0.247299	-4.04369	VVA down vs Control	35.4943	9.75104	2.19777	1
33028	8179709	NM 205854 // SFTA2 // surfactant associated 2 // 6p21.33 // 389376 /// ENST00000	SFTA2	NM 205854	0.00232384	0.00232384	0.247361	-4.04268	VVA down vs Control	19.2543	9.74756	4.05003	1
19980	8054712	NM 000575 // IL1A // interleukin 1, alpha // 2q14 // 3552 /// ENST00000263339 //	IL1A	NM 000575	0.0274364	0.0274364	0.250272	-3.99566	VVA down vs Control	7.24372	9.58495	10.5857	1
21418	8069511	NR 027270 // C21orf81 // ankyrin repeat domain 20 family, member A3 pseudogene /	C21orf81	NR 027270	0.00426716	0.00426716	0.251986	-3.96847	VVA down vs Control	15.5613	9.49072	4.87914	1
29069	8143803	NM 173681 // ATG9B // ATG9 autophagy related 9 homolog B (S. cerevisiae) // 7q36	ATG9B	NM 173681	0.00287346	0.00287346	0.252038	-3.96766	VVA down vs Control	17.8992	9.4879	4.2406	1
27867	8132725	NM 003364 // UPP1 // uridine phosphorylase 1 // 7p12.3 // 7378 /// NM 181597 //	UPP1	NM 003364	0.0089555	0.0089555	0.254376	-3.93119	VVA down vs Control	11.7646	9.36118	6.36568	1
24998	8104723	---		---	0.00698097	0.00698097	0.255492	-3.91401	VVA down vs Control	12.9609	9.30139	5.74118	1
24017	8095728	NM 001432 // EREG // epiregulin // 4q13.3 // 2069 /// ENST00000244869 // EREG //	EREG	NM 001432	0.0148424	0.0148424	0.256943	-3.89191	VVA down vs Control	9.56103	9.22434	7.71828	1
26855	8122637	NM 015278 // SASH1 // SAM and SH3 domain containing 1 // 6q24.3 // 23328 /// ENS	SASH1	NM 015278	0.000566726	0.000566726	0.258371	-3.8704	VVA down vs Control	30.365	9.14926	2.41048	1
26713	8121277	NM 001624 // AIM1 // absent in melanoma 1 // 6q21 // 202 /// ENST00000369066 //	AIM1	NM 001624	0.000903742	0.000903742	0.259495	-3.85363	VVA down vs Control	26.2461	9.09064	2.7709	1
7578	7930882	NM 207009 // FAM45A // family with sequence similarity 45, member A // 10q25 //	FAM45A	NM 207009	0.000652343	0.000652343	0.263711	-3.79203	VVA down vs Control	29.0719	8.87477	2.44216	1
2350	7894865	---		---	0.019028	0.019028	0.263918	-3.78905	VVA down vs Control	8.5789	8.86431	8.26615	1
17845	8033780	NM 024106 // ZNF426 // zinc finger protein 426 // 19p13.2 // 79088 /// ENST00000	ZNF426	NM 024106	0.00274825	0.00274825	0.264222	-3.7847	VVA down vs Control	18.1776	8.849	3.89447	1
28248	8136336	NM 020299 // AKR1B10 // aldo-keto reductase family 1, member B10 (aldose reducta	AKR1B10	NM 020299	0.0239614	0.0239614	0.264274	-3.78395	VVA down vs Control	7.72286	8.84637	9.16383	1
10421	7958262	NM 152772 // TCP11L2 // t-complex 11 (mouse)-like 2 // 12q23.3 // 255394 /// ENS	TCP11L2	NM 152772	0.00259086	0.00259086	0.264752	-3.77712	VVA down vs Control	18.5509	8.82236	3.80461	1
16488	8020343	BC127726 // ANKRD20A5 // ankyrin repeat domain 20 family, member A5 // 18p11.21	ANKRD20A5	BC127726	0.00540645	0.00540645	0.266228	-3.75618	VVA down vs Control	14.2705	8.74873	4.90453	1
27118	8124855	NM 205854 // SFTA2 // surfactant associated 2 // 6p21.33 // 389376 /// ENST00000	SFTA2	NM 205854	0.00248334	0.00248334	0.26629	-3.7553	VVA down vs Control	18.8227	8.74561	3.71704	1
21111	8066489	NM 080869 // WFDC12 // WAP four-disulfide core domain 12 // 20q12-q13.1 // 12848	WFDC12	NM 080869	0.00334364	0.00334364	0.266529	-3.75194	VVA down vs Control	16.9754	8.73378	4.11597	1
18352	8038760	NM 015596 // KLK13 // kallikrein-related peptidase 13 // 19q13.3-q13.4 // 26085	KLK13	NM 015596	0.0182384	0.0182384	0.267275	-3.74146	VVA down vs Control	8.74199	8.69688	7.95872	1
27608	8130173	NM 130900 // RAET1L // retinoic acid early transcript 1L // 6q25.1 // 154064 ///	RAET1L	NM 130900	0.00141732	0.00141732	0.267707	-3.73543	VVA down vs Control	22.7071	8.67561	3.05653	1
10422	7958273	---		---	0.00536975	0.00536975	0.270208	-3.70086	VVA down vs Control	14.3065	8.55364	4.78307	1
21005	8065485	NM 025176 // NINL // ninein-like // 20p11.22-p11.1 // 22981 /// ENST00000278886	NINL	NM 025176	0.00318842	0.00318842	0.272269	-3.67284	VVA down vs Control	17.2613	8.45457	3.91838	1
4629	7901054	NM 004073 // PLK3 // polo-like kinase 3 (Drosophila) // 1p34.1 // 1263 /// ENST0	PLK3	NM 004073	0.00773329	0.00773329	0.27358	-3.65524	VVA down vs Control	12.4601	8.39225	5.38824	1
5445	7909283	---		---	0.00815004	0.00815004	0.274194	-3.64705	VVA down vs Control	12.2083	8.36322	5.48036	1
24997	8104693	NM 178140 // PDZD2 // PDZ domain containing 2 // 5p13.3 // 23037 /// ENST0000043	PDZD2	NM 178140	0.00132079	0.00132079	0.274544	-3.6424	VVA down vs Control	23.2357	8.34674	2.87376	1
7486	7929816	NM 005063 // SCD // stearoyl-CoA desaturase (delta-9-desaturase) // 10q24.31 //	SCD	NM 005063	0.0141085	0.0141085	0.274571	-3.64205	VVA down vs Control	9.76944	8.3455	6.83396	1
7412	7928994	NM 001080518 // LIPK // lipase, family member K // 10q23.31 // 643414 /// ENST00	LIPK	NM 001080518	0.0226113	0.0226113	0.274882	-3.63793	VVA down vs Control	7.93334	8.33089	8.40089	1
4368	7898413	NM 013358 // PADI1 // peptidyl arginine deiminase, type I // 1p36.13 // 29943 //	PADI1	NM 013358	0.0028084	0.0028084	0.275619	-3.6282	VVA down vs Control	18.0419	8.29638	3.67872	1
6531	7920196	NM 006945 // SPRR2D // small proline-rich protein 2D // 1q21-q22 // 6703 /// NM	SPRR2D	NM 006945	0.0417321	0.0417321	0.276904	-3.61136	VVA down vs Control	5.86516	8.2366	11.2346	1
18839	8043310	NM 022780 // RMND5A // required for meiotic nuclear division 5 homolog A (S. cer	RMND5A	NM 022780	0.000252838	0.000252838	0.278266	-3.59368	VVA down vs Control	38.7338	8.17376	1.68819	1
22832	8083707	ENST00000326474 // LOC401097 // Similar to LOC166075 // 3q25.33 // 401097 /// BC	LOC401097	ENST00000326474	0.00752513	0.00752513	0.279174	-3.582	VVA down vs Control	12.5923	8.1322	5.16644	1
20704	8062571	NM 030919 // FAM83D // family with sequence similarity 83, member D // 20q11.22-	FAM83D	NM 030919	0.00389168	0.00389168	0.27932	-3.58013	VVA down vs Control	16.0851	8.12554	4.04128	1
8757	7942551	---		---	0.00386388	0.00386388	0.282542	-3.5393	VVA down vs Control	16.1264	7.98004	3.95875	1
14898	8003875	NM 001124758 // SPNS2 // spinster homolog 2 (Drosophila) // 17p13.2 // 124976 //	SPNS2	NM 001124758	0.00787319	0.00787319	0.284258	-3.51794	VVA down vs Control	12.3737	7.90378	5.11005	1
18070	8036079	NM 033317 // DMKN // dermokine // 19q13.12 // 93099 /// NM 001126061 // DMKN //	DMKN	NM 033317	0.000267561	0.000267561	0.288794	-3.46267	VVA down vs Control	38.0901	7.70605	1.61849	1
16696	8022356	NM 001128626 // SPIRE1 // spire homolog 1 (Drosophila) // 18p11.21 // 56907 ///	SPIRE1	NM 001128626	0.00280863	0.00280863	0.289644	-3.45251	VVA down vs Control	18.0414	7.66964	3.40091	1
7448	7929466	NM 000772 // CYP2C18 // cytochrome P450, family 2, subfamily C, polypeptide 18 /	CYP2C18	NM 000772	0.0250107	0.0250107	0.291685	-3.42835	VVA down vs Control	7.56936	7.58295	8.01437	1
29572	8148553	NM 017527 // LY6K // lymphocyte antigen 6 complex, locus K // 8q24.3 // 54742 //	LY6K	NM 017527	0.00603351	0.00603351	0.292833	-3.41492	VVA down vs Control	13.6978	7.5347	4.40055	1
18355	8038785	BC032383 // LOC147645 // hypothetical protein LOC147645 // 19q13.41 // 147645 //	LOC147645	BC032383	0.00724785	0.00724785	0.292917	-3.41393	VVA down vs Control	12.7758	7.53115	4.71587	1
20525	8060745	NM 175839 // SMOX // spermine oxidase // 20p13 // 54498 /// NM 175842 // SMOX //	SMOX	NM 175839	0.00810923	0.00810923	0.294001	-3.40135	VVA down vs Control	12.2322	7.48593	4.89588	1
7287	7927745	---		---	0.0205775	0.0205775	0.295722	-3.38155	VVA down vs Control	8.28236	7.4147	7.16191	1
23379	8089467	NM 024508 // ZBED2 // zinc finger, BED-type containing 2 // 3q13.2 // 79413 ///	ZBED2	NM 024508	0.00989964	0.00989964	0.296503	-3.37264	VVA down vs Control	11.3043	7.38262	5.22465	1
7404	7928907	---		---	0.00211596	0.00211596	0.296968	-3.36737	VVA down vs Control	19.876	7.36364	2.96382	1
13564	7990054	NM 018003 // UACA // uveal autoantigen with coiled-coil domains and ankyrin repe	UACA	NM 018003	0.00340488	0.00340488	0.297978	-3.35595	VVA down vs Control	16.8671	7.32247	3.47301	1
28291	8136790	---		---	0.004601	0.004601	0.298222	-3.35321	VVA down vs Control	15.1419	7.3126	3.86349	1
8904	7943867	NM 031938 // BCO2 // beta-carotene oxygenase 2 // 11q22.3-q23.1 // 83875 /// NM	BCO2	NM 031938	0.00236406	0.00236406	0.298266	-3.35271	VVA down vs Control	19.1421	7.31082	3.05539	1
31379	8165639	BC052297 // C9orf169 // chromosome 9 open reading frame 169 // 9q34.3 // 375791	C9orf169	BC052297	0.0035996	0.0035996	0.299068	-3.34372	VVA down vs Control	16.5384	7.2784	3.52072	1
24220	8097743	---		---	0.00973989	0.00973989	0.299446	-3.3395	VVA down vs Control	11.3782	7.26318	5.10674	1
30154	8153424	NM 001100878 // C8orf73 // chromosome 8 open reading frame 73 // 8q24.3 // 64247	C8orf73	NM 001100878	0.00942619	0.00942619	0.299616	-3.3376	VVA down vs Control	11.5279	7.25632	5.03568	1
24322	8098604	NM 181726 // ANKRD37 // ankyrin repeat domain 37 // 4q35.1 // 353322 /// NM 0183	ANKRD37	NM 181726	0.0149097	0.0149097	0.303408	-3.29589	VVA down vs Control	9.54259	7.10569	5.95703	1
8195	7937438	NM 174940 // TMEM80 // transmembrane protein 80 // 11p15.5 // 283232 /// NM 0010	TMEM80	NM 174940	0.00139952	0.00139952	0.303599	-3.29382	VVA down vs Control	22.8011	7.09821	2.49048	1
16327	8018966	NM 003255 // TIMP2 // TIMP metallopeptidase inhibitor 2 // 17q25 // 7077 /// ENS	TIMP2	NM 003255	0.0079921	0.0079921	0.30419	-3.28742	VVA down vs Control	12.3018	7.07506	4.601	1
22674	8082058	NM 005213 // CSTA // cystatin A (stefin A) // 3q21 // 1475 /// ENST00000264474 /	CSTA	NM 005213	0.0137532	0.0137532	0.304835	-3.28046	VVA down vs Control	9.87531	7.04987	5.71111	1
22937	8084757	NM 198485 // TPRG1 // tumor protein p63 regulated 1 // 3q28 // 285386 /// ENST00	TPRG1	NM 198485	0.0475026	0.0475026	0.304851	-3.28029	VVA down vs Control	5.47011	7.04927	10.3095	1
8345	7938625	---		---	0.0237314	0.0237314	0.305146	-3.27712	VVA down vs Control	7.75764	7.0378	7.25767	1
16845	8023696	NM 006919 // SERPINB3 // serpin peptidase inhibitor, clade B (ovalbumin), member	SERPINB3	NM 006919	0.0161652	0.0161652	0.305188	-3.27667	VVA down vs Control	9.21633	7.03617	6.10756	1
11047	7965036	---		---	0.0121338	0.0121338	0.307153	-3.25571	VVA down vs Control	10.4055	6.96026	5.35119	1
19896	8053744	NR 003366 // ANKRD20B // ankyrin repeat domain 20B // 2q11.1 // 729171 /// NM 00	ANKRD20B	NR 003366	0.00661193	0.00661193	0.307292	-3.25424	VVA down vs Control	13.2321	6.95494	4.20489	1
21417	8069508	ENST00000333394 // CCDC29 // coiled-coil domain containing 29 // 9q21.11 // 7287	CCDC29	ENST00000333394	0.0311743	0.0311743	0.307413	-3.25295	VVA down vs Control	6.80735	6.95027	8.16795	1
20825	8063729	NM 207032 // EDN3 // endothelin 3 // 20q13.2-q13.3 // 1908 /// NM 207034 // EDN3	EDN3	NM 207032	0.0268777	0.0268777	0.307848	-3.24835	VVA down vs Control	7.31541	6.93362	7.58248	1
5621	7911138	NM 152609 // C1orf71 // chromosome 1 open reading frame 71 // 1q44 // 163882 ///	C1orf71	NM 152609	0.00714239	0.00714239	0.30794	-3.24738	VVA down vs Control	12.8479	6.9301	4.31515	1
30357	8155397	NM 001012419 // ANKRD20A3 // ankyrin repeat domain 20 family, member A3 // 9p12	ANKRD20A3	NM 001012419	0.00898665	0.00898665	0.308781	-3.23854	VVA down vs Control	11.7484	6.89806	4.69719	1
30958	8161388	NM 001012419 // ANKRD20A3 // ankyrin repeat domain 20 family, member A3 // 9p12	ANKRD20A3	NM 001012419	0.00898665	0.00898665	0.308781	-3.23854	VVA down vs Control	11.7484	6.89806	4.69719	1
14719	8002082	NM 030819 // GFOD2 // glucose-fructose oxidoreductase domain containing 2 // 16q	GFOD2	NM 030819	0.00454348	0.00454348	0.30902	-3.23603	VVA down vs Control	15.2114	6.88897	3.62305	1
30392	8155574	NM 032250 // ANKRD20A1 // ankyrin repeat domain 20 family, member A1 // 9q13 //	ANKRD20A1	NM 032250	0.0084509	0.0084509	0.309586	-3.23011	VVA down vs Control	12.0363	6.86751	4.56452	1
17610	8031414	NM 133180 // EPS8L1 // EPS8-like 1 // 19q13.42 // 54869 /// NM 017729 // EPS8L1	EPS8L1	NM 133180	0.0120411	0.0120411	0.310806	-3.21744	VVA down vs Control	10.4386	6.82152	5.22794	1
5105	7905563	NM 000427 // LOR // loricrin // 1q21 // 4014 /// ENST00000368742 // LOR // loric	LOR	NM 000427	0.0176754	0.0176754	0.31124	-3.21296	VVA down vs Control	8.8638	6.80525	6.14206	1
16490	8020349	NR 003366 // ANKRD20B // ankyrin repeat domain 20B // 2q11.1 // 729171 /// ENST0	ANKRD20B	NR 003366	0.0292379	0.0292379	0.311303	-3.21231	VVA down vs Control	7.02459	6.8029	7.74754	1

Column #	Transcript Cluster ID	gene_assignment	Gene Symbol	RefSeq	p-value(Group)	p-value(VVA vs. Control)	Ratio(VVA vs. Control)	Fold-Change(VVA vs. Control)	Fold-Change(VVA vs. Control) (Description)	F(Group)	SS(Group)	SS(Error)	F(Error)
19067	8045229	NM_032995 // ARHGEF4 // Rho guanine nucleotide exchange factor (GEF) 4 // 2q22 /	ARHGEF4	NM_032995	0.00341914	0.00341914	0.312207	-3.203	VVA down vs Control	16.8423	6.76912	3.2153	1
8692	7941761	NM_014578 // RHOD // ras homolog gene family, member D // 11q14.3 // 29984 /// E	RHOD	NM_014578	0.00133124	0.00133124	0.313129	-3.19357	VVA down vs Control	23.1762	6.73486	2.32476	1
26843	8122426	NM_014721 // PHACTR2 // phosphatase and actin regulator 2 // 6q24.2 // 9749 ///	PHACTR2	NM_014721	0.00105532	0.00105532	0.313395	-3.19086	VVA down vs Control	24.981	6.72052	2.15364	1
27625	8130422	NM_173515 // CNKSR3 // CNKSR family member 3 // 6q25.2 // 154043 /// ENST0000036	CNKSR3	NM_173515	0.00270652	0.00270652	0.316183	-3.16272	VVA down vs Control	18.2739	6.62274	2.89932	1
13010	7983867	NM_032866 // CGNL1 // cingulin-like 1 // 15q21.3 // 84952 /// ENST00000281282 //	CGNL1	NM_032866	0.00919613	0.00919613	0.31957	-3.12921	VVA down vs Control	11.6416	6.50076	4.46724	1
18189	8037179	NM_032488 // CNFN // cornifelin // 19q13.2 // 84518 /// ENST00000222032 // CNFN	CNFN	NM_032488	0.0186805	0.0186805	0.319661	-3.12831	VVA down vs Control	8.6496	6.49749	6.00951	1
11874	7973149	NM_032572 // RNASE7 // ribonuclease, RNase A family, 7 // 14q11.2 // 84659 /// E	RNASE7	NM_032572	0.0491019	0.0491019	0.322033	-3.10527	VVA down vs Control	5.37134	6.41354	9.55224	1
26645	8120756	---		---	0.00111517	0.00111517	0.322644	-3.09939	VVA down vs Control	24.5428	6.39211	2.08358	1
19762	8052654	NM_020651 // PELI1 // pellino homolog 1 (Drosophila) // 2p13.3 // 57162 /// ENST	PELI1	NM_020651	0.00393957	0.00393957	0.32286	-3.09731	VVA down vs Control	16.0148	6.38453	3.18931	1
5325	7907847	---		---	0.0257496	0.0257496	0.323405	-3.0921	VVA down vs Control	7.46607	6.36551	6.82074	1
26901	8122986	NM_016224 // SNX9 // sorting nexin 9 // 6q25.1-q26 // 51429 /// ENST00000392185	SNX9	NM_016224	0.00726627	0.00726627	0.324365	-3.08295	VVA down vs Control	12.7634	6.33212	3.96893	1
25276	8107632	NM_014035 // SNX24 // sorting nexin 24 // 5q23.2 // 28966 /// ENST00000261369 //	SNX24	NM_014035	0.0118787	0.0118787	0.324864	-3.07821	VVA down vs Control	10.4972	6.31483	4.81257	1
30397	8155602	NM_001012421 // ANKRD20A2 // ankyrin repeat domain 20 family, member A2 // 9p12	ANKRD20A2	NM_001012421	0.0116225	0.0116225	0.326408	-3.06365	VVA down vs Control	10.5918	6.2617	4.72949	1
21209	8067543	ENST00000370520 // RP11-93B14.6 // hypothetical protein FLJ32154 // 20q13.33 //	RP11-93B14.6	ENST00000370520	0.00688477	0.00688477	0.326525	-3.06256	VVA down vs Control	13.0299	6.2577	3.84206	1
6550	7920297	NM_020672 // S100A14 // S100 calcium binding protein A14 // 1q21.3 // 57402 ///	S100A14	NM_020672	0.00168656	0.00168656	0.328535	-3.04382	VVA down vs Control	21.4432	6.18925	2.30908	1
14379	7998664	NR_002327 // SNORA10 // small nucleolar RNA, H/ACA box 10 // 16p13.3 // 574042	SNORA10	NR_002327	0.0288233	0.0288233	0.328672	-3.04255	VVA down vs Control	7.07348	6.18463	6.99472	1
18627	8041204	NR_002327 // SNORA10 // small nucleolar RNA, H/ACA box 10 // 16p13.3 // 574042	SNORA10	NR_002327	0.0288233	0.0288233	0.328672	-3.04255	VVA down vs Control	7.07348	6.18463	6.99472	1
16328	8018972	AK057217 // TIMP2 // TIMP metallopeptidase inhibitor 2 // 17q25 // 7077	TIMP2	AK057217	0.0132136	0.0132136	0.329299	-3.03675	VVA down vs Control	10.0428	6.16343	4.90971	1
17470	8030067	NM_004605 // SULT2B1 // sulfotransferase family, cytosolic, 2B, member 1 // 19q1	SULT2B1	NM_004605	0.00796634	0.00796634	0.329524	-3.03468	VVA down vs Control	12.3172	6.15587	3.99821	1
9293	7947462	NM_145804 // ABTB2 // ankyrin repeat and BTB (POZ) domain containing 2 // 11p13	ABTB2	NM_145804	0.0491291	0.0491291	0.329962	-3.03065	VVA down vs Control	5.3697	6.14114	9.14933	1
11783	7972487	NM_015296 // DOCK9 // dedicator of cytokinesis 9 // 13q32.3 // 23348 /// NM_0011	DOCK9	NM_015296	0.00102537	0.00102537	0.330858	-3.02245	VVA down vs Control	25.2122	6.11116	1.93911	1
11543	7970381	NR_003366 // ANKRD20B // ankyrin repeat domain 20B // 2q11.1 // 729171 /// NM_00	ANKRD20B	NR_003366	0.0109162	0.0109162	0.332269	-3.00961	VVA down vs Control	10.8667	6.06421	4.46443	1
10797	7962274	NM_017641 // KIF21A // kinesin family member 21A // 12q12 // 55605 /// ENST00000	KIF21A	NM_017641	0.00454519	0.00454519	0.332898	-3.00392	VVA down vs Control	15.2093	6.04339	3.17878	1
14424	7999079	NM_001116 // ADCY9 // adenylate cyclase 9 // 16p13.3 // 115 /// ENST00000294016	ADCY9	NM_001116	0.0094459	0.0094459	0.3329	-3.00391	VVA down vs Control	11.5183	6.04335	4.1974	1
22306	8078227	NM_003884 // KAT2B // K(lysine) acetyltransferase 2B // 3p24 // 8850 /// ENST000	KAT2B	NM_003884	0.00621858	0.00621858	0.332905	-3.00386	VVA down vs Control	13.5429	6.04319	3.56981	1
22794	8083324	NM_014779 // TSC22D2 // TSC22 domain family, member 2 // 3q25.1 // 9819 /// ENST	TSC22D2	NM_014779	0.00300479	0.00300479	0.333033	-3.0027	VVA down vs Control	17.623	6.03894	2.74139	1
4695	7901675	NM_152607 // C1orf177 // chromosome 1 open reading frame 177 // 1p32.3 // 163747	C1orf177	NM_152607	0.0245837	0.0245837	0.333447	-2.99898	VVA down vs Control	7.63082	6.02531	6.31682	1
5057	7905220	NM_004425 // ECM1 // extracellular matrix protein 1 // 1q21 // 1893 /// NM_02266	ECM1	NM_004425	0.00243992	0.00243992	0.333722	-2.9965	VVA down vs Control	18.9367	6.01626	2.54163	1
11049	7965040	NM_007350 // PHLDA1 // pleckstrin homology-like domain, family A, member 1 // 12	PHLDA1	NM_007350	0.0155292	0.0155292	0.334068	-2.99341	VVA down vs Control	9.37746	6.00493	5.12287	1
18930	8044008	NM_003854 // IL1RL2 // interleukin 1 receptor-like 2 // 2q12 // 8808 /// ENST000	IL1RL2	NM_003854	0.00328491	0.00328491	0.334292	-2.99139	VVA down vs Control	17.0816	5.99756	2.8089	1
24153	8097098	NM_019050 // USP53 // ubiquitin specific peptidase 53 // 4q26 // 54532 /// ENST0	USP53	NM_019050	0.00175188	0.00175188	0.335427	-2.98128	VVA down vs Control	21.1744	5.96053	2.25198	1
20471	8060187	NM_001085437 // C2orf54 // chromosome 2 open reading frame 54 // 2q37.3 // 79919	C2orf54	NM_001085437	0.00524354	0.00524354	0.33579	-2.97805	VVA down vs Control	14.433	5.94873	3.2973	1
12998	7983761	---		---	0.00165861	0.00165861	0.337499	-2.96297	VVA down vs Control	21.5622	5.89352	2.18661	1
24308	8098470	NM_024949 // WWC2 // WW and C2 domain containing 2 // 4q35.1 // 80014 /// ENST00	WWC2	NM_024949	0.0115268	0.0115268	0.340144	-2.93993	VVA down vs Control	10.6278	5.80909	4.37276	1
7664	7931859	NM_017422 // CALML5 // calmodulin-like 5 // 10p15.1 // 51806 /// ENST00000380332	CALML5	NM_017422	0.0115908	0.0115908	0.341527	-2.92803	VVA down vs Control	10.6037	5.76548	4.34979	1
10494	7959131	NM_207311 // CCDC64 // coiled-coil domain containing 64 // 12q24.23 // 92558 ///	CCDC64	NM_207311	0.0038385	0.0038385	0.342605	-2.91881	VVA down vs Control	16.1644	5.73168	2.83669	1
11960	7973974	NM_006194 // PAX9 // paired box 9 // 14q12-q13 // 5083 /// ENST00000402703 // PA	PAX9	NM_006194	0.00712762	0.00712762	0.343407	-2.91199	VVA down vs Control	12.8581	5.70669	3.55055	1
7709	7932407	NM_001004470 // ST8SIA6 // ST8 alpha-N-acetyl-neuraminide alpha-2,8-sialyltransf	ST8SIA6	NM_001004470	0.00740692	0.00740692	0.343473	-2.91144	VVA down vs Control	12.6695	5.70464	3.60212	1
8717	7942118	NM_138768 // MYEOV // myeloma overexpressed (in a subset of t(11;14) positive mu	MYEOV	NM_138768	0.00260261	0.00260261	0.343521	-2.91103	VVA down vs Control	18.5221	5.70317	2.4633	1
16605	8021470	NM_021127 // PMAIP1 // phorbol-12-myristate-13-acetate-induced protein 1 // 18q2	PMAIP1	NM_021127	0.0215888	0.0215888	0.343994	-2.90703	VVA down vs Control	8.10362	5.68847	5.61573	1
4369	7898431	NM_016233 // PADI3 // peptidyl arginine deiminase, type III // 1p36.13 // 51702	PADI3	NM_016233	0.0483607	0.0483607	0.344059	-2.90648	VVA down vs Control	5.4166	5.68645	8.39856	1
25583	8110678	NM_145265 // CCDC127 // coiled-coil domain containing 127 // 5p15.33 // 133957 /	CCDC127	NM_145265	0.013253	0.013253	0.344672	-2.90131	VVA down vs Control	10.0303	5.66752	4.5203	1
15796	8013384	NM_000691 // ALDH3A1 // aldehyde dehydrogenase 3 family, memberA1 // 17p11.2 //	ALDH3A1	NM_000691	0.014203	0.014203	0.345257	-2.89639	VVA down vs Control	9.74187	5.64946	4.63932	1
32635	8176286	NM_018390 // PLCXD1 // phosphatidylinositol-specific phospholipase C, X domain c	PLCXD1	NM_018390	0.0441881	0.0441881	0.345487	-2.89446	VVA down vs Control	5.689	5.64239	7.93446	1
13858	7992967	NM_138440 // VASN // vasorin // 16p13.3 // 114990 /// ENST00000304735 // VASN //	VASN	NM_138440	0.00933501	0.00933501	0.346482	-2.88615	VVA down vs Control	11.5725	5.61189	3.87946	1
5324	7907830	NM_002826 // QSOX1 // quiescin Q6 sulfhydryl oxidase 1 // 1q24 // 5768 /// NM_00	QSOX1	NM_002826	0.00627487	0.00627487	0.347596	-2.8769	VVA down vs Control	13.4969	5.57797	3.30622	1
12219	7976698	NM_001008707 // EML1 // echinoderm microtubule associated protein like 1 // 14q3	EML1	NM_001008707	0.0134241	0.0134241	0.348975	-2.86553	VVA down vs Control	9.97649	5.53623	4.43942	1
12473	7979179	NM_014584 // ERO1L // ERO1-like (S. cerevisiae) // 14q22.1 // 30001 /// ENST0000	ERO1L	NM_014584	0.0262145	0.0262145	0.349382	-2.86219	VVA down vs Control	7.40298	5.52398	5.96946	1
19895	8053741	NR_003366 // ANKRD20B // ankyrin repeat domain 20B // 2q11.1 // 729171 /// NM_00	ANKRD20B	NR_003366	0.0269253	0.0269253	0.350091	-2.8564	VVA down vs Control	7.30922	5.50271	6.02276	1
19343	8047926	NM_002374 // MAP2 // microtubule-associated protein 2 // 2q34-q35 // 4133 /// NM	MAP2	NM_002374	0.0145216	0.0145216	0.350587	-2.85236	VVA down vs Control	9.65051	5.48788	4.5493	1
29773	8150076	NM_001394 // DUSP4 // dual specificity phosphatase 4 // 8p12-p11 // 1846 /// NM	DUSP4	NM_001394	0.0379221	0.0379221	0.351118	-2.84804	VVA down vs Control	6.16628	5.47203	7.0993	1
16081	8016215	NM_199282 // ARHGAP27 // Rho GTPase activating protein 27 // 17q21.31 // 201176	ARHGAP27	NM_199282	0.00305659	0.00305659	0.356907	-2.80185	VVA down vs Control	17.5183	5.30238	2.42142	1
31417	8165808	NM_001141919 // XG // Xg blood group // Xp22.33 // 7499 /// NM_175569 // XG // X	XG	NM_001141919	0.00780416	0.00780416	0.357536	-2.79692	VVA down vs Control	12.4161	5.28428	3.4048	1
28990	8143127	AY358803 // FAM180A // family with sequence similarity 180, member A // 7q33 //	FAM180A	AY358803	0.0314496	0.0314496	0.360281	-2.77561	VVA down vs Control	6.77786	5.20598	6.14469	1
29414	8146955	---		---	0.000267107	0.000267107	0.361402	-2.767	VVA down vs Control	38.1093	5.17434	1.08621	1
10894	7963406	NM_005555 // KRT6B // keratin 6B // 12q12-q13 // 3854 /// ENST00000252252 // KRT	KRT6B	NM_005555	0.0241545	0.0241545	0.3619	-2.76319	VVA down vs Control	7.69397	5.16033	5.36559	1
20737	8062927	NM_002638 // PI3 // peptidase inhibitor 3, skin-derived // 20q12-q13 // 5266 ///	PI3	NM_002638	0.0150456	0.0150456	0.362066	-2.76193	VVA down vs Control	9.50564	5.15569	4.33906	1
7904	7934145	NM_207119 // LRRC20 // leucine rich repeat containing 20 // 10q22.1 // 55222 ///	LRRC20	NM_207119	0.00503004	0.00503004	0.362598	-2.75788	VVA down vs Control	14.6559	5.1408	2.80614	1
6040	7915385	NM_001956 // EDN2 // endothelin 2 // 1p34 // 1907 /// ENST00000372587 // EDN2 //	EDN2	NM_001956	0.0104927	0.0104927	0.364606	-2.74268	VVA down vs Control	11.0425	5.08497	3.68392	1
18380	8038967	NM_001105549 // ZNF83 // zinc finger protein 83 // 19q13.3 // 55769 /// NM_00110	ZNF83	NM_001105549	0.000244118	0.000244118	0.366457	-2.72883	VVA down vs Control	39.1376	5.03406	1.029	1
25419	8109141	NM_152407 // GRPEL2 // GrpE-like 2, mitochondrial (E. coli) // 5q32 // 134266 //	GRPEL2	NM_152407	0.0110311	0.0110311	0.36667	-2.72725	VVA down vs Control	10.8205	5.02825	3.71758	1
30997	8161558	NR_002817 // AQP7P1 // aquaporin 7 pseudogene 1 // 9q13 // 375719 /// NR_002823	AQP7P1	NR_002817	0.0230841	0.0230841	0.36753	-2.72086	VVA down vs Control	7.85787	5.00478	5.0953	1
9910	7953021	NM_024551 // ADIPOR2 // adiponectin receptor 2 // 12p13.31 // 79602 /// ENST0000	ADIPOR2	NM_024551	0.00502117	0.00502117	0.367919	-2.71799	VVA down vs Control	14.6654	4.99422	2.72435	1
13688	7991283	NM_016321 // RHCG // Rh family, C glycoprotein // 15q25 // 51458 /// ENST0000026	RHCG	NM_016321	0.0019213	0.0019213	0.368104	-2.71663	VVA down vs Control	20.5319	4.9892	1.94398	1
26803	8122058	NM_000045 // ARG1 // arginase, liver // 6q23 // 383 /// ENST00000368087 // ARG1	ARG1	NM_000045	0.0209782	0.0209782	0.368623	-2.7128	VVA down vs Control	8.21024	4.97514	4.84774	1
31410	8165711	NM_018390 // PLCXD1 // phosphatidylinositol-specific phospholipase C, X domain c	PLCXD1	NM_018390	0.0379039	0.0379039	0.369429	-2.70688	VVA down vs Control	6.16781	4.95337	6.42481	1
30393	8155591	NR_003366 // ANKRD20B // ankyrin repeat domain 20B // 2q11.1 // 729171 /// ENST0	ANKRD20B	NR_003366	0.0304021	0.0304021	0.370926	-2.69596	VVA down vs Control	6.89188	4.91323	5.7032	1
22833	8083709	NM_005496 // SMC4 // structural maintenance of chromosomes 4 // 3q26.1 // 10051	SMC4	NM_005496	0.0169235	0.0169235	0.370955	-2.69575	VVA down vs Control	9.03436	4.91247	4.35003	1
5446	7909285	NM_006212 // PFKFB2 // 6-phosphofructo-2-kinase/fructose-2,6-biphosphatase 2 //	PFKFB2	NM_006212	0.00912775	0.00912775	0.371147	-2.69435	VVA down vs Control	11.6762	4.90734	3.3623	1
9588	7950062	---		---	0.00263585	0.00263585	0.371358	-2.69282	VVA down vs Control	18.4413	4.9017	2.1264	1
19630	8051187	NM_022823 // FNDC4 // fibronectin type III domain containing 4 // 2p23.3 // 6483	FNDC4	NM_022823	0.00399355	0.00399355	0.371561	-2.69135	VVA down vs Control	15.9369	4.8963	2.45784	1
4485	7899480	NR_002907 // SNORA73A // small nucleolar RNA, H/ACA box 73A // 1p36.1 // 6080 //	SNORA73A	NR_002907	0.030185	0.030185	0.373559	-2.67696	VVA down vs Control	6.91614	4.84341	5.60244	1
8756	7942544	NM_001098638 // RNF169 // ring finger protein 169 // 11q13.4 // 254225 /// ENST0	RNF169	NM_001098638	0.0073387	0.0073387	0.374125	-2.6729	VVA down vs Control	12.7148	4.82851	3.03805	1
12405	7978449	NM_015382 // HECTD1 // HECT domain containing 1 // 14q12 // 25831 /// ENST000003	HECTD1	NM_015382	0.00167429	0.00167429	0.374493	-2.67028	VVA down vs Control	21.4951	4.81887	1.79348	1
10021	7954090	NM_001423 // EMP1 // epithelial membrane protein 1 // 12p12.3 // 2012 /// ENST00	EMP1	NM_001423	0.00405553	0.00405553	0.374978	-2.66682	VVA down vs Control	15.849	4.80616	2.42597	1
7934	7934442	NM_001114133 // SYNPO2L // synaptopodin 2-like // 10q22.2 // 79933 /// NM_024875	SYNPO2L	NM_001114133	0.0151232	0.0151232	0.375848	-2.66065	VVA down vs Control	9.4847	4.78348	4.03469	1
15185	8006736	NM_007026 // DUSP14 // dual specificity phosphatase 14 // 17q12 // 11072 /// ENS	DUSP14	NM_007026	0.0291461	0.0291461	0.376507	-2.65599	VVA down vs Control	7.03534	4.76636	5.41991	1
26998	8123989	NM_005493 // RANBP9 // RAN binding protein 9 // 6p23 // 10048 /// ENST0000001161	RANBP9	NM_005493	0.00135679	0.00135679	0.376691	-2.6547	VVA down vs Control	23.033	4.76161	1.65384	1
21146	8066822	NM_018837 // SULF2 // sulfatase 2 // 20q12-q13.2 // 55959 /// NM_198596 // SULF2	SULF2	NM_018837	0.00317412	0.00317412	0.377826	-2.64672	VVA down vs Control	17.2886	4.7323	2.18979	1
26407	8118061	NM_080870 // DPCR1 // diffuse panbronchiolitis critical region 1 // 6p21.33 // 1	DPCR1	NM_080870	0.00128565	0.00128565	0.377885	-2.64631	VVA down vs Control	23.4403	4.73079	1.61459	1
7449	7929478	NM_000769 // CYP2C19 // cytochrome P450, family 2, subfamily C, polypeptide 19 /	CYP2C19	NM_000769	0.0227124	0.0227124	0.378564	-2.64156	VVA down vs Control	7.91703	4.71335	4.76275	1
573	7893075	---		---	0.0150048	0.0150048	0.379227	-2.63695	VVA down vs Control	9.51669	4.69639	3.94792	1
30770	8159624	BC052297 // C9orf169 // chromosome 9 open reading frame 169 // 9q34.3 // 375791	C9orf169	BC052297	0.00390726	0.00390726	0.379409	-2.63568	VVA down vs Control	16.0621	4.69174	2.3368	1
11102	7965606	NM_002108 // HAL // histidine ammonia-lyase // 12q22-q24.1 // 3034 /// ENST00000	HAL	NM_002108	0.0123716	0.0123716	0.379748	-2.63332	VVA down vs Control	10.3223	4.68309	3.6295	1
5101	7905544	NM_005987 // SPRR1A // small proline-rich protein 1A // 1q21-q22 // 6698 /// ENS	SPRR1A	NM_005987	0.00941353	0.00941353	0.380988	-2.62476	VVA down vs Control	11.534	4.65162	3.22636	1
13011	7983890	NM_001018100 // GCOM1 // GRINL1A complex locus // 15q21.3 // 145781 /// NM_15245	GCOM1	NM_001018100	0.00444739	0.00444739	0.38106	-2.62426	VVA down vs Control	15.33	4.6498	2.42651	1
33030	8179716	NM_001264 // CDSN // corneodesmosin // 6p21.3 // 1041 /// ENST00000445893 // CDS	CDSN	NM_001264	0.027948	0.027948	0.381577	-2.6207	VVA down vs Control	7.17968	4.63673	5.1665	1
30358	8155414	NR_003366 // ANKRD20B // ankyrin repeat domain 20B // 2q11.1 // 729171 /// NM_00	ANKRD20B	NR_003366	0.0303714	0.0303714	0.384238	-2.60256	VVA down vs Control	6.8953	4.57009	5.30227	1
30957	8161384	NR_003366 // ANKRD20B // ankyrin repeat domain 20B // 2q11.1 // 729171 /// NM_00	ANKRD20B	NR_003366	0.0303714	0.0303714	0.384238	-2.60256	VVA down vs Control	6.8953	4.57009	5.30227	1
32510	8175288	NM_019556 // MOSPD1 // motile sperm domain containing 1 // Xq26.3 // 56180 /// E	MOSPD1	NM_019556	0.0101929	0.0101929	0.384681	-2.59956	VVA down vs Control	11.1724	4.55909	3.26452	1
23808	8093961	NM_014743 // KIAA0232 // KIAA0232 // 4p16.1 // 9778 /// NM_001100590 // KIAA0232	KIAA0232	NM_014743	0.00254596	0.00254596	0.385752	-2.59234	VVA down vs Control	18.6627	4.53258	1.94295	1
4284	7897522	NM_052960 // RBP7 // retinol binding protein 7, cellular // 1p36.22 // 116362 //	RBP7	NM_052960	0.0116616	0.0116616	0.386724	-2.58582	VVA down vs Control	10.5772	4.50866	3.41011	1
29825	8150529	NM_014420 // DKK4 // dickkopf homolog 4 (Xenopus laevis) // 8p11.2-p11.1 // 2712	DKK4	NM_014420	0.0451295	0.0451295	0.387409	-2.58125	VVA down vs Control	5.62476	4.49188	6.38873	1
30296	8154848	NM_007343 // PRSS3 // protease, serine, 3 // 9p11.2 // 5646 /// NM_002771 // PRS	PRSS3	NM_007343	0.00803241	0.00803241	0.388164	-2.57623	VVA down vs Control	12.2777	4.47347	2.91486	1
16511	8020495	NM_138375 // CABLES1 // Cdk5 and Abl enzyme substrate 1 // 18q11.2 // 91768 ///	CABLES1	NM_138375	0.00639079	0.00639079	0.388323	-2.57518	VVA down vs Control	13.4039	4.4696	2.66765	1
30985	8161488	NR_002817 // AQP7P1 // aquaporin 7 pseudogene 1 // 9q13 // 375719 /// NM_001170	AQP7P1	NR_002817	0.0250658	0.0250658	0.388726	-2.5725	VVA down vs Control	7.56153	4.45978	4.71839	1
29531	8148184	NM_032899 // FAM83A // family with sequence similarity 83, member A // 8q24.13 /	FAM83A	NM_032899	0.00703508	0.00703508	0.388845	-2.57172	VVA down vs Control	12.9227	4.4569	2.75912	1
4108	7896634	---		---	0.0324511	0.0324511	0.389395	-2.56809	VVA down vs Control	6.67326	4.44357	5.32701	1
7474	7929677	NM_018425 // PI4K2A // phosphatidylinositol 4-kinase type 2 alpha // 10q24 // 55	PI4K2A	NM_018425	0.0152296	0.0152296	0.390199	-2.56279	VVA down vs Control	9.45627	4.42415	3.74283	1
19894	8053737	NR_003366 // ANKRD20B // ankyrin repeat domain 20B // 2q11.1 // 729171 /// NM_00	ANKRD20B	NR_003366	0.029394	0.029394	0.391019	-2.55742	VVA down vs Control	7.00641	4.40445	5.02905	1
11466	7969493	NM_144777 // SCEL // sciellin // 13q22 // 8796 /// NM_003843 // SCEL // sciellin	SCEL	NM_144777	0.00562549	0.00562549	0.391261	-2.55584	VVA down vs Control	14.0613	4.39864	2.50255	1
30362	8155442	NR_002817 // AQP7P1 // aquaporin 7 pseudogene 1 // 9q13 // 375719 /// NM_001170	AQP7P1	NR_002817	0.023506	0.023506	0.391543	-2.554	VVA down vs Control	7.79214	4.39188	4.50904	1
15430	8009241	NR_004380 // SNORD104 // small nucleolar RNA, C/D box 104 // 17q23.3 // 692227	SNORD104	NR_004380	0.0223942	0.0223942	0.391845	-2.55203	VVA down vs Control	7.96868	4.38466	4.4019	1
16619	8021603	NM_012397 // SERPINB13 // serpin peptidase inhibitor, clade B (ovalbumin), membe	SERPINB13	NM_012397	0.0107701	0.0107701	0.391959	-2.55129	VVA down vs Control	10.9264	4.38194	3.20834	1
13860	7992987	NM_001127204 // HMOX2 // heme oxygenase (decycling) 2 // 16p13.3 // 3163 /// NM	HMOX2	NM_001127204	0.0078263	0.0078263	0.392105	-2.55033	VVA down vs Control	12.4024	4.37845	2.82425	1
15013	8005235	NM_201274 // MPRIP // myosin phosphatase Rho interacting protein // 17p11.2 // 2	MPRIP	NM_201274	0.00405555	0.00405555	0.392511	-2.5477	VVA down vs Control	15.849	4.36879	2.2052	1
17358	8028955	NR_001278 // CYP2B7P1 // cytochrome P450, family 2, subfamily B, polypeptide 7 p	CYP2B7P1	NR_001278	0.0278374	0.0278374	0.393117	-2.54377	VVA down vs Control	7.1934	4.35438	4.84264	1
30744	8159318	NM_001145638 // GPSM1 // G-protein signaling modulator 1 (AGS3-like, C. elegans)	GPSM1	NM_001145638	0.0237364	0.0237364	0.393692	-2.54005	VVA down vs Control	7.75687	4.34075	4.47681	1
16547	8020903	NM_020474 // GALNT1 // UDP-N-acetyl-alpha-D-galactosamine:polypeptide N-acetylga	GALNT1	NM_020474	0.00523223	0.00523223	0.394631	-2.53401	VVA down vs Control	14.4445	4.31861	2.39183	1
19764	8052669	NM_014755 // SERTAD2 // SERTA domain containing 2 // 2p14 // 9792 /// ENST000003	SERTAD2	NM_014755	0.0153397	0.0153397	0.397831	-2.51363	VVA down vs Control	9.42707	4.24392	3.60147	1
24674	8101904	NM_000673 // ADH7 // alcohol dehydrogenase 7 (class IV), mu or sigma polypeptide	ADH7	NM_000673	0.0145332	0.0145332	0.398534	-2.50919	VVA down vs Control	9.64723	4.22766	3.5058	1
30387	8155554	NR_002817 // AQP7P1 // aquaporin 7 pseudogene 1 // 9q13 // 375719 /// NM_001170	AQP7P1	NR_002817	0.0249314	0.0249314	0.400211	-2.49868	VVA down vs Control	7.58068	4.18917	4.42089	1
4395	7898663	NM_032409 // PINK1 // PTEN induced putative kinase 1 // 1p36 // 65018 /// ENST00	PINK1	NM_032409	0.00799499	0.00799499	0.400279	-2.49826	VVA down vs Control	12.3	4.1876	2.72364	1
25417	8109093	NM_014945 // ABLIM3 // actin binding LIM protein family, member 3 // 5q32 // 228	ABLIM3	NM_014945	0.0260923	0.0260923	0.400485	-2.49698	VVA down vs Control	7.41944	4.18291	4.51022	1
26067	8115210	NM_006058 // TNIP1 // TNFAIP3 interacting protein 1 // 5q32-q33.1 // 10318 /// E	TNIP1	NM_006058	0.0102009	0.0102009	0.401242	-2.49226	VVA down vs Control	11.1689	4.16565	2.98374	1
6056	7915543	NM_201649 // SLC6A9 // solute carrier family 6 (neurotransmitter transporter, gl	SLC6A9	NM_201649	0.0141759	0.0141759	0.401832	-2.4886	VVA down vs Control	9.74976	4.15225	3.40706	1
3435	7895955	---		---	0.000154824	0.000154824	0.402851	-2.48231	VVA down vs Control	44.7113	4.12923	0.738825	1
27120	8124862	NM_001264 // CDSN // corneodesmosin // 6p21.3 // 1041 /// ENST00000445893 // CDS	CDSN	NM_001264	0.0290549	0.0290549	0.402954	-2.48167	VVA down vs Control	7.04606	4.1269	4.68563	1
17352	8028908	NM_025194 // ITPKC // inositol 1,4,5-trisphosphate 3-kinase C // 19q13.1 // 8027	ITPKC	NM_025194	0.0208365	0.0208365	0.405597	-2.4655	VVA down vs Control	8.23555	4.06775	3.9514	1
9772	7951686	NM_001562 // IL18 // interleukin 18 (interferon-gamma-inducing factor) // 11q22.	IL18	NM_001562	0.0160876	0.0160876	0.406369	-2.46082	VVA down vs Control	9.23555	4.05063	3.50873	1
33279	8180397	---		---	0.0255805	0.0255805	0.406803	-2.45819	VVA down vs Control	7.48937	4.04102	4.31654	1
30155	8153426	NM_001100878 // C8orf73 // chromosome 8 open reading frame 73 // 8q24.3 // 64247	C8orf73	NM_001100878	0.0075884	0.0075884	0.407855	-2.45185	VVA down vs Control	12.5517	4.01785	2.56084	1
16980	8025058	NM_004240 // TRIP10 // thyroid hormone receptor interactor 10 // 19p13.3 // 9322	TRIP10	NM_004240	0.00561336	0.00561336	0.408209	-2.44973	VVA down vs Control	14.0726	4.01008	2.27965	1
32901	8178442	NM_001264 // CDSN // corneodesmosin // 6p21.3 // 1041 /// ENST00000445893 // CDS	CDSN	NM_001264	0.0306337	0.0306337	0.409591	-2.44146	VVA down vs Control	6.86625	3.97988	4.63704	1
32977	8179221	NM_080870 // DPCR1 // diffuse panbronchiolitis critical region 1 // 6p21.33 // 1	DPCR1	NM_080870	0.00185769	0.00185769	0.410549	-2.43576	VVA down vs Control	20.7645	3.95907	1.52532	1
18979	8044552	NM_173170 // IL1F5 // interleukin 1 family, member 5 (delta) // 2q14 // 26525 //	IL1F5	NM_173170	0.0094188	0.0094188	0.410639	-2.43523	VVA down vs Control	11.5315	3.95714	2.74528	1
16772	8022996	NM_020964 // KIAA1632 // KIAA1632 // 18q12.3-q21.1 // 57724 /// ENST00000282041	KIAA1632	NM_020964	0.0137983	0.0137983	0.410889	-2.43375	VVA down vs Control	9.86168	3.95172	3.20572	1
25490	8109773	NM_001161661 // WWC1 // WW and C2 domain containing 1 // 5q34 // 23286 /// NM_00	WWC1	NM_001161661	0.0163139	0.0163139	0.411118	-2.43239	VVA down vs Control	9.17982	3.94678	3.43953	1
29694	8149324	NM_053279 // FAM167A // family with sequence similarity 167, member A // 8p23-p2	FAM167A	NM_053279	0.00327106	0.00327106	0.412379	-2.42496	VVA down vs Control	17.107	3.91963	1.83299	1
30962	8161415	NR_003366 // ANKRD20B // ankyrin repeat domain 20B // 2q11.1 // 729171 /// ENST0	ANKRD20B	NR_003366	0.0247313	0.0247313	0.412575	-2.4238	VVA down vs Control	7.60943	3.91543	4.11639	1
16697	8022378	---		---	0.0374826	0.0374826	0.414195	-2.41432	VVA down vs Control	6.20347	3.88082	5.0047	1
16621	8021623	NM_003784 // SERPINB7 // serpin peptidase inhibitor, clade B (ovalbumin), member	SERPINB7	NM_003784	0.0157803	0.0157803	0.415054	-2.40932	VVA down vs Control	9.31285	3.8626	3.31809	1
4880	7903565	NM_013296 // GPSM2 // G-protein signaling modulator 2 (AGS3-like, C. elegans) //	GPSM2	NM_013296	0.0278854	0.0278854	0.415479	-2.40686	VVA down vs Control	7.18744	3.85362	4.28928	1
9421	7948397	---		---	0.0174483	0.0174483	0.415667	-2.40577	VVA down vs Control	8.91433	3.84964	3.45479	1
27599	8130071	NM_024713 // C15orf29 // chromosome 15 open reading frame 29 // 15q14 // 79768 /	C15orf29	NM_024713	0.00604923	0.00604923	0.416806	-2.3992	VVA down vs Control	13.6844	3.8257	2.23654	1
15801	8013465	NM_005557 // KRT16 // keratin 16 // 17q12-q21 // 3868 /// ENST00000301653 // KRT	KRT16	NM_005557	0.0010514	0.0010514	0.417206	-2.39689	VVA down vs Control	25.0108	3.8173	1.22101	1
24388	8099218	NM_020416 // PPP2R2C // protein phosphatase 2 (formerly 2A), regulatory subunit	PPP2R2C	NM_020416	0.0080707	0.0080707	0.418824	-2.38764	VVA down vs Control	12.255	3.78357	2.46991	1
15517	8010139	NM_001143998 // SEC14L1 // SEC14-like 1 (S. cerevisiae) // 17q25.1-q25.2 // 6397	SEC14L1	NM_001143998	0.0211514	0.0211514	0.419481	-2.3839	VVA down vs Control	8.17961	3.76996	3.68718	1
17359	8028963	NM_000767 // CYP2B6 // cytochrome P450, family 2, subfamily B, polypeptide 6 //	CYP2B6	NM_000767	0.017399	0.017399	0.420093	-2.38043	VVA down vs Control	8.92542	3.75732	3.36775	1
5900	7913938	NM_001039775 // AIM1L // absent in melanoma 1-like // 1p36.11 // 55057 /// ENST0	AIM1L	NM_001039775	0.00646404	0.00646404	0.420478	-2.37825	VVA down vs Control	13.349	3.7494	2.247	1
19791	8052872	NM_003236 // TGFA // transforming growth factor, alpha // 2p13 // 7039 /// NM_00	TGFA	NM_003236	0.0302561	0.0302561	0.421734	-2.37117	VVA down vs Control	6.90817	3.72362	4.31214	1
23801	8093878	NM_153717 // EVC // Ellis van Creveld syndrome // 4p16 // 2121 /// ENST000003826	EVC	NM_153717	0.0285084	0.0285084	0.421881	-2.37034	VVA down vs Control	7.11121	3.72061	4.18563	1
17786	8033043	NM_000150 // FUT6 // fucosyltransferase 6 (alpha (1,3) fucosyltransferase) // 19	FUT6	NM_000150	0.0251323	0.0251323	0.422628	-2.36614	VVA down vs Control	7.55209	3.70536	3.92512	1
21221	8067662	NM_005975 // PTK6 // PTK6 protein tyrosine kinase 6 // 20q13.3 // 5753 /// ENST0	PTK6	NM_005975	0.00778981	0.00778981	0.423465	-2.36147	VVA down vs Control	12.425	3.68837	2.37482	1
24018	8095736	NM_001657 // AREG // amphiregulin // 4q13-q21 // 374 /// BC009799 // AREG // amp	AREG	NM_001657	0.0333944	0.0333944	0.42486	-2.35371	VVA down vs Control	6.57841	3.66018	4.45114	1
9907	7952986	NM_213655 // HSN2 // hereditary sensory neuropathy, type II // 12p13.3 // 378465	HSN2	NM_213655	0.000622389	0.000622389	0.425781	-2.34863	VVA down vs Control	29.4988	3.64169	0.987617	1
28170	8135514	NM_001550 // IFRD1 // interferon-related developmental regulator 1 // 7q22-q31 /	IFRD1	NM_001550	0.0463615	0.0463615	0.426388	-2.34528	VVA down vs Control	5.54321	3.62954	5.23818	1
23067	8085984	NM_017784 // OSBPL10 // oxysterol binding protein-like 10 // 3p22.3 // 114884 //	OSBPL10	NM_017784	0.000683529	0.000683529	0.427261	-2.34049	VVA down vs Control	28.6527	3.61215	1.00853	1
12930	7982938	NM_006293 // TYRO3 // TYRO3 protein tyrosine kinase // 15q15.1-q21.1 // 7301 ///	TYRO3	NM_006293	0.010054	0.010054	0.427518	-2.33908	VVA down vs Control	11.2343	3.60705	2.56859	1
33278	8180396	---		---	0.0239295	0.0239295	0.428932	-2.33137	VVA down vs Control	7.72766	3.57907	3.70521	1
15206	8006968	NM_178171 // GSDMA // gasdermin A // 17q21.1 // 284110 /// ENST00000301659 // GS	GSDMA	NM_178171	0.00834431	0.00834431	0.428967	-2.33118	VVA down vs Control	12.0964	3.57838	2.36658	1
16573	8021183	NR_003003 // SCARNA17 // small Cajal body-specific RNA 17 // 18q21.1 // 677769	SCARNA17	NR_003003	0.0130122	0.0130122	0.429802	-2.32665	VVA down vs Control	10.1076	3.56196	2.82203	1
3624	7896146	---		---	0.0251358	0.0251358	0.430594	-2.32237	VVA down vs Control	7.5516	3.54644	3.75702	1

Column #	Transcript Cluster ID	gene_assignment	Gene Symbol	RefSeq	p-value(Group)	p-value(VVA vs. Control)	Ratio(VVA vs. Control)	Fold-Change(VVA vs. Control)	Fold-Change(VVA vs. Control) (Description)	F(Group)	SS(Group)	SS(Error)	F(Error)
19595	8050719	NM_006277 // ITSN2 // intersectin 2 // 2pter-p25.1 // 50618 /// NM_019595 // ITS	ITSN2	NM_006277	0.0100194	0.0100194	0.430604	-2.32232	VVA down vs Control	11.2499	3.54624	2.5218	1
23809	8093974	---		---	0.00117812	0.00117812	0.431213	-2.31904	VVA down vs Control	24.1125	3.53435	1.17262	1
18160	8036913	NM_000713 // BLVRB // biliverdin reductase B (flavin reductase (NADPH)) // 19q13	BLVRB	NM_000713	0.0262854	0.0262854	0.431487	-2.31757	VVA down vs Control	7.3935	3.52902	3.81851	1
22395	8079140	NM_017719 // SNRK // SNF related kinase // 3p22.1 // 54861 /// NM_001100594 // S	SNRK	NM_017719	0.0025429	0.0025429	0.431593	-2.317	VVA down vs Control	18.6704	3.52696	1.51125	1
27671	8130803	AK127120 // LOC401286 // hypothetical LOC401286 // 6q27 // 401286 /// ENST000003	LOC401286	AK127120	0.0162537	0.0162537	0.433391	-2.30739	VVA down vs Control	9.19453	3.49215	3.03846	1
27687	8130976	AK127120 // LOC401286 // hypothetical LOC401286 // 6q27 // 401286 /// ENST000003	LOC401286	AK127120	0.0162537	0.0162537	0.433391	-2.30739	VVA down vs Control	9.19453	3.49215	3.03846	1
4758	7902367	NM_000016 // ACADM // acyl-Coenzyme A dehydrogenase, C-4 to C-12 straight chain	ACADM	NM_000016	0.0138068	0.0138068	0.434141	-2.3034	VVA down vs Control	9.85913	3.47772	2.82193	1
11216	7966839	NM_019086 // FLJ20674 // hypothetical protein FLJ20674 // 12q24.23 // 54621 ///	FLJ20674	NM_019086	0.00832013	0.00832013	0.434641	-2.30075	VVA down vs Control	12.1101	3.46813	2.29106	1
8369	7938812	NM_153347 // TMEM86A // transmembrane protein 86A // 11p15.1 // 144110 /// ENST0	TMEM86A	NM_153347	0.00714495	0.00714495	0.434986	-2.29893	VVA down vs Control	12.8462	3.46153	2.15568	1
13346	7987180	NM_024713 // C15orf29 // chromosome 15 open reading frame 29 // 15q14 // 79768 /	C15orf29	NM_024713	0.00374177	0.00374177	0.437024	-2.2882	VVA down vs Control	16.3122	3.42277	1.67862	1
15694	8012349	NM_002616 // PER1 // period homolog 1 (Drosophila) // 17p13.1-p12 // 5187 /// EN	PER1	NM_002616	0.0162062	0.0162062	0.437076	-2.28793	VVA down vs Control	9.20622	3.42178	2.97345	1
17768	8032804	NM_003025 // SH3GL1 // SH3-domain GRB2-like 1 // 19p13.3 // 6455 /// ENST0000026	SH3GL1	NM_003025	0.0120461	0.0120461	0.437171	-2.28743	VVA down vs Control	10.4368	3.41997	2.62147	1
20440	8059854	NM_005737 // ARL4C // ADP-ribosylation factor-like 4C // 2q37.1 // 10123 /// ENS	ARL4C	NM_005737	0.0119385	0.0119385	0.437488	-2.28577	VVA down vs Control	10.4755	3.41398	2.60721	1
4199	7896725	---		---	0.0213948	0.0213948	0.437935	-2.28344	VVA down vs Control	8.13708	3.40557	3.3482	1
8196	7937443	NM_022772 // EPS8L2 // EPS8-like 2 // 11p15.5 // 64787 /// ENST00000318562 // EP	EPS8L2	NM_022772	0.00468576	0.00468576	0.437978	-2.28322	VVA down vs Control	15.0415	3.40475	1.81085	1
15058	8005638	NM_001031806 // ALDH3A2 // aldehyde dehydrogenase 3 family, member A2 // 17p11.2	ALDH3A2	NM_001031806	0.0024486	0.0024486	0.4383	-2.28154	VVA down vs Control	18.9137	3.3987	1.43756	1
13447	7988581	NM_014701 // SECISBP2L // SECIS binding protein 2-like // 15q21.1 // 9728 /// EN	SECISBP2L	NM_014701	0.0149586	0.0149586	0.438408	-2.28098	VVA down vs Control	9.52923	3.39667	2.85158	1
18958	8044301	NM_023016 // ANKRD57 // ankyrin repeat domain 57 // 2q13 // 65124 /// ENST000003	ANKRD57	NM_023016	0.016331	0.016331	0.438454	-2.28074	VVA down vs Control	9.17564	3.39579	2.9607	1
2168	7894679	---		---	0.00908398	0.00908398	0.438677	-2.27958	VVA down vs Control	11.6984	3.3916	2.31936	1
8103	7936463	NM_002313 // ABLIM1 // actin binding LIM protein 1 // 10q25 // 3983 /// NM_00100	ABLIM1	NM_002313	0.00086509	0.00086509	0.439463	-2.27551	VVA down vs Control	26.6117	3.3769	1.01516	1
8354	7938687	NM_005013 // NUCB2 // nucleobindin 2 // 11p15.1-p14 // 4925 /// ENST00000323688	NUCB2	NM_005013	0.0277691	0.0277691	0.439664	-2.27447	VVA down vs Control	7.2019	3.37314	3.74694	1
7528	7930276	NM_014720 // SLK // STE20-like kinase (yeast) // 10q24.33 // 9748 /// ENST000003	SLK	NM_014720	0.00135061	0.00135061	0.440003	-2.27271	VVA down vs Control	23.0674	3.36682	1.16765	1
17240	8027604	NM_014686 // KIAA0355 // KIAA0355 // 19q13.11 // 9710 /// ENST00000299505 // KIA	KIAA0355	NM_014686	0.00914832	0.00914832	0.440609	-2.26959	VVA down vs Control	11.6657	3.35554	2.30112	1
28796	8141140	NM_005221 // DLX5 // distal-less homeobox 5 // 7q22 // 1749 /// ENST00000222598	DLX5	NM_005221	0.0424318	0.0424318	0.442354	-2.26063	VVA down vs Control	5.81366	3.32325	4.57303	1
9820	7952249	NM_004205 // USP2 // ubiquitin specific peptidase 2 // 11q23.3 // 9099 /// NM_17	USP2	NM_004205	0.0483928	0.0483928	0.442487	-2.25995	VVA down vs Control	5.41462	3.3208	4.90642	1
18123	8036584	NM_002307 // LGALS7 // lectin, galactoside-binding, soluble, 7 // 19q13.2 // 396	LGALS7	NM_002307	0.0134532	0.0134532	0.442764	-2.25854	VVA down vs Control	9.96744	3.3157	2.66122	1
31167	8163149	NM_002829 // PTPN3 // protein tyrosine phosphatase, non-receptor type 3 // 9q31	PTPN3	NM_002829	0.0114674	0.0114674	0.443006	-2.25731	VVA down vs Control	10.6503	3.31126	2.48725	1
19340	8047854	NM_001142300 // CCNYL1 // cyclin Y-like 1 // 2q33.3 // 151195 /// NM_152523 // C	CCNYL1	NM_001142300	0.0136217	0.0136217	0.443539	-2.25459	VVA down vs Control	9.91537	3.30148	2.66372	1
16296	8018579	NM_001988 // EVPL // envoplakin // 17q25 // 2125 /// ENST00000301607 // EVPL //	EVPL	NM_001988	0.00279024	0.00279024	0.444393	-2.25026	VVA down vs Control	18.0824	3.28588	1.45373	1
12092	7975412	NM_015351 // TTC9 // tetratricopeptide repeat domain 9 // 14q24.2 // 23508 /// E	TTC9	NM_015351	0.0282185	0.0282185	0.444872	-2.24784	VVA down vs Control	7.14641	3.27716	3.66859	1
18923	8043909	NM_002518 // NPAS2 // neuronal PAS domain protein 2 // 2q11.2 // 4862 /// ENST00	NPAS2	NM_002518	0.019037	0.019037	0.445683	-2.24375	VVA down vs Control	8.57708	3.26243	3.04293	1
22830	8083690	NM_000882 // IL12A // interleukin 12A (natural killer cell stimulatory factor 1,	IL12A	NM_000882	0.0250904	0.0250904	0.447021	-2.23703	VVA down vs Control	7.55803	3.23828	3.42764	1
20114	8055688	NM_005168 // RND3 // Rho family GTPase 3 // 2q23.3 // 390 /// ENST00000375734 //	RND3	NM_005168	0.0355395	0.0355395	0.44706	-2.23683	VVA down vs Control	6.37485	3.23756	4.06292	1
24730	8102518	NM_001001701 // C4orf3 // chromosome 4 open reading frame 3 // 4q26 // 401152 //	C4orf3	NM_001001701	0.0113787	0.0113787	0.447125	-2.23651	VVA down vs Control	10.6842	3.2364	2.42331	1
9009	7944931	NM_198277 // SLC37A2 // solute carrier family 37 (glycerol-3-phosphate transport	SLC37A2	NM_198277	0.0253207	0.0253207	0.4476	-2.23414	VVA down vs Control	7.52556	3.22788	3.43138	1
8101	7936439	NM_001001936 // AFAP1L2 // actin filament associated protein 1-like 2 // 10q25.3	AFAP1L2	NM_001001936	0.0079925	0.0079925	0.447771	-2.23329	VVA down vs Control	12.3015	3.22481	2.09718	1
5103	7905553	NM_003125 // SPRR1B // small proline-rich protein 1B (cornifin) // 1q21-q22 // 6	SPRR1B	NM_003125	0.0382443	0.0382443	0.447871	-2.23279	VVA down vs Control	6.13935	3.22301	4.19981	1
26884	8122856	---		---	0.0124517	0.0124517	0.448072	-2.23178	VVA down vs Control	10.2947	3.21941	2.5018	1
30315	8155048	NM_014806 // RUSC2 // RUN and SH3 domain containing 2 // 9p13.3 // 9853 /// NM_0	RUSC2	NM_014806	0.02259	0.02259	0.449357	-2.2254	VVA down vs Control	7.93679	3.19648	3.22193	1
6537	7920220	NM_052891 // PGLYRP3 // peptidoglycan recognition protein 3 // 1q21 // 114771 //	PGLYRP3	NM_052891	0.0111322	0.0111322	0.44942	-2.22509	VVA down vs Control	10.7803	3.19537	2.37127	1
7288	7927747	NM_014951 // ZNF365 // zinc finger protein 365 // 10q21.2 // 22891 /// NM_199451	ZNF365	NM_014951	0.00845501	0.00845501	0.44958	-2.2243	VVA down vs Control	12.034	3.19252	2.12232	1
4681	7901497	NM_001004339 // ZYG11A // zyg-11 homolog A (C. elegans) // 1p32.3 // 440590 ///	ZYG11A	NM_001004339	0.00548451	0.00548451	0.449632	-2.22404	VVA down vs Control	14.1947	3.19159	1.79875	1
24495	8100231	NM_003215 // TEC // tec protein tyrosine kinase // 4p12 // 7006 /// ENST00000381	TEC	NM_003215	0.0160162	0.0160162	0.449989	-2.22228	VVA down vs Control	9.25334	3.18526	2.75383	1
5881	7913768	NM_021258 // IL22RA1 // interleukin 22 receptor, alpha 1 // 1p36.11 // 58985 ///	IL22RA1	NM_021258	0.0172109	0.0172109	0.45077	-2.21842	VVA down vs Control	8.96805	3.17144	2.8291	1
31261	8164217	NM_022833 // FAM129B // family with sequence similarity 129, member B // 9q33.3-	FAM129B	NM_022833	0.0147105	0.0147105	0.451598	-2.21436	VVA down vs Control	9.59754	3.15685	2.63138	1
30066	8152453	NM_014112 // TRPS1 // trichorhinophalangeal syndrome I // 8q24.12 // 7227 /// EN	TRPS1	NM_014112	0.00725744	0.00725744	0.451656	-2.21407	VVA down vs Control	12.7693	3.15583	1.97713	1
21004	8065469	NM_001042472 // ABHD12 // abhydrolase domain containing 12 // 20p11.21 // 26090	ABHD12	NM_001042472	0.00335833	0.00335833	0.451989	-2.21244	VVA down vs Control	16.9492	3.14998	1.48678	1
27742	8131475	NM_020156 // C1GALT1 // core 1 synthase, glycoprotein-N-acetylgalactosamine 3-be	C1GALT1	NM_020156	0.0125246	0.0125246	0.452018	-2.2123	VVA down vs Control	10.2698	3.14947	2.4534	1
10065	7954527	NM_020183 // ARNTL2 // aryl hydrocarbon receptor nuclear translocator-like 2 //	ARNTL2	NM_020183	0.011428	0.011428	0.452068	-2.21206	VVA down vs Control	10.6653	3.1486	2.36174	1
31355	8165334	NM_004669 // CLIC3 // chloride intracellular channel 3 // 9q34.3 // 9022 /// ENS	CLIC3	NM_004669	0.0207266	0.0207266	0.452193	-2.21145	VVA down vs Control	8.25532	3.14641	3.04909	1
27949	8133176	NM_014504 // RABGEF1 // RAB guanine nucleotide exchange factor (GEF) 1 // 7q11.2	RABGEF1	NM_014504	0.00540897	0.00540897	0.452474	-2.21007	VVA down vs Control	14.268	3.14147	1.76141	1
26937	8123407	NM_001040001 // MLLT4 // myeloid/lymphoid or mixed-lineage leukemia (trithorax h	MLLT4	NM_001040001	0.00666706	0.00666706	0.452948	-2.20776	VVA down vs Control	13.1904	3.13318	1.90028	1
15317	8007976	NM_006310 // NPEPPS // aminopeptidase puromycin sensitive // 17q21 // 9520 /// E	NPEPPS	NM_006310	0.00533691	0.00533691	0.453252	-2.20628	VVA down vs Control	14.3391	3.12788	1.7451	1
22131	8076223	NR_002439 // SNORD43 // small nucleolar RNA, C/D box 43 // 22q13 // 26807 /// EN	SNORD43	NR_002439	0.00800263	0.00800263	0.453454	-2.2053	VVA down vs Control	12.2955	3.12437	2.03286	1
21759	8072659	NR_024194 // TOM1 // target of myb1 (chicken) // 22q13.1 // 10043 /// NM_005488	TOM1	NR_024194	0.0066943	0.0066943	0.453549	-2.20483	VVA down vs Control	13.17	3.1227	1.89686	1
10495	7959144	---		---	0.00159627	0.00159627	0.454237	-2.20149	VVA down vs Control	21.8371	3.11074	1.13962	1
24031	8095834	NM_020859 // SHROOM3 // shroom family member 3 // 4q21.1 // 57619 /// ENST000002	SHROOM3	NM_020859	0.00190259	0.00190259	0.455023	-2.19769	VVA down vs Control	20.5993	3.09713	1.20281	1
17323	8028546	NM_002307 // LGALS7 // lectin, galactoside-binding, soluble, 7 // 19q13.2 // 396	LGALS7	NM_002307	0.0114056	0.0114056	0.455371	-2.19601	VVA down vs Control	10.6739	3.09112	2.31676	1
1917	7894427	---		---	0.0129793	0.0129793	0.4556	-2.19491	VVA down vs Control	10.1183	3.08716	2.44085	1
25731	8112274	NM_024930 // ELOVL7 // ELOVL family member 7, elongation of long chain fatty aci	ELOVL7	NM_024930	0.0177385	0.0177385	0.455644	-2.1947	VVA down vs Control	8.84991	3.08641	2.79	1
5075	7905428	NM_020127 // TUFT1 // tuftelin 1 // 1q21 // 7286 /// NM_001126337 // TUFT1 // tu	TUFT1	NM_020127	0.00509558	0.00509558	0.456609	-2.19006	VVA down vs Control	14.5862	3.06981	1.68368	1
10052	7954407	NM_018686 // CMAS // cytidine monophosphate N-acetylneuraminic acid synthetase /	CMAS	NM_018686	0.0128629	0.0128629	0.456836	-2.18897	VVA down vs Control	10.1564	3.06593	2.41497	1
29733	8149685	NM_139278 // LGI3 // leucine-rich repeat LGI family, member 3 // 8p21.3 // 20319	LGI3	NM_139278	0.0174238	0.0174238	0.456873	-2.18879	VVA down vs Control	8.91984	3.0653	2.7492	1
29862	8150870	NM_002806 // PSMC6 // proteasome (prosome, macropain) 26S subunit, ATPase, 6 //	PSMC6	NM_002806	0.0307691	0.0307691	0.45707	-2.18785	VVA down vs Control	6.85137	3.06191	3.57524	1
10712	7961371	NM_030640 // DUSP16 // dual specificity phosphatase 16 // 12p13 // 80824 /// ENS	DUSP16	NM_030640	0.00301264	0.00301264	0.457132	-2.18755	VVA down vs Control	17.607	3.06085	1.39074	1
5052	7905154	BC027999 // C1orf51 // chromosome 1 open reading frame 51 // 1q21.2 // 148523 //	C1orf51	BC027999	0.0217715	0.0217715	0.457801	-2.18436	VVA down vs Control	8.07246	3.04944	3.02207	1
31900	8169928	NR_027141 // FAM45B // family with sequence similarity 45, member A pseudogene /	FAM45B	NR_027141	0.00368496	0.00368496	0.459173	-2.17783	VVA down vs Control	16.4013	3.02611	1.47603	1
28594	8139367	NM_013389 // NPC1L1 // NPC1 (Niemann-Pick disease, type C1, gene)-like 1 // 7p13	NPC1L1	NM_013389	0.0376004	0.0376004	0.460793	-2.17017	VVA down vs Control	6.19345	2.99879	3.8735	1
30637	8157947	NM_001099270 // ZBTB34 // zinc finger and BTB domain containing 34 // 9q33.3 //	ZBTB34	NM_001099270	0.00758844	0.00758844	0.460917	-2.16959	VVA down vs Control	12.5516	2.99671	1.91	1
30398	8155627	AL833349 // FAM95B1 // family with sequence similarity 95, member B1 // 9p12 //	FAM95B1	AL833349	0.0198485	0.0198485	0.461755	-2.16565	VVA down vs Control	8.41822	2.98267	2.83449	1
30956	8161381	AL833349 // FAM95B1 // family with sequence similarity 95, member B1 // 9p12 //	FAM95B1	AL833349	0.0198485	0.0198485	0.461755	-2.16565	VVA down vs Control	8.41822	2.98267	2.83449	1
16004	8015366	NM_000526 // KRT14 // keratin 14 // 17q12-q21 // 3861 /// ENST00000167586 // KRT	KRT14	NM_000526	0.00135974	0.00135974	0.46351	-2.15745	VVA down vs Control	23.0168	2.95345	1.02654	1
13471	7988970	NM_019600 // KIAA1370 // KIAA1370 // 15q21.2-q21.3 // 56204 /// ENST00000261844	KIAA1370	NM_019600	0.0196912	0.0196912	0.463604	-2.15702	VVA down vs Control	8.44838	2.95191	2.79524	1
6952	7924582	NM_025160 // WDR26 // WD repeat domain 26 // 1q42.11-q42.12 // 80232 /// NM_0011	WDR26	NM_025160	0.00396829	0.00396829	0.463694	-2.15659	VVA down vs Control	15.9732	2.95041	1.47768	1
18752	8042503	NM_002357 // MXD1 // MAX dimerization protein 1 // 2p13-p12 // 4084 /// ENST0000	MXD1	NM_002357	0.0288163	0.0288163	0.464422	-2.15321	VVA down vs Control	7.07431	2.93838	3.32287	1
28343	8137112	NM_012256 // ZNF212 // zinc finger protein 212 // 7q36.1 // 7988 /// ENST0000033	ZNF212	NM_012256	0.0107101	0.0107101	0.464646	-2.15218	VVA down vs Control	10.9512	2.93469	2.14384	1
16532	8020762	NM_001944 // DSG3 // desmoglein 3 (pemphigus vulgaris antigen) // 18q12.1-q12.2	DSG3	NM_001944	0.0102765	0.0102765	0.465471	-2.14836	VVA down vs Control	11.1357	2.92111	2.09855	1
11149	7966072	NM_012406 // PRDM4 // PR domain containing 4 // 12q23-q24.1 // 11108 /// ENST000	PRDM4	NM_012406	0.016329	0.016329	0.466543	-2.14342	VVA down vs Control	9.17612	2.90356	2.53141	1
21733	8072382	NM_030758 // OSBP2 // oxysterol binding protein 2 // 22q12.2 // 23762 /// ENST00	OSBP2	NM_030758	0.0316279	0.0316279	0.467541	-2.13885	VVA down vs Control	6.75893	2.88732	3.41748	1
8593	7940667	NR_004390 // SNORA57 // small nucleolar RNA, H/ACA box 57 // 11q12.2 // 692158	SNORA57	NR_004390	0.0227142	0.0227142	0.467922	-2.13711	VVA down vs Control	7.91675	2.88113	2.91142	1
17197	8027292	NM_133473 // ZNF431 // zinc finger protein 431 // 19p12 // 170959 /// ENST000003	ZNF431	NM_133473	0.00992407	0.00992407	0.467925	-2.13709	VVA down vs Control	11.2931	2.88108	2.04094	1
19657	8051462	---		---	0.00978165	0.00978165	0.468138	-2.13612	VVA down vs Control	11.3587	2.87762	2.02672	1
16124	8016519	---		---	0.00814959	0.00814959	0.468359	-2.13512	VVA down vs Control	12.2085	2.87406	1.88331	1
9935	7953303	NM_018173 // PLEKHG6 // pleckstrin homology domain containing, family G (with Rh	PLEKHG6	NM_018173	0.0263174	0.0263174	0.468445	-2.13472	VVA down vs Control	7.38921	2.87267	3.11012	1
3390	7895909	---		---	0.0399596	0.0399596	0.468482	-2.13456	VVA down vs Control	6.0007	2.87207	3.82898	1
5806	7912706	NM_004431 // EPHA2 // EPH receptor A2 // 1p36 // 1969 /// ENST00000358432 // EPH	EPHA2	NM_004431	0.0451175	0.0451175	0.468495	-2.13449	VVA down vs Control	5.62557	2.87184	4.08399	1
23003	8085272	AK022260 // LOC401052 // hypothetical LOC401052 // 3p25.3 // 401052	LOC401052	AK022260	0.020747	0.020747	0.468581	-2.1341	VVA down vs Control	8.25163	2.87047	2.78294	1
16512	8020508	NM_003831 // RIOK3 // RIO kinase 3 (yeast) // 18q11.2 // 8780 /// ENST0000033948	RIOK3	NM_003831	0.0102288	0.0102288	0.468898	-2.13266	VVA down vs Control	11.1567	2.86535	2.05463	1
9800	7952036	NM_198275 // MPZL3 // myelin protein zero-like 3 // 11q23.3 // 196264 /// ENST00	MPZL3	NM_198275	0.00669505	0.00669505	0.468959	-2.13238	VVA down vs Control	13.1694	2.86436	1.74001	1
634	7893136	---		---	0.0238468	0.0238468	0.469004	-2.13218	VVA down vs Control	7.74013	2.86363	2.95978	1
12085	7975368	NM_001039465 // SFRS5 // splicing factor, arginine/serine-rich 5 // 14q24 // 643	SFRS5	NM_001039465	0.000249915	0.000249915	0.469033	-2.13205	VVA down vs Control	38.8672	2.86316	0.589323	1
5908	7914015	NM_052943 // FAM46B // family with sequence similarity 46, member B // 1p36.11 /	FAM46B	NM_052943	0.0254484	0.0254484	0.469409	-2.13034	VVA down vs Control	7.50771	2.85711	3.04446	1
6360	7918768	NM_198459 // DENND2C // DENN/MADD domain containing 2C // 1p13.2 // 163259 /// E	DENND2C	NM_198459	0.0127317	0.0127317	0.46948	-2.13002	VVA down vs Control	10.1999	2.85597	2.23999	1
578	7893080	---		---	0.00740152	0.00740152	0.469673	-2.12914	VVA down vs Control	12.6731	2.85285	1.80089	1
13461	7988763	NM_207381 // TNFAIP8L3 // tumor necrosis factor, alpha-induced protein 8-like 3	TNFAIP8L3	NM_207381	0.00566533	0.00566533	0.470127	-2.12708	VVA down vs Control	14.0244	2.84557	1.62321	1
25596	8110858	AK295082 // PDCD6 // programmed cell death 6 // 5p15.33 // 10016 /// NR_003713 /	PDCD6	AK295082	0.00740217	0.00740217	0.470234	-2.1266	VVA down vs Control	12.6726	2.84386	1.79527	1
17775	8032899	NM_182919 // TICAM1 // toll-like receptor adaptor molecule 1 // 19p13.3 // 14802	TICAM1	NM_182919	0.0466437	0.0466437	0.470837	-2.12388	VVA down vs Control	5.52492	2.83421	4.10389	1
2140	7894651	---		---	0.0438028	0.0438028	0.470892	-2.12363	VVA down vs Control	5.71581	2.83332	3.9656	1
26652	8120833	NM_031469 // SH3BGRL2 // SH3 domain binding glutamic acid-rich protein like 2 //	SH3BGRL2	NM_031469	0.00708248	0.00708248	0.472261	-2.11747	VVA down vs Control	12.8895	2.81153	1.745	1
28490	8138553	NM_032581 // FAM126A // family with sequence similarity 126, member A // 7p15.3	FAM126A	NM_032581	0.0237996	0.0237996	0.473092	-2.11375	VVA down vs Control	7.74728	2.79836	2.88964	1
16667	8022085	NM_004746 // DLGAP1 // discs, large (Drosophila) homolog-associated protein 1 //	DLGAP1	NM_004746	0.0190426	0.0190426	0.473188	-2.11332	VVA down vs Control	8.57595	2.79684	2.60901	1
26709	8121257	NM_001198 // PRDM1 // PR domain containing 1, with ZNF domain // 6q21-q22.1 // 6	PRDM1	NM_001198	0.0487808	0.0487808	0.473252	-2.11304	VVA down vs Control	5.39084	2.79584	4.14902	1
17268	8027884	---		---	0.0238678	0.0238678	0.473698	-2.11105	VVA down vs Control	7.73696	2.7888	2.88362	1
33245	8180363	---		---	0.0030609	0.0030609	0.473864	-2.11031	VVA down vs Control	17.5097	2.78618	1.27298	1
18423	8039306	NM_001145971 // RDH13 // retinol dehydrogenase 13 (all-trans/9-cis) // 19q13.42	RDH13	NM_001145971	0.0255914	0.0255914	0.474153	-2.10902	VVA down vs Control	7.48787	2.78164	2.97189	1
17439	8029779	NM_198541 // IGFL1 // IGF-like family member 1 // 19q13.32 // 374918 /// ENST000	IGFL1	NM_198541	0.00418758	0.00418758	0.474454	-2.10769	VVA down vs Control	15.6674	2.77691	1.41793	1
33244	8180362	---		---	0.00267985	0.00267985	0.474985	-2.10533	VVA down vs Control	18.3364	2.76858	1.2079	1
7124	7926152	NM_024693 // ECHDC3 // enoyl Coenzyme A hydratase domain containing 3 // 10p14 /	ECHDC3	NM_024693	0.028641	0.028641	0.475162	-2.10455	VVA down vs Control	7.09525	2.76582	3.1185	1
16714	8022488	NM_138340 // ABHD3 // abhydrolase domain containing 3 // 18q11.2 // 171586 /// E	ABHD3	NM_138340	0.0292284	0.0292284	0.47531	-2.10389	VVA down vs Control	7.0257	2.76349	3.14672	1
32509	8175269	NM_145284 // FAM122B // family with sequence similarity 122B // Xq26.3 // 159090	FAM122B	NM_145284	0.0105324	0.0105324	0.475516	-2.10298	VVA down vs Control	11.0257	2.76027	2.0028	1
7680	7932023	NM_014688 // USP6NL // USP6 N-terminal like // 10p13 // 9712 /// NM_001080491 //	USP6NL	NM_014688	0.0142133	0.0142133	0.4757	-2.10216	VVA down vs Control	9.73886	2.7574	2.26507	1
27581	8129888	AK299585 // NHSL1 // NHS-like 1 // 6q23.3 // 57224 /// NM_001144060 // NHSL1 //	NHSL1	AK299585	0.007389	0.007389	0.475999	-2.10085	VVA down vs Control	12.6813	2.75275	1.73657	1
8337	7938544	NM_021961 // TEAD1 // TEA domain family member 1 (SV40 transcriptional enhancer	TEAD1	NM_021961	0.0195609	0.0195609	0.476292	-2.09955	VVA down vs Control	8.47357	2.74818	2.59459	1
2269	7894783	---		---	0.0131192	0.0131192	0.476788	-2.09737	VVA down vs Control	10.0731	2.74048	2.17648	1
18498	8039937	NM_001029885 // GLTPD1 // glycolipid transfer protein domain containing 1 // 1p3	GLTPD1	NM_001029885	0.0333363	0.0333363	0.477565	-2.09395	VVA down vs Control	6.58416	2.72843	3.31515	1
30697	8158783	NM_005085 // NUP214 // nucleoporin 214kDa // 9q34.1 // 8021 /// ENST00000359428	NUP214	NM_005085	0.0042858	0.0042858	0.478369	-2.09044	VVA down vs Control	15.5368	2.71603	1.3985	1
4792	7902687	NM_001554 // CYR61 // cysteine-rich, angiogenic inducer, 61 // 1p31-p22 // 3491	CYR61	NM_001554	0.0309006	0.0309006	0.478642	-2.08924	VVA down vs Control	6.83701	2.71182	3.17311	1
2134	7894645	---		---	0.0118981	0.0118981	0.478776	-2.08866	VVA down vs Control	10.4902	2.70977	2.06652	1
3196	7895713	---		---	0.0246001	0.0246001	0.478998	-2.08769	VVA down vs Control	7.62844	2.70636	2.83817	1
12410	7978538	NR_027263 // C14orf128 // chromosome 14 open reading frame 128 // 14q12 // 84837	C14orf128	NR_027263	0.0245578	0.0245578	0.479362	-2.0861	VVA down vs Control	7.6346	2.70077	2.83003	1
22356	8078738	NM_005109 // OXSR1 // oxidative-stress responsive 1 // 3p22-p21.3 // 9943 /// EN	OXSR1	NM_005109	0.00188721	0.00188721	0.480086	-2.08296	VVA down vs Control	20.6553	2.6897	1.04175	1
14746	8002303	NM_000903 // NQO1 // NAD(P)H dehydrogenase, quinone 1 // 16q22.1 // 1728 /// NM	NQO1	NM_000903	0.0153083	0.0153083	0.480442	-2.08142	VVA down vs Control	9.43537	2.68427	2.27592	1
18526	8040211	NM_003597 // KLF11 // Kruppel-like factor 11 // 2p25 // 8462 /// ENST00000305883	KLF11	NM_003597	0.0274495	0.0274495	0.481134	-2.07842	VVA down vs Control	7.24206	2.67374	2.95357	1
17219	8027402	NM_001238 // CCNE1 // cyclin E1 // 19q12 // 898 /// NM_057182 // CCNE1 // cyclin	CCNE1	NM_001238	0.0322058	0.0322058	0.481316	-2.07764	VVA down vs Control	6.6985	2.67098	3.18994	1
14117	7995803	AF348994 // MT1JP // metallothionein 1J (pseudogene) // 16q13 // 4498 /// BC1038	MT1JP	AF348994	0.0495282	0.0495282	0.481749	-2.07577	VVA down vs Control	5.3457	2.6644	3.98736	1
21540	8070701	NM_000100 // CSTB // cystatin B (stefin B) // 21q22.3 // 1476 /// ENST0000029156	CSTB	NM_000100	0.00539028	0.00539028	0.482229	-2.0737	VVA down vs Control	14.2863	2.65715	1.48794	1
16477	8020220	NM_032525 // TUBB6 // tubulin, beta 6 // 18p11.21 // 84617 /// ENST00000317702 /	TUBB6	NM_032525	0.0217432	0.0217432	0.482273	-2.07351	VVA down vs Control	8.07726	2.65648	2.63107	1
16670	8022104	---		---	0.011946	0.011946	0.483129	-2.06984	VVA down vs Control	10.4728	2.64358	2.01939	1
21725	8072328	NM_012429 // SEC14L2 // SEC14-like 2 (S. cerevisiae) // 22q12.2 // 23541 /// NM	SEC14L2	NM_012429	0.0368667	0.0368667	0.48314	-2.06979	VVA down vs Control	6.25655	2.64341	3.38002	1
27260	8126379	ENST00000341865 // C6orf132 // chromosome 6 open reading frame 132 // 6p21.1 //	C6orf132	ENST00000341865	0.0374205	0.0374205	0.483598	-2.06783	VVA down vs Control	6.20877	2.63654	3.39718	1
12557	7979943	NM_033141 // MAP3K9 // mitogen-activated protein kinase kinase kinase 9 // 14q24	MAP3K9	NM_033141	0.0153997	0.0153997	0.48379	-2.06701	VVA down vs Control	9.41128	2.63366	2.23872	1
18982	8044574	NM_173842 // IL1RN // interleukin 1 receptor antagonist // 2q14.2 // 3557 /// NM	IL1RN	NM_173842	0.0117404	0.0117404	0.484213	-2.06521	VVA down vs Control	10.5479	2.62732	1.99267	1
13060	7984319	NM_002755 // MAP2K1 // mitogen-activated protein kinase kinase 1 // 15q22.1-q22.	MAP2K1	NM_002755	0.0214667	0.0214667	0.484514	-2.06392	VVA down vs Control	8.12464	2.62282	2.58258	1
16474	8020197	ENST00000314074 // ANKRD62 // ankyrin repeat domain 62 // 18p11.21 // 342850 ///	ANKRD62	ENST00000314074	0.0205511	0.0205511	0.48505	-2.06164	VVA down vs Control	8.28718	2.61481	2.5242	1
7652	7931754	NM_004508 // IDI1 // isopentenyl-diphosphate delta isomerase 1 // 10p15.3 // 342	IDI1	NM_004508	0.00504613	0.00504613	0.485152	-2.06121	VVA down vs Control	14.6387	2.6133	1.42816	1
13590	7990400	NM_001099436 // ULK3 // unc-51-like kinase 3 (C. elegans) // 15q24.1 // 25989 //	ULK3	NM_001099436	0.0238015	0.0238015	0.485187	-2.06106	VVA down vs Control	7.747	2.61278	2.69811	1
12016	7974473	NM_017943 // FBXO34 // F-box protein 34 // 14q22.3 // 55030 /// NM_152231 // FBX	FBXO34	NM_017943	0.0118567	0.0118567	0.485386	-2.06021	VVA down vs Control	10.5052	2.60981	1.98743	1
16572	8021181	NR_003003 // SCARNA17 // small Cajal body-specific RNA 17 // 18q21.1 // 677769	SCARNA17	NR_003003	0.00660044	0.00660044	0.485496	-2.05975	VVA down vs Control	13.2408	2.60818	1.57584	1
5308	7907611	NM_170692 // RASAL2 // RAS protein activator like 2 // 1q24 // 9462 /// NM_00484	RASAL2	NM_170692	0.0147939	0.0147939	0.485516	-2.05966	VVA down vs Control	9.57443	2.60788	2.17904	1
18660	8041636	---		---	0.0297601	0.0297601	0.486182	-2.05684	VVA down vs Control	6.96426	2.598	2.98438	1
31159	8163063	NM_003798 // CTNNAL1 // catenin (cadherin-associated protein), alpha-like 1 // 9	CTNNAL1	NM_003798	0.00315514	0.00315514	0.486312	-2.05629	VVA down vs Control	17.3249	2.59607	1.19877	1
5487	7909789	NM_001135599 // TGFB2 // transforming growth factor, beta 2 // 1q41 // 7042 ///	TGFB2	NM_001135599	0.0135363	0.0135363	0.486764	-2.05438	VVA down vs Control	9.94166	2.58939	2.08367	1
24509	8100362	NM_001126328 // LNX1 // ligand of numb-protein X 1 // 4q12 // 84708 /// NM_03262	LNX1	NM_001126328	0.0475062	0.0475062	0.486927	-2.0537	VVA down vs Control	5.46988	2.58697	3.78359	1
19593	8050702	NM_004881 // TP53I3 // tumor protein p53 inducible protein 3 // 2p23.3 // 9540 /	TP53I3	NM_004881	0.0117836	0.0117836	0.487861	-2.04977	VVA down vs Control	10.532	2.57322	1.95459	1
22644	8081779	NM_173570 // ZDHHC23 // zinc finger, DHHC-type containing 23 // 3q13.31 // 25488	ZDHHC23	NM_173570	0.0126204	0.0126204	0.488364	-2.04765	VVA down vs Control	10.2373	2.56582	2.00508	1
30359	8155418	AL833349 // FAM95B1 // family with sequence similarity 95, member B1 // 9p12 //	FAM95B1	AL833349	0.0259895	0.0259895	0.48866	-2.04641	VVA down vs Control	7.43334	2.56149	2.75676	1
24152	8097096	---		---	0.0129667	0.0129667	0.488811	-2.04578	VVA down vs Control	10.1224	2.55929	2.02267	1
20602	8061447	NM_002862 // PYGB // phosphorylase, glycogen; brain // 20p11.2-p11.1 // 5834 ///	PYGB	NM_002862	0.0350352	0.0350352	0.489305	-2.04371	VVA down vs Control	6.42128	2.55206	3.1795	1
18611	8040980	NM_032266 // C2orf16 // chromosome 2 open reading frame 16 // 2p23.3 // 84226 //	C2orf16	NM_032266	0.0188589	0.0188589	0.489358	-2.04349	VVA down vs Control	8.6131	2.55129	2.36968	1
17116	8026456	NM_000896 // CYP4F3 // cytochrome P450, family 4, subfamily F, polypeptide 3 //	CYP4F3	NM_000896	0.00520824	0.00520824	0.489497	-2.04291	VVA down vs Control	14.4691	2.54927	1.4095	1
25489	8109752	AK302302 // ODZ2 // odz, odd Oz/ten-m homolog 2 (Drosophila) // 5q34 // 57451 //	ODZ2	AK302302	0.0378554	0.0378554	0.490171	-2.0401	VVA down vs Control	6.17188	2.53945	3.29164	1
11597	7970831	NM_007106 // UBL3 // ubiquitin-like 3 // 13q12-q13 // 5412 /// ENST00000380680 /	UBL3	NM_007106	0.0132177	0.0132177	0.490663	-2.03806	VVA down vs Control	10.0415	2.53231	2.01747	1
8117	7936614	NM_003750 // EIF3A // eukaryotic translation initiation factor 3, subunit A // 1	EIF3A	NM_003750	0.0023503	0.0023503	0.490677	-2.038	VVA down vs Control	19.1802	2.53212	1.05614	1

Column #	Transcript Cluster ID	gene_assignment	Gene Symbol	RefSeq	p-value(Group)	p-value(VVA vs. Control)	Ratio(VVA vs. Control)	Fold-Change(VVA vs. Control)	Fold-Change(VVA vs. Control) (Description)	F(Group)	SS(Group)	SS(Error)	F(Error)
11144	7966035	NM 006825 // CKAP4 // cytoskeleton-associated protein 4 // 12q23.3 // 10970 ///	CKAP4	NM 006825	0.00452317	0.00452317	0.490825	-2.03739	VVA down vs Control	15.2362	2.52997	1.3284	1
21658	8071649	BC043345 // BMS1 // BMS1 homolog, ribosome assembly protein (yeast) // 10q11.21	BMS1	BC043345	0.00176906	0.00176906	0.491267	-2.03555	VVA down vs Control	21.1057	2.52357	0.956544	1
26144	8115918	NM 007097 // CLTB // clathrin, light chain (Lcb) // 4q2-q3|5q35 // 1212 /// NM 0	CLTB	NM 007097	0.00917107	0.00917107	0.492573	-2.03015	VVA down vs Control	11.6543	2.50475	1.71937	1
29849	8150757	NM 014781 // RB1CC1 // RB1-inducible coiled-coil 1 // 8q11 // 9821 /// NM 001083	RB1CC1	NM 014781	0.00972777	0.00972777	0.49308	-2.02807	VVA down vs Control	11.3839	2.49748	1.7551	1
24546	8100655	NM 000406 // GNRHR // gonadotropin-releasing hormone receptor // 4q21.2 // 2798	GNRHR	NM 000406	0.0124669	0.0124669	0.493606	-2.02591	VVA down vs Control	10.2895	2.48996	1.93593	1
22396	8079149	---		---	0.0184549	0.0184549	0.493833	-2.02497	VVA down vs Control	8.69639	2.48671	2.28758	1
22312	8078272	NM 005126 // NR1D2 // nuclear receptor subfamily 1, group D, member 2 // 3p24.2	NR1D2	NM 005126	0.0247946	0.0247946	0.494113	-2.02383	VVA down vs Control	7.60029	2.48271	2.61328	1
17273	8027938	NM 007000 // UPK1A // uroplakin 1A // 19q13.13 // 11045 /// ENST00000222275 // U	UPK1A	NM 007000	0.0179614	0.0179614	0.494478	-2.02234	VVA down vs Control	8.80131	2.47752	2.25196	1
7238	7927280	NM 005972 // PPYR1 // pancreatic polypeptide receptor 1 // 10q11.2 // 5540 /// E	PPYR1	NM 005972	0.0205353	0.0205353	0.494501	-2.02224	VVA down vs Control	8.29006	2.47719	2.39052	1
19228	8046726	NM 001130445 // SSFA2 // sperm specific antigen 2 // 2q31.3 // 6744 /// NM 00675	SSFA2	NM 001130445	0.0010882	0.0010882	0.494564	-2.02198	VVA down vs Control	24.7366	2.47629	0.800852	1
18347	8038707	NM 144505 // KLK8 // kallikrein-related peptidase 8 // 19q13.3-q13.4 // 11202 //	KLK8	NM 144505	0.0409368	0.0409368	0.495004	-2.02019	VVA down vs Control	5.92506	2.47004	3.33505	1
10485	7959070	NM 002567 // PEBP1 // phosphatidylethanolamine binding protein 1 // 12q24.23 //	PEBP1	NM 002567	0.020314	0.020314	0.496796	-2.0129	VVA down vs Control	8.33075	2.44473	2.34767	1
9068	7945503	NM 021008 // DEAF1 // deformed epidermal autoregulatory factor 1 (Drosophila) //	DEAF1	NM 021008	0.0291426	0.0291426	0.496872	-2.01259	VVA down vs Control	7.03576	2.44366	2.77856	1
1271	7893774	---		---	0.00603651	0.00603651	0.497108	-2.01163	VVA down vs Control	13.6952	2.44034	1.42551	1
26837	8122343	NM 016217 // HECA // headcase homolog (Drosophila) // 6q23-q24 // 51696 /// ENST	HECA	NM 016217	0.013427	0.013427	0.497966	-2.00817	VVA down vs Control	9.97559	2.42831	1.9474	1
12523	7979698	NM 015994 // ATP6V1D // ATPase, H+ transporting, lysosomal 34kDa, V1 subunit D /	ATP6V1D	NM 015994	0.0181552	0.0181552	0.498611	-2.00557	VVA down vs Control	8.75967	2.4193	2.20949	1
16473	8020183	NM 014214 // IMPA2 // inositol(myo)-1(or 4)-monophosphatase 2 // 18p11.2 // 3613	IMPA2	NM 014214	0.00902053	0.00902053	0.498838	-2.00466	VVA down vs Control	11.7309	2.41614	1.6477	1
21664	8071691	NM 004327 // BCR // breakpoint cluster region // 22q11|22q11.23 // 613 /// NM 02	BCR	NM 004327	0.0124798	0.0124798	0.499298	-2.00281	VVA down vs Control	10.2851	2.40974	1.87436	1
30301	8154872	NM 016525 // UBAP1 // ubiquitin associated protein 1 // 9p13.3 // 51271 /// ENST	UBAP1	NM 016525	0.0130543	0.0130543	0.499924	-2.0003	VVA down vs Control	10.094	2.40105	1.90296	1
20255	8057744	NM 007315 // STAT1 // signal transducer and activator of transcription 1, 91kDa	STAT1	NM 007315	0.01823	0.01823	2.0019	2.0019	VVA up vs Control	8.74377	2.40658	2.20187	1
30841	8160346	NM 001010915 // PTPLAD2 // protein tyrosine phosphatase-like A domain containing	PTPLAD2	NM 001010915	0.0431518	0.0431518	2.00208	2.00208	VVA up vs Control	5.76178	2.40721	3.34231	1
9261	7947138	NM 022725 // FANCF // Fanconi anemia, complementation group F // 11p15 // 2188 /	FANCF	NM 022725	0.0425027	0.0425027	2.003	2.003	VVA up vs Control	5.8085	2.41039	3.31981	1
559	7893061	---		---	0.0383688	0.0383688	2.00328	2.00328	VVA up vs Control	6.12902	2.41135	3.14745	1
10266	7956613	NM 005981 // TSPAN31 // tetraspanin 31 // 12q13.3 // 6302 /// ENST00000257910 //	TSPAN31	NM 005981	0.00654914	0.00654914	2.00405	2.00405	VVA up vs Control	13.2801	2.41402	1.45421	1
30326	8155192	NM 022343 // GLIPR2 // GLI pathogenesis-related 2 // 9p13-p12 // 152007 /// ENST	GLIPR2	NM 022343	0.0287269	0.0287269	2.00462	2.00462	VVA up vs Control	7.08497	2.41601	2.72804	1
11150	7966089	NM 001142343 // CMKLR1 // chemokine-like receptor 1 // 12q24.1 // 1240 /// NM 00	CMKLR1	NM 001142343	0.0210287	0.0210287	2.00507	2.00507	VVA up vs Control	8.20127	2.41755	2.35822	1
33064	8180078	NM 002118 // HLA-DMB // major histocompatibility complex, class II, DM beta // 6	HLA-DMB	NM 002118	0.0268575	0.0268575	2.00531	2.00531	VVA up vs Control	7.31803	2.41839	2.64375	1
32867	8178188	NR 003129 // RNF5P1 // ring finger protein 5 pseudogene 1 // 8p11.22 // 286140 /	RNF5P1	NR 003129	0.000161231	0.000161231	2.00554	2.00554	VVA up vs Control	44.1888	2.4192	0.437975	1
384	7892884	---		---	0.0250106	0.0250106	2.00556	2.00556	VVA up vs Control	7.56937	2.41925	2.55689	1
26180	8116372	NM 018434 // RNF130 // ring finger protein 130 // 5q35.3 // 55819 /// ENST000002	RNF130	NM 018434	0.0260242	0.0260242	2.0079	2.0079	VVA up vs Control	7.42864	2.42737	2.61407	1
14140	7996081	NM 201524 // GPR56 // G protein-coupled receptor 56 // 16q12.2-q21 // 9289 /// N	GPR56	NM 201524	0.0269424	0.0269424	2.00959	2.00959	VVA up vs Control	7.30701	2.43326	2.66402	1
23304	8088848	NM 015009 // PDZRN3 // PDZ domain containing ring finger 3 // 3p13 // 23024 ///	PDZRN3	NM 015009	0.0341817	0.0341817	2.01199	2.01199	VVA up vs Control	6.50183	2.44156	3.00415	1
27179	8125530	NM 002118 // HLA-DMB // major histocompatibility complex, class II, DM beta // 6	HLA-DMB	NM 002118	0.0255292	0.0255292	2.01217	2.01217	VVA up vs Control	7.49648	2.44219	2.60623	1
6664	7921637	NM 003874 // CD84 // CD84 molecule // 1q24 // 8832 /// ENST00000368054 // CD84 /	CD84	NM 003874	0.021428	0.021428	2.01506	2.01506	VVA up vs Control	8.13132	2.45224	2.41263	1
11073	7965335	NM 001946 // DUSP6 // dual specificity phosphatase 6 // 12q22-q23 // 1848 /// NM	DUSP6	NM 001946	0.0157361	0.0157361	2.0155	2.0155	VVA up vs Control	9.32414	2.45375	2.10529	1
1756	7894264	---		---	0.00219407	0.00219407	2.01782	2.01782	VVA up vs Control	19.6338	2.46181	1.00309	1
10834	7962794	NM 152320 // ZNF641 // zinc finger protein 641 // 12q13.11 // 121274 /// ENST000	ZNF641	NM 152320	0.0201458	0.0201458	2.01913	2.01913	VVA up vs Control	8.36207	2.46638	2.35959	1
14922	8004184	NM 017523 // XAF1 // XIAP associated factor 1 // 17p13.1 // 54739 /// NM 199139	XAF1	NM 017523	0.0206948	0.0206948	2.01975	2.01975	VVA up vs Control	8.26106	2.46853	2.39052	1
20617	8061564	NM 181353 // ID1 // inhibitor of DNA binding 1, dominant negative helix-loop-hel	ID1	NM 181353	0.0118244	0.0118244	2.02238	2.02238	VVA up vs Control	10.517	2.47769	1.88471	1
25943	8114010	NM 002198 // IRF1 // interferon regulatory factor 1 // 5q31.1 // 3659 /// ENST00	IRF1	NM 002198	0.0141466	0.0141466	2.0224	2.0224	VVA up vs Control	9.75829	2.47775	2.0313	1
10403	7958019	NM 018370 // DRAM1 // DNA-damage regulated autophagy modulator 1 // 12q23.2 // 5	DRAM1	NM 018370	0.00870613	0.00870613	2.02474	2.02474	VVA up vs Control	11.8964	2.48589	1.67169	1
29769	8150014	NM 018250 // INTS9 // integrator complex subunit 9 // 8p21.1 // 55756 /// NM 001	INTS9	NM 018250	0.0215555	0.0215555	2.02509	2.02509	VVA up vs Control	8.10933	2.48709	2.45356	1
18545	8040365	NM 021643 // TRIB2 // tribbles homolog 2 (Drosophila) // 2p24.3 // 28951 /// NR	TRIB2	NM 021643	0.043297	0.043297	2.02511	2.02511	VVA up vs Control	5.75144	2.48719	3.45956	1
28921	8142452	NM 012252 // TFEC // transcription factor EC // 7q31.2 // 22797 /// NM 001018058	TFEC	NM 012252	0.0199909	0.0199909	2.02525	2.02525	VVA up vs Control	8.3912	2.48765	2.37168	1
5877	7913694	NM 000147 // FUCA1 // fucosidase, alpha-L- 1, tissue // 1p34 // 2517 /// ENST000	FUCA1	NM 000147	0.00730134	0.00730134	2.02565	2.02565	VVA up vs Control	12.7397	2.48907	1.56302	1
4165	7896691	---		---	0.0265919	0.0265919	2.02595	2.02595	VVA up vs Control	7.3528	2.49012	2.7093	1
22097	8075886	NM 000878 // IL2RB // interleukin 2 receptor, beta // 22q13|22q13.1 // 3560 ///	IL2RB	NM 000878	0.0265919	0.0265919	2.02595	2.02595	VVA up vs Control	7.3528	2.49012	2.7093	1
14532	8000284	NM 015044 // GGA2 // golgi associated, gamma adaptin ear containing, ARF binding	GGA2	NM 015044	0.00937451	0.00937451	2.02643	2.02643	VVA up vs Control	11.5531	2.49179	1.72545	1
9085	7945666	NM 001909 // CTSD // cathepsin D // 11p15.5 // 1509 /// ENST00000236671 // CTSD	CTSD	NM 001909	0.00375307	0.00375307	2.02697	2.02697	VVA up vs Control	16.2947	2.49367	1.22428	1
15135	8006239	NM 001042492 // NF1 // neurofibromin 1 // 17q11.2 // 4763 /// NM 000267 // NF1 /	NF1	NM 001042492	0.042514	0.042514	2.02731	2.02731	VVA up vs Control	5.80768	2.49482	3.43659	1
22787	8083272	NM 004130 // GYG1 // glycogenin 1 // 3q24-q25.1 // 2992 /// ENST00000345003 // G	GYG1	NM 004130	0.00354903	0.00354903	2.02781	2.02781	VVA up vs Control	16.6216	2.49657	1.2016	1
28984	8143054	NM 001628 // AKR1B1 // aldo-keto reductase family 1, member B1 (aldose reductase	AKR1B1	NM 001628	0.0077556	0.0077556	2.02919	2.02919	VVA up vs Control	12.4462	2.50137	1.6078	1
21803	8073081	NM 145298 // APOBEC3F // apolipoprotein B mRNA editing enzyme, catalytic polypep	APOBEC3F	NM 145298	1.82E-05	1.82E-05	2.03186	2.03186	VVA up vs Control	81.3682	2.5107	0.246848	1
10669	7961059	NM 002258 // KLRB1 // killer cell lectin-like receptor subfamily B, member 1 //	KLRB1	NM 002258	0.0414694	0.0414694	2.03234	2.03234	VVA up vs Control	5.88478	2.51237	3.41541	1
26055	8115076	NM 005211 // CSF1R // colony stimulating factor 1 receptor // 5q33-q35 // 1436 /	CSF1R	NM 005211	0.00977723	0.00977723	2.033	2.033	VVA up vs Control	11.3608	2.51465	1.77076	1
22678	8082100	NM 017554 // PARP14 // poly (ADP-ribose) polymerase family, member 14 // 3q21.1	PARP14	NM 017554	0.0268146	0.0268146	2.03526	2.03526	VVA up vs Control	7.32363	2.52255	2.75553	1
354	7892854	---		---	0.00166736	0.00166736	2.03669	2.03669	VVA up vs Control	21.5247	2.52753	0.939398	1
22672	8082035	NM 175862 // CD86 // CD86 molecule // 3q21 // 942 /// NM 006889 // CD86 // CD86	CD86	NM 175862	0.0127732	0.0127732	2.03688	2.03688	VVA up vs Control	10.1861	2.52821	1.98561	1
6496	7919856	NM 181746 // LASS2 // LAG1 homolog, ceramide synthase 2 // 1q21.3 // 29956 /// N	LASS2	NM 181746	0.00494863	0.00494863	2.03696	2.03696	VVA up vs Control	14.744	2.52848	1.37194	1
4169	7896695	---		---	0.00074917	0.00074917	2.0371	2.0371	VVA up vs Control	27.8438	2.52898	0.726618	1
15131	8006214	NM 018404 // ADAP2 // ArfGAP with dual PH domains 2 // 17q11.2 // 55803 /// ENST	ADAP2	NM 018404	0.00339812	0.00339812	2.03883	2.03883	VVA up vs Control	16.879	2.535	1.20149	1
24455	8099834	NM 003263 // TLR1 // toll-like receptor 1 // 4p14 // 7096 /// ENST00000308979 //	TLR1	NM 003263	0.0381227	0.0381227	2.03958	2.03958	VVA up vs Control	6.14948	2.53763	3.30126	1
27189	8125713	NM 172208 // TAPBP // TAP binding protein (tapasin) // 6p21.3 // 6892 /// NM 003	TAPBP	NM 172208	0.00285313	0.00285313	2.04059	2.04059	VVA up vs Control	17.9433	2.54114	1.13296	1
5583	7910706	NM 006499 // LGALS8 // lectin, galactoside-binding, soluble, 8 // 1q42-q43 // 39	LGALS8	NM 006499	0.00651539	0.00651539	2.04115	2.04115	VVA up vs Control	13.3062	2.54312	1.52898	1
8553	7940259	NM 021201 // MS4A7 // membrane-spanning 4-domains, subfamily A, member 7 // 11q1	MS4A7	NM 021201	0.0374706	0.0374706	2.04144	2.04144	VVA up vs Control	6.2045	2.54412	3.28036	1
2574	7895090	---		---	0.0193325	0.0193325	2.04223	2.04223	VVA up vs Control	8.51826	2.54688	2.39193	1
3518	7896038	---		---	0.00423213	0.00423213	2.04293	2.04293	VVA up vs Control	15.6077	2.54934	1.30671	1
30278	8154727	AF091236 // LOC138412 // solute carrier family 25 (mitochondrial carrier; adenin	LOC138412	AF091236	0.0190365	0.0190365	2.04536	2.04536	VVA up vs Control	8.57718	2.55782	2.38569	1
22788	8083282	NM 032383 // HPS3 // Hermansky-Pudlak syndrome 3 // 3q24 // 84343 /// ENST000002	HPS3	NM 032383	0.0295754	0.0295754	2.04546	2.04546	VVA up vs Control	6.98544	2.55817	2.92972	1
15685	8012257	NM 000546 // TP53 // tumor protein p53 // 17p13.1 // 7157 /// NM 001126112 // TP	TP53	NM 000546	0.0167308	0.0167308	2.04841	2.04841	VVA up vs Control	9.0796	2.56849	2.26309	1
18263	8037835	NM 005628 // SLC1A5 // solute carrier family 1 (neutral amino acid transporter),	SLC1A5	NM 005628	0.0315186	0.0315186	2.04894	2.04894	VVA up vs Control	6.77052	2.57032	3.03707	1
16763	8022927	NM 012319 // SLC39A6 // solute carrier family 39 (zinc transporter), member 6 //	SLC39A6	NM 012319	0.0214848	0.0214848	2.0504	2.0504	VVA up vs Control	8.12151	2.57543	2.5369	1
22286	8078008	NM 014463 // LSM3 // LSM3 homolog, U6 small nuclear RNA associated (S. cerevisia	LSM3	NM 014463	0.0106404	0.0106404	2.05104	2.05104	VVA up vs Control	10.9802	2.5777	1.87807	1
17812	8033362	NM 000208 // INSR // insulin receptor // 19p13.3-p13.2 // 3643 /// NM 001079817	INSR	NM 000208	0.0253893	0.0253893	2.05264	2.05264	VVA up vs Control	7.51596	2.58327	2.74964	1
454	7892954	---		---	0.0466506	0.0466506	2.05281	2.05281	VVA up vs Control	5.52448	2.58386	3.74169	1
18056	8035980	NM 033103 // RHPN2 // rhophilin, Rho GTPase binding protein 2 // 19q13.11 // 854	RHPN2	NM 033103	0.0162555	0.0162555	2.05339	2.05339	VVA up vs Control	9.19411	2.5859	2.25005	1
8933	7944179	NM 000733 // CD3E // CD3e molecule, epsilon (CD3-TCR complex) // 11q23 // 916 //	CD3E	NM 000733	0.0412042	0.0412042	2.05407	2.05407	VVA up vs Control	5.90475	2.58827	3.50669	1
10733	7961546	NM 004447 // EPS8 // epidermal growth factor receptor pathway substrate 8 // 12q	EPS8	NM 004447	0.0161226	0.0161226	2.05698	2.05698	VVA up vs Control	9.22687	2.59846	2.25295	1
4496	7899562	NM 133178 // PTPRU // protein tyrosine phosphatase, receptor type, U // 1p35.3-p	PTPRU	NM 133178	0.0103911	0.0103911	2.05865	2.05865	VVA up vs Control	11.086	2.60432	1.87936	1
30320	8155096	NM 006368 // CREB3 // cAMP responsive element binding protein 3 // 9pter-p22.1 /	CREB3	NM 006368	0.0206138	0.0206138	2.0635	2.0635	VVA up vs Control	8.27576	2.62132	2.53398	1
25227	8107129	NM 180991 // SLCO4C1 // solute carrier organic anion transporter family, member	SLCO4C1	NM 180991	0.0050271	0.0050271	2.06416	2.06416	VVA up vs Control	14.659	2.62365	1.43182	1
24797	8103025	NM 178835 // ZNF827 // zinc finger protein 827 // 4q31.21-q31.22 // 152485 /// E	ZNF827	NM 178835	0.0125103	0.0125103	2.06505	2.06505	VVA up vs Control	10.2746	2.62676	2.04524	1
7808	7933204	NM 007021 // C10orf10 // chromosome 10 open reading frame 10 // 10q11.21 // 1106	C10orf10	NM 007021	0.00825032	0.00825032	2.06539	2.06539	VVA up vs Control	12.1501	2.62795	1.73032	1
13880	7993167	NM 024997 // ATF7IP2 // activating transcription factor 7 interacting protein 2	ATF7IP2	NM 024997	0.0323723	0.0323723	2.06611	2.06611	VVA up vs Control	6.68134	2.63049	3.14965	1
32439	8174692	NM 145799 // SEPT6 // septin 6 // Xq24 // 23157 /// NM 145800 // SEPT6 // septin	05-Sep	NM 145799	0.0306432	0.0306432	2.06711	2.06711	VVA up vs Control	6.8652	2.63398	3.06937	1
8572	7940473	NM 016464 // TMEM138 // transmembrane protein 138 // 11q12.2 // 51524 /// ENST00	TMEM138	NM 016464	0.00719704	0.00719704	2.06811	2.06811	VVA up vs Control	12.8104	2.63751	1.6471	1
14315	7997940	NR 002450 // SNORD68 // small nucleolar RNA, C/D box 68 // 16q24.3 // 606500 ///	SNORD68	NR 002450	0.0100252	0.0100252	2.06874	2.06874	VVA up vs Control	11.2472	2.63973	1.8776	1
4779	7902553	NM 006417 // IFI44 // interferon-induced protein 44 // 1p31.1 // 10561 /// ENST0	IFI44	NM 006417	0.00138927	0.00138927	2.07349	2.07349	VVA up vs Control	22.8559	2.65641	0.929793	1
29199	8144857	NM 001160170 // NAT1 // N-acetyltransferase 1 (arylamine N-acetyltransferase) //	NAT1	NM 001160170	0.0154152	0.0154152	2.07413	2.07413	VVA up vs Control	9.4072	2.65866	2.26095	1
24100	8096617	NM 017935 // BANK1 // B-cell scaffold protein with ankyrin repeats 1 // 4q24 //	BANK1	NM 017935	0.0129978	0.0129978	2.07694	2.07694	VVA up vs Control	10.1123	2.66853	2.11112	1
28216	8136039	NM 004231 // ATP6V1F // ATPase, H+ transporting, lysosomal 14kDa, V1 subunit F /	ATP6V1F	NM 004231	0.000580675	0.000580675	2.07888	2.07888	VVA up vs Control	30.1382	2.67536	0.710157	1
27177	8125512	NM 000593 // TAP1 // transporter 1, ATP-binding cassette, sub-family B (MDR/TAP)	TAP1	NM 000593	0.0262286	0.0262286	2.07951	2.07951	VVA up vs Control	7.4011	2.67758	2.89425	1
32941	8178867	NM 000593 // TAP1 // transporter 1, ATP-binding cassette, sub-family B (MDR/TAP)	TAP1	NM 000593	0.0262286	0.0262286	2.07951	2.07951	VVA up vs Control	7.4011	2.67758	2.89425	1
33062	8180061	NM 000593 // TAP1 // transporter 1, ATP-binding cassette, sub-family B (MDR/TAP)	TAP1	NM 000593	0.0262286	0.0262286	2.07951	2.07951	VVA up vs Control	7.4011	2.67758	2.89425	1
19869	8053584	NR 027353 // CD8A // CD8a molecule // 2p12 // 925 /// NM 001768 // CD8A // CD8a	CD8A	NR 027353	0.0285906	0.0285906	2.07955	2.07955	VVA up vs Control	7.10131	2.67769	3.01656	1
7549	7930537	NM 001146274 // TCF7L2 // transcription factor 7-like 2 (T-cell specific, HMG-bo	TCF7L2	NM 001146274	0.0119812	0.0119812	2.08019	2.08019	VVA up vs Control	10.4601	2.67996	2.04966	1
21261	8067963	---		---	0.0209523	0.0209523	2.08057	2.08057	VVA up vs Control	8.21484	2.6813	2.61118	1
25918	8113717	---		---	0.0248753	0.0248753	2.08095	2.08095	VVA up vs Control	7.5887	2.68261	2.82801	1
28936	8142585	NM 017954 // CADPS2 // Ca++-dependent secretion activator 2 // 7q31.3 // 93664 /	CADPS2	NM 017954	0.0399592	0.0399592	2.08218	2.08218	VVA up vs Control	6.00073	2.68695	3.58216	1
28082	8134552	NM 005720 // ARPC1B // actin related protein 2/3 complex, subunit 1B, 41kDa // 7	ARPC1B	NM 005720	0.0314984	0.0314984	2.08305	2.08305	VVA up vs Control	6.77267	2.69003	3.17751	1
10197	7955908	NM 005337 // NCKAP1L // NCK-associated protein 1-like // 12q13.1 // 3071 /// ENS	NCKAP1L	NM 005337	0.03734	0.03734	2.08573	2.08573	VVA up vs Control	6.21566	2.69946	3.47439	1
10682	7961173	---		---	0.0160785	0.0160785	2.08937	2.08937	VVA up vs Control	9.23782	2.71228	2.34885	1
9813	7952185	NM 001382 // DPAGT1 // dolichyl-phosphate (UDP-N-acetylglucosamine) N-acetylgluc	DPAGT1	NM 001382	0.00673511	0.00673511	2.09037	2.09037	VVA up vs Control	13.1395	2.71581	1.65352	1
28041	8134036	NM 152999 // STEAP2 // six transmembrane epithelial antigen of the prostate 2 //	STEAP2	NM 152999	0.0322272	0.0322272	2.09306	2.09306	VVA up vs Control	6.69628	2.72527	3.25586	1
22056	8075493	NM 014323 // PATZ1 // POZ (BTB) and AT hook containing zinc finger 1 // 22q12.2	PATZ1	NM 014323	7.99E-05	7.99E-05	2.09392	2.09392	VVA up vs Control	54.0357	2.72831	0.403927	1
992	7893494	---		---	0.0108711	0.0108711	2.09429	2.09429	VVA up vs Control	10.885	2.72962	2.00615	1
8551	7940237	NM 024021 // MS4A4A // membrane-spanning 4-domains, subfamily A, member 4 // 11q	MS4A4A	NM 024021	0.00167857	0.00167857	2.09464	2.09464	VVA up vs Control	21.477	2.73084	1.01722	1
16398	8019622	NM 145041 // TMEM106A // transmembrane protein 106A // 17q21.31 // 113277 /// EN	TMEM106A	NM 145041	0.00307062	0.00307062	2.09689	2.09689	VVA up vs Control	17.4903	2.7388	1.25272	1
30082	8152628	NM 024295 // DERL1 // Der1-like domain family, member 1 // 8q24.13 // 79139 ///	DERL1	NM 024295	0.00250565	0.00250565	2.09921	2.09921	VVA up vs Control	18.7651	2.74696	1.17109	1
12759	7981919	NM 022807 // SNRPN // small nuclear ribonucleoprotein polypeptide N // 15q11.2 /	SNRPN	NM 022807	0.0240796	0.0240796	2.10096	2.10096	VVA up vs Control	7.70514	2.75316	2.85852	1
4178	7896704	---		---	0.0372736	0.0372736	2.10136	2.10136	VVA up vs Control	6.22136	2.75455	3.54206	1
26024	8114787	NM 005471 // GNPDA1 // glucosamine-6-phosphate deaminase 1 // 5q21 // 10007 ///	GNPDA1	NM 005471	0.0123902	0.0123902	2.10461	2.10461	VVA up vs Control	10.3159	2.76602	2.14507	1
9642	7950597	NM 001293 // CLNS1A // chloride channel, nucleotide-sensitive, 1A // 11q13.5-q14	CLNS1A	NM 001293	0.0457676	0.0457676	2.10654	2.10654	VVA up vs Control	5.58218	2.77286	3.97388	1
31619	8167656	NM 001005333 // MAGED1 // melanoma antigen family D, 1 // Xp11.23 // 9500 /// NM	MAGED1	NM 001005333	0.0075897	0.0075897	2.10788	2.10788	VVA up vs Control	12.5508	2.77758	1.77045	1
8645	7941243	NM 006268 // DPF2 // D4, zinc and double PHD fingers family 2 // 11q13 // 5977 /	DPF2	NM 006268	0.0079501	0.0079501	2.10792	2.10792	VVA up vs Control	12.327	2.77775	1.80271	1
19622	8051066	NM 002437 // MPV17 // MpV17 mitochondrial inner membrane protein // 2p23.3 // 43	MPV17	NM 002437	0.0424244	0.0424244	2.1095	2.1095	VVA up vs Control	5.8142	2.78332	3.82969	1
30226	8154233	NM 014143 // CD274 // CD274 molecule // 9p24 // 29126 /// ENST00000381577 // CD2	CD274	NM 014143	0.00865078	0.00865078	2.10965	2.10965	VVA up vs Control	11.9263	2.78384	1.86736	1
2893	7895409	---		---	0.0356236	0.0356236	2.1102	2.1102	VVA up vs Control	6.36719	2.78579	3.50018	1
23071	8086028	NM 000404 // GLB1 // galactosidase, beta 1 // 3p21.33 // 2720 /// NM 001079811 /	GLB1	NM 000404	0.000958619	0.000958619	2.11118	2.11118	VVA up vs Control	25.7593	2.78926	0.866254	1
12784	7981992	NR 003336 // SNORD116-22 // small nucleolar RNA, C/D box 116-22 // 15q11.2 // 10	SNORD116-22	NR 003336	0.0175325	0.0175325	2.1116	2.1116	VVA up vs Control	8.89551	2.79074	2.5098	1
10032	7954196	NM 145792 // MGST1 // microsomal glutathione S-transferase 1 // 12p12.3-p12.1 //	MGST1	NM 145792	0.0415473	0.0415473	2.11168	2.11168	VVA up vs Control	5.87895	2.79103	3.798	1
26800	8122013	NM 032438 // L3MBTL3 // l(3)mbt-like 3 (Drosophila) // 6q23 // 84456 /// NM 0010	L3MBTL3	NM 032438	0.000930497	0.000930497	2.11329	2.11329	VVA up vs Control	26.0043	2.79672	0.860387	1
17595	8031223	NM 006669 // LILRB1 // leukocyte immunoglobulin-like receptor, subfamily B (with	LILRB1	NM 006669	0.0382968	0.0382968	2.11355	2.11355	VVA up vs Control	6.13499	2.79764	3.64811	1
4183	7896709	---		---	0.0184589	0.0184589	2.1153	2.1153	VVA up vs Control	8.69555	2.80384	2.57957	1
4718	7901895	NM 032852 // ATG4C // ATG4 autophagy related 4 homolog C (S. cerevisiae) // 1p31	ATG4C	NM 032852	0.0478294	0.0478294	2.11567	2.11567	VVA up vs Control	5.44959	2.80513	4.11793	1
32362	8174119	NM 001011657 // ZMAT1 // zinc finger, matrin type 1 // Xq21 // 84460 /// NM 0324	ZMAT1	NM 001011657	0.0376149	0.0376149	2.11638	2.11638	VVA up vs Control	6.19222	2.80765	3.62733	1
15262	8007483	NM 145041 // TMEM106A // transmembrane protein 106A // 17q21.31 // 113277 /// EN	TMEM106A	NM 145041	0.00256799	0.00256799	2.11867	2.11867	VVA up vs Control	18.6075	2.81577	1.21059	1
23686	8092578	NM 004454 // ETV5 // ets variant 5 // 3q28 // 2119 /// ENST00000306376 // ETV5 /	ETV5	NM 004454	0.0197137	0.0197137	2.11881	2.11881	VVA up vs Control	8.44405	2.81624	2.66815	1
24866	8103601	NM 001012967 // DDX60L // DEAD (Asp-Glu-Ala-Asp) box polypeptide 60-like // 4q32	DDX60L	NM 001012967	0.0311865	0.0311865	2.12156	2.12156	VVA up vs Control	6.80605	2.826	3.32175	1
5402	7908777	---		---	0.010362	0.010362	2.12159	2.12159	VVA up vs Control	11.0986	2.82611	2.0371	1
6597	7920903	NM 003145 // SSR2 // signal sequence receptor, beta (translocon-associated prote	SSR2	NM 003145	0.0125338	0.0125338	2.12274	2.12274	VVA up vs Control	10.2666	2.83018	2.20534	1
2411	7894926	---		---	0.0414643	0.0414643	2.12285	2.12285	VVA up vs Control	5.88517	2.83057	3.84773	1
6349	7918694	NM 001010922 // BCL2L15 // BCL2-like 15 // 1p13.2 // 440603 /// ENST00000369580	BCL2L15	NM 001010922	0.0211133	0.0211133	2.1235	2.1235	VVA up vs Control	8.18631	2.83287	2.7684	1
10006	7953981	NM 001987 // ETV6 // ets variant 6 // 12p13 // 2120 /// ENST00000266427 // ETV6	ETV6	NM 001987	0.00465775	0.00465775	2.13104	2.13104	VVA up vs Control	15.0745	2.8596	1.51759	1
26822	8122202	NM 001130173 // MYB // v-myb myeloblastosis viral oncogene homolog (avian) // 6q	MYB	NM 001130173	0.0110047	0.0110047	2.13192	2.13192	VVA up vs Control	10.831	2.86273	2.11446	1
23674	8092457	NM 005787 // ALG3 // asparagine-linked glycosylation 3, alpha-1,3- mannosyltrans	ALG3	NM 005787	0.0156672	0.0156672	2.13235	2.13235	VVA up vs Control	9.34178	2.86423	2.45283	1
11412	7969060	NM 001079673 // FNDC3A // fibronectin type III domain containing 3A // 13q14.2 /	FNDC3A	NM 001079673	0.0238249	0.0238249	2.13398	2.13398	VVA up vs Control	7.74344	2.87001	2.9651	1
12785	7981994	NR 003337 // SNORD116-23 // small nucleolar RNA, C/D box 116-23 // 15q11.2 // 10	SNORD116-23	NR 003337	0.00799421	0.00799421	2.13421	2.13421	VVA up vs Control	12.3005	2.87083	1.86713	1
14929	8004247	NM 001142798 // C17orf49 // chromosome 17 open reading frame 49 // 17p13.1 // 12	C17orf49	NM 001142798	0.000862828	0.000862828	2.13503	2.13503	VVA up vs Control	26.6337	2.87375	0.863191	1
23471	8090509	NM 004637 // RAB7A // RAB7A, member RAS oncogene family // 3q21.3 // 7879 /// EN	RAB7A	NM 004637	0.00329263	0.00329263	2.13696	2.13696	VVA up vs Control	17.0675	2.8806	1.35022	1
19277	8047272	NM 015535 // SPATS2L // spermatogenesis associated, serine-rich 2-like // 2q33.1	SPATS2L	NM 015535	3.76E-05	3.76E-05	2.13722	2.13722	VVA up vs Control	66.6881	2.88154	0.345674	1
21862	8073743	NM 006953 // UPK3A // uroplakin 3A // 22q13.31 // 7380 /// ENST00000216211 // UP	UPK3A	NM 006953	0.0442889	0.0442889	2.13806	2.13806	VVA up vs Control	5.68204	2.8845	4.06122	1
8993	7944803	NM 001130142 // VWA5A // von Willebrand factor A domain containing 5A // 11q23 /	VWA5A	NM 001130142	0.00139111	0.00139111	2.13834	2.13834	VVA up vs Control	22.8461	2.8855	1.01042	1
10462	7958828	NM 001143906 // TRAFD1 // TRAF-type zinc finger domain containing 1 // 12q // 10	TRAFD1	NM 001143906	0.0067079	0.0067079	2.13869	2.13869	VVA up vs Control	13.1598	2.88674	1.75489	1
11542	7970376	NM 032436 // ZNF828 // zinc finger protein 828 // 13q34 // 283489 /// ENST000003	ZNF828	NM 032436	0.0209006	0.0209006	2.13877	2.13877	VVA up vs Control	8.22406	2.88703	2.80838	1
15258	8007446	NM 005533 // IFI35 // interferon-induced protein 35 // 17q21 // 3430 /// ENST000	IFI35	NM 005533	0.000348491	0.000348491	2.14012	2.14012	VVA up vs Control	35.2027	2.89183	0.657185	1
3400	7895919	---		---	0.0387663	0.0387663	2.14242	2.14242	VVA up vs Control	6.09633	2.9	3.80557	1
16852	8023757	NM 006566 // CD226 // CD226 molecule // 18q22.3 // 10666 /// ENST00000280200 //	CD226	NM 006566	0.0335487	0.0335487	2.14542	2.14542	VVA up vs Control	6.56323	2.91065	3.54783	1
19464	8049246	NM 001017915 // INPP5D // inositol polyphosphate-5-phosphatase, 145kDa // 2q37.1	INPP5D	NM 001017915	0.0210484	0.0210484	2.14832	2.14832	VVA up vs Control	8.19778	2.92095	2.85048	1
3461	7895981	---		---	0.0127453	0.0127453	2.15112	2.15112	VVA up vs Control	10.1954	2.93093	2.29981	1
10152	7955441	NM 014033 // METTL7A // methyltransferase like 7A // 12q13.12 // 25840 /// ENST0	METTL7A	NM 014033	0.0178707	0.0178707	2.15171	2.15171	VVA up vs Control	8.821	2.93301	2.66003	1
2503	7895018	---		---	0.0224482	0.0224482	2.15289	2.15289	VVA up vs Control	7.95985	2.93721	2.95203	1
29365	8146544	NM 001077619 // UBXN2B // UBX domain protein 2B // 8q12.1 // 137886 /// ENST0000	UBXN2B	NM 001077619	0.0381158	0.0381158	2.1529	2.1529	VVA up vs Control	6.15005	2.93724	3.82077	1

Column #	Transcript Cluster ID	gene_assignment	Gene Symbol	RefSeq	p-value(Group)	p-value(VVA vs. Control)	Ratio(VVA vs. Control)	Fold-Change(VVA vs. Control)	Fold-Change(VVA vs. Control) (Description)	F(Group)	SS(Group)	SS(Error)	F(Error)
8573	7940479	NM 016499 // TMEM216 // transmembrane protein 216 // 11q13.1 // 51259 /// ENST00	TMEM216	NM 016499	0.00434832	0.00434832	2.15378	2.15378	VVA up vs Control	15.4556	2.94041	1.52198	1
22828	8083673	NM 001042706 // IQCJ // IQ motif containing J // 3q25.32 // 654502 /// NM 001042	IQCJ	NM 001042706	0.0033916	0.0033916	2.1546	2.1546	VVA up vs Control	16.8904	2.94332	1.39408	1
4174	7896700	---		---	0.0171367	0.0171367	2.15547	2.15547	VVA up vs Control	8.98505	2.9464	2.62338	1
23702	8092726	NM 021101 // CLDN1 // claudin 1 // 3q28-q29 // 9076 /// ENST00000295522 // CLDN1	CLDN1	NM 021101	0.00465065	0.00465065	2.15591	2.15591	VVA up vs Control	15.0828	2.94798	1.56362	1
16100	8016387	NM 024320 // ATAD4 // ATPase family, AAA domain containing 4 // 17q21.32 // 7917	ATAD4	NM 024320	0.00242177	0.00242177	2.15629	2.15629	VVA up vs Control	18.9851	2.94933	1.2428	1
27182	8125556	NM 033554 // HLA-DPA1 // major histocompatibility complex, class II, DP alpha 1	HLA-DPA1	NM 033554	0.020777	0.020777	2.15692	2.15692	VVA up vs Control	8.24623	2.95156	2.86343	1
32944	8178891	NM 033554 // HLA-DPA1 // major histocompatibility complex, class II, DP alpha 1	HLA-DPA1	NM 033554	0.020777	0.020777	2.15692	2.15692	VVA up vs Control	8.24623	2.95156	2.86343	1
4192	7896718	---		---	0.0215894	0.0215894	2.15922	2.15922	VVA up vs Control	8.10351	2.95975	2.92195	1
29713	8149448	NM 002445 // MSR1 // macrophage scavenger receptor 1 // 8p22 // 4481 /// NM 1387	MSR1	NM 002445	0.000361686	0.000361686	2.15924	2.15924	VVA up vs Control	34.8117	2.95983	0.680191	1
1552	7894057	---		---	0.00828913	0.00828913	2.16423	2.16423	VVA up vs Control	12.1278	2.97761	1.96415	1
4162	7896688	---		---	0.018892	0.018892	2.16433	2.16433	VVA up vs Control	8.60636	2.97796	2.76815	1
10467	7958884	NM 016816 // OAS1 // 2',5'-oligoadenylate synthetase 1, 40/46kDa // 12q24.1 // 4	OAS1	NM 016816	0.00721637	0.00721637	2.16553	2.16553	VVA up vs Control	12.7972	2.98225	1.86431	1
8795	7942824	NM 001031 // RPS28 // ribosomal protein S28 // 19p13.2 // 6234 /// ENST000004170	RPS28	NM 001031	0.0031795	0.0031795	2.16574	2.16574	VVA up vs Control	17.2783	2.98299	1.38115	1
20880	8064388	NM 033409 // C20orf54 // chromosome 20 open reading frame 54 // 20p13 // 113278	C20orf54	NM 033409	0.0120532	0.0120532	2.16657	2.16657	VVA up vs Control	10.4343	2.98597	2.28936	1
4875	7903507	NM 001010883 // FAM102B // family with sequence similarity 102, member B // 1p13	FAM102B	NM 001010883	0.00453115	0.00453115	2.17015	2.17015	VVA up vs Control	15.2265	2.99871	1.57552	1
22314	8078286	NM 000965 // RARB // retinoic acid receptor, beta // 3p24 // 5915 /// NM 016152	RARB	NM 000965	0.0130231	0.0130231	2.17038	2.17038	VVA up vs Control	10.1041	2.99954	2.37491	1
19860	8053484	NM 003896 // ST3GAL5 // ST3 beta-galactoside alpha-2,3-sialyltransferase 5 // 2p	ST3GAL5	NM 003896	0.0189996	0.0189996	2.1705	2.1705	VVA up vs Control	8.5846	2.99995	2.79566	1
4945	7904287	NM 001767 // CD2 // CD2 molecule // 1p13.1 // 914 /// ENST00000369478 // CD2 //	CD2	NM 001767	0.0470176	0.0470176	2.17723	2.17723	VVA up vs Control	5.50091	3.024	4.39781	1
22616	8081431	NM 001627 // ALCAM // activated leukocyte cell adhesion molecule // 3q13.1 // 21	ALCAM	NM 001627	0.0145844	0.0145844	2.18036	2.18036	VVA up vs Control	9.63281	3.03517	2.52069	1
11122	7965812	NM 024312 // GNPTAB // N-acetylglucosamine-1-phosphate transferase, alpha and be	GNPTAB	NM 024312	0.0305858	0.0305858	2.18147	2.18147	VVA up vs Control	6.87153	3.03912	3.53822	1
22081	8075709	NM 030643 // APOL4 // apolipoprotein L, 4 // 22q11.2-q13.2 // 80832 /// NM 14566	APOL4	NM 030643	0.00728219	0.00728219	2.18154	2.18154	VVA up vs Control	12.7526	3.03936	1.90665	1
1549	7894054	---		---	0.0074162	0.0074162	2.18282	2.18282	VVA up vs Control	12.6634	3.04395	1.92299	1
21165	8067040	NM 012340 // NFATC2 // nuclear factor of activated T-cells, cytoplasmic, calcine	NFATC2	NM 012340	0.00194501	0.00194501	2.18293	2.18293	VVA up vs Control	20.4476	3.04433	1.19107	1
19413	8048717	NM 152386 // SGPP2 // sphingosine-1-phosphate phosphotase 2 // 2q36.1 // 130367	SGPP2	NM 152386	0.0369815	0.0369815	2.18503	2.18503	VVA up vs Control	6.24658	3.05184	3.90849	1
8203	7937508	NM 004357 // CD151 // CD151 molecule (Raph blood group) // 11p15.5 // 977 /// NM	CD151	NM 004357	0.0297728	0.0297728	2.18574	2.18574	VVA up vs Control	6.9628	3.05438	3.50937	1
18687	8041940	NM 172311 // STON1-GTF2A1L // STON1-GTF2A1L readthrough transcript // 2p16.3 //	STON1-GTF2A1L	NM 172311	0.0360244	0.0360244	2.18579	2.18579	VVA up vs Control	6.33098	3.05454	3.8598	1
8760	7942569	NM 001145211 // SLCO2B1 // solute carrier organic anion transporter family, memb	SLCO2B1	NM 001145211	0.00970379	0.00970379	2.18638	2.18638	VVA up vs Control	11.3951	3.05666	2.14595	1
11012	7964701	NM 002076 // GNS // glucosamine (N-acetyl)-6-sulfatase // 12q14 // 2799 /// ENST	GNS	NM 002076	0.00504846	0.00504846	2.18721	2.18721	VVA up vs Control	14.6362	3.05965	1.67238	1
17006	8025395	NM 001031 // RPS28 // ribosomal protein S28 // 19p13.2 // 6234 /// ENST000004170	RPS28	NM 001031	0.00289633	0.00289633	2.18826	2.18826	VVA up vs Control	17.85	3.06338	1.37295	1
1827	7894335	---		---	0.0387517	0.0387517	2.18856	2.18856	VVA up vs Control	6.09752	3.06445	4.02059	1
6898	7924029	NM 001017402 // LAMB3 // laminin, beta 3 // 1q32 // 3914 /// NM 001127641 // LAM	LAMB3	NM 001017402	0.00984207	0.00984207	2.18909	2.18909	VVA up vs Control	11.3307	3.06634	2.16497	1
9619	7950391	NM 173582 // PGM2L1 // phosphoglucomutase 2-like 1 // 11q13.4 // 283209 /// ENST	PGM2L1	NM 173582	0.00927279	0.00927279	2.19259	2.19259	VVA up vs Control	11.6033	3.07886	2.12274	1
25333	8108217	NM 000358 // TGFBI // transforming growth factor, beta-induced, 68kDa // 5q31 //	TGFBI	NM 000358	0.00989359	0.00989359	2.1943	2.1943	VVA up vs Control	11.3071	3.08501	2.18271	1
6095	7915955	NM 019073 // SPATA6 // spermatogenesis associated 6 // 1p33 // 54558 /// ENST000	SPATA6	NM 019073	0.0205531	0.0205531	2.20084	2.20084	VVA up vs Control	8.2868	3.10841	3.00083	1
6491	7919800	NM 004079 // CTSS // cathepsin S // 1q21 // 1520 /// ENST00000368985 // CTSS //	CTSS	NM 004079	0.010365	0.010365	2.20215	2.20215	VVA up vs Control	11.0973	3.1131	2.24423	1
28301	8136849	NM 001143679 // GSTK1 // glutathione S-transferase kappa 1 // 7q35 // 373156 ///	GSTK1	NM 001143679	0.0270425	0.0270425	2.20338	2.20338	VVA up vs Control	7.29407	3.11751	3.41923	1
21804	8073088	NM 021822 // APOBEC3G // apolipoprotein B mRNA editing enzyme, catalytic polypep	APOBEC3G	NM 021822	0.00220314	0.00220314	2.20395	2.20395	VVA up vs Control	19.6064	3.11953	1.27286	1
10510	7959267	NM 002560 // P2RX4 // purinergic receptor P2X, ligand-gated ion channel, 4 // 12	P2RX4	NM 002560	0.00245669	0.00245669	2.20625	2.20625	VVA up vs Control	18.8924	3.12777	1.32446	1
8016	7935251	NM 015631 // TCTN3 // tectonic family member 3 // 10q24.1 // 26123 /// NM 001143	TCTN3	NM 015631	0.0488102	0.0488102	2.21249	2.21249	VVA up vs Control	5.38905	3.15015	4.67637	1
25798	8112803	NM 005779 // LHFPL2 // lipoma HMGIC fusion partner-like 2 // 5q14.1 // 10184 ///	LHFPL2	NM 005779	0.0323027	0.0323027	2.21312	2.21312	VVA up vs Control	6.6885	3.15239	3.77052	1
24786	8102950	NM 003866 // INPP4B // inositol polyphosphate-4-phosphatase, type II, 105kDa //	INPP4B	NM 003866	0.00168926	0.00168926	2.21423	2.21423	VVA up vs Control	21.4318	3.15639	1.17821	1
6238	7917514	---		---	0.010925	0.010925	2.21453	2.21453	VVA up vs Control	10.8631	3.15746	2.32527	1
9946	7953428	NM 000616 // CD4 // CD4 molecule // 12pter-p12 // 920 /// ENST00000011653 // CD4	CD4	NM 000616	0.0199272	0.0199272	2.21555	2.21555	VVA up vs Control	8.40326	3.16113	3.00943	1
25999	8114536	NM 198282 // TMEM173 // transmembrane protein 173 // 5q31.2 // 340061 /// ENST00	TMEM173	NM 198282	0.00343504	0.00343504	2.21559	2.21559	VVA up vs Control	16.8147	3.16127	1.50405	1
2185	7894698	---		---	0.00205551	0.00205551	2.21671	2.21671	VVA up vs Control	20.0713	3.16527	1.26161	1
10398	7957966	NM 002465 // MYBPC1 // myosin binding protein C, slow type // 12q23.2 // 4604 //	MYBPC1	NM 002465	0.00298619	0.00298619	2.21733	2.21733	VVA up vs Control	17.6612	3.1675	1.43478	1
8225	7937802	NM 004356 // CD81 // CD81 molecule // 11p15.5 // 975 /// ENST00000263645 // CD81	CD81	NM 004356	0.00496945	0.00496945	2.21808	2.21808	VVA up vs Control	14.7213	3.1702	1.72278	1
18965	8044391	NM 006343 // MERTK // c-mer proto-oncogene tyrosine kinase // 2q14.1 // 10461 //	MERTK	NM 006343	0.000540198	0.000540198	2.21893	2.21893	VVA up vs Control	30.8161	3.17324	0.823787	1
8457	7939839	NM 002843 // PTPRJ // protein tyrosine phosphatase, receptor type, J // 11p11.2	PTPRJ	NM 002843	0.0492331	0.0492331	2.21927	2.21927	VVA up vs Control	5.36342	3.17445	4.73497	1
24211	8097657	NM 005900 // SMAD1 // SMAD family member 1 // 4q31 // 4086 /// NM 001003688 // S	SMAD1	NM 005900	0.00494518	0.00494518	2.22014	2.22014	VVA up vs Control	14.7478	3.17761	1.72371	1
15034	8005471	NM 001031 // RPS28 // ribosomal protein S28 // 19p13.2 // 6234 /// ENST000004170	RPS28	NM 001031	0.00344974	0.00344974	2.22437	2.22437	VVA up vs Control	16.7894	3.19277	1.52133	1
9641	7950578	NM 001128620 // PAK1 // p21 protein (Cdc42/Rac)-activated kinase 1 // 11q13-q14	PAK1	NM 001128620	0.00353706	0.00353706	2.22923	2.22923	VVA up vs Control	16.6415	3.21023	1.54323	1
6507	7919984	NM 003944 // SELENBP1 // selenium binding protein 1 // 1q21-q22 // 8991 /// ENST	SELENBP1	NM 003944	0.0212059	0.0212059	2.23321	2.23321	VVA up vs Control	8.17003	3.22454	3.15744	1
27544	8129458	NM 033515 // ARHGAP18 // Rho GTPase activating protein 18 // 6q22.33 // 93663 //	ARHGAP18	NM 033515	0.00775714	0.00775714	2.23538	2.23538	VVA up vs Control	12.4452	3.23233	2.0778	1
18907	8043718	NM 001862 // COX5B // cytochrome c oxidase subunit Vb // 2cen-q13 // 1329 /// EN	COX5B	NM 001862	0.0106885	0.0106885	2.23817	2.23817	VVA up vs Control	10.9601	3.24238	2.36667	1
1955	7894465	---		---	0.0301402	0.0301402	2.23927	2.23927	VVA up vs Control	6.92117	3.24633	3.75235	1
2730	7895246	---		---	0.0177974	0.0177974	2.24052	2.24052	VVA up vs Control	8.837	3.25083	2.94292	1
13036	7984112	NM 016530 // RAB8B // RAB8B, member RAS oncogene family // 15q22.2 // 51762 ///	RAB8B	NM 016530	0.0239167	0.0239167	2.24106	2.24106	VVA up vs Control	7.72958	3.25275	3.36655	1
24728	8102482	NM 014822 // SEC24D // SEC24 family, member D (S. cerevisiae) // 4q26 // 9871 //	SEC24D	NM 014822	0.00855195	0.00855195	2.24152	2.24152	VVA up vs Control	11.9803	3.2544	2.17317	1
9325	7947815	NM 001610 // ACP2 // acid phosphatase 2, lysosomal // 11p11.2-p11.11|11p12-p11 /	ACP2	NM 001610	0.00549084	0.00549084	2.24268	2.24268	VVA up vs Control	14.1886	3.25859	1.8373	1
22483	8080144	NM 001003931 // PARP3 // poly (ADP-ribose) polymerase family, member 3 // 3p21.3	PARP3	NM 001003931	0.021157	0.021157	2.24688	2.24688	VVA up vs Control	8.17862	3.27372	3.20222	1
22870	8083996	---		---	0.0109877	0.0109877	2.24852	2.24852	VVA up vs Control	10.8379	3.2796	2.42084	1
10762	7961891	NM 030762 // BHLHE41 // basic helix-loop-helix family, member e41 // 12p11.23-p1	BHLHE41	NM 030762	0.0268759	0.0268759	2.24943	2.24943	VVA up vs Control	7.31564	3.28288	3.58999	1
20281	8058147	NM 131916 // PPIL3 // peptidylprolyl isomerase (cyclophilin)-like 3 // 2q33.1 //	PPIL3	NM 131916	0.0316722	0.0316722	2.24957	2.24957	VVA up vs Control	6.75425	3.28339	3.88897	1
28359	8137250	NM 015660 // GIMAP2 // GTPase, IMAP family member 2 // 7q36.1 // 26157 /// ENST0	GIMAP2	NM 015660	0.0297622	0.0297622	2.25002	2.25002	VVA up vs Control	6.96401	3.28501	3.7737	1
16577	8021208	NM 002396 // ME2 // malic enzyme 2, NAD(+)-dependent, mitochondrial // 6p25-p24|	ME2	NM 002396	0.0147509	0.0147509	2.25057	2.25057	VVA up vs Control	9.58631	3.28698	2.74306	1
33067	8180100	NM 033554 // HLA-DPA1 // major histocompatibility complex, class II, DP alpha 1	HLA-DPA1	NM 033554	0.0178805	0.0178805	2.25068	2.25068	VVA up vs Control	8.81887	3.28741	2.98216	1
13773	7992071	NM 005823 // MSLN // mesothelin // 16p13.3 // 10232 /// NM 013404 // MSLN // mes	MSLN	NM 005823	0.0112239	0.0112239	2.25227	2.25227	VVA up vs Control	10.7442	3.29311	2.452	1
15842	8013788	NM 004475 // FLOT2 // flotillin 2 // 17q11-q12 // 2319 /// ENST00000394908 // FL	FLOT2	NM 004475	0.00437474	0.00437474	2.25257	2.25257	VVA up vs Control	15.4218	3.29419	1.70885	1
33142	8180260	---		---	0.00887161	0.00887161	2.25342	2.25342	VVA up vs Control	11.8084	3.29724	2.23383	1
9938	7953341	NM 018009 // TAPBPL // TAP binding protein-like // 12p13.31 // 55080 /// ENST000	TAPBPL	NM 018009	0.010688	0.010688	2.25441	2.25441	VVA up vs Control	10.9603	3.30082	2.40928	1
6239	7917516	NM 002053 // GBP1 // guanylate binding protein 1, interferon-inducible, 67kDa //	GBP1	NM 002053	0.00478186	0.00478186	2.25852	2.25852	VVA up vs Control	14.9304	3.31565	1.77659	1
24080	8096425	NM 001145065 // FAM190A // family with sequence similarity 190, member A // 4q22	FAM190A	NM 001145065	0.0134782	0.0134782	2.26349	2.26349	VVA up vs Control	9.95965	3.33356	2.67765	1
10469	7958913	NM 002535 // OAS2 // 2'-5'-oligoadenylate synthetase 2, 69/71kDa // 12q24.2 // 4	OAS2	NM 002535	0.000498144	0.000498144	2.26973	2.26973	VVA up vs Control	31.5912	3.35604	0.849866	1
23628	8092035	NM 024947 // PHC3 // polyhomeotic homolog 3 (Drosophila) // 3q26.2 // 80012 ///	PHC3	NM 024947	0.0427809	0.0427809	2.27121	2.27121	VVA up vs Control	5.78836	3.3614	4.64573	1
20415	8059650	NM 080424 // SP110 // SP110 nuclear body protein // 2q37.1 // 3431 /// NM 004509	SP110	NM 080424	0.0108372	0.0108372	2.28521	2.28521	VVA up vs Control	10.8988	3.41196	2.50446	1
8934	7944185	NM 000073 // CD3G // CD3g molecule, gamma (CD3-TCR complex) // 11q23 // 917 ///	CD3G	NM 000073	0.0251103	0.0251103	2.28721	2.28721	VVA up vs Control	7.55521	3.41916	3.62045	1
18778	8042801	NM 080916 // DGUOK // deoxyguanosine kinase // 2p13 // 1716 /// NM 080918 // DGU	DGUOK	NM 080916	0.0187683	0.0187683	2.28996	2.28996	VVA up vs Control	8.63157	3.4291	3.17819	1
24865	8103563	NM 017631 // DDX60 // DEAD (Asp-Glu-Ala-Asp) box polypeptide 60 // 4q32.3 // 556	DDX60	NM 017631	0.00269696	0.00269696	2.29132	2.29132	VVA up vs Control	18.2962	3.43404	1.50153	1
25711	8112070	---		---	0.00842405	0.00842405	2.29346	2.29346	VVA up vs Control	12.0514	3.44177	2.28473	1
19326	8047763	---		---	0.0278049	0.0278049	2.29408	2.29408	VVA up vs Control	7.19744	3.444	3.82802	1
20145	8056102	NM 014880 // CD302 // CD302 molecule // 2q24.2 // 9936 /// ENST00000259053 // CD	CD302	NM 014880	0.0353791	0.0353791	2.29897	2.29897	VVA up vs Control	6.38953	3.46169	4.33421	1
3519	7896039	---		---	0.00576955	0.00576955	2.30086	2.30086	VVA up vs Control	13.9293	3.46852	1.99207	1
23246	8088247	NM 001128615 // ARHGEF3 // Rho guanine nucleotide exchange factor (GEF) 3 // 3p2	ARHGEF3	NM 001128615	0.00968265	0.00968265	2.3015	2.3015	VVA up vs Control	11.405	3.47085	2.43461	1
18942	8044127	NM 024093 // C2orf49 // chromosome 2 open reading frame 49 // 2q12.1 // 79074 //	C2orf49	NM 024093	0.0261246	0.0261246	2.30655	2.30655	VVA up vs Control	7.41507	3.48912	3.76436	1
6877	7923812	NM 003929 // RAB7L1 // RAB7, member RAS oncogene family-like 1 // 1q32 // 8934 /	RAB7L1	NM 003929	0.00179582	0.00179582	2.30693	2.30693	VVA up vs Control	21.0005	3.49048	1.32968	1
11908	7973564	NM 176783 // PSME1 // proteasome (prosome, macropain) activator subunit 1 (PA28	PSME1	NM 176783	0.00706316	0.00706316	2.31299	2.31299	VVA up vs Control	12.903	3.51245	2.17776	1
25673	8111677	NM 002310 // LIFR // leukemia inhibitory factor receptor alpha // 5p13-p12 // 39	LIFR	NM 002310	0.0226785	0.0226785	2.3136	2.3136	VVA up vs Control	7.92249	3.51463	3.54902	1
3788	7896310	---		---	0.0172306	0.0172306	2.31431	2.31431	VVA up vs Control	8.96357	3.51722	3.13912	1
9612	7950307	NM 003355 // UCP2 // uncoupling protein 2 (mitochondrial, proton carrier) // 11q	UCP2	NM 003355	0.0148781	0.0148781	2.31518	2.31518	VVA up vs Control	9.55125	3.52038	2.94863	1
2585	7895101	---		---	0.00361103	0.00361103	2.31711	2.31711	VVA up vs Control	16.5198	3.52738	1.70819	1
14285	7997712	NM 002163 // IRF8 // interferon regulatory factor 8 // 16q24.1 // 3394 /// ENST0	IRF8	NM 002163	0.0230898	0.0230898	2.31875	2.31875	VVA up vs Control	7.85697	3.53329	3.59762	1
30840	8160332	NM 004529 // MLLT3 // myeloid/lymphoid or mixed-lineage leukemia (trithorax homo	MLLT3	NM 004529	0.0258966	0.0258966	2.32325	2.32325	VVA up vs Control	7.44598	3.54961	3.81372	1
14478	7999588	NM 003561 // PLA2G10 // phospholipase A2, group X // 16p13.1-p12 // 8399 /// ENS	PLA2G10	NM 003561	0.0224552	0.0224552	2.32427	2.32427	VVA up vs Control	7.9587	3.55332	3.57175	1
26422	8118158	NM 004847 // AIF1 // allograft inflammatory factor 1 // 6p21.3 // 199 /// NM 001	AIF1	NM 004847	0.00712172	0.00712172	2.32695	2.32695	VVA up vs Control	12.8622	3.56302	2.21612	1
32851	8177996	NM 004847 // AIF1 // allograft inflammatory factor 1 // 6p21.3 // 199 /// NM 001	AIF1	NM 004847	0.00712172	0.00712172	2.32695	2.32695	VVA up vs Control	12.8622	3.56302	2.21612	1
32987	8179276	NM 004847 // AIF1 // allograft inflammatory factor 1 // 6p21.3 // 199 /// NM 001	AIF1	NM 004847	0.00712172	0.00712172	2.32695	2.32695	VVA up vs Control	12.8622	3.56302	2.21612	1
21770	8072744	NM 013416 // NCF4 // neutrophil cytosolic factor 4, 40kDa // 22q13.1 // 4689 ///	NCF4	NM 013416	0.0238433	0.0238433	2.32818	2.32818	VVA up vs Control	7.74066	3.5675	3.68703	1
8599	7940711	NM 199337 // TMEM179B // transmembrane protein 179B // 11q12.3 // 374395 /// ENS	TMEM179B	NM 199337	0.00476964	0.00476964	2.32957	2.32957	VVA up vs Control	14.9444	3.57254	1.91245	1
24616	8101260	NM 058172 // ANTXR2 // anthrax toxin receptor 2 // 4q21.21 // 118429 /// NM 0011	ANTXR2	NM 058172	0.00932464	0.00932464	2.331	2.331	VVA up vs Control	11.5776	3.57774	2.47217	1
3961	7896484	---		---	0.0028395	0.0028395	2.3377	2.3377	VVA up vs Control	17.9731	3.60204	1.6033	1
5414	7908917	NM 006763 // BTG2 // BTG family, member 2 // 1q32 // 7832 /// ENST00000290551 //	BTG2	NM 006763	0.0397986	0.0397986	2.33935	2.33935	VVA up vs Control	6.01339	3.60802	4.79998	1
20394	8059350	NM 001039569 // AP1S3 // adaptor-related protein complex 1, sigma 3 subunit // 2	AP1S3	NM 001039569	0.0286686	0.0286686	2.34602	2.34602	VVA up vs Control	7.09195	3.63222	4.09729	1
9604	7950248	NM 014824 // FCHSD2 // FCH and double SH3 domains 2 // 11q13.4 // 9873 /// ENST0	FCHSD2	NM 014824	0.0138357	0.0138357	2.34985	2.34985	VVA up vs Control	9.85042	3.64614	2.96121	1
23593	8091715	NM 020169 // LXN // latexin // 3q25.32 // 56925 /// ENST00000264265 // LXN // la	LXN	NM 020169	0.00848913	0.00848913	2.35442	2.35442	VVA up vs Control	12.015	3.66274	2.43877	1
24644	8101648	NM 016245 // HSD17B11 // hydroxysteroid (17-beta) dehydrogenase 11 // 4q22.1 //	HSD17B11	NM 016245	0.0263284	0.0263284	2.35591	2.35591	VVA up vs Control	7.38775	3.66817	3.97217	1
7229	7927186	NM 032023 // RASSF4 // Ras association (RalGDS/AF-6) domain family member 4 // 1	RASSF4	NM 032023	0.00682204	0.00682204	2.35817	2.35817	VVA up vs Control	13.0755	3.67637	2.24933	1
24835	8103389	NM 001334 // CTSO // cathepsin O // 4q31-q32 // 1519 /// ENST00000281527 // CTSO	CTSO	NM 001334	0.0102059	0.0102059	2.3622	2.3622	VVA up vs Control	11.1667	3.69103	2.64431	1
12621	7980580	NM 000153 // GALC // galactosylceramidase // 14q31 // 2581 /// NM 001037525 // G	GALC	NM 000153	0.0164709	0.0164709	2.36619	2.36619	VVA up vs Control	9.1417	3.70551	3.24273	1
27564	8129677	NM 001143676 // SGK1 // serum/glucocorticoid regulated kinase 1 // 6q23 // 6446	SGK1	NM 001143676	0.0189732	0.0189732	2.36633	2.36633	VVA up vs Control	8.58994	3.70605	3.45153	1
8948	7944341	NM 006760 // UPK2 // uroplakin 2 // 11q23 // 7379 /// ENST00000264031 // UPK2 //	UPK2	NM 006760	0.00860501	0.00860501	2.36685	2.36685	VVA up vs Control	11.9512	3.70795	2.48205	1
26452	8118571	NM 002800 // PSMB9 // proteasome (prosome, macropain) subunit, beta type, 9 (lar	PSMB9	NM 002800	0.0227619	0.0227619	2.37152	2.37152	VVA up vs Control	7.90909	3.72493	3.76775	1
32871	8178211	NM 002800 // PSMB9 // proteasome (prosome, macropain) subunit, beta type, 9 (lar	PSMB9	NM 002800	0.0227619	0.0227619	2.37152	2.37152	VVA up vs Control	7.90909	3.72493	3.76775	1
33005	8179495	NM 002800 // PSMB9 // proteasome (prosome, macropain) subunit, beta type, 9 (lar	PSMB9	NM 002800	0.0227619	0.0227619	2.37152	2.37152	VVA up vs Control	7.90909	3.72493	3.76775	1
4188	7896714	---		---	0.0145417	0.0145417	2.37225	2.37225	VVA up vs Control	9.64482	3.72756	3.09186	1
8810	7942957	NM 007173 // PRSS23 // protease, serine, 23 // 11q14.1 // 11098 /// ENST00000280	PRSS23	NM 007173	0.0102381	0.0102381	2.37712	2.37712	VVA up vs Control	11.1526	3.74527	2.68657	1
21131	8066619	NM 006227 // PLTP // phospholipid transfer protein // 20q12-q13.1 // 5360 /// NM	PLTP	NM 006227	0.0153572	0.0153572	2.38775	2.38775	VVA up vs Control	9.42246	3.78397	3.21272	1
31736	8168557	NM 003022 // SH3BGRL // SH3 domain binding glutamic acid-rich protein like // Xq	SH3BGRL	NM 003022	0.0420944	0.0420944	2.3896	2.3896	VVA up vs Control	5.83836	3.79072	5.19422	1
9120	7945962	NM 003141 // TRIM21 // tripartite motif-containing 21 // 11p15.5 // 6737 /// ENS	TRIM21	NM 003141	0.00064731	0.00064731	2.39034	2.39034	VVA up vs Control	29.1419	3.79341	1.04136	1
6244	7917561	NM 052941 // GBP4 // guanylate binding protein 4 // 1p22.2 // 115361 /// ENST000	GBP4	NM 052941	0.00239361	0.00239361	2.3946	2.3946	VVA up vs Control	19.0611	3.80893	1.59862	1
25071	8105495	NR 024617 // PART1 // prostate androgen-regulated transcript 1 // 5q12.1 // 2585	PART1	NR 024617	0.0159515	0.0159515	2.39705	2.39705	VVA up vs Control	9.26956	3.81787	3.29498	1
25298	8107868	NM 020240 // CDC42SE2 // CDC42 small effector 2 // 5q23.3-q31.1 // 56990 /// NM	CDC42SE2	NM 020240	0.0144205	0.0144205	2.39776	2.39776	VVA up vs Control	9.67922	3.82046	3.15766	1
6242	7917532	NM 004120 // GBP2 // guanylate binding protein 2, interferon-inducible // 1p22.2	GBP2	NM 004120	0.00254104	0.00254104	2.39868	2.39868	VVA up vs Control	18.6751	3.82381	1.63804	1
1336	7893839	---		---	0.00401637	0.00401637	2.40015	2.40015	VVA up vs Control	15.9044	3.82918	1.9261	1
9656	7950731	NM 199418 // PRCP // prolylcarboxypeptidase (angiotensinase C) // 11q14 // 5547	PRCP	NM 199418	0.0361077	0.0361077	2.40353	2.40353	VVA up vs Control	6.32353	3.84149	4.85993	1
5282	7907297	NM 002022 // FMO4 // flavin containing monooxygenase 4 // 1q23-q25 // 2329 /// E	FMO4	NM 002022	0.00791129	0.00791129	2.41184	2.41184	VVA up vs Control	12.3505	3.8718	2.50794	1
33072	8180166	NM 003190 // TAPBP // TAP binding protein (tapasin) // 6p21.3 // 6892 /// NM 172	TAPBP	NM 003190	0.00194577	0.00194577	2.41534	2.41534	VVA up vs Control	20.445	3.88455	1.52	1
1072	7893574	---		---	0.0265321	0.0265321	2.41602	2.41602	VVA up vs Control	7.3607	3.88704	4.22464	1
2664	7895180	---		---	0.0217316	0.0217316	2.41699	2.41699	VVA up vs Control	8.07924	3.89055	3.85239	1
26059	8115147	NM 001025159 // CD74 // CD74 molecule, major histocompatibility complex, class I	CD74	NM 001025159	0.00803313	0.00803313	2.419	2.419	VVA up vs Control	12.2773	3.89789	2.53991	1
3586	7896108	---		---	0.033013	0.033013	2.42217	2.42217	VVA up vs Control	6.61635	3.90948	4.72705	1
32950	8178977	NM 172208 // TAPBP // TAP binding protein (tapasin) // 6p21.3 // 6892 /// NM 003	TAPBP	NM 172208	0.00190247	0.00190247	2.42404	2.42404	VVA up vs Control	20.5997	3.91628	1.52091	1
30196	8153959	NM 203447 // DOCK8 // dedicator of cytokinesis 8 // 9p24.3 // 81704 /// ENST0000	DOCK8	NM 203447	0.00467031	0.00467031	2.4259	2.4259	VVA up vs Control	15.0597	3.92309	2.08402	1
20148	8056184	NM 000888 // ITGB6 // integrin, beta 6 // 2q24.2 // 3694 /// ENST00000283249 //	ITGB6	NM 000888	0.00917599	0.00917599	2.43141	2.43141	VVA up vs Control	11.6518	3.9432	2.70736	1
6109	7916120	NM 015913 // TXNDC12 // thioredoxin domain containing 12 (endoplasmic reticulum)	TXNDC12	NM 015913	0.0144161	0.0144161	2.44173	2.44173	VVA up vs Control	9.68047	3.98088	3.28982	1
8199	7937476	NM 001004 // RPLP2 // ribosomal protein, large, P2 // 11p15.5-p15.4 // 6181 ///	RPLP2	NM 001004	0.0125326	0.0125326	2.44211	2.44211	VVA up vs Control	10.267	3.98225	3.10294	1
33230	8180348	---		---	0.015014	0.015014	2.44259	2.44259	VVA up vs Control	9.51419	3.984	3.34994	1
31531	8166784	NM 004615 // TSPAN7 // tetraspanin 7 // Xp11.4 // 7102 /// ENST00000378482 // TS	TSPAN7	NM 004615	0.00135725	0.00135725	2.44692	2.44692	VVA up vs Control	23.0305	3.99984	1.38941	1
28223	8136078	---		---	0.00537684	0.00537684	2.44692	2.44692	VVA up vs Control	14.2995	3.99982	2.23773	1
11188	7966448	NM 138341 // TMEM116 // transmembrane protein 116 // 12q24.13 // 89894 /// ENST0	TMEM116	NM 138341	0.017297	0.017297	2.44814	2.44814	VVA up vs Control	8.94846	4.00429	3.57987	1
32935	8178802				0.0217587	0.0217587	2.45162	2.45162	VVA up vs Control	8.07462	4.01699	3.97987	1
2912	7895428	---		---	0.00914093	0.00914093	2.4544	2.4544	VVA up vs Control	11.6695	4.02717	2.76082	1
8538	7940160	NM 015177 // DTX4 // deltex homolog 4 (Drosophila) // 11q12.1 // 23220 /// ENST0	DTX4	NM 015177	0.0140226	0.0140226	2.45825	2.45825	VVA up vs Control	9.79474	4.04122	3.30073	1
19433	8048940	NM 001080391 // SP100 // SP100 nuclear antigen // 2q37.1 // 6672 /// NM 003113 /	SP100	NM 001080391	0.0190151	0.0190151	2.45969	2.45969	VVA up vs Control	8.58148	4.0465	3.77231	1
33057	8180003				0.0149099	0.0149099	2.46345	2.46345	VVA up vs Control	9.54253	4.06024	3.40391	1
32936	8178811				0.0133789	0.0133789	2.47544	2.47544	VVA up vs Control	9.99062	4.10409	3.28636	1
28871	8141843	NM 006989 // RASA4 // RAS p21 protein activator 4 // 7q22 // 10156 /// NM 001079	RASA4	NM 006989	0.00616321	0.00616321	2.4786	2.4786	VVA up vs Control	13.5886	4.11565	2.423	1
31541	8166906	NM 001097579 // GPR34 // G protein-coupled receptor 34 // Xp11.4-p11.3 // 2857 /	GPR34	NM 001097579	0.000921169	0.000921169	2.48609	2.48609	VVA up vs Control	26.0876	4.14306	1.27051	1
32869	8178199				0.030224	0.030224	2.48701	2.48701	VVA up vs Control	6.91176	4.14645	4.79929	1
33004	8179489				0.030224	0.030224	2.48701	2.48701	VVA up vs Control	6.91176	4.14645	4.79929	1
6955	7924636	NM 014698 // TMEM63A // transmembrane protein 63A // 1q42.12 // 9725 /// ENST000	TMEM63A	NM 014698	0.03077	0.03077	2.49203	2.49203	VVA up vs Control	6.85128	4.16482	4.86311	1
4855	7903334	NM 003672 // CDC14A // CDC14 cell division cycle 14 homolog A (S. cerevisiae) //	CDC14A	NM 003672	0.00981913	0.00981913	2.49647	2.49647	VVA up vs Control	11.3413	4.18108	2.94926	1
27757	8131614	NM 001621 // AHR // aryl hydrocarbon receptor // 7p15 // 196 /// ENST00000401808	AHR	NM 001621	0.002575	0.002575	2.49754	2.49754	VVA up vs Control	18.5901	4.18499	1.80096	1

Column #	Transcript Cluster ID	gene_assignment	Gene Symbol	RefSeq	p-value(Group)	p-value(VVA vs. Control)	Ratio(VVA vs. Control)	Fold-Change(VVA vs. Control)	Fold-Change(VVA vs. Control) (Description)	F(Group)	SS(Group)	SS(Error)	F(Error)
23186	8087485	NM 003335 // UBA7 // ubiquitin-like modifier activating enzyme 7 // 3p21 // 7318	UBA7	NM 003335	0.00237894	0.00237894	2.503	2.503	VVA up vs Control	19.1011	4.20499	1.76115	1
12517	7979663	NM 198686 // RAB15 // RAB15, member RAS onocogene family // 14q23.3 // 376267 //	RAB15	NM 198686	0.00474392	0.00474392	2.51085	2.51085	VVA up vs Control	14.9739	4.23373	2.26192	1
8395	7939120	NM 002901 // RCN1 // reticulocalbin 1, EF-hand calcium binding domain // 11p13 /	RCN1	NM 002901	0.0241025	0.0241025	2.51249	2.51249	VVA up vs Control	7.70173	4.23975	4.40394	1
16773	8023043	NM 024430 // PSTPIP2 // proline-serine-threonine phosphatase interacting protein	PSTPIP2	NM 024430	0.00573116	0.00573116	2.52114	2.52114	VVA up vs Control	13.9641	4.27144	2.4471	1
10663	7960947	NM 000014 // A2M // alpha-2-macroglobulin // 12p13.3-p12.3 // 2 /// ENST00000318	A2M	NM 000014	0.00534065	0.00534065	2.52123	2.52123	VVA up vs Control	14.3354	4.27175	2.3839	1
26835	8122334	NM 178445 // CCRL1 // chemokine (C-C motif) receptor-like 1 // 3q22 // 51554 ///	CCRL1	NM 178445	0.0191501	0.0191501	2.52187	2.52187	VVA up vs Control	8.55444	4.27411	3.99709	1
870	7893372	---		---	0.0489171	0.0489171	2.52815	2.52815	VVA up vs Control	5.38254	4.29712	6.38675	1
26321	8117476	NM 006994 // BTN3A3 // butyrophilin, subfamily 3, member A3 // 6p21.3 // 10384 /	BTN3A3	NM 006994	0.0186408	0.0186408	2.54531	2.54531	VVA up vs Control	8.65777	4.36002	4.02877	1
25930	8113790	NM 178450 // MARCH3 // membrane-associated ring finger (C3HC4) 3 // 5q23.2 // 11	02-Mar	NM 178450	0.00517595	0.00517595	2.55102	2.55102	VVA up vs Control	14.5023	4.38097	2.4167	1
33282	8180400	---		---	0.00475892	0.00475892	2.55632	2.55632	VVA up vs Control	14.9567	4.40041	2.35369	1
990	7893492	---		---	0.0227472	0.0227472	2.55932	2.55932	VVA up vs Control	7.91145	4.41143	4.4608	1
33003	8179481	NM 019111 // HLA-DRA // major histocompatibility complex, class II, DR alpha //	HLA-DRA	NM 019111	0.00410436	0.00410436	2.56275	2.56275	VVA up vs Control	15.781	4.424	2.2427	1
18748	8042468	NM 001153 // ANXA4 // annexin A4 // 2p13 // 307 /// ENST00000394295 // ANXA4 //	ANXA4	NM 001153	0.029511	0.029511	2.57076	2.57076	VVA up vs Control	6.99286	4.4534	5.09479	1
15445	8009353	NM 181671 // PITPNC1 // phosphatidylinositol transfer protein, cytoplasmic 1 //	PITPNC1	NM 181671	0.000839213	0.000839213	2.57947	2.57947	VVA up vs Control	26.8681	4.48535	1.33552	1
5330	7907893	NM 001531 // MR1 // major histocompatibility complex, class I-related // 1q25.3	MR1	NM 001531	0.00717522	0.00717522	2.58029	2.58029	VVA up vs Control	12.8253	4.48834	2.79967	1
6740	7922404	NR 003940 // SNORD80 // small nucleolar RNA, C/D box 80 // 1q25.1 // 26774 /// B	SNORD80	NR 003940	0.00403754	0.00403754	2.58585	2.58585	VVA up vs Control	15.8744	4.50878	2.27223	1
28995	8143188	NM 194071 // CREB3L2 // cAMP responsive element binding protein 3-like 2 // 7q34	CREB3L2	NM 194071	0.00652568	0.00652568	2.58612	2.58612	VVA up vs Control	13.2982	4.50975	2.713	1
5189	7906400	NM 005531 // IFI16 // interferon, gamma-inducible protein 16 // 1q22 // 3428 ///	IFI16	NM 005531	0.0114048	0.0114048	2.58916	2.58916	VVA up vs Control	10.6742	4.52092	3.38828	1
25677	8111772	NM 001343 // DAB2 // disabled homolog 2, mitogen-responsive phosphoprotein (Dros	DAB2	NM 001343	0.0114914	0.0114914	2.58919	2.58919	VVA up vs Control	10.6412	4.52103	3.39889	1
7546	7930498	NM 016234 // ACSL5 // acyl-CoA synthetase long-chain family member 5 // 10q25.1-	ACSL5	NM 016234	0.005082	0.005082	2.59338	2.59338	VVA up vs Control	14.6006	4.5364	2.4856	1
17596	8031238	NM 006847 // LILRB4 // leukocyte immunoglobulin-like receptor, subfamily B (with	LILRB4	NM 006847	0.0178734	0.0178734	2.59699	2.59699	VVA up vs Control	8.82041	4.54965	4.12647	1
30594	8157605	NM 004099 // STOM // stomatin // 9q34.1 // 2040 /// NM 198194 // STOM // stomati	STOM	NM 004099	0.00435912	0.00435912	2.59733	2.59733	VVA up vs Control	15.4418	4.55092	2.35772	1
23078	8086125	NM 014831 // LBA1 // lupus brain antigen 1 // 3p22.2 // 9881 /// ENST00000301807	LBA1	NM 014831	0.00251992	0.00251992	2.6055	2.6055	VVA up vs Control	18.7286	4.58092	1.95676	1
26454	8118594	NM 002121 // HLA-DPB1 // major histocompatibility complex, class II, DP beta 1 /	HLA-DPB1	NM 002121	0.00961787	0.00961787	2.60725	2.60725	VVA up vs Control	11.4357	4.58732	3.20913	1
33138	8180256	---		---	0.0329098	0.0329098	2.60897	2.60897	VVA up vs Control	6.62671	4.59364	5.5456	1
11354	7968344	NM 001629 // ALOX5AP // arachidonate 5-lipoxygenase-activating protein // 13q12	ALOX5AP	NM 001629	0.00617057	0.00617057	2.61415	2.61415	VVA up vs Control	13.5825	4.61267	2.71683	1
31420	8165843	NM 004042 // ARSF // arylsulfatase F // Xp22.3 // 416 /// ENST00000359361 // ARS	ARSF	NM 004042	0.0036599	0.0036599	2.61447	2.61447	VVA up vs Control	16.4412	4.61385	2.24502	1
21763	8072710	NM 030641 // APOL6 // apolipoprotein L, 6 // 22q12.3 // 80830 /// ENST0000040965	APOL6	NM 030641	0.000349197	0.000349197	2.61662	2.61662	VVA up vs Control	35.1813	4.62175	1.05096	1
22868	8083978	NM 207015 // NAALADL2 // N-acetylated alpha-linked acidic dipeptidase-like 2 //	NAALADL2	NM 207015	0.00883828	0.00883828	2.63052	2.63052	VVA up vs Control	11.826	4.67279	3.16104	1
32868	8178193	NM 019111 // HLA-DRA // major histocompatibility complex, class II, DR alpha //	HLA-DRA	NM 019111	0.00377929	0.00377929	2.63862	2.63862	VVA up vs Control	16.2543	4.70255	2.31449	1
4894	7903753	NM 000848 // GSTM2 // glutathione S-transferase mu 2 (muscle) // 1p13.3 // 2946	GSTM2	NM 000848	0.0146312	0.0146312	2.64183	2.64183	VVA up vs Control	9.61967	4.71434	3.92058	1
26449	8118548	NM 019111 // HLA-DRA // major histocompatibility complex, class II, DR alpha //	HLA-DRA	NM 019111	0.00353573	0.00353573	2.64419	2.64419	VVA up vs Control	16.6438	4.72303	2.27017	1
7419	7929052	NM 001031683 // IFIT3 // interferon-induced protein with tetratricopeptide repea	IFIT3	NM 001031683	0.00303052	0.00303052	2.6484	2.6484	VVA up vs Control	17.5707	4.73849	2.15745	1
23385	8089544	NM 199511 // CCDC80 // coiled-coil domain containing 80 // 3q13.2 // 151887 ///	CCDC80	NM 199511	0.0432198	0.0432198	2.65061	2.65061	VVA up vs Control	5.75693	4.74659	6.59599	1
21801	8073068	NM 014508 // APOBEC3C // apolipoprotein B mRNA editing enzyme, catalytic polypep	APOBEC3C	NM 014508	0.00174897	0.00174897	2.65152	2.65152	VVA up vs Control	21.1861	4.74994	1.79361	1
26700	8121212	---		---	0.0399387	0.0399387	2.65184	2.65184	VVA up vs Control	6.00235	4.75111	6.33234	1
10730	7961524	NM 152321 // ERP27 // endoplasmic reticulum protein 27 // 12p12.3 // 121506 ///	ERP27	NM 152321	0.000615948	0.000615948	2.65496	2.65496	VVA up vs Control	29.594	4.76256	1.28744	1
31527	8166730	NM 000397 // CYBB // cytochrome b-245, beta polypeptide // Xp21.1 // 1536 /// EN	CYBB	NM 000397	0.0282381	0.0282381	2.65619	2.65619	VVA up vs Control	7.14402	4.76711	5.33829	1
12326	7977615	NM 198232 // RNASE1 // ribonuclease, RNase A family, 1 (pancreatic) // 14q11.2 /	RNASE1	NM 198232	0.0062598	0.0062598	2.65748	2.65748	VVA up vs Control	13.5092	4.77183	2.82583	1
24323	8098611	NM 003265 // TLR3 // toll-like receptor 3 // 4q35 // 7098 /// ENST00000296795 //	TLR3	NM 003265	0.0224938	0.0224938	2.65859	2.65859	VVA up vs Control	7.95241	4.77592	4.8045	1
7407	7928937	NM 004897 // MINPP1 // multiple inositol polyphosphate histidine phosphatase, 1	MINPP1	NM 004897	0.0379035	0.0379035	2.65984	2.65984	VVA up vs Control	6.16784	4.78049	6.20054	1
33007	8179519	NM 002121 // HLA-DPB1 // major histocompatibility complex, class II, DP beta 1 /	HLA-DPB1	NM 002121	0.0114771	0.0114771	2.6663	2.6663	VVA up vs Control	10.6466	4.80423	3.60996	1
28873	8141872	NM 001122838 // NAPEPLD // N-acyl phosphatidylethanolamine phospholipase D // 7q	NAPEPLD	NM 001122838	0.0493817	0.0493817	2.66906	2.66906	VVA up vs Control	5.35448	4.81439	7.19306	1
32349	8173979	NM 007052 // NOX1 // NADPH oxidase 1 // Xq22 // 27035 /// NM 013955 // NOX1 // N	NOX1	NM 007052	0.0304222	0.0304222	2.67475	2.67475	VVA up vs Control	6.88965	4.83529	5.61456	1
31204	8163618	NM 005118 // TNFSF15 // tumor necrosis factor (ligand) superfamily, member 15 //	TNFSF15	NM 005118	0.0355721	0.0355721	2.677	2.677	VVA up vs Control	6.37187	4.84358	6.0812	1
9084	7945663	ENST00000340134 // CTSD // cathepsin D // 11p15.5 // 1509 /// ENST00000382123 //	CTSD	ENST00000340134	0.00355095	0.00355095	2.67786	2.67786	VVA up vs Control	16.6184	4.84673	2.33318	1
9245	7946977	NM 006512 // SAA4 // serum amyloid A4, constitutive // 11p15.1-p14 // 6291 /// E	SAA4	NM 006512	0.0277076	0.0277076	2.68474	2.68474	VVA up vs Control	7.20958	4.872	5.40614	1
6302	7918149	---		---	0.0449912	0.0449912	2.68504	2.68504	VVA up vs Control	5.63409	4.87312	6.91948	1
13608	7990620	NM 005724 // TSPAN3 // tetraspanin 3 // 15q24.3 // 10099 /// NM 198902 // TSPAN3	TSPAN3	NM 005724	0.00682622	0.00682622	2.68658	2.68658	VVA up vs Control	13.0724	4.87878	2.98569	1
16374	8019486	NM 003004 // SECTM1 // secreted and transmembrane 1 // 17q25 // 6398 /// ENST000	SECTM1	NM 003004	0.0230878	0.0230878	2.69803	2.69803	VVA up vs Control	7.85729	4.92084	5.01022	1
7418	7929047	NM 001547 // IFIT2 // interferon-induced protein with tetratricopeptide repeats	IFIT2	NM 001547	0.0238782	0.0238782	2.69953	2.69953	VVA up vs Control	7.73538	4.92636	5.09488	1
9411	7948332	NM 004811 // LPXN // leupaxin // 11q12.1 // 9404 /// NM 001143995 // LPXN // leu	LPXN	NM 004811	0.0135085	0.0135085	2.7058	2.7058	VVA up vs Control	9.95024	4.94941	3.97933	1
2554	7895069	---		---	0.000370451	0.000370451	2.71058	2.71058	VVA up vs Control	34.5618	4.96697	1.1497	1
31566	8167185	NM 003254 // TIMP1 // TIMP metallopeptidase inhibitor 1 // Xp11.3-p11.23 // 7076	TIMP1	NM 003254	0.00656473	0.00656473	2.71857	2.71857	VVA up vs Control	13.2681	4.99633	3.01253	1
30073	8152522	NM 006209 // ENPP2 // ectonucleotide pyrophosphatase/phosphodiesterase 2 // 8q24	ENPP2	NM 006209	0.0478082	0.0478082	2.72262	2.72262	VVA up vs Control	5.45092	5.01123	7.3547	1
10397	7957962	NM 152323 // SPIC // Spi-C transcription factor (Spi-1/PU.1 related) // 12q23.2	SPIC	NM 152323	0.000934852	0.000934852	2.72701	2.72701	VVA up vs Control	25.9657	5.02737	1.54892	1
21800	8073062	NM 004900 // APOBEC3B // apolipoprotein B mRNA editing enzyme, catalytic polypep	APOBEC3B	NM 004900	0.00283296	0.00283296	2.72772	2.72772	VVA up vs Control	17.9875	5.02996	2.23709	1
6031	7915286	NM 000310 // PPT1 // palmitoyl-protein thioesterase 1 // 1p32 // 5538 /// NM 001	PPT1	NM 000310	0.00560948	0.00560948	2.72828	2.72828	VVA up vs Control	14.0763	5.03204	2.85987	1
4893	7903742	NM 000850 // GSTM4 // glutathione S-transferase mu 4 // 1p13.3 // 2948 /// NR 02	GSTM4	NM 000850	0.00471262	0.00471262	2.72879	2.72879	VVA up vs Control	15.0102	5.03392	2.68294	1
25855	8113250	NM 001040458 // ERAP1 // endoplasmic reticulum aminopeptidase 1 // 5q15 // 51752	ERAP1	NM 001040458	0.0263863	0.0263863	2.72884	2.72884	VVA up vs Control	7.38003	5.03408	5.45697	1
31714	8168424	NM 006517 // SLC16A2 // solute carrier family 16, member 2 (monocarboxylic acid	SLC16A2	NM 006517	0.000685347	0.000685347	2.73796	2.73796	VVA up vs Control	28.629	5.06762	1.41608	1
22851	8083839	NM 014373 // GPR160 // G protein-coupled receptor 160 // 3q26.2-q27 // 26996 ///	GPR160	NM 014373	0.00376542	0.00376542	2.7406	2.7406	VVA up vs Control	16.2756	5.07732	2.49566	1
20347	8058708	NM 173076 // ABCA12 // ATP-binding cassette, sub-family A (ABC1), member 12 // 2	ABCA12	NM 173076	0.0300455	0.0300455	2.74615	2.74615	VVA up vs Control	6.93183	5.0977	5.88323	1
622	7893124	---		---	0.00232473	0.00232473	2.75222	2.75222	VVA up vs Control	19.2518	5.12003	2.1276	1
28121	8135033	ENST00000379442 // MUC12 // mucin 12, cell surface associated // 7q22 // 10071 /	MUC12	ENST00000379442	0.0472373	0.0472373	2.75233	2.75233	VVA up vs Control	5.4869	5.12041	7.46564	1
26317	8117435	NM 007047 // BTN3A2 // butyrophilin, subfamily 3, member A2 // 6p22.1 // 11118 /	BTN3A2	NM 007047	0.0121409	0.0121409	2.75412	2.75412	VVA up vs Control	10.4031	5.12701	3.9427	1
14901	8003903	NM 004313 // ARRB2 // arrestin, beta 2 // 17p13 // 409 /// NM 199004 // ARRB2 //	ARRB2	NM 004313	0.0211352	0.0211352	2.75724	2.75724	VVA up vs Control	8.18245	5.13847	5.0239	1
20416	8059672	---		---	0.0137768	0.0137768	2.75741	2.75741	VVA up vs Control	9.86818	5.1391	4.1662	1
8520	7940028	NM 000062 // SERPING1 // serpin peptidase inhibitor, clade G (C1 inhibitor), mem	SERPING1	NM 000062	0.00777907	0.00777907	2.76717	2.76717	VVA up vs Control	12.4316	5.17495	3.33018	1
6237	7917503	NM 018284 // GBP3 // guanylate binding protein 3 // 1p22.2 // 2635 /// ENST00000	GBP3	NM 018284	0.0129747	0.0129747	2.77124	2.77124	VVA up vs Control	10.1198	5.1899	4.10277	1
32872	8178220	NM 002121 // HLA-DPB1 // major histocompatibility complex, class II, DP beta 1 /	HLA-DPB1	NM 002121	0.00504053	0.00504053	2.77672	2.77672	VVA up vs Control	14.6447	5.21005	2.84612	1
11099	7965573	NM 021229 // NTN4 // netrin 4 // 12q22|12q22-q23 // 59277 /// ENST00000343702 //	NTN4	NM 021229	0.0399578	0.0399578	2.78432	2.78432	VVA up vs Control	6.00084	5.23799	6.983	1
21799	8073056	NM 145699 // APOBEC3A // apolipoprotein B mRNA editing enzyme, catalytic polypep	APOBEC3A	NM 145699	0.0270003	0.0270003	2.79215	2.79215	VVA up vs Control	7.29951	5.26675	5.77217	1
30116	8152976	NM 144649 // TMEM71 // transmembrane protein 71 // 8q24.22 // 137835 /// NM 0011	TMEM71	NM 144649	0.0286803	0.0286803	2.80465	2.80465	VVA up vs Control	7.09055	5.31267	5.99408	1
28933	8142540	NM 014888 // FAM3C // family with sequence similarity 3, member C // 7q31 // 104	FAM3C	NM 014888	0.018735	0.018735	2.81014	2.81014	VVA up vs Control	8.6384	5.33282	4.93871	1
10055	7954436	NM 006152 // LRMP // lymphoid-restricted membrane protein // 12p12.1 // 4033 ///	LRMP	NM 006152	0.00549262	0.00549262	2.82125	2.82125	VVA up vs Control	14.1869	5.37365	3.03019	1
15897	8014316	NM 002985 // CCL5 // chemokine (C-C motif) ligand 5 // 17q11.2-q12 // 6352 /// E	CCL5	NM 002985	0.0271524	0.0271524	2.83061	2.83061	VVA up vs Control	7.27993	5.40801	5.94293	1
26319	8117458	NM 001145009 // BTN3A1 // butyrophilin, subfamily 3, member A1 // 6p22.1 // 1111	BTN3A1	NM 001145009	0.00837421	0.00837421	2.83271	2.83271	VVA up vs Control	12.0794	5.41572	3.58674	1
4186	7896712	---		---	0.000375663	0.000375663	2.84116	2.84116	VVA up vs Control	34.4166	5.44675	1.26607	1
10680	7961151	NM 007360 // KLRK1 // killer cell lectin-like receptor subfamily K, member 1 //	KLRK1	NM 007360	0.0457015	0.0457015	2.84421	2.84421	VVA up vs Control	5.58655	5.45797	7.81587	1
25170	8106556	NM 153610 // CMYA5 // cardiomyopathy associated 5 // 5q14.1 // 202333 /// ENST00	CMYA5	NM 153610	0.0307269	0.0307269	2.85268	2.85268	VVA up vs Control	6.856	5.48904	6.40495	1
13512	7989387	NM 020821 // VPS13C // vacuolar protein sorting 13 homolog C (S. cerevisiae) //	VPS13C	NM 020821	0.0305167	0.0305167	2.8568	2.8568	VVA up vs Control	6.87917	5.50418	6.40099	1
31485	8166442	NM 014888 // FAM3C // family with sequence similarity 3, member C // 7q31 // 104	FAM3C	NM 014888	0.0183077	0.0183077	2.86535	2.86535	VVA up vs Control	8.72731	5.53554	5.07423	1
26832	8122279	NM 020340 // KIAA1244 // KIAA1244 // 6q23.3 // 57221 /// ENST00000251691 // KIAA	KIAA1244	NM 020340	0.0092572	0.0092572	2.86802	2.86802	VVA up vs Control	11.6111	5.54535	3.82072	1
25528	8110166	NM 138820 // HIGD2A // HIG1 hypoxia inducible domain family, member 2A // 5q35.2	HIGD2A	NM 138820	0.00165426	0.00165426	2.87705	2.87705	VVA up vs Control	21.581	5.57852	2.06794	1
21108	8066461	NM 006809 // TOMM34 // translocase of outer mitochondrial membrane 34 // --- //	TOMM34	NM 006809	0.00133032	0.00133032	2.89785	2.89785	VVA up vs Control	23.1814	5.65481	1.9515	1
7334	7928306	---		---	0.0147443	0.0147443	2.90435	2.90435	VVA up vs Control	9.58814	5.67864	4.73805	1
22671	8082012	NM 021082 // SLC15A2 // solute carrier family 15 (H+/peptide transporter), membe	SLC15A2	NM 021082	0.0247382	0.0247382	2.91065	2.91065	VVA up vs Control	7.60842	5.70176	5.99521	1
22641	8081710	NM 017699 // SIDT1 // SID1 transmembrane family, member 1 // 3q13.2 // 54847 ///	SIDT1	NM 017699	0.0128946	0.0128946	2.91067	2.91067	VVA up vs Control	10.146	5.70182	4.49581	1
28362	8137264	NM 018487 // TMEM176A // transmembrane protein 176A // 7q36.1 // 55365 /// ENST0	TMEM176A	NM 018487	0.00720334	0.00720334	2.91201	2.91201	VVA up vs Control	12.8061	5.70674	3.56502	1
27020	8124166	NM 001080480 // MBOAT1 // membrane bound O-acyltransferase domain containing 1 /	MBOAT1	NM 001080480	0.0121885	0.0121885	2.91917	2.91917	VVA up vs Control	10.3862	5.73301	4.41585	1
22652	8081826	NM 006952 // UPK1B // uroplakin 1B // 3q13.3-q21 // 7348 /// ENST00000264234 //	UPK1B	NM 006952	0.0350013	0.0350013	2.92358	2.92358	VVA up vs Control	6.42443	5.74919	7.15915	1
10492	7959108	NM 178499 // CCDC60 // coiled-coil domain containing 60 // 12q24.23 // 160777 //	CCDC60	NM 178499	0.0180353	0.0180353	2.93303	2.93303	VVA up vs Control	8.78537	5.78383	5.26678	1
5224	7906757	NM 001136219 // FCGR2A // Fc fragment of IgG, low affinity IIa, receptor (CD32)	FCGR2A	NM 001136219	0.0417511	0.0417511	2.94955	2.94955	VVA up vs Control	5.86375	5.84436	7.97355	1
3339	7895857	---		---	0.00204318	0.00204318	2.95684	2.95684	VVA up vs Control	20.1121	5.87107	2.33534	1
32052	8171172	NM 015419 // MXRA5 // matrix-remodelling associated 5 // Xp22.33 // 25878 /// EN	MXRA5	NM 015419	0.000845086	0.000845086	2.95736	2.95736	VVA up vs Control	26.809	5.87294	1.75253	1
6080	7915787	NM 003629 // PIK3R3 // phosphoinositide-3-kinase, regulatory subunit 3 (gamma) /	PIK3R3	NM 003629	0.0389094	0.0389094	2.97315	2.97315	VVA up vs Control	6.08467	5.93078	7.79767	1
2901	7895417	---		---	0.0181082	0.0181082	2.97338	2.97338	VVA up vs Control	8.76973	5.93162	5.41099	1
5281	7907286	NM 002021 // FMO1 // flavin containing monooxygenase 1 // 1q23-q25 // 2326 /// E	FMO1	NM 002021	0.0062867	0.0062867	2.97806	2.97806	VVA up vs Control	13.4873	5.94877	3.52851	1
15819	8013536	NM 000625 // NOS2 // nitric oxide synthase 2, inducible // 17q11.2-q12 // 4843 /	NOS2	NM 000625	0.0331761	0.0331761	2.98327	2.98327	VVA up vs Control	6.60006	5.96782	7.23365	1
15162	8006531	NM 144975 // SLFN5 // schlafen family member 5 // 17q12 // 162394 /// ENST000002	SLFN5	NM 144975	0.0119093	0.0119093	2.99212	2.99212	VVA up vs Control	10.4861	6.00023	4.57767	1
4783	7902594	NM 182948 // PRKACB // protein kinase, cAMP-dependent, catalytic, beta // 1p36.1	PRKACB	NM 182948	0.00363991	0.00363991	2.99368	2.99368	VVA up vs Control	16.4732	6.00595	2.91671	1
19125	8045664	NM 177964 // LYPD6B // LY6/PLAUR domain containing 6B // 2q23.1-q23.2 // 130576	LYPD6B	NM 177964	0.0175795	0.0175795	2.99488	2.99488	VVA up vs Control	8.88504	6.0103	5.41162	1
29237	8145293	NM 014265 // ADAM28 // ADAM metallopeptidase domain 28 // 8p21.2 // 10863 /// NM	ADAM28	NM 014265	0.00450265	0.00450265	3.01156	3.01156	VVA up vs Control	15.2614	6.07134	3.18258	1
19193	8046380	NM 000210 // ITGA6 // integrin, alpha 6 // 2q31.1 // 3655 /// NM 001079818 // IT	ITGA6	NM 000210	0.0289939	0.0289939	3.01417	3.01417	VVA up vs Control	7.05326	6.0809	6.89713	1
6367	7918857	NM 005725 // TSPAN2 // tetraspanin 2 // 1p13.2 // 10100 /// ENST00000369516 // T	TSPAN2	NM 005725	0.000691491	0.000691491	3.03186	3.03186	VVA up vs Control	28.5495	6.14556	1.72207	1
10632	7960730	NM 005768 // LPCAT3 // lysophosphatidylcholine acyltransferase 3 // 12p13 // 101	LPCAT3	NM 005768	0.00610501	0.00610501	3.03226	3.03226	VVA up vs Control	13.6372	6.14703	3.60603	1
27180	8125537	NM 006120 // HLA-DMA // major histocompatibility complex, class II, DM alpha //	HLA-DMA	NM 006120	0.00381474	0.00381474	3.05457	3.05457	VVA up vs Control	16.2003	6.22855	3.07577	1
8364	7938758	NM 000331 // SAA1 // serum amyloid A1 // 11p15.1 // 6288 /// NM 199161 // SAA1 /	SAA1	NM 000331	0.0448097	0.0448097	3.06185	3.06185	VVA up vs Control	5.64639	6.25513	8.86249	1
20246	8057677	NM 014585 // SLC40A1 // solute carrier family 40 (iron-regulated transporter), m	SLC40A1	NM 014585	0.000918146	0.000918146	3.06781	3.06781	VVA up vs Control	26.1148	6.27686	1.92285	1
5622	7911155	NM 016002 // SCCPDH // saccharopine dehydrogenase (putative) // 1q44 // 51097 //	SCCPDH	NM 016002	0.0162692	0.0162692	3.08466	3.08466	VVA up vs Control	9.19073	6.33836	5.51717	1
28225	8136095	NM 015328 // AHCYL2 // adenosylhomocysteinase-like 2 // 7q32.1 // 23382 /// NM 0	AHCYL2	NM 015328	0.022878	0.022878	3.1094	3.1094	VVA up vs Control	7.89052	6.4286	6.5178	1
23097	8086344	NM 001337 // CX3CR1 // chemokine (C-X3-C motif) receptor 1 // 3p21|3p21.3 // 152	CX3CR1	NM 001337	0.00114424	0.00114424	3.1107	3.1107	VVA up vs Control	24.3404	6.43334	2.11445	1
32943	8178884	NM 006120 // HLA-DMA // major histocompatibility complex, class II, DM alpha //	HLA-DMA	NM 006120	0.0036298	0.0036298	3.11898	3.11898	VVA up vs Control	16.4895	6.46349	3.13582	1
33065	8180086	NM 006120 // HLA-DMA // major histocompatibility complex, class II, DM alpha //	HLA-DMA	NM 006120	0.0036298	0.0036298	3.11898	3.11898	VVA up vs Control	16.4895	6.46349	3.13582	1
5278	7907249	NM 006894 // FMO3 // flavin containing monooxygenase 3 // 1q23-q25 // 2328 /// N	FMO3	NM 006894	0.0153123	0.0153123	3.16239	3.16239	VVA up vs Control	9.43431	6.62154	5.61486	1
5373	7908488	NM 002113 // CFHR1 // complement factor H-related 1 // 1q32 // 3078 /// BC107771	CFHR1	NM 002113	0.00901286	0.00901286	3.17768	3.17768	VVA up vs Control	11.7349	6.67712	4.55198	1
24020	8095751	NM 015393 // DKFZP564O0823 // prostatic androgen-repressed message-1 // 4q13.3-q	DKFZP564O0823	NM 015393	0.0042399	0.0042399	3.18949	3.18949	VVA up vs Control	15.5973	6.72004	3.44676	1
15638	8011713	NM 022059 // CXCL16 // chemokine (C-X-C motif) ligand 16 // 17p13 // 58191 /// N	CXCL16	NM 022059	0.00503863	0.00503863	3.1995	3.1995	VVA up vs Control	14.6467	6.7564	3.69033	1
28748	8140534	NM 006379 // SEMA3C // sema domain, immunoglobulin domain (Ig), short basic doma	SEMA3C	NM 006379	0.0393557	0.0393557	3.203	3.203	VVA up vs Control	6.04864	6.76913	8.95292	1
23940	8095139	NM 024592 // SRD5A3 // steroid 5 alpha-reductase 3 // 4q12 // 79644 /// ENST0000	SRD5A3	NM 024592	0.0134479	0.0134479	3.20947	3.20947	VVA up vs Control	9.96909	6.79261	5.45094	1
16533	8020779	NM 001943 // DSG2 // desmoglein 2 // 18q12.1 // 1829 /// ENST00000261590 // DSG2	DSG2	NM 001943	0.00657155	0.00657155	3.22475	3.22475	VVA up vs Control	13.2629	6.84805	4.13064	1
27296	8126750	NM 021572 // ENPP5 // ectonucleotide pyrophosphatase/phosphodiesterase 5 (putati	ENPP5	NM 021572	0.00545576	0.00545576	3.27996	3.27996	VVA up vs Control	14.2225	7.04808	3.96448	1
25208	8106986	NM 014899 // RHOBTB3 // Rho-related BTB domain containing 3 // 5q15 // 22836 ///	RHOBTB3	NM 014899	0.0093685	0.0093685	3.2862	3.2862	VVA up vs Control	11.5561	7.07064	4.89485	1
18518	8040103	NM 002166 // ID2 // inhibitor of DNA binding 2, dominant negative helix-loop-hel	ID2	NM 002166	0.00598278	0.00598278	3.33188	3.33188	VVA up vs Control	13.7412	7.23567	4.21253	1
12667	7981142	NM 024734 // CLMN // calmin (calponin-like, transmembrane) // 14q32.13 // 79789	CLMN	NM 024734	0.0279848	0.0279848	3.33444	3.33444	VVA up vs Control	7.17512	7.24491	8.07781	1
27525	8129254	NM 005907 // MAN1A1 // mannosidase, alpha, class 1A, member 1 // 6q22 // 4121 //	MAN1A1	NM 005907	0.00199098	0.00199098	3.36495	3.36495	VVA up vs Control	20.2879	7.3549	2.90021	1
26009	8114612	NM 000591 // CD14 // CD14 molecule // 5q22-q32|5q31.1 // 929 /// NM 001040021 //	CD14	NM 000591	0.0186419	0.0186419	3.39626	3.39626	VVA up vs Control	8.65755	7.46763	6.90045	1
4778	7902541	NM 006820 // IFI44L // interferon-induced protein 44-like // 1p31.1 // 10964 ///	IFI44L	NM 006820	0.00321424	0.00321424	3.39818	3.39818	VVA up vs Control	17.2126	7.47453	3.47399	1
30455	8156199	NM 004938 // DAPK1 // death-associated protein kinase 1 // 9q34.1 // 1612 /// EN	DAPK1	NM 004938	0.00816957	0.00816957	3.41373	3.41373	VVA up vs Control	12.1969	7.53043	4.93925	1
4414	7898805	NM 000491 // C1QB // complement component 1, q subcomponent, B chain // 1p36.12	C1QB	NM 000491	0.00127964	0.00127964	3.44358	3.44358	VVA up vs Control	23.476	7.63761	2.6027	1
20146	8056113	NM 002349 // LY75 // lymphocyte antigen 75 // 2q24 // 4065 /// AF011333 // LY75	LY75	NM 002349	0.00457459	0.00457459	3.47125	3.47125	VVA up vs Control	15.1737	7.73679	4.07905	1
6241	7917530	---		---	0.00379629	0.00379629	3.50011	3.50011	VVA up vs Control	16.2283	7.84006	3.86488	1
21450	8069795	NM 199328 // CLDN8 // claudin 8 // 21q22.11 // 9073 /// ENST00000399899 // CLDN8	CLDN8	NM 199328	0.046178	0.046178	3.50985	3.50985	VVA up vs Control	5.55519	7.87487	11.3406	1
10651	7960874	NM 004054 // C3AR1 // complement component 3a receptor 1 // 12p13.31 // 719 ///	C3AR1	NM 004054	0.0266289	0.0266289	3.51366	3.51366	VVA up vs Control	7.34794	7.88849	8.58852	1
31225	8163896	NM 004099 // STOM // stomatin // 9q34.1 // 2040 /// NM 198194 // STOM // stomati	STOM	NM 004099	0.0125355	0.0125355	3.53178	3.53178	VVA up vs Control	10.266	7.95321	6.19768	1
4413	7898799	NM 001114101 // C1QC // complement component 1, q subcomponent, C chain // 1p36.	C1QC	NM 001114101	0.00735465	0.00735465	3.53646	3.53646	VVA up vs Control	12.7041	7.96992	5.01879	1
8272	7938035	NM 006074 // TRIM22 // tripartite motif-containing 22 // 11p15 // 10346 /// ENST	TRIM22	NM 006074	0.0190095	0.0190095	3.55491	3.55491	VVA up vs Control	8.58262	8.03571	7.49022	1
4648	7901256	NM 001099772 // CYP4B1 // cytochrome P450, family 4, subfamily B, polypeptide 1	CYP4B1	NM 001099772	0.000643031	0.000643031	3.56439	3.56439	VVA up vs Control	29.202	8.06951	2.21068	1
5220	7906742	NM 001102566 // PCP4L1 // Purkinje cell protein 4 like 1 // 1q23.3 // 654790 ///	PCP4L1	NM 001102566	2.70E-05	2.70E-05	3.56518	3.56518	VVA up vs Control	73.0649	8.0723	0.88385	1
13055	7984263	NM 016395 // PTPLAD1 // protein tyrosine phosphatase-like A domain containing 1	PTPLAD1	NM 016395	0.024413	0.024413	3.58042	3.58042	VVA up vs Control	7.65577	8.12658	8.49198	1
5034	7905047	NM 000566 // FCGR1A // Fc fragment of IgG, high affinity Ia, receptor (CD64) //	FCGR1A	NM 000566	0.0288457	0.0288457	3.64323	3.64323	VVA up vs Control	7.07081	8.34971	9.44696	1
21317	8068383	NM 053277 // CLIC6 // chloride intracellular channel 6 // 21q22.12 // 54102 ///	CLIC6	NM 053277	0.00444249	0.00444249	3.7044	3.7044	VVA up vs Control	15.3361	8.56613	4.46847	1
19665	8051573	NM 006449 // CDC42EP3 // CDC42 effector protein (Rho GTPase binding) 3 // 2p21 /	CDC42EP3	NM 006449	0.0175869	0.0175869	3.71346	3.71346	VVA up vs Control	8.88339	8.59812	7.7431	1
24047	8096070	NM 001201 // BMP3 // bone morphogenetic protein 3 // 4q21 // 651 /// ENST0000028	BMP3	NM 001201	0.0137569	0.0137569	3.74298	3.74298	VVA up vs Control	9.87419	8.70222	7.05048	1
9428	7948444	NM 001062 // TCN1 // transcobalamin I (vitamin B12 binding protein, R binder fam	TCN1	NM 001062	0.0360096	0.0360096	3.75822	3.75822	VVA up vs Control	6.33231	8.75589	11.0618	1
29761	8149927	NM 001831 // CLU // clusterin // 8p21-p12 // 1191 /// NM 203339 // CLU // cluste	CLU	NM 001831	0.00252947	0.00252947	3.7967	3.7967	VVA up vs Control	18.7043	8.89117	3.80282	1
30456	8156228	NM 001912 // CTSL1 // cathepsin L1 // 9q21-q22 // 1514 /// NM 145918 // CTSL1 //	CTSL1	NM 001912	0.0101531	0.0101531	3.81558	3.81558	VVA up vs Control	11.19	8.95738	6.40382	1
6712	7922174	NM 000130 // F5 // coagulation factor V (proaccelerin, labile factor) // 1q23 //	F5	NM 000130	0.0253054	0.0253054	3.88646	3.88646	VVA up vs Control	7.52771	9.20534	9.78288	1
17803	8033257	NM 000064 // C3 // complement component 3 // 19p13.3-p13.2 // 718 /// ENST000002	C3	NM 000064	0.0124936	0.0124936	3.89349	3.89349	VVA up vs Control	10.2804	9.22985	7.18252	1
28929	8142524	NM 012338 // TSPAN12 // tetraspanin 12 // 7q31.31 // 23554 /// ENST00000222747 /	TSPAN12	NM 012338	0.00371991	0.00371991	3.93654	3.93654	VVA up vs Control	16.3463	9.37978	4.59053	1
6214	7917199	NM 024686 // TTLL7 // tubulin tyrosine ligase-like family, member 7 // 1p31.1 //	TTLL7	NM 024686	0.014885	0.014885	3.95848	3.95848	VVA up vs Control	9.54933	9.45601	7.92182	1
32325	8173745	NM 006639 // CYSLTR1 // cysteinyl leukotriene receptor 1 // Xq13.2-q21.1 // 1080	CYSLTR1	NM 006639	0.0142698	0.0142698	3.97693	3.97693	VVA up vs Control	9.7225	9.52004	7.83341	1

Column #	Transcript Cluster ID	gene_assignment	Gene Symbol	RefSeq	p-value(Group)	p-value(VVA vs. Control)	Ratio(VVA vs. Control)	Fold-Change(VVA vs. Control)	Fold-Change(VVA vs. Control) (Description)	F(Group)	SS(Group)	SS(Error)	F(Error)
7232	7927215	NM 000698 // ALOX5 // arachidonate 5-lipoxygenase // 10q11.2 // 240 /// ENST0000	ALOX5	NM 000698	0.00215357	0.00215357	4.0618	4.0618	VVA up vs Control	19.758	9.8135	3.97347	1
24013	8095697	NM 001511 // CXCL1 // chemokine (C-X-C motif) ligand 1 (melanoma growth stimulat	CXCL1	NM 001511	0.0208458	0.0208458	4.1128	4.1128	VVA up vs Control	8.23387	9.98903	9.70531	1
25823	8112980	NM 005711 // EDIL3 // EGF-like repeats and discoidin I-like domains 3 // 5q14 //	EDIL3	NM 005711	0.0104406	0.0104406	4.1419	4.1419	VVA up vs Control	11.0647	10.0889	7.29444	1
2778	7895294	---		---	0.0247558	0.0247558	4.17242	4.17242	VVA up vs Control	7.60589	10.1934	10.7216	1
31924	8170179	NM 016267 // VGLL1 // vestigial like 1 (Drosophila) // Xq26.3 // 51442 /// ENST0	VGLL1	NM 016267	0.00314646	0.00314646	4.17335	4.17335	VVA up vs Control	17.3417	10.1966	4.70386	1
6937	7924450	NM 007207 // DUSP10 // dual specificity phosphatase 10 // 1q41 // 11221 /// NM 1	DUSP10	NM 007207	0.0120063	0.0120063	4.20147	4.20147	VVA up vs Control	10.4511	10.2927	7.87874	1
6379	7918936	NM 024626 // VTCN1 // V-set domain containing T cell activation inhibitor 1 // 1	VTCN1	NM 024626	0.000781297	0.000781297	4.26249	4.26249	VVA up vs Control	27.4796	10.5004	3.05694	1
24711	8102328	NM 000204 // CFI // complement factor I // 4q25 // 3426 /// ENST00000394634 // C	CFI	NM 000204	0.000981277	0.000981277	4.34863	4.34863	VVA up vs Control	25.5684	10.7923	3.37676	1
15946	8014903	NM 001042471 // GSDMB // gasdermin B // 17q12 // 55876 /// NM 018530 // GSDMB //	GSDMB	NM 001042471	0.00420067	0.00420067	4.36206	4.36206	VVA up vs Control	15.6497	10.8376	5.54007	1
27560	8129637	NM 004665 // VNN2 // vanin 2 // 6q23-q24 // 8875 /// NM 078488 // VNN2 // vanin	VNN2	NM 004665	0.0322713	0.0322713	4.36472	4.36472	VVA up vs Control	6.69173	10.8466	12.9671	1
6240	7917528	---		---	0.00151142	0.00151142	4.39448	4.39448	VVA up vs Control	22.2335	10.9468	3.93886	1
28607	8139488	NM 001013398 // IGFBP3 // insulin-like growth factor binding protein 3 // 7p13-p	IGFBP3	NM 001013398	0.0126214	0.0126214	4.52008	4.52008	VVA up vs Control	10.237	11.3676	8.88357	1
21769	8072735	NM 145343 // APOL1 // apolipoprotein L, 1 // 22q13.1 // 8542 /// NM 003661 // AP	APOL1	NM 145343	0.00630136	0.00630136	4.63164	4.63164	VVA up vs Control	13.4755	11.738	6.96852	1
16739	8022666	NM 031422 // CHST9 // carbohydrate (N-acetylgalactosamine 4-0) sulfotransferase	CHST9	NM 031422	0.00331626	0.00331626	4.63306	4.63306	VVA up vs Control	17.0246	11.7427	5.51798	1
7316	7928046	NM 012339 // TSPAN15 // tetraspanin 15 // 10q22.1 // 23555 /// ENST00000373290 /	TSPAN15	NM 012339	0.000627128	0.000627128	4.74166	4.74166	VVA up vs Control	29.4296	12.1003	3.28928	1
9665	7950810	NM 206927 // SYTL2 // synaptotagmin-like 2 // 11q14 // 54843 /// NM 206928 // SY	SYTL2	NM 206927	0.019227	0.019227	4.79767	4.79767	VVA up vs Control	8.53912	12.2836	11.508	1
8605	7940775	NM 004585 // RARRES3 // retinoic acid receptor responder (tazarotene induced) 3	RARRES3	NM 004585	0.00369514	0.00369514	4.82656	4.82656	VVA up vs Control	16.3852	12.3778	6.04339	1
28119	8135015	AB038783 // MUC3B // mucin 3B, cell surface associated // 7q22 // 57876 /// AF00	MUC3B	AB038783	0.0164248	0.0164248	4.9025	4.9025	VVA up vs Control	9.15284	12.6245	11.0344	1
23594	8091723	NM 206963 // RARRES1 // retinoic acid receptor responder (tazarotene induced) 1	RARRES1	NM 206963	0.0166452	0.0166452	5.00433	5.00433	VVA up vs Control	9.09993	12.9532	11.3875	1
24035	8095886	NM 006419 // CXCL13 // chemokine (C-X-C motif) ligand 13 // 4q21 // 10563 /// EN	CXCL13	NM 006419	0.0209222	0.0209222	5.17779	5.17779	VVA up vs Control	8.2202	13.5072	13.1453	1
30080	8152617	NM 005328 // HAS2 // hyaluronan synthase 2 // 8q24.12 // 3037 /// ENST0000030392	HAS2	NM 005328	0.00690095	0.00690095	5.30686	5.30686	VVA up vs Control	13.0182	13.9147	8.55092	1
25851	8113214	NM 002064 // GLRX // glutaredoxin (thioltransferase) // 5q14 // 2745 /// NM 0011	GLRX	NM 002064	0.00357609	0.00357609	5.3636	5.3636	VVA up vs Control	16.5769	14.0926	6.80104	1
24628	8101429	NM 016619 // PLAC8 // placenta-specific 8 // 4q21.22 // 51316 /// NM 001130716 /	PLAC8	NM 016619	0.0202948	0.0202948	5.51333	5.51333	VVA up vs Control	8.33431	14.5584	13.9744	1
30689	8158671	NM 000050 // ASS1 // argininosuccinate synthetase 1 // 9q34.1 // 445 /// NM 0540	ASS1	NM 000050	0.00305059	0.00305059	5.70001	5.70001	VVA up vs Control	17.5303	15.1319	6.90547	1
33020	8179617	NM 007028 // TRIM31 // tripartite motif-containing 31 // 6p21.3 // 11074 /// ENS	TRIM31	NM 007028	0.0476641	0.0476641	5.84906	5.84906	VVA up vs Control	5.45995	15.584	22.834	1
28469	8138381	NM 006408 // AGR2 // anterior gradient homolog 2 (Xenopus laevis) // 7p21.3 // 1	AGR2	NM 006408	0.0150326	0.0150326	6.17577	6.17577	VVA up vs Control	9.50915	16.5579	13.9301	1
4639	7901175	NM 005727 // TSPAN1 // tetraspanin 1 // 1p34.1 // 10103 /// ENST00000372003 // T	TSPAN1	NM 005727	0.0246151	0.0246151	6.29974	6.29974	VVA up vs Control	7.62626	16.9214	17.7507	1
24011	8095688	NM 002993 // CXCL6 // chemokine (C-X-C motif) ligand 6 (granulocyte chemotactic	CXCL6	NM 002993	0.0180624	0.0180624	6.50333	6.50333	VVA up vs Control	8.77954	17.5113	15.9565	1
20745	8063000	NM 006103 // WFDC2 // WAP four-disulfide core domain 2 // 20q12-q13.2 // 10406 /	WFDC2	NM 006103	0.00832392	0.00832392	6.61473	6.61473	VVA up vs Control	12.108	17.8304	11.781	1
26440	8118345	NM 001710 // CFB // complement factor B // 6p21.3 // 629 /// BC004143 // CFB //	CFB	NM 001710	0.0477693	0.0477693	6.93188	6.93188	VVA up vs Control	5.45335	18.7253	27.4699	1
28482	8138487	---		---	0.00208	0.00208	7.09501	7.09501	VVA up vs Control	19.9914	19.178	7.6745	1
29824	8150509	NM 000930 // PLAT // plasminogen activator, tissue // 8p12 // 5327 /// NM 033011	PLAT	NM 000930	0.0222861	0.0222861	7.19491	7.19491	VVA up vs Control	7.98642	19.4527	19.4857	1
32863	8178115	NM 001710 // CFB // complement factor B // 6p21.3 // 629 /// BC004143 // CFB //	CFB	NM 001710	0.0469248	0.0469248	7.32622	7.32622	VVA up vs Control	5.50684	19.8109	28.78	1
33185	8180303	---		---	0.0225969	0.0225969	7.72813	7.72813	VVA up vs Control	7.93568	20.8877	21.057	1
30690	8158684	---		---	0.00949802	0.00949802	8.28307	8.28307	VVA up vs Control	11.493	22.3284	15.5422	1
5442	7909250	NM 153758 // IL19 // interleukin 19 // 1q32.2 // 29949 /// NM 013371 // IL19 //	IL19	NM 153758	0.0324829	0.0324829	8.3067	8.3067	VVA up vs Control	6.67001	22.3886	26.8529	1
6887	7923929	NM 002644 // PIGR // polymeric immunoglobulin receptor // 1q31-q41 // 5284 /// E	PIGR	NM 002644	0.0200583	0.0200583	8.63577	8.63577	VVA up vs Control	8.37849	23.2179	22.169	1
5371	7908459	NM 000186 // CFH // complement factor H // 1q32 // 3075 /// NM 001014975 // CFH	CFH	NM 000186	0.00479345	0.00479345	8.71593	8.71593	VVA up vs Control	14.9172	23.4173	12.5586	1
27150	8125149	NM 025257 // SLC44A4 // solute carrier family 44, member 4 // 6p21.3 // 80736 //	SLC44A4	NM 025257	0.0222679	0.0222679	9.19378	9.19378	VVA up vs Control	7.98943	24.5861	24.6186	1
32922	8178653	NM 025257 // SLC44A4 // solute carrier family 44, member 4 // 6p21.3 // 80736 //	SLC44A4	NM 025257	0.0222679	0.0222679	9.19378	9.19378	VVA up vs Control	7.98943	24.5861	24.6186	1
33047	8179861	NM 025257 // SLC44A4 // solute carrier family 44, member 4 // 6p21.3 // 80736 //	SLC44A4	NM 025257	0.0222679	0.0222679	9.19378	9.19378	VVA up vs Control	7.98943	24.5861	24.6186	1
9714	7951217	NM 002423 // MMP7 // matrix metallopeptidase 7 (matrilysin, uterine) // 11q21-q2	MMP7	NM 002423	0.00772885	0.00772885	13.7483	13.7483	VVA up vs Control	12.4629	34.3135	22.0261	1
